# Genome-wide identification and expression profiling analysis of sucrose synthase (SUS) and sucrose phosphate synthase (SPS) genes family in *Actinidia chinensis* and *A. eriantha*

**DOI:** 10.1186/s12870-022-03603-y

**Published:** 2022-04-26

**Authors:** Guanglian Liao, Yiqi Li, Hailing Wang, Qing Liu, Min Zhong, Dongfeng Jia, Chunhui Huang, Xiaobiao Xu

**Affiliations:** 1 College of Forestry, Jiangxi Provincial Key Laboratory of Silviculture, Jiangxi Agricultural University, 330045 Nanchang Jiangxi, P. R. China; 2 College of Agronomy, Jiangxi Agricultural University, Kiwifruit Institute of Jiangxi Agricultural University, 330045 Nanchang Jiangxi, P. R. China

**Keywords:** *Actinidia*, Sucrose synthase, Sucrose phosphate synthase, Gene, Bioinformatics analysis, Expression profiling analysis

## Abstract

Sucrose synthase (SUS) is a common sugar-base transfer enzyme in plants, and sucrose phosphate synthase (SPS) is one of the major enzymes in higher plants that regulates sucrose synthesis. However, information of the SPS and SUS gene families in *Actinidia*, as well as their evolutionary and functional properties, is limited. According to the SPS and SUS proteins conserved domain of *Arabidopsis thaliana*, we found 6 SPS genes and 6 SUS genes from *A. chinensis* (cultivar: ‘Hongyang’), and 3 SPS genes and 6 SUS genes from *A. eriantha* (cultivar: ‘White’). The novel CDC50 conserved domains were discovered on AcSUS2, and all members of the gene family contain similar distinctive conserved domains. The majority of SUS and SPS proteins were hydrophilic, lipid-soluble enzymes that were expected to be found in the cytoplasm. The tertiary structure of SPS and SUS protein indicated that there were many tertiary structures in SPS, and there were windmill-type and spider-type tertiary structures in SUS. The phylogenetic tree was created using the neighbor-joining method, and members of the SPS and SUS gene families are grouped into three subgroups. Genes with comparable intron counts, conserved motifs, and phosphorylation sites were clustered together first. SPS and SUS were formed through replication among their own family members. *AcSPS1*, *AcSPS2*, *AcSPS4*, *AcSPS5*, *AcSUS5*, *AcSUS6*, *AeSPS3*, *AeSUS3* and *AeSUS4* were the important genes in regulating the synthesis and accumulation of sucrose for *Actinidia* during the fruit growth stages.

**Supplementary information:**

The online version contains supplementary material available at 10.1186/s12870-022-03603-y.

## Background

Key enzymes involved in sucrose production and accumulation include sucrose synthase (SUS, EC 2.4.1.13) and sucrose phosphate synthase (SPS, EC 2.4.1.14). SPS catalyzes the conversion of uridine diphosphate glucose (UDPG) and fructose-6-phosphoric acid (F6P) to sucrose-6-phosphoric acid (S6P), which is then irreversibly converted to sucrose by sucrose phosphatase (SPP) [1]. SPS is critical in the accumulation of sucrose because the direction is irreversible. SUS, on the other hand, is a reversible enzyme that allows sucrose to engage in a variety of metabolic activities, including tissue formation, material storage, and plant cell metabolism [2, 3]. A large number of studies have shown that sucrose accumulation during fruit development was closely related to the increased activity of SUS and SPS [4–6]. As a result, identifying and analyzing SPS and SUS genes can give a theoretical foundation for plant growth and development as well as fruit quality formation.

*SUS* and *SPS* genes have been cloned in many species since *SUS* and *SPS* were first discovered in wheat germ in 1955 [7]. For example, *SUS* genes was cloned in carrot [8], Arabidopsis [9], sugarcane [10], citrus [11] and strawberry [12]. *SPS* genes were cloned in corn [13], apple [14], rice [15] and orange [16]. In recent years, an increasing amount of genome-wide data has been released, and SUS and SPS genes were discovered to exist in plants as a family. But the number of members varies greatly between different species, for example, peaches have 6 *SUS* genes [17], grapes have 5 *SUS* genes [18], oranges have 4 *SPS* genes [19] and pears have 17 *SUS* genes and 8 *SPS* genes [20]. Despite the fact that the number of SPS and SUS genes in various plants varies, their protein sequences are similar and contain certain distinct domains. SUS is thought to have conservative domains for sucrose-synth and glycos-transf-1, whereas SPS contains S6PP conservative domains in addition to the above two conservative domains. Furthermore, the expression features and roles of members of the gene family vary. OsSPS1 was found to be expressed preferentially in “source” organs, whereas OsSPS2, OsSPS6, and OsSPS8 were found in both “source” and “library” organs [21].

Kiwifruit is one of the domesticated fruit crops from the last century [22], which originated in China and is widely cultivated in New Zealand, Chile, Italy, Consumers are attracted to its fruit, flesh color, and nutrition, particularly A. eriantha, which has high vitamin C and other nutrients in its fruit. The genomes of *Actinidia chinensis* and *A. eriantha* have been sequenced in recent years [23, 24]. Benefitting from the publication of the genome of kiwifruit, a large number of functional genes and their gene family members involved in ascorbic acid, anthocyanin and resistance [25] had been reported. However, in-depth analysis of the genome data will be necessary. Previous research has discovered that sucrose makes up the majority of the sugar in *A. eriantha* [26], however, the relationship between fruit sucrose accumulation and *SUS*, *SPS* genes is still unclear. In order to understand the characteristics of the *SUS* and *SPS* family members of kiwifruit and their role in the sucrose accumulation, genome-wide identification and sequencing analysis of SUS and SPS genes in kiwifruit were performed. SUS and SPS were subjected to bioinformatics analysis, and the expression of genes in fruit at different growth stages was measured using qRT-PCR. Our findings pave the way for further research into the molecular mechanisms of sucrose accumulation in *Actinidia*.

## Results

### Identification of SPS and SUS gene families in kiwifruit

6 *AcSPS* genes, 6 *AcSUS*, 3 *AeSPS* and 6 *AeSUS* genes were discovered after searching the Kiwifruit Genome Database (*A. chinensis* ‘Hongyang’ and *A.eriantha* ‘White’). These genes were named AcSPS1-AcSPS6, AcSUS1-AcSUS6, AeSPS1-AcSPS3, and AeSUS1-AeSUS6, respectively. Table 1 contains detailed information on these genes, including their location and subcellular localization predictions. Except for AcSPS3, AcSPS6, AcSUS1, AcSUS5, and AcSUS6, which are irregularly distributed on chromosomes 5, 6, 10, 12, 13, 20, 21, 23, 26, and 28. According to the results of subcellular predictive localization, all the other SPS and SUS genes were located in cytoplasmic except AeSPS1 in nuclear, AcSUS2 in outer membrane and AeSUS1 in chloroplast.

**Fig. 1:**
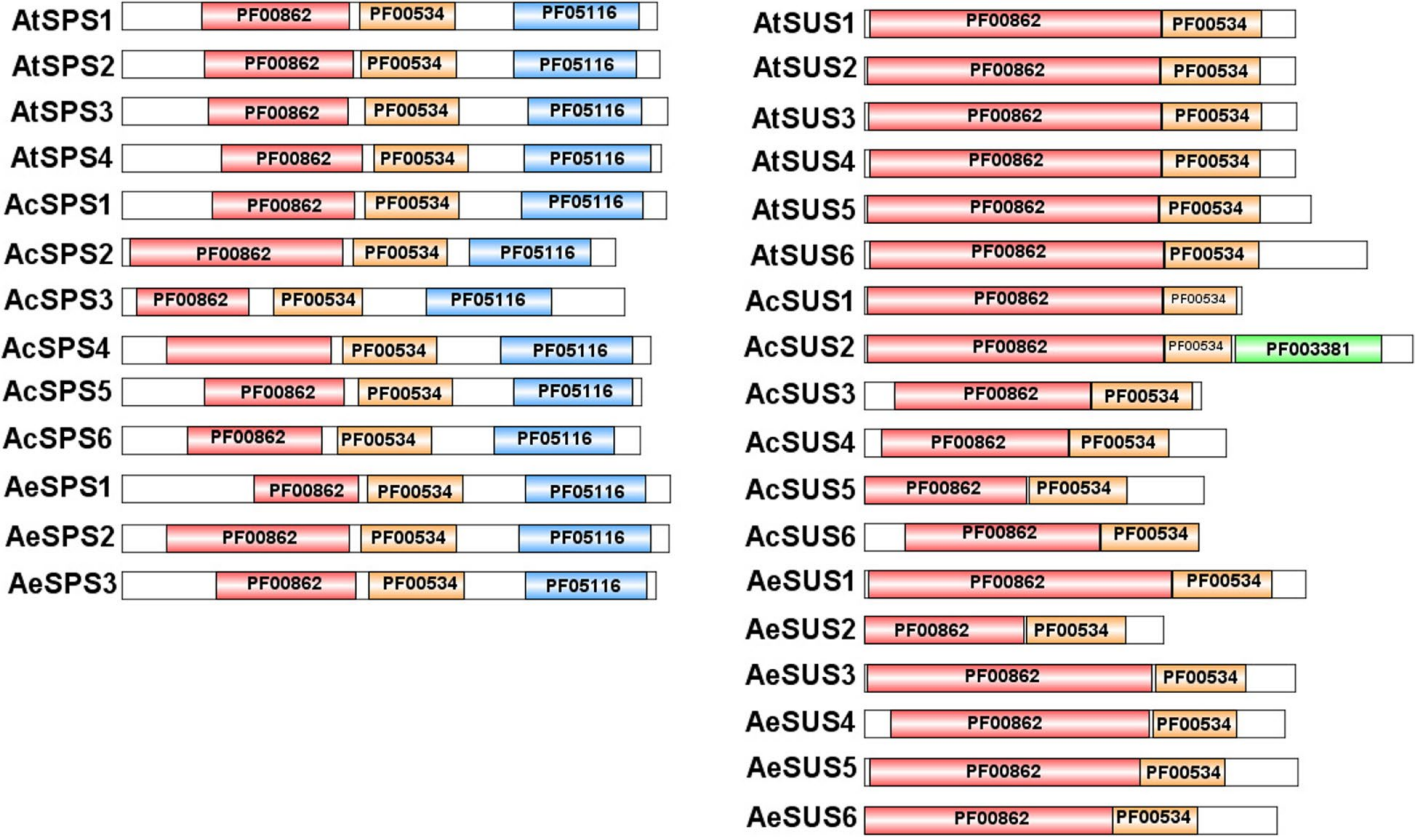
Protein conservative domains of the SUS and SPS gene families in *Actinidia*. The different colored boxes represent different protein sequences, the white box represents the full length of the protein, the red box represents the sucrose synthase domain (ID: PF00862), the yellow box represents the Glycose-transf-1 domain (ID: PF00534), the bule box represents the S6PP domain (ID: PF05116), and the green box represents the CDC50 domain (ID: PF00381)

As shown in Fig. 1, all SPS proteins obtained from the kiwifruit contain characteristic conserved domains PF00862, PF00534 and PF05116 contained in the SPS gene family, and all SUS proteins obtained from the kiwifruit contain characteristic conserved domains PF00862 and PF00534. It is worth noting that AcSUS2 has expanded out the new domain CDC50 (PF00381).

**Table 1:** Detailed information of SPS and SUS gene families in *Actinidia*

Genes name	Genes ID	Location	Subcellular predictive localization
*AcSPS1*	Ach13g383801.2	Chr 13: 11,492,092–11,507,975	Cytoplasmic
*AcSPS2*	Ach06g074871.2	Chr 6: 12,965,825–12,984,875	Cytoplasmic
*AcSPS3*	Ach00g065491.2	Chr 0: 26,451,767–26,460,535	Cytoplasmic
*AcSPS4*	Ach06g354691	Chr 6: 8,830,939–8,839,218	Cytoplasmic
*AcSPS5*	Ach10g218701	Chr 10: 5,212,785–5,222,867	Cytoplasmic
*AcSPS6*	Ach00g471611.2	Chr 0: 103,042,227–103,058,005	Cytoplasmic
*AcSUS1*	Ach00g335801.2	Chr 0: 87,008,273–87,018,638	Cytoplasmic
*AcSUS2*	Ach21g388531.2	Chr 21: 1,615,680–1,625,811	Outer membrane
*AcSUS3*	Ach23g024141.2	Chr 23: 20,279,444–20,286,507	Cytoplasmic
*AcSUS4*	Ach12g167901.2	Chr 12: 12,786,216–12,791,743	Cytoplasmic
*AcSUS5*	Ach00g240251	Chr 0: 61,503,551–61,505,984	Cytoplasmic
*AcSUS6*	Ach00g318231.2	Chr 0: 84,918,425–84,925,401	Cytoplasmic
*AeSPS1*	DTZ79_13g06220	Chr 13: 6,047,439–6,061,745	Nuclear
*AeSPS2*	DTZ79_06g05460	Chr 6: 7,916,729–7,927,173	Cytoplasmic
*AeSPS3*	DTZ79_10g06570	Chr 10: 13,619,623–13,629,990	Cytoplasmic
*AeSUS1*	DTZ79_20g14180	Chr 20: 21,233,113–21,238,518	Chloroplast
*AeSUS2*	DTZ79_12g00380	Chr 12: 402,170–407,809	Cytoplasmic
*AeSUS3*	DTZ79_21g10250	Chr 21: 15,292,975–15,297,928	Cytoplasmic
*AeSUS4*	DTZ79_05g01940	Chr 05: 2,860,524–2,865,544	Cytoplasmic
*AeSUS5*	DTZ79_26g10540	Chr 26: 17,935,857–17,940,483	Cytoplasmic
*AeSUS6*	DTZ79_28g13300	Chr 28: 19,821,275–19,825,741	Cytoplasmic

### Physicochemical properties analysis

The analysis of physicochemical properties analysis showed that the number of amino acids, molecular weight, isoelectric point in SPS were 961–1068, 108065.21-120006.62 Da and 5.98–7.92, respectively, in SUS were 557–1027, 64009.90-116585.04 and 5.62–8.82, respectively (Table 2). In addition, all SUS and SPS proteins were lipid soluble, hydrophilicity proteins. The biggest difference between SUS and SPS proteins was in stability, all SPS proteins were unstable proteins, while SUS was stable proteins.

**Table 2:** Physicochemical properties of SPS and SUS gene family proteins. Instability index more than 40 means unstable; aliphatic index less than 100 means lipid soluble protein; value of grand average of hydrophobicity being positive means hydrophobicity, while negative means hydrophilicity

Genes name	No. of amino acids	Molecular weight(Da)	Isoelectric point	Instability index	Aliphatic index	Grand average of hydrophobicity
AcSPS1	1061	119363.67	6.12	44.75	86.66	-0.417
AcSPS2	961	108065.21	6.27	44.74	90.09	-0.340
AcSPS3	979	108879.28	7.92	42.45	84.04	-0.378
AcSPS4	1029	116473.64	6.28	45.19	83.02	-0.500
AcSPS5	1012	114451.41	6.03	47.09	86.43	-0.434
AcSPS6	1009	113275.63	6.07	46.81	85.14	-0.467
AcSUS1	707	79655.45	5.84	35.81	91.30	-0.188
AcSUS2	1027	116585.04	8.84	39.10	86.11	-0.295
AcSUS3	678	77296.51	5.62	34.96	91.46	-0.226
AcSUS4	635	73631.83	6.74	37.38	87.02	-0.305
AcSUS5	557	64009.90	6.23	37.18	91.69	-0.206
AcSUS6	674	76659.80	5.82	34.50	93.01	-0.202
AeSPS1	1068	119880.10	5.98	41.90	85.64	-0.387
AeSPS2	1065	120006.62	6.23	45.19	83.50	-0.469
AeSPS3	1039	117934.47	6.81	46.76	84.37	-0.472
AeSUS1	827	95135.86	6.13	35.71	94.40	-0.183
AeSUS2	559	64055.08	6.77	36.49	90.48	-0.145
AeSUS3	806	91434.90	6.66	35.52	85.78	-0.294
AeSUS4	787	89046.69	7.56	34.59	81.16	-0.379
AeSUS5	812	91789.47	8.04	37.26	87.60	-0.243
AeSUS6	773	87788.68	6.75	34.86	85.94	-0.255

### Prediction of secondary and tertiary structure of proteins

**Fig. 2:**
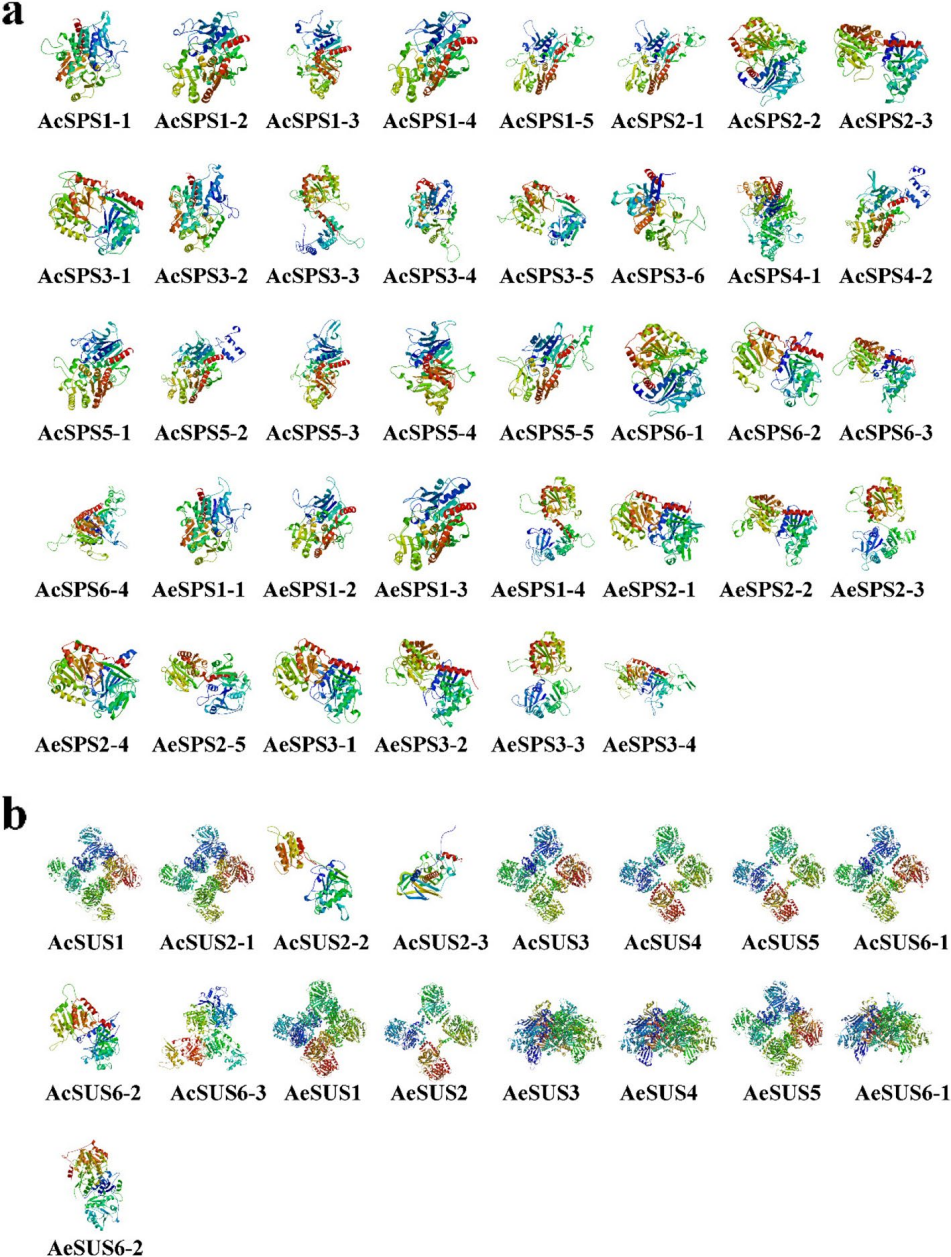
Tertiary structure analysis. The tertiary structure of AcSPS and AeSPS protein was shown in (**a**), AcSUS and AeSUS protein was shown in (**b**). There may be multiple tertiary structure models for the same protein sequence

The secondary structures of SUS and SPS proteins were all composed of four structural patterns: α-helix, random coil, extend strand and β-turn (Supplementary file 1), and showed α-helix > random coil > extend strand > β-turn on all SUS and SPS proteins. It was also found that β-turn of SPS was higher than that of SUS. The tertiary structure of the protein indicates that all SPSs contain multiple tertiary structures, AcSPS3 had the most, with 6 tertiary structures. In addition, based on the tertiary structure of SUS protein, we infer that there were two kinds of stable tertiary structure of SUS protein on kiwifruit, which were windmill type (e.g. AcSUS1, AeSUS1) and spider type (AeSUS3, AeSUS4 and AeSUS6-1) (Fig. 2), crucially, the latter only appeared on *A. eriantha*.

### Gene structure analysis

**Fig. 3:**
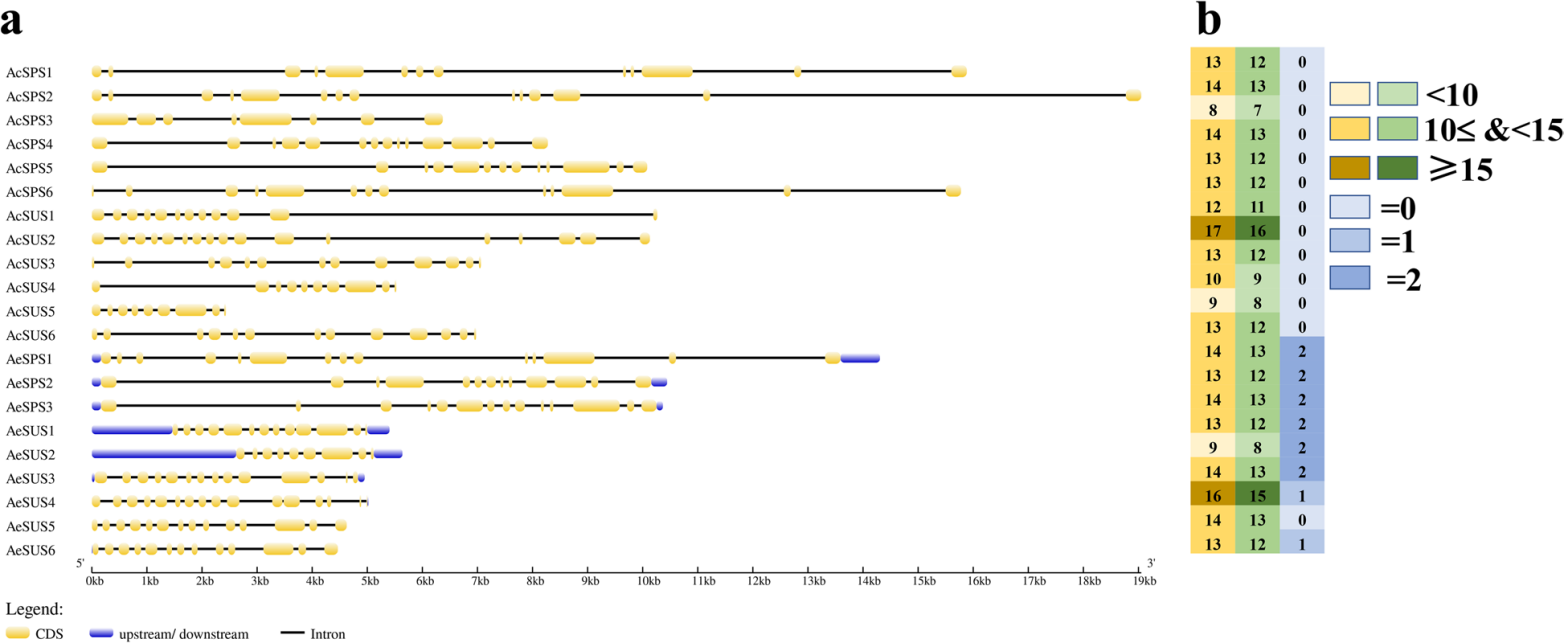
Intron-exon organization structure of *SUS* and *SPS* were analysis (**a**); the number of introns, CDS and upstream/downstream were shown in **b**, the first column was the number of CDS, and the second column was the number of introns, and the third column was the number of upstream/downstream

The gene structure was analyzed according to the CDS sequences (Supplementary file 2) and the corresponding genome sequences (Supplementary file 3) (Fig. 3a), *AcSPS and AcSUS* contain 8–14 CDS and 9–17 CDS, respectively. As for *AeSPS and AeSUS*, they contain 13–14 CDS and 9–16 CDS, respectively (Fig. 3b).

### Conserved motif analysis

**Fig. 4:**
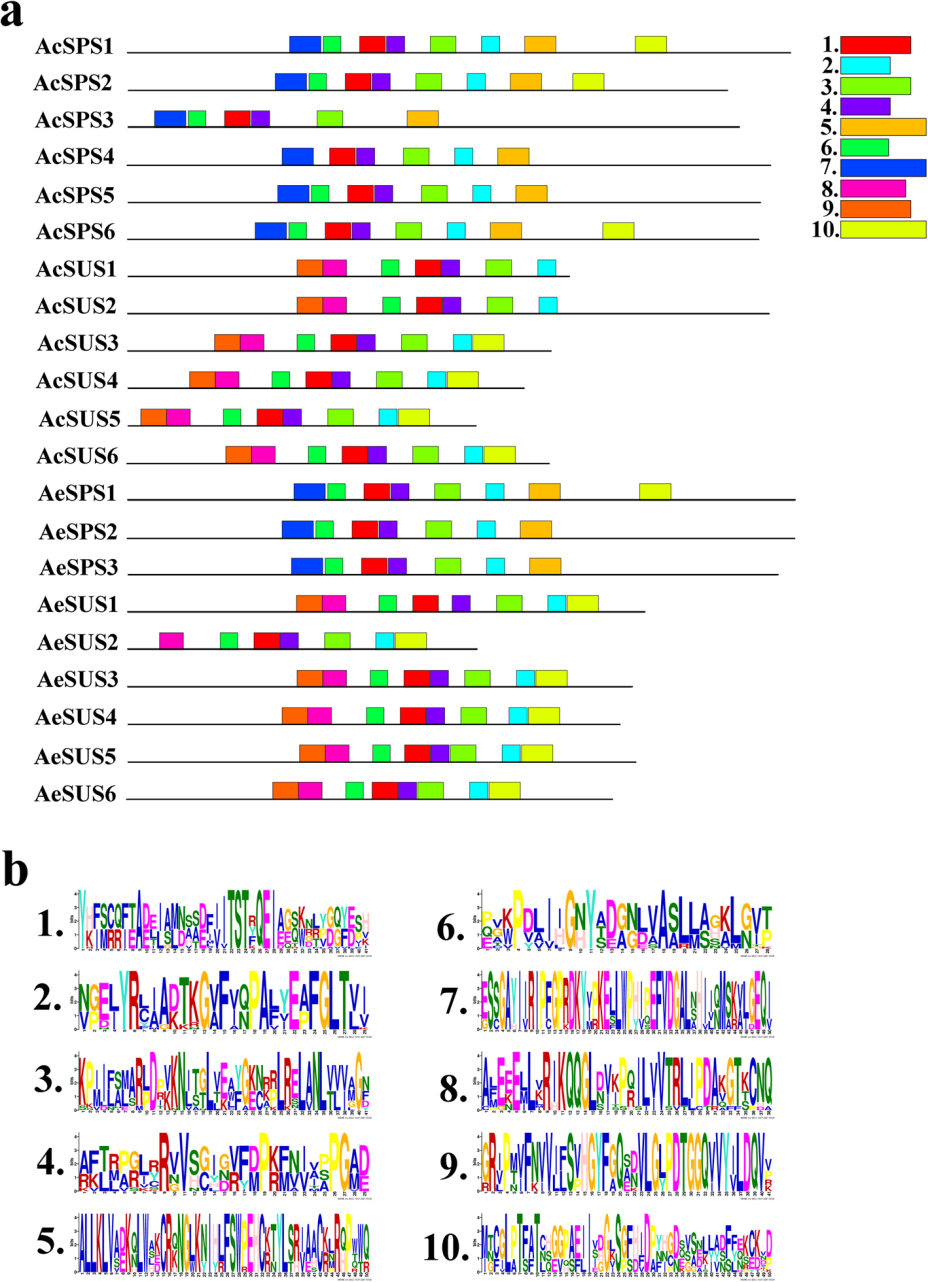
The distribution of conserved motif and amino acid sequence. Conserved motifs of SPS and SUS protein sequences were analyzed (**a**). Ten different motifs were recognized and indicated with deferent colors. The conservation of the sequences for each conserved domain was also presented (**b**)

MEME analyzed SPS and SUS protein sequences of *Actinidia*, all members of the SUS and SPS gene family members contain motif 1, motif 3 and motif 4, however, motif 5 and motif 7 only existed in SPS and motif 8 only in SUS (Fig. 4a). And identified 10 conserved motifs ranging in length from 29 to 50 amino acid residues (Fig. 4b).

### Phosphorylation site analysis

**Fig. 5:**
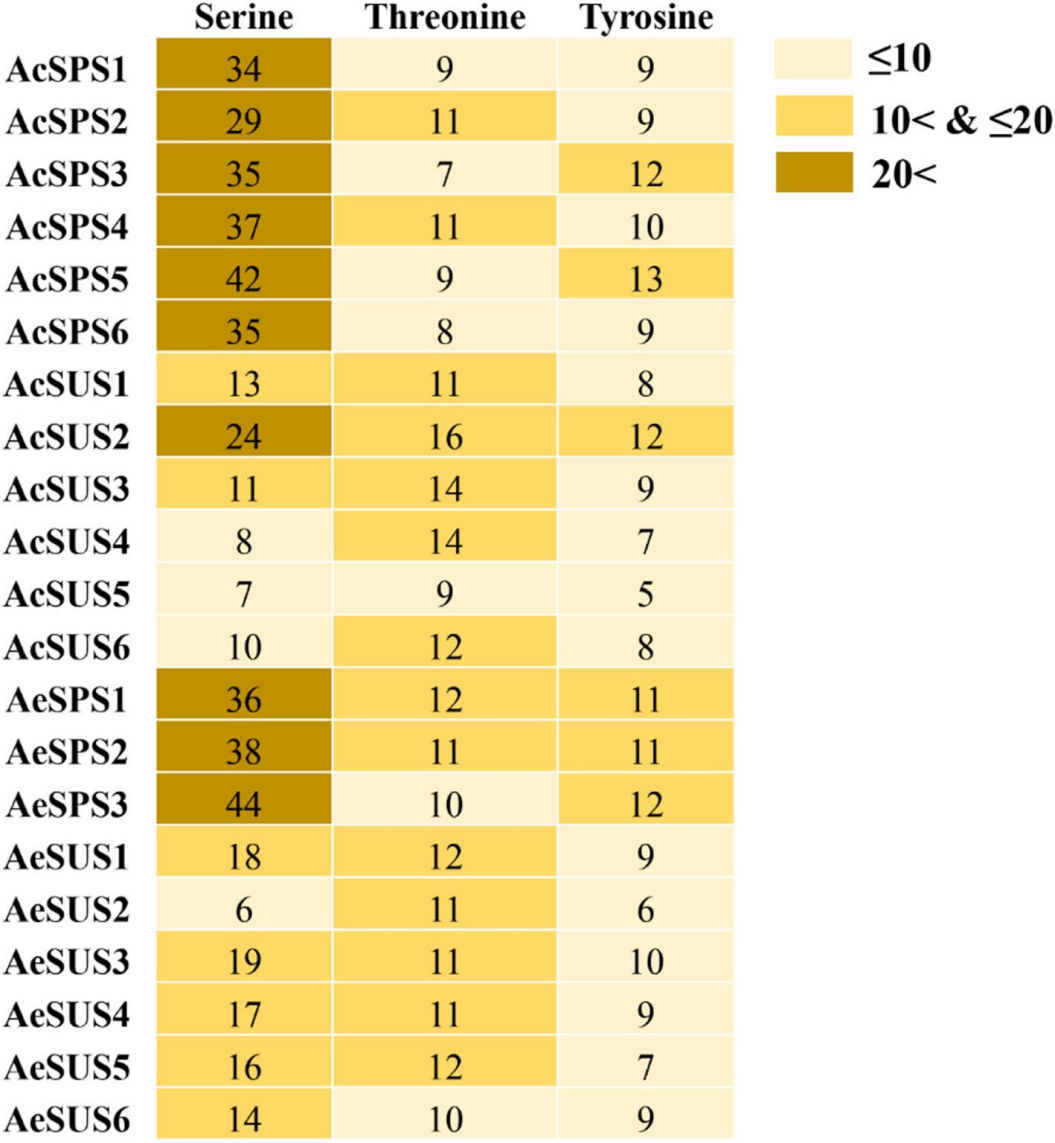
Phosphorylation site analysis of SPS and SUS proteins. The numbers in the first, second and third columns respectively represent the number of serine, threonine and tyrosine in the corresponding protein sequence

Phosphorylation sites of all SUS and SPS proteins were analyzed (Fig. 5, Supplementary file 4), the results showed that the main phosphorylation site of AcSPS and AeSPS were serine. The main phosphorylation site of AcSUS was threonine, except for AcSUS2. And the main phosphorylation site of AcSUS was serin, except for AeSUS2.

### Promoter cis-element analysis

**Fig. 6:**
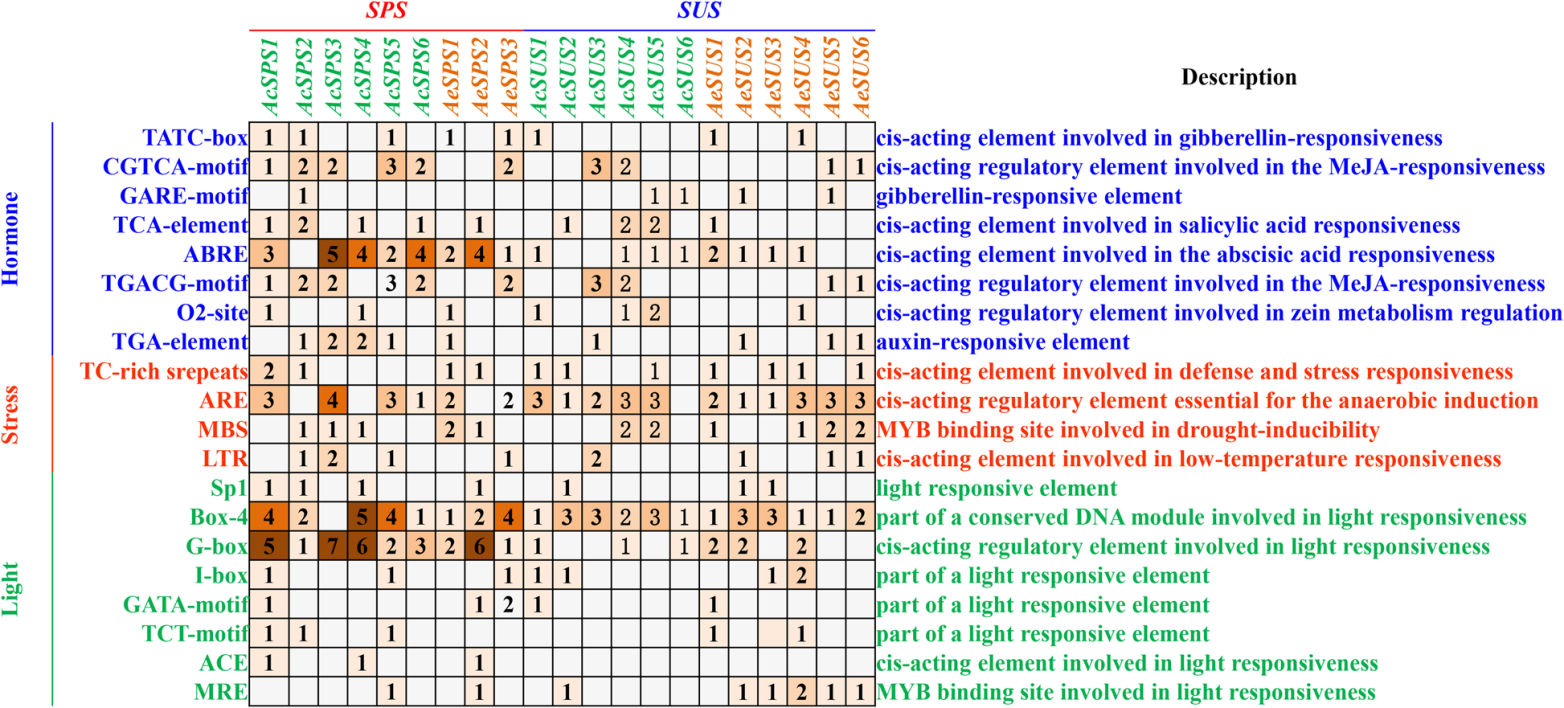
Promoter cis-element analysis of *SPS* and *SUS* gene family. The figure does not show all cis-acting elements, but only the cis-acting elements that are prevalent on *SPS* and *SUS*. The cis-acting elements related to hormones were shown in blue, those related to stress were shown in red, and those related to light were shown in green. The number in the box represents the number of corresponding cis-acting elements in the corresponding promoter sequence

Promoter cis-elements play an important regulatory role in plant growth and development. The analysis results of the promoter cis-element showed that there were many cis-acting elements related to hormones, stress and light in the promoter region (Fig. 6). Among them, we only found the ACE cis-acting element in the *AcSPS* and *AeSPS* genes, and GARE was only found in *AcSPS*. Interestingly, TCT-motif have not been found in *AeSPS* and *AcSUS*. The promoter sequences were shown in Supplementary file 5.

### Phylogenetic analysis

**Fig. 7:**
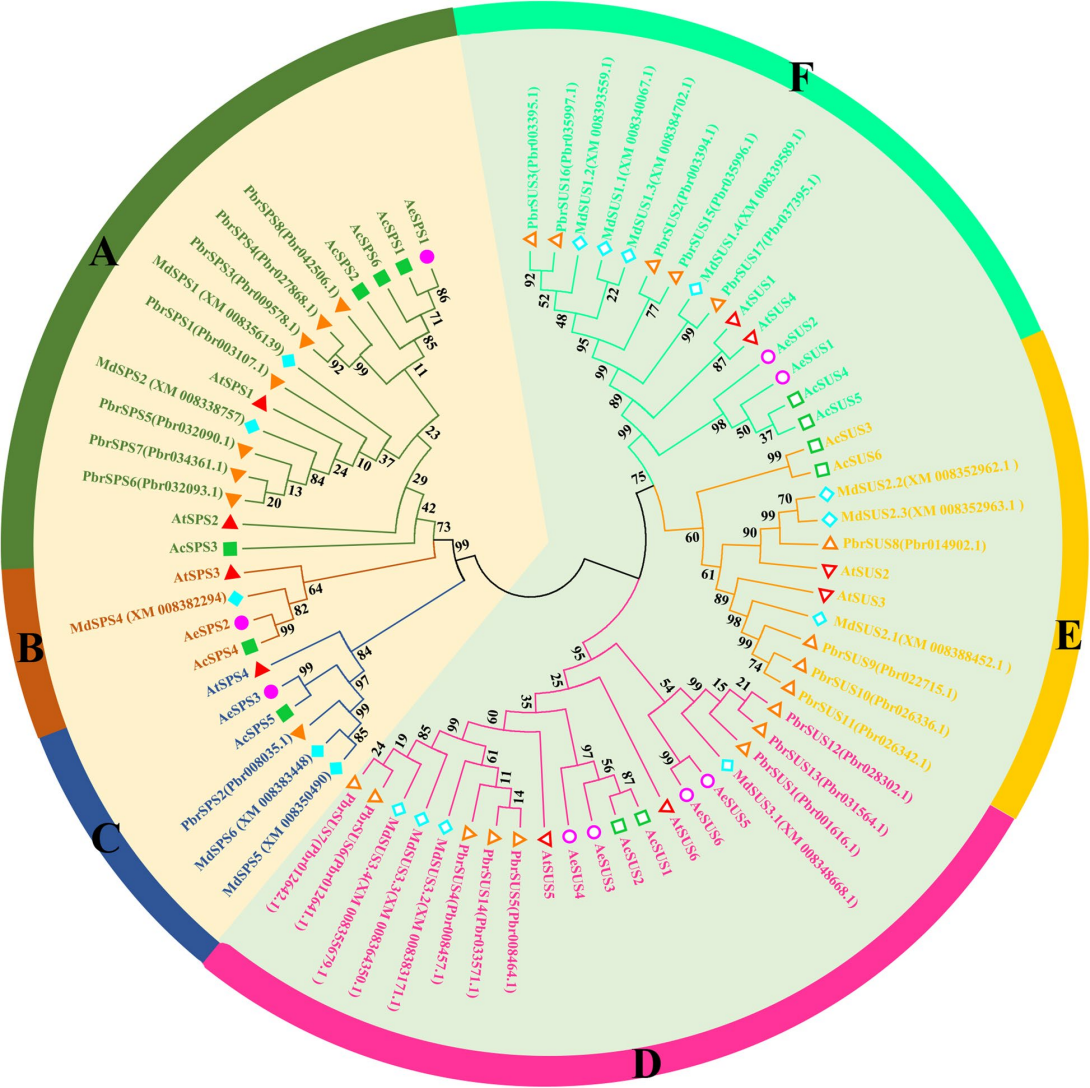
Phylogenetic analysis of SPS and SUS proteins from *Actinidia* and other plants. At: *Arabidopsis thaliana*; Ac: *Actinidia chinensis*; Ae: *Actinidia eriantha*; Md: *Malus domestica*; Pbr: *Pyrus bretschneideri*. The solid symbols represent the members of the SPS family, and the hollow symbols represent the members of the SUS family. The circle, regular triangle, inverted triangle, rhombus and square represent *A. eriantha*, *Arabidopsis thaliana*, *Pyrus bretschneideri*, *Malus domestica* and *A. chinensis*, respectively. A, B, C, D, E and F represent the six groups, respectively

To understand in-depth the evolutionary and phylogenetic relationships of SPS and SUS, a neighbor-joining phylogenetic tree was constructed using protein sequences from *Arabidopsis thaliana*, *A. chinensis*, *Malus domestica*, *Pyrus bretschneideri* and *A. eriantha* (Fig. 7). SUS and SPS were divided into two families, of which the SPS family includes three subfamilies (A, B and C) and SUS family also includes three subfamilies (D, E and F). Group A has more G-Box cis-acting elements, group B and C consist of cytoplasmic SPSs. As for group E, AcSUS3, AcSUS6 and SUS family members of other species grouped together. Genes with a similar number of introns clustered together.

### Multicollinearity analysis

**Fig. 8:**
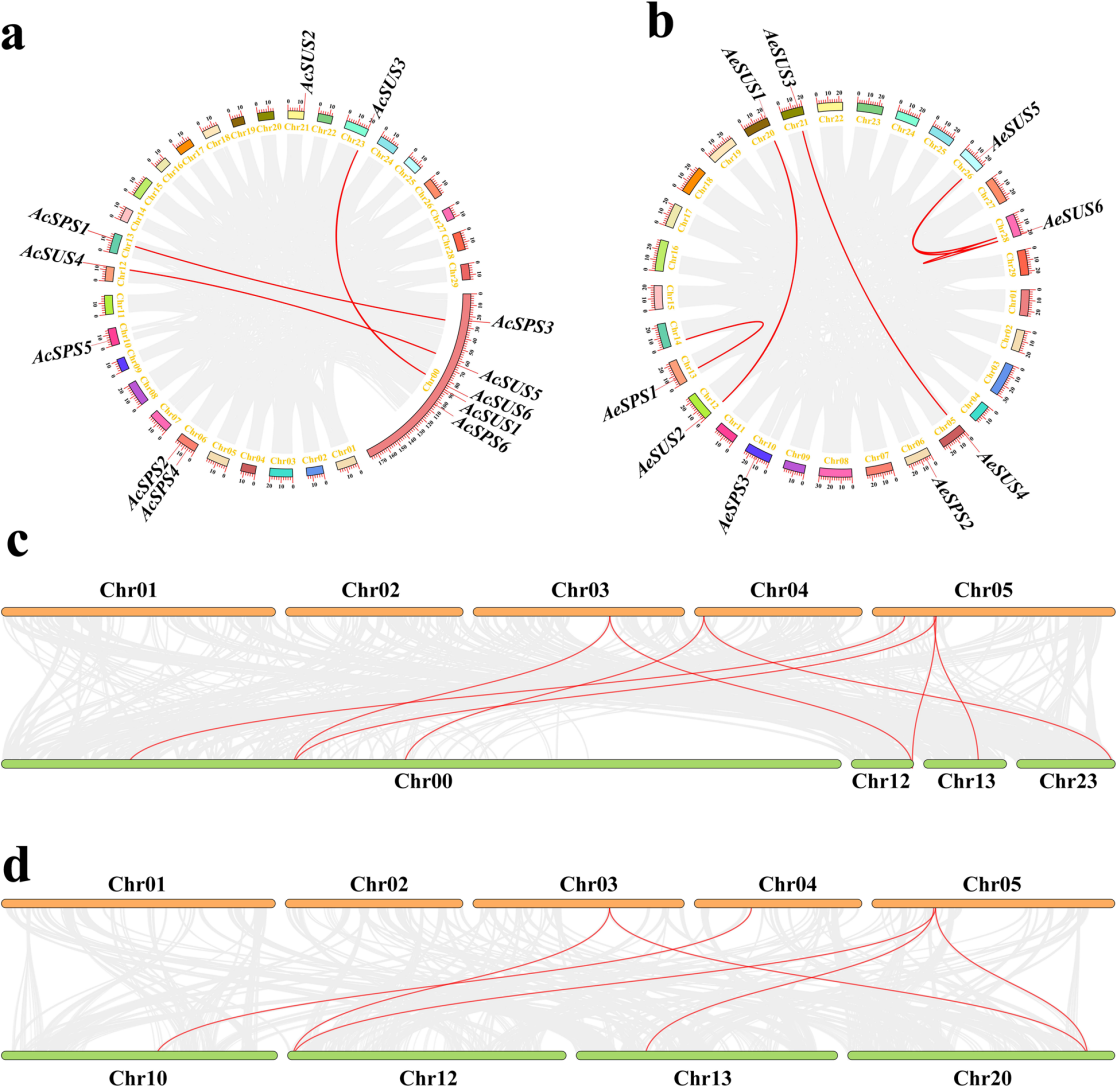
Collinearity analysis of the SUS and SPS gene families in *Actinidia chinensis* (**a**), *A. eriantha* (**b**), between *A. chinensis* and *Arabidopsis thaliana* (**c**), between *A. eriantha* and *A. thaliana* (**d**). The red lines connect two genes which exist multicollinearity. In a and b, the squares around the circles represent 29 chromosomes of *A. chinensis* and *A. eriantha*, respectively. Among them, *A. chinensis* has a sequence that has not been assembled into chromosomes. In c and d, the green boxes represent parts chromosomes of *A. chinensis* and *A. eriantha*, respectively. The orange boxes represent the chromosomes of *A. thaliana*

TBtools was used to analyze the multicollinearity of SPS and SUS gene family members in kiwifruit, and a circle graph was drawn (Fig. 8). Three multicollinearity gene pairs were found in both *A. chinensis* (Fig. 8a) and *A. eriantha* (Fig. 8b), they were *AcSPS1* and *AcSPS3*, *AcSUS3* and *AcSUS6*, *AcSUS4* and *AcSUS5*, *AeSUS1* and *AeSUS2*, *AeSUS3* and *AeSUS4*, *AeSUS5* and *AeSUS6*. In addition, we found a multicollinearity relationship between *AeSPS1*, *AeSUS5*, *AeSUS6* and other members of the non-*SUS* and *SPS* gene family. In the results of multicollinearity analysis with *A. thaliana*, 6 members of *AcSUS* and *AcSPS* gene families were found to have multicollinearity with *A. thaliana* (Fig. 8c), 4 members of *AeSUS* and *AeSPS* gene families were found to have multicollinearity with *A. thaliana* (Fig. 8d).

### Gene expression of fruits in different growth stages

**Fig. 9:**
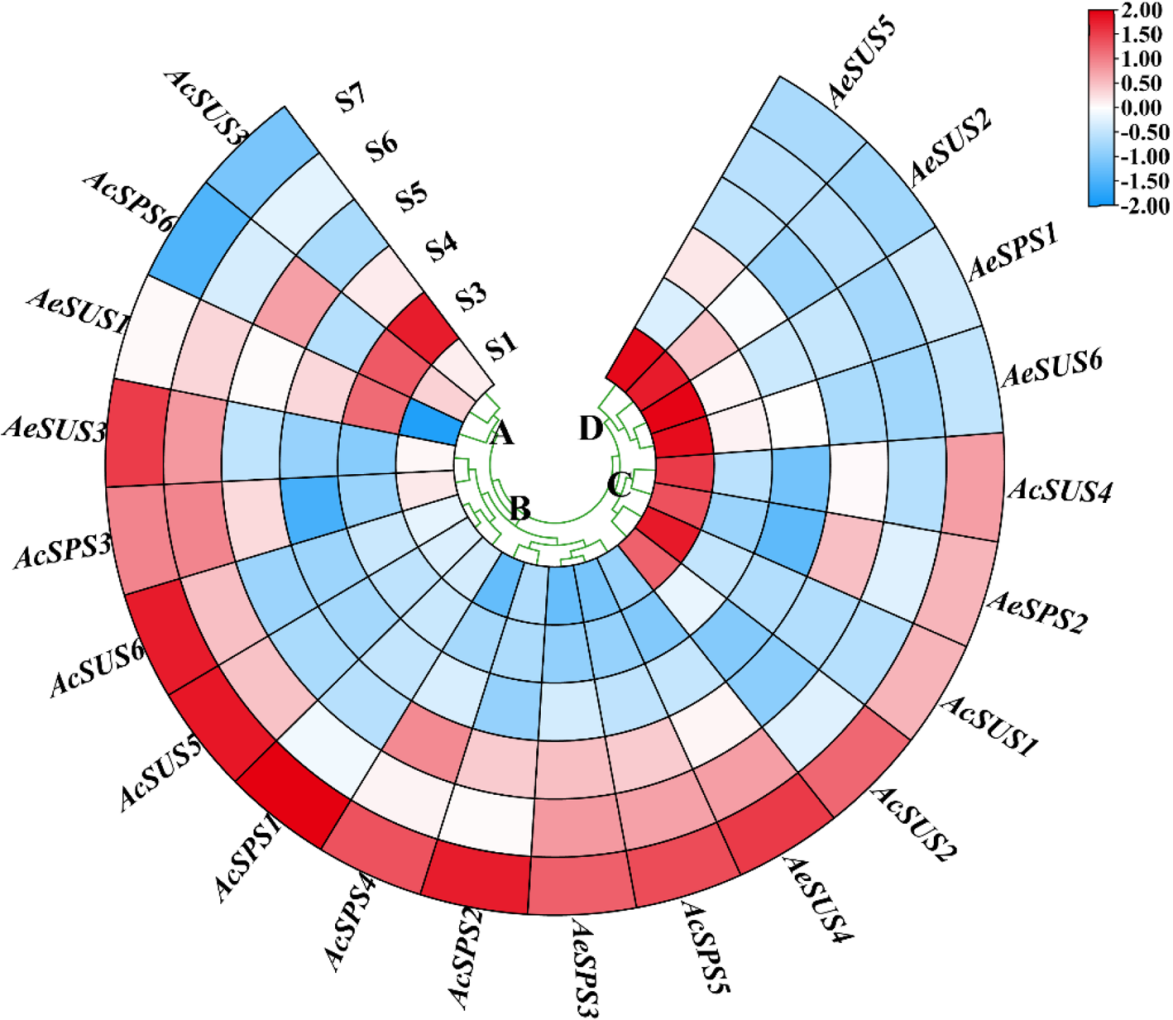
Relative expression levels of *SUS* and *SPS* genes at different stages of fruit development in *Actinidia eriantha* ‘Ganlv 2’. A, B, C and D represent the four groups, respectively. S1 to S7 represent different developmental stages of the fruit, representing 25 d, 50 d, 75 d, 125 d, 130 d, 145 d and 160 d after flowering, respectively. The higher the expression, the redder the color; the lower the expression level, the bluer the color

**Fig. 10:**
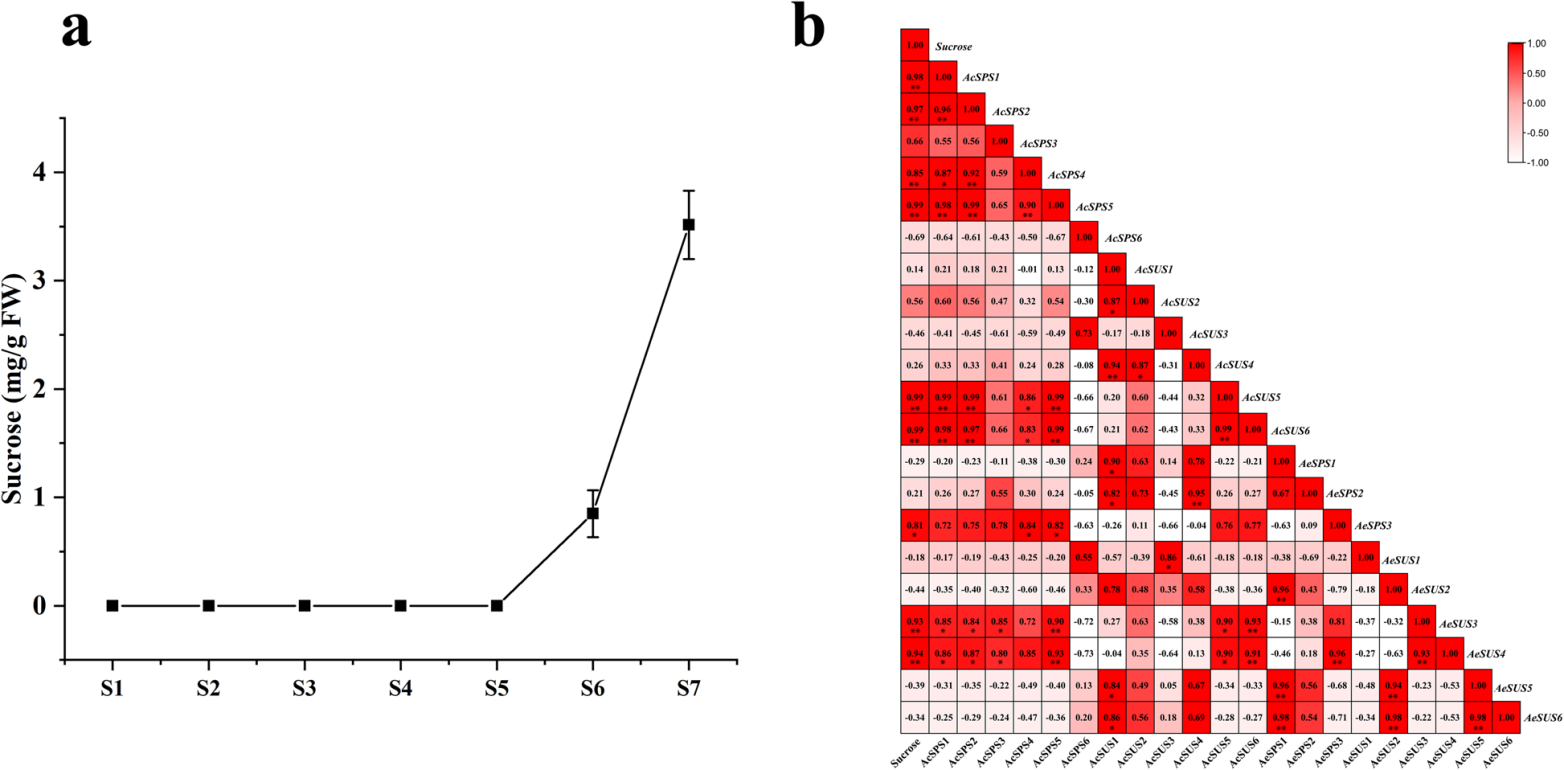
Sucrose content during fruit growth and development (**a**) and correlation analysis between genes and sucrose content (**b**). S1 to S7 represent different developmental stages of the fruit, representing 25 d, 50 d, 75 d, 125 d, 130 d, 145 d and 160 d after flowering, respectively. The number in the box was the Pearson coefficient, “*” means that the two was significant at the 0.05 level, and “**” means that the two was significant at the 0.01 level

We measured the relative expression of all genes, according to gene expression, genes can be divided into four groups: A, B, C and D (Fig. 9). Among them, the gene expression trend of group B was consistent with that of sucrose accumulation, it is very likely that the genes in group B play a key role in the metabolism of sucrose in the later stages of fruit development. While, the expression pattern of the genes in group D was completely opposite to that in group B, which may promote the decomposition of sucrose in the early stage, and decrease the expression in the later stage to promote the accumulation of sucrose. The peak of relative expression of group A at S3, and the relative expression level of group C was high in the early and late stages of fruit development. This suggests that different gene family members function at different stages of fruit development. In addition, the sucrose content of ‘Ganlv 2’ fruit in different development stages was measured, and it was found that sucrose content could not be detected before S6 stage, but increased sharply in the later stage of fruit development (Fig. 10a). The correlation analysis between genes and sucrose content showed that *AeSPS3*, *AeSUS3*, *AeSUS4*, *AcSPS1*, *AcSPS2*, *AcSPS4*, *AcSPS5*, *AcSUS5*, and *AcSUS6* were closely related to the regulation of sucrose (Fig. 10b).

## Discussion

Comparative genomics was used in this study to screen out 6 AcSPS, 6 AcSPS, 3 AeSPS, and 6 AeSUS genes from the Genomic Database of Kiwifruit (*A. chinensis* ‘Hongyang’ and *A. eriantha* ‘White’), which was similar to *Arabidopsis* [27] but significantly less than pear [20]. And the sucrose-synth and glycos-transf-1 domains expected of the SUS gene family, AcSUS2 also contains the specific structure domain CDC50, which is required for phospholipid translocation through the plasma membrane in saccharomyces cerevisiae [28], therefore, it is speculated that AcSUS2 genes may be involved in the phospholipid transport of cytoplasm or mitochondria, this is similar to the research reported on pears [20]. Phosphorylation and other post-translational modifications are responsible for protein function and protein-protein interaction [29, 30]. Furthermore, phosphorylation events involved various cellular processes affecting the subcellular localization and stability of target proteins [31, 32]. In the present study, more phosphorylation sites were predicted in SPS proteins than SUS proteins in *A. chinensis* and *A. eriantha*, indicating that SPSs are more influenced by post-translation modification events.

The tertiary structure is further coiled and folded on the basis of the secondary structure, and a better understanding of them could help us better understand gene function. Previous researches [19, 20] have missed the tertiary structure analysis of members of the SUS and SPS gene families. In our research, we noticed that AcSPS and AeSPS have more tertiary structures than AcSUS and AeSUS, which is one of the reasons why all SPS proteins are unstable. It’s worth noting that members of the SUS gene family had two common tertiary structure types (windmill and spider), whereas SPS had a wide range of tertiary structures. The spider-like secondary structure was discovered solely in *A. eriantha*. It needs to be seen whether these two tertiary structures cause the enzyme to serve a different role. At present, the function of sucrose synthase is known to break down sucrose, so that sucrose can be widely involved in plant metabolism and the composition of cell structure [33].

Previous studies have found that the gene intron/exon sequencing characteristics are crucial for understanding gene function and evolutionary relationships [34]. In this study, the genetic structure and conserved motifs of SPS and SUS were found to be very similar to those of that in plants [35], we found that the number of introns and CDS among members of SUS and SPS gene families was consistent, indicating that *SUS* and *SPS* genes were highly conserved during the evolutionary process. The results of conserved motif analysis also confirm this view, many SUS and SPS family members share the same conserved motifs. That’s one of the main reasons why some genes came together in the phylogenetic tree. According to the SUS subfamily classification on *Arabidopsis*, D, E and F correspond to SUS2 subfamily, SUSA subfamily and SUS1 subfamily of *Arabidopsis*, respectively, this was consistent with the previous studies [19, 20, 36]. The multicollinearity analysis of *SPS* and *SUS* shows that *SPS* and *SUS* are generated by replication among their own family members. However, we found three abnormal cases, *AeSPS1*, *AeSUS5* and *AeSUS6*. Among them, *AeSPS1* was copied by a gene on chromosome 14, but this gene was not a member of the *AeSPS* gene family. Through conserved domain analysis, we found that it lacks a characteristic conserved domain. This is most likely due to the deletion of fragments that occur during gene replication. The same is true for *AeSUS5* and *AeSUS6*.

Gene promoter analysis showed that all members of the SPS and SUS family have multiple light-response elements, hormone regulatory elements and stress regulatory elements. These findings suggested that several homologous genes were formed gradually over the development of plants, avoiding the scenario in which plant growth was slowed or even stopped owing to the loss of function of a single gene due to mutation. SPS and SUS genes, on the other hand, are involved in stress management as well as growth and development processes. The cis-elements of genes that were highly related to sucrose were analyzed, and it was discovered that the cis-elements of *AeSPS* and *AcSPS* promoters were primarily light-responsive related elements, with Box-4 elements accounting for the majority; *AeSUS* and *AcSUS* promoters were primarily stress-related elements, with ARE elements accounting for the majority; and *AeSUS* and *AcSUS* promoters were primarily stress-related elements, with SPS gene members involved in sucrose regulation were also predicted to play a role in light response, whereas SUS gene members play a role in stress [20].

Previous researches [26, 37] indicated that kiwifruit was a high-sucrose accumulation fruit, which is consistent with our research results, and we also found that sucrose was accumulated in the late stage. As is known to all, the concentration of sucrose in fruits is regulated by a variety of sucrose metabolism enzymes, including NIV, SUS, and SPS [6, 38]. The accumulating mechanism of sucrose can be better understood by determining the expression levels of enzyme genes at different stages of fruit growth. The findings of gene expression profiling revealed that most SPS genes showed a tendency of increased expression with fruit ripening, while *AcSPS1*, *AcSPS2*, *AcSPS4*, *AcSPS5* and *AeSPS3* had the highest correlation coefficients. It was shown that these genes may have a role in the high-sucrose accumulation type of kiwifruit, which is similar to the findings of apple and melon study [39, 40]. One of the most interesting findings was that most SPS genes, including *AcSPS5*, were significantly expressed at harvest time, suggesting that *SPS* genes were involved in sucrose accumulation in the fruit’s late stages. Although *AcSPS6*, *AeSPS1*, and *AeSPS2* were expressed in the early stages, invertase activity was strong in the early stages, resulting in invertase-mediated hydrolysis of the synthesized sucrose [3]. So, it didn’t promote the accumulation of sucrose in the fruit. According to studies on *Arabidopsis*, *AtSPS4* mutation reduced the activity of sucrose phosphate synthase by only 13%, indicating that *AtSPS4* had minimal effect on sucrose accumulation in *Arabidopsis* [41]. However, *AtSPS4* and *AcSPS5* were in the same subfamily according to the results of phylogenetic tree, and the expression of *AcSPS5* was the highest and consistent with the sucrose trend. This is most likely due to species differences, or it might be that *Arabidopsis* does not have a high sucrose content and that the reduced activity of SPS has little effect on its sucrose. Some gene expression patterns, such as *AcSUS6*, *AeSUS3*, and *AeSUS4*, ran counter to the sucrose content trend. This suggests that these were primarily responsible for the overall activity of SUS [39].

## Conclusions

In this study, we identified 6 *SPS* genes and 6 *SUS* genes from *A. chinensis* (cultivar: ‘Hongyang’), and 3 *SPS* genes and 6 *SUS* genes from *A. eriantha* (cultivar: ‘White’). We carried out bioinformatics analysis of these genes, and detected the expression levels of these genes during the growth and development of kiwifruit. The results showed that *AcSPS1*, *AcSPS2*, *AcSPS4*, *AcSPS5*, *AcSUS5*, *AcSUS6*, *AeSPS3*, *AeSUS3* and *AeSUS4* were the important genes in regulating the synthesis and accumulation of sucrose for *Actinidia*. Our work would provide a basis for further study on the molecular mechanism of sucrose accumulation in *Actinidia*.

## Materials and methods

### Materials

*A. eriantha* ‘Ganlv 2’ has been formally identified by Professor Xu of Jiangxi Agricultural University (the new plant cultivar number is 20,191,004,327) [42] and used as the experimental material, were grown in the kiwifruit germplasm nursery of Fengxin County, Jiangxi Province, China. After getting permission from the orchard owner, we selected six plants with the same growth status, and each two plants were used as a biological repeat. Study protocol comply with relevant institutional, national, and international guidelines and legislation. Flesh from seven different periods were used for genes expression, they were 25 days after full bloom (DAF) (S1), 50 DAF (S2), 75 DAF (S3), 100 DAF (S4), 125 DAF (S5), 135 DAF (S6) and 145 DAF (S7), respectively, and four fruit were collected from the four directions of the vines each time. At 145 DAF, the fruit reached the commercial harvest standard (soluble solid content = 6.5% [43]. The harvested fruit were put in liquid nitrogen containers and instantly carried back to the lab for measured the expression of genes.

### Identification of *AcSUS* and *AcSPS*

The AtSUS and AtSPS protein sequences (Supplementary file 6) were obtained from *Arabidopsis thaliana* germplasm information database (http://www.arabidopsis.org), and the candidate sequence of AcSPS, AcSUS, AeSPS and AeSUS were obtained by Blastp analysis in kiwifruit database (http://kiwifruitgenome.org/). The candidate sequences were detected by Pfam (http://pfam.xfam.org) and Uniprot (http://www.uniprot.org/) for whether they contained conserved sucrose synth domain (PF00862), glycose-transf-1 domain (PF00534) and S6PP domain (PF05116). The sequences containing the sucrose synth (PF00862), glycose-transf-1 (PF00534) were selected as candidate sequences of the *SUS* gene family members (Supplementary file 7), the sequences containing the sucrose synth (PF00862), Glycose-transf-1 (PF00534) and S6PP (PF05116) were selected as candidate sequences of the *SPS* gene family members (Supplementary file 8).

### Bioinformatics analysis

ProtParam (https://web.expasy.org/protparam/) predicted Physicochemical properties. NPSA (https://npsa-prabi.ibcp.fr/cgi-bin/npsa_automat.pl?page=npsa_sopma.html) and SWISS-MODEL (https://swissmodel.expasy.org/) predicted the secondary structure and tertiary structure, respectively. Netphos 2.0 Server (http://www.cbs.dtu.dk/services/NetPhos/) was used to analyze phosphorylation sites, with a prediction threshold of 0.5. Gene Structure Display Server (http://gsds.gao-lab.org/index.php) [44] was used to analyze gene structure after extracting genomic and CDS sequences with TBtools [45]. MEME (https://meme-suite.org/meme/tools/meme) [46] was used to examine the conserved motifs, with the number of motifs set to 10. MEGA software (version 5.05) was used to create an unrooted phylogenetic tree using the neighbor-joining method, with 1000 bootstrap repetitions. TBtools [45] performed multicollinearity analysis among gene family members. Also, TBtools was used to extract the 2000 bp sequence upstream of the gene coding base (ATG), then using PlantCARE (http://bioinformatics.psb.ugent.be/webtools/plantcare/html/) to analyze the promoter cis-regulatory element.

### Determination of sucrose content

Sucrose was determined by high performance liquid chromatography (HPLC) [47]. Mobile phase: A (purified water after degassing) and B (0.8% acetonitrile solution), the flow rate was 0.2 mL/min. The column temperature was 30 ℃, and the injection volume was 2 µL. The sucrose content was calculated according to the peak area of the sample and the external standard curve, and the standard sample was purchased from Sigma.

### RNA extraction and qRT‑PCR

RNA extraction and qRT‑PCR were performed with our previous methods [48]. Using Primer 5 to design qPCR primer (Table 3) and the *β-actin* in the kiwifruit was considered as the control gene for normalization [49]. Finally, the relative expressions were calculated using the 2^−ΔΔCt^ method [50].

**Table 3:** Designed Primers of quantitative real-time PCR

Gene Name	Forward primer sequence	Reverse primer sequence
*AcSPS1*	CTATCAATGACAAGAAGGGCGAAAA	GCAACGGTGAGCCTGAATCCT
*AcSPS2*	ATGCTTTTCACTGGTCACTCACT	CATCATACAGACGCCATTGCTC
*AcSPS3*	TTCTGAAGTTGGTCCTTTTGGG	TCGTCTTCAGCAACTTGTCCTC
*AcSPS4*	ACGAGCACCAAGCAGGAGATT	CCACAACATTGCTGAAGTCCATA
*AcSPS5*	GGAGAAGGAAAAGGGTGATGC	CCTGACCTCCAGTGTCTGAATC
*AcSPS6*	CAATGGCGTCTGTATGATGGT	GGGTCTGGAGAAGTAGGCTGAT
*AcSUS1*	AACTTGGGATTACTCTGGGAACT	ATTTCTTGGTATGTGCTGGTGAT
*AcSUS2*	GGTGGCTTACAGAAGGCTCAG	CCACTGCCTAAACCTTTGCTC
*AcSUS3*	TCAAAGAATACAACTTGGATGGC	CAAGTGGCAAATGTTGGAAGC
*AcSUS4*	TTGGGCTATCCTGACACTGGT	CAATGAGAATGCGTGGAATGA
*AcSUS5*	ACACTGGTGGTCAGGTGGTTT	GCGAATGTTCTGCTCCGTAAA
*AcSUS6*	ATCAAAGCCCATTATCTTCTCCA	TGACTTCTTCACATCATTGTAACCC
*AeSPS1*	TATCGCTTGATGCCTCTGAAA	TCCTGGCTCGTAGTTTCCGT
*AeSPS2*	GGGCTTTGAATAATGGTCTGC	TCAAGTATGTGCGGCAGTGTT
*AeSPS3*	GGGCTTTGAATAATGGTCTGC	TCAAGTATGTGCGGCAGTGTT
*AeSUS1*	ACCTTGTTGCCTCATTGTTAGC	CCTGGAAAGTGCTGGTGATTAT
*AeSUS2*	AAGAGCAAGCCGAGATGAAGA	CCGTCAACCCAAAAGCCTCA
*AeSUS3*	TACTGCGGAAAGAGTGAGGGA	ACATAGACCACCTGCCCACC
*AeSUS4*	GAGTTCTTCCGCAATGGGTT	AGTTCCCAGAGTAATCCCAAGTT
*AeSUS5*	TGAGAAAGGGTGGGGAGATAA	AAGTAACCGTGGACCGAGAAG
*AeSUS6*	ATGTTGCTTTGGCAGTGAGG	GTCGCAGATTGTTTCTTTGAGC

### Date analysis

The experimental data was statistically analyzed by Origin 2018. R × 64 3.5.0 was used for make gene expression heat map. Significant analysis and difference analysis were analyzed by IBM SPSS Statistics 22.0.

## Supplementary Information

**Additional file 1.**

**Additional file 2.**

**Additional file 3.**

**Additional file 4.**

**Additional file 5.**

**Additional file 6.**

**Additional file 7.**

**Additional file 8.**

## Data Availability

The datasets supporting the conclusions of this article are included in the article. The public databases used in this research are all open, and links to all databases as follows. Kiwifruit Genome Database (*A. chinensis* ‘Hongyang’): http://kiwifruitgenome.org/organism/2; *Arabidopsis thaliana* germplasm information database: http://www.arabidopsis.org; Pfam: http://pfam.xfam.org; Uniprot: http://www.uniprot.org/; ProtParam: https://web.expasy.org/protparam/); NPSA: https://npsa-prabi.ibcp.fr/cgi-bin/npsa_automat.pl?page=npsa_sopma.html; SWISS-MODEL: https://swissmodel.expasy.org/; PlantCARE: http://bioinformatics.psb.ugent.be/webtools/plantcare/html/; Netphos 2.0 Server: http://www.cbs.dtu.dk/services/NetPhos/.

## Abbreviations

SUS: Sucrose synthase
SPS: Sucrose phosphate synthase
DAF: Days after full bloom
Chr: Chromosome
CDS: Coding sequence
