# Supplementary material for: Genome-wide identification and expression profiling analysis of sucrose synthase (SUS) and sucrose phosphate synthase (SPS) genes family in Actinidia chinensis and A. eriantha
Source: BMC Plant Biol. 2022 Apr 26;22:215. doi: 10.1186/s12870-022-03603-y (PMC9040251; doi:10.1186/s12870-022-03603-y)

Supplementary file 1 Secondary structure analysis of members of *SPS* and *SUS* gene families in *Actinidia*.


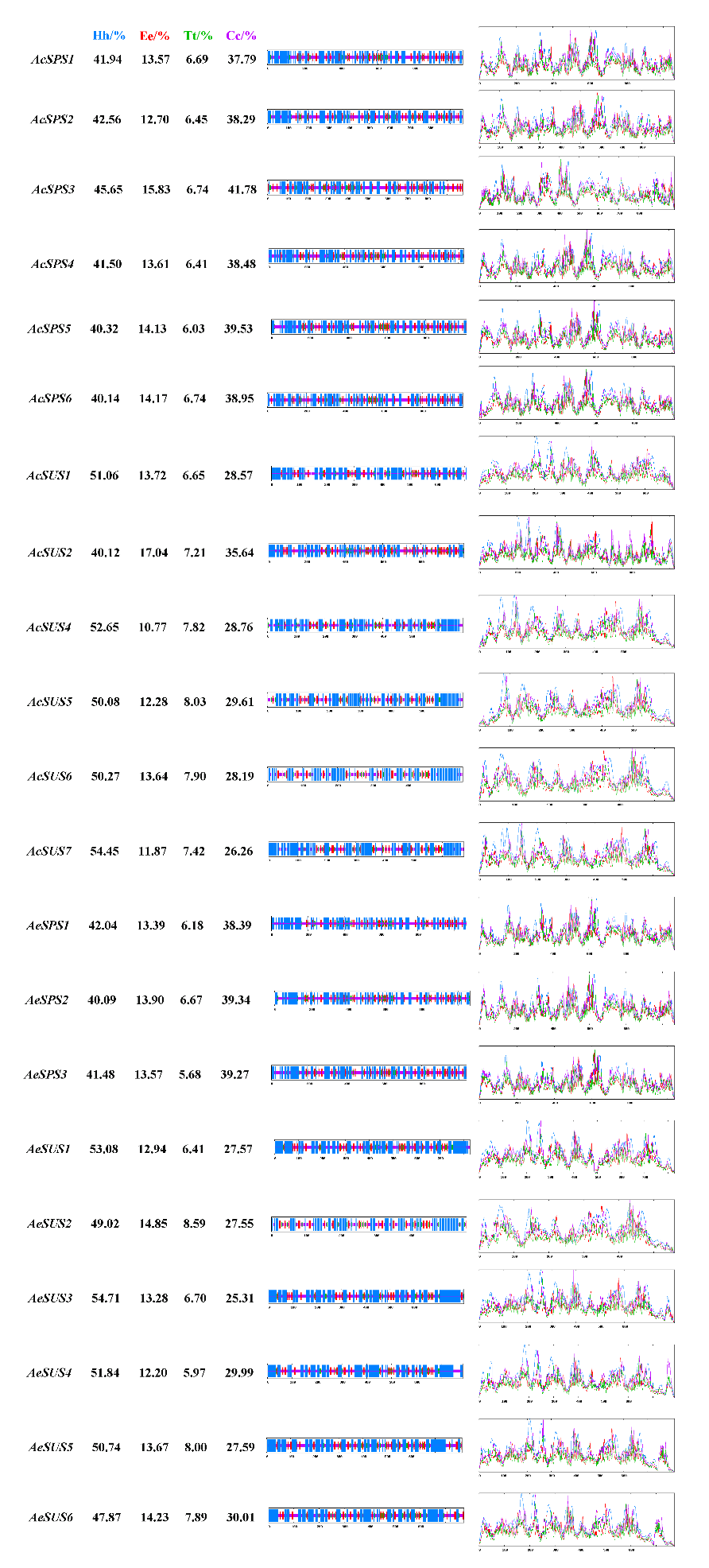

Supplement: Supplementary file 1 — Additional file 1. [file 12870_2022_3603_MOESM1_ESM.docx]
