# Supplementary material for: Genome-wide identification and expression profiling analysis of sucrose synthase (SUS) and sucrose phosphate synthase (SPS) genes family in Actinidia chinensis and A. eriantha
Source: BMC Plant Biol. 2022 Apr 26;22:215. doi: 10.1186/s12870-022-03603-y (PMC9040251; doi:10.1186/s12870-022-03603-y)
Supplement: Supplementary file 2 — Additional file 2. [file 12870_2022_3603_MOESM2_ESM.docx]

Supplementary file 2 CDS sequences of members of the *SUS* and *SPS* gene families in *Actinidia*.

>AcSPS1

ATGGCGGGAAACGACTGGATAAACAGTTACCTGGAGGCGATACTGGATGTGGGGCCAGGGATCGACGACGCGAAATCGTCGTTGCTGCTTAGAGAGAGAGGCAGGTTCAGTCCCACTCGCTACTTCGTCGAGCAGGTCATCGGTTTCGATGAGACCGATCTCTACCGCTCCTGGGCGGCGGCGACGAGAAGTCCGCAGGAGCGGAATACGAGACTCGAGAACATGTGCTGGCGGATTTGGAATTTGGCTCGCCAGAAAAAGCAGCTTGAGGGAGAGGAAGCTCAAAGGATGGCTAAACGTCGTCTTGAACGTGAAAGAGGACGCAGAGAAGCAACTGCTGATATGTCTGAAGACTTATCTGAGGGGGAAAAAGGAGATACAGTCAGCGATCTGTCGGCTCATGGTGAAAGCAACAGGGGCCGATTACCTAGAATTAGCTCCGTTGAGACAATGGAGGCATGGGTTAGTCAACATAAGGGGAAAAAGCTGTACATTGTCTTAATAAGGCATGAATTTGAATTCCATTCATTTGGCTTGCAAATCTTCCTTCATGGTCTAATACGGGGTGAAAATATGGAGCTTGGTCGTGATTCTGATACTGGTGGCCAGGTTAAGTATGTTGTGGAACTTGCAAGGGCTTTGGGTTCAATGCCAGGAGTGTATCGGGTTGATTTGCTCACTAGACAAGTATCATCACCAGAAGTAGACTGGAGTTATGGTGAACCCACTGAAATGTTGCCTCCAAGAAATTCTGATGGTTTGATGGATGAGATGGGGGAGAGTAGTGGCGCTTATATTATTCGTATTCCATTTGGCCCAAGAGATAAATATGTACCGAAAGAACTTCTGTGGCCGCACATCCCTGAATTTGTTGATGGTGCTCTTAACCACATCATACAGATGTCCAAAGTACTTGGTGAGCAAATTGGCAGTGGGCATCCTGTGTGGCCTGTTGCTATCCATGGGCATTATGCAGATGCAGGTGATGCCGCTGCTCTTCTATCAGGTGCTTTAAATGTACCCATGCTTTTCACTGGTCACTCACTTGGTAGGGATAAGCTGGAACAGCTTTTGAGACAAAGTCGATTATCAAAGGATGAAATAAATAAGACATACAAAATAATGCGTCGTATAGAAGCTGAGGAGTTATCGCTTGATGCCTCTGAAATAGTGATAACTAGCACTAGACAGGAGATAGAACAGCAATGGCGTTTGTATGATGGTTTTGATCCGGTGATAGAACGGAAACTACGAGCCAGGATCAGGCGTAATGTGAGCTGTTATGGCAGGTTCATGCCTCGCATGGTTGTAATGCCCCCTGGGATGGAATTTCATCACATTGTTCCACATGAAGGTGATATGGACGGTGAAACTGAAGGAAATGAAGACCAGCCTACTTCTCCAGACCCACCCATTTGGCCTGAGATAGTGCGCTTCTTTACTAATCCACGCAAGCCGATGATACTTGCCCTAGCTAGGCCAGATCCCAAAAAGAATCTCGCAACTTTGGTTGAAGCATTTGGGGAATGTCGTCCATTAAGAGAGCTGGCTAATCTGACTTTAATAATGGGTAACCGAGGTGATGTTGATGAAATGTCAAGCACTAATTCTTCTGTTCTTCTCTCAATACTTAAGCTTATTGATAAGTATGATCTCTATGGTCAAGTGGCATACCCCAAACACCACAAGCAGTCTGATGTTCCTGATATCTACCGTCTGGCAGCTAAGACAAAGGGTGTTTTCATTAATCCAGCTGTCATTGAGCCCTTTGGGCTTACTCTGATCGAGGCAGCAGCTTATGGGTTACCAATTGTCGCCACAAAAAATGGAGGTCCTGTTGACATACATCGGGCTCTTGACAATGGTCTCCTTGTGGACCCCCATGATCAGAAGTCTATTGCTGATGCTCTTTTAAAACTGGTTGCGGATAAGCAACTTTGGTCCAAGTGCCGCCAGAATGGGTTGAAAAATATTTACCTTTTCTCATGGCCAGAACATTGTAAAACTTACCTATCTCGAATAGCAGCTTGCAAACTGAGGCAACCATGGTGGCAAAGAAGTGACGATGGGGATGAAAATTCTGAGTCGGATTCACCAAGTGACTCCTTGAGAGATATATCCTTGAACTTAAAGTTTTCACTGGATGGAGAAAAGAATGAAGGCAGCGGAAATGCCGATGGTTCTTTAGAATTTGAAGATCGCAAGATTAAGTTGGAGAATGCTGTTTTGACATGGTCAAAGGGTTTCCAGAAGGGCACACAAAAGGCTGGGGTTACAGAGAAAGCAGATACGAACATCACTGCTGGAAAGTTCCCAGTGTTGAGGAGGAGGAAGAATATTATTGTTATTGCCGTGGATTTTGGTGCTATATCAGATTATTCCGATAGTATTAGAAAGATATTTGACGCTGTGGAGAAGGAAAGGACTGAAGGCTCTATAGGATTTATATTAGCAACATCCTTTACTTTGTCCGAAGTCCATTCTTTTCTCATCTCTGGCGGACTGAGCCCTTCTGATTTTGATGCGTTTATCTGCAATAGTGGTAGTGATCTCTACTATTCATCTCTTAATTCAGAGGATAATCCCTTCGTTGTTGACTTATATTACCACTCACATATTGAATACCGCTGGGGTGGAGAAGGGTTAAGGAAGACTTTGATTCGTTGGATGGGTTCTATCAATGACAAGAAGGGCGAAAATGAAGAGCAGATTGTTACCGAAGATGAAAAGATTTCAACCAATTATTGTTATGCTTTCAAAGTGCGAAATGCAGGGAAGGTTCCTCCTGTCAAGGAAATCAGAAAATTAATGAGGATTCAGGCTCACCGTTGCCATGTTATTTATTGCCAGAATGGGAATAAGATAAATGTAATTCCAGTATTGGCGTCTCGTTCCCAAGCCCTGAGGTATCTCTATCTCCGGTGGGGCGTGGACTTGTCAAAAATGGTGGTTTTTGTCGGAGAAAGCGGGGACACTGACTACGAGGGCTTGCTTGGTGGCATACACAAGTCTGTAATATTGAAGGGAGTTTGTAGCGGTCCGACCAATCAACTCCATGCCAACAGAACCTACCCTCTTTCTGATGTCCTGCCGATTGACAGCCCTAACATTGTCCAGGCAGCTGAGGAATGCAGCAGTGCCGATCTCCGGACCTCGTTGTTGAAGCTAGGGTTCATCAAGGGCTAG

>AcSPS2

ATGGCGGGAAACGACTGGATAAACAGTTACCTGGAGGCGATACTGGATGTGGGGCCAGGGATCGACGACGCGAAATCGTCTTTGCTGCTTAGAGAGCGAGGCAGGTTCAGTCCCACTCGCTACTTCGTCGAGCAGGTCATCGGCTTCGGTGAGACCGATCTCTATCGCTCCTGGGTTAAGGCGGCTGCGACGAGAAGTCCTCAGGAGCGGAATACGAGACTCGAGAACATGAGCTGGCGGATTTGGAATTTGCTTGAGGGAGAGGAAGCTCAAAGGATGGCTACACGTCGTCTTGAACATGAAAGAGGCCTCAGAGAAGCAACTGCTGATATGTCTGATGACTTGTCCGATGGGGACAAAGGAGATACGGACAGCGATTTGTCTGCTCATGGTGAAAGCAACAGGGGCCGATTACCTAGAATTAGCTCCGTTAAGACAATGGAGGCATGGGTTAGTCAACAGAAGGGGAAAAAGCTCCTTCATGGTCTAATACGGGGTGAAAATATGGAGCTTGGTCGTGATTCTGATACTGGTGGCCAGGTTAAGTATGTTGTGGAACTTGCAAGGGCTTTGGGTTCAATGCCAGGAGTGTATCGCGTTGATTTACTCACTAGACAAGTATCATCACCAGAAGTAGACTGGAGTTATGGTGAACCCACTGAAATGTTGACTCCAAGAAATTCTGATGTTTTGATGGATGAGATAGGGGAGAGTAGCGGTGCTCATATTATTCGTATTCCATTTGGCCCAAGAGATAAATATGTACCGAAAGAACTTCTGTGGCCACACATTCCTGAATTTGTTGATGGTGCTCTTAACCACATCATACAGATGTCCAAAGTACTTGATGAGCAAATTGGCAGTGGGCATCCTGTGTGGCCTGTTGCTATCCATGGGCATTATGCAGATGCAGGTGATGCCGCTGCTCTTCTATCAGGTGTTCTAAATGTACCCATGCTTTTCACTGGTCACTCACTTGGTAGGGATAAGCTGGAACAGCTTTTGAGACAAAGTCGACTATCAAAGGATGAAATAAATAAGACGTACAAAATAATGCGTCGTATAGAGGCCGAGGAATTATCTCTTCATGCCTCCGAAATAGTGATAACTAGCACTAGACAGGAGATAGAAGAGCAATGGCGTCTGTATGATGGTTTTGATCCTGTACTTGAACGGAAACTACGAGCCAGGATAAGGCGTAATGTGAGCTGTTATGGCAGGTTCATGCCTCGCATGGTTGTAATTCCCCCTGGGGTGGAATTTCATCACATTGTTCCACATGAAGGTGATATGGACGGTGAAACTGAAGGAAATGAAGATCAGCCTACTTCTCCAGACCCACCCATTTGGCCTGAGATAATGCGCTTCTTTACTAATCCACGCAAGCAGATGATACTTGCCCTAGCTAGGCCAGATCCCAAAAATAATCTCACAACTTTGGTTGAAGCATTTGGGGAATGTCGTCCATTAAGAGAGCTGGCTAATCTGACTTTAATAATGGGTAACCGAGATGATGTTACTGAAATGTCAAGCACTAATTCTTCGGTTCTTCTCTCAATACTTGAGCTTATTGATAAGTATGATCTCTATGGTCAAGTGGCATACCTCAAACACCACAAGCAGTCTGACGTTCCTGATATCTACCGTCTGGCAGCTAAGACAAAGGGCGTTTTCATTAATCCAGCTTTCATTGAGCCGTTTGGGCTTACTCTGATCGAGGCAGCAGCTTATGGCTTACCAATTGTCGCCACAAAAAATGGAGGTCCTGTTGACATACATTGGGCTCTTGACAGTGGTTTCCTTGTGGACCCCCATGATCGGCAGTCTATTGCTGATGCTCTTTTAAAGCTGGTTGTGGATAAGCAACTTTGGGCCAAGTGCCGTCAGAATGGGTTAAAAAATATTCACCTTTTCTCATGGCGAGAACATTGTAAAACTTACCTATCTCGAATAGCAGCTTGCAAACTGAGGCAACCATGGTGGCAAAGAAATCAGGACAGTACTGCTGGAAAGTTCCCAGCGTTGAGGAGGAAGAATATTATTGTTATTGCCGTGGATTTTGGTGCTATATCGGATCTCTCCGAAAGTATTAGAAAGATATTTGACGCTGTGGCGAAGGAAAGGACTGAAGGCTCTATAGGATTCGTATTAGCTACATCCTTTACTTTGTCTGAAGTCCAGTCTTTTCTCATCTCTGGGGGACTGAGCCCTTCTGATTTTGATGCTTTTATCTGCAATAGTGGTAGTGATATCTACTATTCATCTCTTAATCCAGAGGATAATCCCTTCGTTGTTGACTTGTATTACCACTCACATATTGAATACCGCTGGGGTGGAGAAGGGTTGAGGAAGACTTTGATTCGTTGGGCTGGTTCTATCACTGATAAGAAGGGTGAAAATGAAGAACAGATTGTTACCGAAGATGAAAAGATTTCCACCAATTATTGTTATGCTTTCAAAGTGCGAAACGCAGGAAAGGTTCCTCCTGTTAAAGAAATCAGAAAATTAATGAGGATTCAGGCTCTCCGTTGCCATGTTATTTATTGCCAGAATGGGAATAAGATAAATGTAATTCCGGTATTGGCATCTCGTTCCCAAGCCCTAAGGTATCTCTATCTCCGGTGGGGCGTGGACTTATCAAAAATGGTGGTTTTTGTCGGAGAAACTGGCGACACTGATTACGAGGGCTTGCTCGGTGGCATACACAAGTCTGTAATATTGAAGGGAGTTTGTAGCGGTCCGACCCATCAACTCCATGCTAACAGAACCTACCCTCTTTCCGACGTCCTGCCGATCGACAGCCCCAACATTGTTCAGGCAGCTGAGAAATGCAGCGGTGCCGACCTACGAACCTCGTTGGGGAAGCTAGGGTTCATCAAGTGCTAG

>AcSPS3

ATGCCTGGAGTTTATCGGGTTGATTTGCTGACAAGACAGGTATCAGCACCAGATGTCGATTGGAGTTATGGTGAACCAACAGAGATGCTAAATACAAGGAGCTCTGAAAATGATATGAAAGAAACTGGGGAAAGTAGCGGTGCTTATATAATTCGTATACCATTTGGTCCGAAAGATAAATACATTCCCAAAGAGTTCCTTTGGCCTCACATTTCTGAATTTGTGGATGGTGCACTTAGTCATGTTATACAAATGTCCAAAGTTCTTGGTGAGCAAATTGGTGGCGGTGAACCAGTCTGGCCTGTTGCCATCCATGGACATTATGCAGATGCAGGTGACTCTGCCTCTCTTCTTTCTGGGGCTCTAAACATCCCAATGCTTATTACTGGCCACTCGCTTGGACGAGACAAGCTTGAACAAATCTTGCAACAAGGACGGCAATCTAAGGAAGAAATAAATGCTACATACAGAATAATGCGGCGTATAGAGGCCGAGGAAATATCTCTTGATGCTTCCGAAGTTGTTATTACCAGCACTAGACAGGAAATAGAAGAGCAATGGCGTCTGTATGATGGATTTGATCCCATAATAGAGCGCAAACTAAGAGCAAGAATCAAGCGCAATGTGAGCTGCCATGGAAGGTTTATGCCTCGCATGGTTGTGATTCCTCCTGGAATGGAGTTCCATCATATTGTTCCACATGATTCCGATATGGACCGAGAAACAGAAGGAAACGAGGATAATCCTGCTACTCCGGATCCACCGATTTGGTCTGAGGTGTTAACTATTCCTTGTCGCTATCTCTCCGCTCAACGAACACCTTGTTTGTCTAATGAAGGGCTCCCTTATATTTTTTACCGGGGTAATTTTCTATTTTTGCAGATTATGCGTTTCTTCACCAATCCTCGCAAGCCCATGATACTTGCACTTGCCAGGCCAGACCCCAAAAAGAACATCACGAATTTAGTCAAAGCTTTTGGAGAATGCAAGCCATTGAGGGAACTTGCTAACCTTACATTAATAATGGGTAACCGTGATGATGTCGATGAAATGTCTGGCACAAATGCGTCTGTTCTCCTTTCAATACTTAAATTGATTGACAAATACGATCTGTATGGTCAAGTGGCATATCCTAAGCACCACAAGCAGTACGAAGTTCCTGACATATATCGACTTGCAGCAAAATCGAAGGCAGCAGCTCACGGTTTGCCTACGGTTGCCACAAAAAATGGAGGTCCGGTTGATATCAATCGGGTGTGTTCTTTGTTGGAACCTTCTTATTGCAAGGTTCTCGATAATGGTCTGCTAGTTGACCCTCATAATCAGCAGTCTATTGCCGATGCTCTTTTGAAACTTGTTGCAGATAAGCAACTTTGGGCAAAATGTAGACATAACGGATTAAAAAACATTCACCTTTTCTCGTGGCCAGAGCATTGTAAGACGTATTTATCTCGAATTGCCAGCTGCAAACCAAGGCAGCCACGGTGGCAAAAAAGTGACGATGGGTATGAGAAGTCAGACTCGGACTCACCGGGTGATTCGTTGAGGGACATACAGGATCTATCTCTAAACTTAAAGATTTCTCTGGACGGGGATAAGGGAAGTGGCACGCTTGATAATGCCTTAGATTGTGAAGAGAATGCGTCTGGTGGAAAGAACCGATTAGAGAATGCAGTTTCGGTATTATCAAAGGGTGAAGAACAAAACCCACGAAAGGCTGGCTCCATGCCGAAACTAGACTACAACAATAGTAAATTTCTAACATTGAGGAGGCGGAAGTTCGTCTTTGTCATTGCTGTGGATTGTGATATGACTTCCGAGTATCTTAAAATGGTCAAAGTGATTGTTGAGGCTGCGGGGGAGAATAAGTCAGGCTTTATAGGCTTCATATTGTCGACAGCCTCGAGCATACCGGAGATATTCTCTTGTCTGAAGTCAGGAGGCTTGAATCCTATGGACTTCGATGCTTTTATTTGCAATAGCGGTAGTGAACTCTACTATCCATCTTCAACTTCTGAAGTTGGTCCTTTTGGGCTTCCAGTCGTAGCAGACTCAGACTATCATTCCCACATTGATTACCGTTGGGGAGGAGAAGGTTTGAGGAAAACTTTGGTCCGCTGGGCTGCTTCTATGAATGACAAAAAAGGAGCAGGAGGAGGACAAGTTGCTGAAGACGAATCCAGATCGGCTACGCATTGCTGTGCATTCAAAGTGACGAACCCAGCAATGCTTCCTCCTGTTAAGGAACTTCGGAAATTGATGCGGATTCAGGGTCTTCGATGCCACATTATTTATTGTCAGAATGGTATAAAGATGAATGTGATTCCCGTGGTAGCTTCCCGATCCCAGGCCCTCAGGTACCTGTATGTTCGATGGGGCGTGGACTTGTCGAGTATGGTGGTCTTCGTAGGAGAATGCGGGGACACGGATTACGAAGGATTGCTCGGTGGAGTACACAAAACTGTGATACTAAAAGGTGTGTGTGTCGATGCACGTAAACTACATACTAACAGAAACTATCCTCTCGAACACGTCGTGCCGTCCGACAGCCCTAATCTTGTGGAGTCTGAAGGTTGCAACAACACCAACATTAGAGCAACATTGGCGCCAGATGTGTCCACTTCCAACCCCAAGCTATTCAAAAACTTAACCAGGGTTCTTTGCAATGCCAACACTTCAGAAGGTTTCCAACCACCGAGAGATGTCTCCTTGCGGGAAATAAAAATCCCACACAACCACCTTGGGCCACCCCGCCTTGGCCAGCCTCCAAACAACCACTCCATCCTCGCCTTCTTTGCAGGCGGTGACCACAATCACGCACGAAAATCCTACTCAGATACTGGAAAGACAAAGATAACAATATTCAAGTCCACGACTACCTCCCAAAACCCTAAATTACTTCGAGCTAATGGGTCGAGTCAAGTTGTGCTTATGTGCTAG

>AcSPS4

ATGGCCGGGAACGAGTGGATAAATGGGTACTTGGAGGCGATTCTTGACAGCGGCGCGGCGGCGATTGAGGATCAGAGCAAGCCAATCCCCGTGAATCTTAGAGAGAGAGGGAATTTCAATCCCACCAAGTATTTTGTCGAAGAGGTTGTTACTGGGGTCGATGAGACCGATCTTCACAGAACATGGATCAAGGTGGTTGCGACTCGCAATACTCGCGAGAGGAGCTCCAGATTGGAGAACATGTGTTGGAGGATTTGGCATCTCACTCGCAAGAAGAAGCAGTTGGAATGGGAGGATTTCCAAAGGATGGCACACCGAAGATGGGAACGTGAACAAGGACGCAGGGATGCAACAGAAGACATGTCCGAAGACTTGTCTGAAGGAGAGAAGGGAGATGTACTTGGAGAAACAATGGTGAATGAAAGTCCGAGGAAAAAGTTCCAGCGTAACTTTTCCAACTTGGAAGTATGGTCGGATAATAACAAGGAGAAGAAGCTTTACATTGTTCTTATCAGTTTGCACGGTTTGGTCCGTGGGGAAAATATGGAGCTTGGGCGTGATTCCGATACTGGTGGTCAGATCAAATATGTGGTAGAACTTGCTCGGGCCCTGGCTAGGATGCCGGGGGTATATAGGGTCGATCTGTTTACCCGCCAAATCTCTTCTCCCGAAGTCGATTGGAGCTATGGAGAGCCCACAGAGATGCTCACCGCAGGTGCCGATGACGATGCTGATGTTGAAGAAAGCAGCGGGGCTTACATTGTAAGGATACCCTTTGGTCCACGTGATAAGTACATGAGAAAAGAATTGTTGTGGCCCTATATTCAAGAATTTGTAGATGGAGCTTTGGCTCACATTCTCAATATGTCAAAGGCTTTGGGTGAACAAATTGGACATTCACTAGGTAGAAACAAGTTAGAACAGCTTCTCAAGCAAGGAAGGCAATCAAAAGAGGATATCAATTCTACGTATAAGATTATGAGGAGGATAGAGGCGGAAGAGCTTTCCCTTGATGTTGCAGAGCTTGTTATCACGAGCACCAAGCAGGAGATTGACGAGCAATGGGGGCTTTATGACGGGTTTGATGTCAAGCTTGAGAAAGTTTTGCGGGCACGTGTTAGACGAGGGGTCAATTGCCATGGACGATACATGCCAAGGATGGCGGTTATTCCTCCAGGTATGGACTTCAGCAATGTTGTGGTTCAAGAGGATACCGCTGAAGCTGACGGGGAGCTGACAGCACTAACTACTGCAGACGGGTCTTCTCCAAAAGCTTTCCCACCAATATGGTCAGAAATGATGCGTTTTCTAACAAATCCCCATAAACCAATGATCTTGGCCTTATCAAGACCTGATCCGAAAAAGAATATCACCACTCTTTTGAAAGCATTTGGAGAATGCCGCCCGTTGAGAGAACTTGCTAATCTGACGCTTATAATGGGAAATAGGGACGATATTGATGAGATGTCCGCTGGGAACGCTAGTGTCCTCACCACAGTTCTGAAACTGGTTGACAAATATGATCTGTACGGGCAAGTGGCTTTCCCAAAGCATCACAAGCAAAGTGATGTTCCGGAAATCTACCGCCTTGCTGGGAAAACTAAGGGGGTCTTCATAAATCCAGCTTTGGTTGAGCCTTTTGGGCTTACCCTAATTGAGGCTGCAGCACATGGACTTCCAATGGTGGCAACTAAAAATGGTGGTCCAGTTGACATTCATCGGGCTTTGAATAATGGTCTGCTTGTCGACCCACATGATCAGGAAGCAATTGCTAATGCGCTGCTTAAACTAGTGTCAGAGAAGAATTTATGGCATGATTGCAGAAAGAATGGTTGGAAGAACATACACTTATTTTCATGGCCCGAACACTGCCGCACATACTTGACTAGGGTGGCAGCATGTAGAATGCGACACCCTCAGTGGCAAACTGACACCCCGGCAGATGAATTTGCCGCTGAAGAGTCCCTAAATGACTCACTCAAGGACGTGCAAGACATGTCCCTGAGGCTGTCAATTGATGGGGAAAGGACATCACTTAATGAATCGCTACTATGTAACTGCAACTGGTGGTGGCCCTGAGCTCCAAGACCAAGTGAAACAGCTACTAAGCAGGATGAGGAAACCAGAAACAAAAGCTCAAGATTCCGAAGGCAGTGGGAAACTTGTTGATAATATTGCAAGCAAATATCCGATGTTGAGAAGGAGGCGTCGGTTGATTGTCATAGCACTTGATTGCTATGACAGCGATGGAGCCCCCGAGAAAAAGATGATTCGGATAATTCAAGAGATATTTAGGGCTGTTAATGTAGTCTCTCAAACTGCTAGGTTCTCGGGATTTGCTCTATCGACAGCTATGTCAATGTCGGAGCTGAAAGCCTTCTTGAAAACTGGGAATATTCAAGTAAATGAGTTCGATGCTTTGATTTGTAGCAGTGGGAGTGAAGTCTACTACCCGGGTACTTATACACAAGAAGACGGGAAGCTTTATCCTGACCCAGACTATGCCACACATATTGACTACCGTTGGGGTTGTGATGGCTTGAAAAAGACTATTTGGAAGCTGATGAATTCACATGAAGGCGGGTCTTCTCATTCTAAAAGCCCAATTGAAGCAGATGTGAAATCTAGTAATTCGCATTGCGTCTCCTACTTGATAAAGGATCTCAGTAAGGCAAAGAAAGTGGATGATATGAGGCAGAAGCTGAGGATGCGGGGTCTCCGTTGCCATCTGATGTACTGCAGAAACTCAACAAGAATGCAAGTTGTCCCTCTTCTTGCATCTCGAGCACAAGCACTGAGGTACCTTTTTGTTCGTTGGAGACTAAACGTATCTAACATGTATGTGATTCTCGGTGAAACTGGAGACACTGATTATGAGGAACTGATATCTGGCACCCATAAGACGCTAATCATGAAAAACATGGTGGAGAAAGGTTCCGAAGAGCTGCTCAGAGCAGCAGGAAGCTATCTAAAAGATGACATTGTCCCCGAGGAGAGCCCACTGGTGACCTACACAAGTGGAGAAGCGAAAGCCGATGACATTGCAAATGCTTTGAAGCAAATCTCTAAATCTAGCCCGGGAATATGA

>AcSPS5

ATGGCGAATAACGAGTGGATAAACGGGTACTTGGAGGCGATTTTGGACGCGGGGAGTCGGAGAAGCGGATTGAGAGAGAATGGCTATGCTGATGAAGGAATTAGAAGCAACAATAGCATGAACAACATGAGTATAAGGAAAAGGCTTGAAGACAAGTTGAGAATCGAAAAGTTCGACGATGACAAAGGAAAGGAAGAGAAGCTGTTTAGTCCCACCAAGTATTTCGTTGAGGAAGTTGTTAATTGCTTTGATGAGTCTGACCTCCACAGGACATGGATCAAGATAGCTTGGGATGATGCAAAAAGGCTTGCAAAAAGACGAATCGAGCGTGAGAAAGGTCGGAATGATGCTGCAGAAGACCTGTCTGAGCTTTCCGAAGGGGAGAAGGAAAAGGGTGATGCCAATCAGACAGAGCCTATATCAGAAAAGATTTCCAGAATTAACTCTGACATGAAAATATGGTCAGATGATGATAAATCTAGGCGCCTCTACATTGTCCTAATCAGTCTACATGGATTGGTGCGTGGGGAAAATATGGAACTCGGAAGAGATTCAGACACTGGAGGTCAGGTGAAGTATGTAGTAGAGCTTGCCCGAGCCCTGGCCAACATGAAAGGAATCTATCGTGTTGACCTTCTGACTCGACAAATCACCTCGTCGGAGATTGACTTTAGCTATGGTGAGCCCAATGAGATGCTCTCGTGCCCATCCGATGGCAGTGGTAGTTGTGGTGCTTACATTATTCGGATCCCATGTGGACCTCGCGACAAGTACATTCCAAAAGAGTCACTCTGGCCTCACATACCAGAATTTGTAGATGGGGCCCTAAGCCACATCGTGAACATGGCAAGAGCTATAGGAGAACAAGTGGATGGGGGAAAGCCGACGTGGCCTTACGTAATTCACGGTCACTATGCTGACGCAGGGGAGGTAGCAGCACGCCTATCCGGGGCATTGAATGTGCCAATGGTGCTAACGGGGCACTCCTTAGGAAGAAACAAATTTGAGCAATTACTTAAA

CAAGGGAGGTTGTCTAGGGAAGATATAAATTCAGCTTACAAGATAATGAGGAGGATTGAGGCTGAAGAGTTGGGGTTGGATGCTGCTGAAATGGTGGTAACTAGCACGAGGCAAGAGATCGAAGAGCAATGGGGTTTATACGATGGGTTTGATATCAAGTTGGAGAGGAAGCTTAGGGTTAGGAAACGGCGTGGAGTGAGTTGCCTTGGTCGATACATGCCAAGGATGGTGGTTACACCACCGGGGATGGACTTCAGCTATGTCACAACACAAGATTCATTGGAAGGTGACGGAGATCTAAAGTCGTTGATTGGCTCTGATAGAACTCAAAACAAAAGGCACATACCTCCAATATGGTCCGAGGTAATGCGATTTTTCACAAACCCTCACAAACCTATGATCCTAGCATTGTCACGTCCAGACCCCAAGAAAAATGTGACCACTTTGCTCAAGGCTTTTGGAGAATGCCGACCACTCAAAGAATTAGCCAACTTGACCCTAATACTTGGTAACAGAGACGACATAGAAGATATGTCTAACAGCAGTTCAGTAGTTCTCACAACAGTGCTTAAGCTCATTGACAAGTATGACTTGTATGGTCAGGTAGCATATCCCAAACATCACAAACAATCCGAAGTTCCTGAGATCTATCGGCTGGCTGCAAAAACAAAGGGAGTTTTCATCAACCCAGCCCTGGTTGAACCATTTGGTCTCACACTCATTGAGGCGGCTGCTTATGGTTTACCAATTGTTGCCACGAAAAATGGTGGCCCTGTGGATATTCTCAAGGCACTCAACAATGGCCTTCTCATTGATCCACATGACCAGAAAGCCATAGCAGATGCCCTCCTAAAGCTTGTAGCTGAAAAGAATCTTTGGCTTGAGTGCCGCAAAAATGGCCTAAAGTATATTCATCGTTTTTCGTGGCCAGAACATTGTCGTAACTATCTTTCCCACGTCGAGCATTGCAGGAACCGCCATCCTACAACCCGTCTTGAGATCATGCCAACTCCTGAAGAACCCATGAGCGAATCGTTAAGGGATGTGGAAGACATTTCTTTAAAATTCTCCATTGATGCAGACTTCAAGCTCAATGGAGACCTTGATGTACCAAATAGACAATGCAAACTCATCGAGGCCTTAACACAGATGGGTTCCTCCAATAGCCCTTCCAGCACTAGTTACTGTCCAGGAAGAAGGCAGGCGCTATTTATAATTGCTACTGATTGTTATAACAGTGGTGGAATGTGCACTGAGACCTTTCCATTGGTCATCAAGAATGTGATGCAAGCTGCAGTCTCAAACTCAGGCAAGATAGGCTTTATATTGTCAACAGGTTTAACTTTACTTGAGACAAAGGAATTGTTAAGACATTGCCATGTAAATTTGGAAGATTTCGACGCATTTGTATGCAATAGTGGAAGTGAAATGTACTATCCATGGAGAGACTCCACAGCTGATATGGATTATGAAGCCCATATTGAATACAGGTGGCCCGGCGAGAATGTGAGATCAATGATAATGAGGCTTGGTCGGGTAGGAGATGGAGATGGAGATGAATATGATATTATGGAAAGTCTAGATGCATTTAGCTCCCGATGCTATTCTTATAGCATTAAACAAGGAAGCAAGACTCGAAGGATCGATGAACTGCGCCAGAGGCTACGCATGAGAGGTTTACGGTGCAATGTTATCTACACTCGTGCTTCATCACTGTTGAAGGTAGTACCTTTATTTGCATCAAGAGCTCAAGCACTAAGGTGGGCTATTGATCTTTCCAAAATGGTAGTGTTTGTCGGAGAAAGAGGGGATACAGACTATGAAGACCTGTTGGTTGGCCTACACAAGACTGTTATTCTAAGAAATTCTGTAGAATATGGCAGTGAGATGCTTCTGCGCAGTGAAGAAAGTTTTAAACGTGAAGATGTGGTCCCCCAAGATAGCCCTAGGATTGCCTTTGGTGCATGTTATGAAACCCATGATATCTCTGCAGCTTTAGATGCTCTACAGGTCATATGA

>AcSPS6

ATGAGTAGGAGAGCTGAAGAACAAAAGAAAGAAGGGATCGACGACGCGAAATCGTCGTTGCTGCTTAGAGAGAGAGGCAGGTTCAGTCCCACTCGCTACTTCGTCGAGCAGGTCATCGGCTTCGATGAGACCGATCTCTATCGCTCCTGGGTTAAGCTTGAGGGAGAGGAAGCTCAAAGGATGGCTAAACGTCGTCTTGAACGTGAAAGAGGCCGCAGAGAAGCAACTGCTGATATGTCTGAAGACTTGTCCGAGGGGGAAAAAGGAGATAAGGTCAGTGATTTGTCTGCTCATGGTGAAAGCAACAGGGGCCGATTACCTAGAATTAGCTCCGTTGAGACAATGGAGGCATGGGTTAGTCAACAGAAGGGGAAAAGGCTCCTTCACGGTCTAATACGGGGTGAAAATATGGAGCTTGGTCATGATTCTGATACTGGTGGCCAGGTTAAGTATGTTGTGGAACTTGCAAGGGCTTTGGGTTCAATGCCAGGAGTGTATCGCGTTGATTTACTCACTAGACAAGTATCATCACCAGAAGTAGACTGGAGTTATGGTGAACCCACTGAAATGTTGCCTCCAAGAAATTCTGATGTTTTAATGGATGAGATGGGGGAGAGTAGCGGTGCTTATATTATTCGTATTCCATTTGGCCCAAGAGATAAATATGTACCGAAAGAACTTCTGTGGCCACACGTTCCTGAATTTGTTGATGGTGCTCTTAACCACATCATACAGATGTCCAAAGTACTTGGTGAGCAAATTGGCAGTGGGCATCCTGTGTGGCCTGTTGCTATCCATGGGCATTATGCAGATGCAGGTGATGCCGCTGCTCTTCTATCAGGTGCTCTAAATGTACCCATGCTTTTCACTGGTCACTCACTTGGTAGGGATAAGCTGGAACAGCTTTTGAGGCAAAGTCGACTATCAAAGGATGAAATAAATAAGACGTACAAAATAATGCGTCGTATAGAGGCTGAGGAATTATCTCTTGATGCCTCTGAAATAGTGATAACTAGCACTAGACAAGAGATAGAACAGCAATGGCGTCTGTATGATGGTTTTGATCCTGTACTTGAACGGAAACTACGAGCCAGGATCAGGCGTAATGTGAGCTGTTATGGCAGGATCATGCCTCGCATGGTTGTAATTCCCCCTGGGATGGAATTTCATCACATTGTTCCACATGAAGGTGATATGGACGGTGAAACTGAAGGAAATGAAGATCAGCCTACTTCTCCAGACCCACCCATTTGGCCTGAGATAATGCGTTTCTTTACTAATCCACGCAAGCCGATGATACTTGCCCTAGCTAGGCCAGATCCCAAAAAGAATCTCACAACTTTGGTTGAAGCATTTGGGGAATGTCGTCCATTAAGAGAGCTGGCTAATCTGACTTTAATAATGGGTAACCGAGATGATGTTGATGAAATGTCAAGCACTAATTCTTCGGTTCTTCTCTCAATACTTAAGCTTATTGATAAGTATGATCTCTATGGTCAAGTGGCATACCCCAAACACCACAAGCAGTCTGAAGTTCCTAATATCTACCGTCTGGCAGCTAAGACAAAGGGCGTTTTCATTAATCCAGCTTTCATTGAGCCATTTGGGCTTACTCTGATCGAGGCAGCAGCTTATGGTTTACCAATTGTCGCCACAAAAAATGGAGGTCCTGTTGACATACATCGGGCTCTTGACAATGGTCTCCTTGTGGACCCCCATGATCGGCAGTCTATTGCTGATGCTCTTTTAAAGCTGGTTGCGGATAAGCAACTTTGGGCCAAGTGCCGTCAGAATGGGTTAAAAAATATTCACCTTTTCTCATGGCCAGAACATTGTAAAACTTACCTATCTCGAATAGCAGCTTGCAAACTGAGGCAACCATGGTGGCAAAGAAGTGACGATGGGAATGAAAATTCAGAGTCAGATTCACCAAGTGACTCCTGGAGAGATATACAGGATATATCCTTGAACTTAAAGTTTTCACTGGATGGAGAAAAGAACGAAGGTAGCGGAAATGCTGACAGTTCTTTAGACTTTGAAGATCGCAAGAGTAAGTTGGAGAATGCTGTTCTGACATGGTCAAAGGGAGTCCAGAAAGGCACACAAAAGGCTGGGCTTACGGAGAAAGCAGATCAGAACAGTACTGCTGGAAAGTTCCCAGCGTTGAGGAGGAGGAAGAATATTGTTGTTATTGCCATGGATTTTGGTGCTATATCAGATCTCTCCGAAAGTATTAGAAAGATATTTGATGCTATGGCGAAGGAAAGGACCGAAGGCTCTATAGGATTCATATTAGCTACATCCTTTACTTTGTCTGAAGTTCAGTCTTTTCTCATCTCTGGGGGACTGAGCCCTTCTGATTTTGATGCTTTTATCTGCAATAGTGGTAGTGATCTCTACTATTCATCTCTTAATTCAGAGGATAAACCCTTCGTTGTTGACTTATATTACCACTCACATATTGAATACCGCTGGGGTGGAGAAGGGTTGAGGAAGACTTTGATTCGTTGGGCTGGTTCTATCACTGATAAGAAGGGTGAAAATGAAGAGCAGATTGTTACCGAAGATGAAAAGATTTCCACCAATTATTGTTATGCTTTCAAAGTGCAAAATGCTGGAAAGGATCCTCCTGTTAAAGAAATCAGAAAATTAATGAGGATTCAGGCTCTCCGTTGCCATGTTATTTATTGCCAGAATGGGAATAAGATAAATGTAATTCCGGTATTGGCATCTCGTTCCCAAGCCCTAAGGTATCTCTATCTCCGGTGGGGTGTGGACTTGTCAAAAATGGTGGTTTTTGTTGGAGAAAGCGGCGACACTGACTACGAGGGCTTGCTTGGTGGCATACACAAGTCTGTAATATTGAAGGGAGTTTGTAGCGGTCCGACCCATCAACTCCATGCCAACAGAACCTACCCTCTTTCCGACGTCCTGCCAATTGACAGCCCTAACATTGTTCAGGCAGCCGAGGAATGCAGCGGTGCCGACCTCCGGACCTCGTTGGGGAAGCTAGAGTTCATCAAGGGCTAA

>AcSUS1

ATGGCAGCCTTGAAGAGGTCTGACTCGATAGCTGATAGCATGCCGGATGCCTTGAGAGAAAGCCGGTACCACATGAAGAAGTGCTTAGCTAAGTACATTGAGAAGGGGAAGAGGTTGATGAAACTTCACCACTTAATGAGCGAAATGGAGAAAGTGATCGATGATAAGACCGAGAGAGAGCAGATCTTGAACGGCCTTCTTGGCTACATTTTATGCACCACTCAGGAGGCAGTTGTTATTCCTCCATATGTTGCCTTTGCAATTAGACCAAATCCCGGGTTCTGGGAATTCGTTAAAGTGAGCTCTACAGATCTATCAGTAGAGGGCATCACCGCCACGGACTACTTGAAATCCAAAGAAATGCTGGTTGATGAGGACTGGGCAAAGGATGAAAATGCTTTAGAAGTTGATTTTGGCGCGATGGACTTTTCCGAGCCTAACCTGACCATGTCTTCTTCGATTGGGAACGGAATCAATTTTATTTCCAAATTCCTTTCTTCTATACTATATGGTGGCTCACAGAAGGCTCAGCCTCTTGTTGATTACCTACTCTCACTAAATCACCATGAAGAAAAACTAATGATTAACGAGACCCTCAACACCGCTGCCAAGCTTCAGAGCGCGCTAATAGTAGCTGAAGCGGCCCTTTTGACACTGCCCAAGGACACACCATACCAGGACTTTGAGCAAAGGTTTAGGCAGTGGGGTTTTGAGAAGGGATGGGGCGATACTGCAGAAAGAGTGAGGGAGACAATGAGATCGCTTTCAGAGATATTCCAGGCACCGGACCCGTTAAACATGGACAAGTTCTTTGGCAGGGTTCCAACTGTTTTCAATGTTGTTTTGTTTTCGGTCCATGGGTATTTTGGTCAATCTGATGTCCTCGGTTTGCCAGATACCGGTGGGCAGGTGGTCTATGTATTGGATCAAGTAGTTGCTTTTGAAGAAGAACTGCTCGTTCGGATTAAGCAGCAAGGGCTTAATGTGAAGCCTCAAATTCTTGTGGTCACTCGACTTATCCCCGATGCCAAGGGAACTAAGTGCAACCAGGTGCTAGAACCGATTGCCAACACAAAGCATTCCAACATCCTTCGCGTGCCATTTAGGACGGAAGATGGAGTTCTTCCGCAATGGGTTTCTCGTTTCGACATATATCCCTACCTCGAAAGTTCTGTTTTCAATCAACAGGACGCTACGGACAAAATCTTGGAAGTCATGGAAGGGAAACCGGATCTCATCATCGGAAACTACACAGATGGGAATTTGGTGGCATCACTCATGGCTAGCAAACTTGGGATTACTCTGGGAACTATTGCACATGCTTTGGAGAAGACAAAGTATGAGGATTCAGACCTAAAATGGAAACAATTAGACCCCAAGTATCATTTCTCATGCCAATTCACCGCAGACACGATCGCAATGAATTCTGCAGATTTCATCATCACCAGCACATACCAAGAAATTGCTGGAAGCAAAGATAGGCCGGGGCAGTACGAAAGCCACGCTGCATTTACACTTCCAGGGCTTTGCAGAGTTGTTTCAGGCATAAATGTGTTTGATCCCAAATTCAATATAGCTGCTCCCGGGGCAGACCAATCCGTCTATTTCCCTTACACAGATAGACAGAAGCGATTCACTTCATTTCGTCCTGCCATAGAAGAACTACTCTTTAGCAAAGTTAATAACAATGAGCACATTGGATATCTAGAAGACAGGAAGAAGCCTATTCTGTTCTCAATGGCAAGGCTTGATATAGTGAAGAACATCAGTGGATTAACCGAGTGGTACGGGAAGAACAAGAGGCTGAGAAGTTTGGCTAATCTCGTTGTTGTCGCAGGGTTCTTTGATCCTACTAAATCCAAAGACAGAGAAGAGGCAGCCGAAATAACAAAAATGCACATGTTGATTGAGAATTACAAACTTAAGGGTCAGATTCGATGGATAGCAGCACAGACTGACAGGCAACGAAACGGGGAGCTGTACCGCTGCATTGCTGACACAAAAGGAGCATTTGTGCAGCCTGCACTTTACGAGGCGTTTGGCCTCACGGTCATTACAGCAGTTGTATCTGTTGGCGTCATCTTCATCCCCATTGGCCTTCCTTGTTTGCATCAGAACATGTAA

>AcSUS2

ATGGCAGCCTTGAAGAGGTCTGAGTCGATGGCTGATAGCATGCCGGACGCCCTGAGAGAGAGCCGGTACCACATGAAGAAGTGCTTTGCTAAGTACATTGAGCAAGGAAAGAGATTGATGAAACTTCGACACTTAATGAGCGAAATGGAGAAAGTGATCGATGATAAGACTGAGAGAGAGCAGTTCTTGAACAGCCTACTTGGCTACATTTTGTGCACCACTCAGGAGGCGGTTGTTATTCCTCCATATGTTGCCTTCGCCATTAGACCAAATCCTGGGTTCTGGGAATTCATTAAGGTGAGCTCTACCGATCTATCGGTGGAGGGCATCACTGCCACGGACTACTTGAAATACAAAGAAATGTTGGTTGATGAGGACTGGGCAAAGGATGAAAATGCATTGGAAGTTGATTTTGGAGCGATGGACTTTTCCACGCCTAGCCTGACCGTGTCTTCTTCGATTGGAAACGGAATCAATTTCGTTTCCAAATTCCTTTCTTCTAAACTACATGGTGGCTTACAGAAGGCTCAGCCTCTTGTTGATTACTTACTCTCACTAAATTACCATGAAGAAAAACTAATGATTAACGAGACCATCAACACTGCTGCAAAGCTTCAGAGCGCGCTAATAGTAGCTGAAGCGGCCCTTTTGACACTGCCCAAGGACACACCATACCAGGACTTCGAGCAAAGGTTTAGGCAGTGGGGATTTGAGAAGGGATGGGGCGATACTGCGGAAAGAGTGAGGGAGACAATGAGATCTCTTTCAGAGATATTCCAGGCACCGGACCCGTTAAATATGGAGAAGTTCTTTGGCAGGGTTCCAACGGTTTTCAAAGTCGTTTTGTTCTCGGTCCATGGGTATTTTGGTCAATCCGATGTCCTCGGTTTGCCAGACACCGGTGGGCAGGTGGTCTATGTTTTAGATCAAGTAGTTGCTTTTGAAGAAGAACTGCTTGTTCGGATTAAGCAGCAAGGGCTTAATGTGAAGCCTCAAATTCTTGTGGTCACGCGACTCATCCCCGATGCCAAGGGGACTAAGTGCAACCAGGCGTTGGAACCGGTCGCCAACACCAAGCACTCTAACATCCTTCGGGTTCCATTTAGGACAGAAAATGGAGATCTTCCGCAATGGGTTTCCCGTTTCGACATCTACCCCTACCTCGAAAGTATCACTTTATTTTTCAATCAACAGGACGCTACGGACAAAATCTTGGAAATCATGGAAGGGAAACCGGATCTCATCATTGGAAACTACACAGATGGGAATTTGGTGGCATCGCTCATGGCTAGCAAACTTGGCATAACTCTGGGAACTATTGCACATGCTTTGGAGAAGACAAAGTATGAGGATTCAGACCTAAAATGGAAAGAATTGGACCCCAAGTATCACTTCTCCTGCCAATTCACCGCTGACACGATCGCAATGAATTCTTCAGATTTCATCATCACTAGCACATACCAAGAAATTGCTGGAAGCAAGGATAGGCCGGGGCAGTACGAAAGCCATGCTGCATTTACGCTTCCAGGGCTTTGCAGAGTTGTTTCAGGCATAAATGTGTTTGATCCCAAATTCAATATAGCTGCTCCCGGGGCTGACCAATCCGTCTATTTCCCTTACACTGATAGACAGAAGCGATTCACAAAGTTTCATCCTTCCATAGAAGAACTACTCTTTAGCAAAGTTGATAACATTGAGCACATTGGATATCTAGAAGACAGGAAGAAGCCTATTCTCTTCTCAATGGCAAGGCTTGATATAGTGAAGAACATCAGCGGATTAACCGAGTGGTACGGGAAGAACAAGAGGCTTAGAATTTTGGTTAATCTCGTTGTTGTCGCGGGGTTCTTCGACCCTACTAAATCCAAAGACCGAGAAGAGGCAGCCGAAATAAAGAAGATGCACATGTTGATCGAGAAATACCAACTTAAGGGTCAGATTCGATGGATAGCAGCACAGACCGACAGGCAACGAAACGGGGAACTGTACCGTTGCATTGCTGACACGAAAGGAGCGTTTGCGCAGCCTGCACTTTATGAGGCGTTTGGCCTCACGGTGAAGAATGTCCCCACTTCAAGGGTTGAACCTCAACAACAGCCCAATGAAAAACAACCTAAAGCACAGCCCTCTCAAAGTTTTTTCATTAATAAACCAATTGGCTGGTGTTTGTTACATGTTCTAGATTCGAAGTTTACGCAACAAGATCTTCCTGCTTGCAAACCAATTCTAACTCATGGATGGGTCCCAAAGCAAATGAAAAGTCCCATCTATATATATTATCAGCTAGACAACTTCTACCAGAATCATCGCCGGTATGTTAACAGTAGAAGTGACGAGCAATTGCGCAGCAAGGCATATGAGTTTGACACAGATGACTGTGACCCGGAATCAAATACAGACAAAGGTCCCATAGTTCCTTGTGGCCTTATTGCTTGGAGTTTGTTCAACGACACATATGGGTTTTCCTTGCAGAACAAAGTTCTCCAAGTCAGTAAAAAGGGCATTGCATGGAAAAGCGACCAAAAGCATAAATTTGGGTCCGACGTCTATCCTAAAAATTTTCAGAGCAGTGGTTTGATTGGGGGTGCAAAACTCAATAAAAGCATACCCTTGAGCAACCAAGTGGATCTTATCGTTTGGATGCGAACTGCGGCATTGCCAACTTTCAGAAAGCTCTATGGGAAGATAGAGGTGGATCTTGAAGCGAATCAAAAGATAACAGTGGTAATACAGAACAATTATAACACGTATAGCTTTGGGGGTAAAAAGAAGCTGGTCCTTTCAACCACAAGTTGGTTTGGTGGAAAAAATGATTTCCTGGGTATAGCATACCTTACAGTTGGTGGACTTTCCTTATTTATGGCAATAAGCTTCATACTTTTGTATGTTGTTAAGCCAAGAAACATAACCAGTGCTGTGAGGAAGCACATTTGCAAGACTAGCATAGGCTGGAAATTCGTGGGAGAAGTGCAAAGCAGTAACTTTGACATGGCGGGGACAAAGAAGACCAAGAGGCTTAAAGCCCAAGTCAAGGTAGCTAAGTTGGCCAACATATGGAAGTTCATCGACCAAAGCTGCTATTGA

>AcSUS3

ATGATGGAAAAACAAAACGAAACAATCATACCATGGGCAGTGCAGTATGTGGAGCAGGGGAAGGGGATATTGCAACCGCATCACTTGATCGACGAGCTTGATAAGATCGTCGGCGATGACGAAGCGAATCTTACTCTCATCAATGGTCCGTTCGGCGAAGTCCTCAAGTCTGCACAGGTGTTAATGTTAAACGATCGAATATATAGCATGCCCAGACTTCAGTCTGCATTGACTAAGGCAGAGGGTTATCTTGCTAAGCTATCAGCTGATACACCCTATTCTGAGTTTGAACATGACTTTCAAGTAATGGGTTTTGAGAGAGGTTGGGGTGATACTGCAGGACGGGTTTTGGAGATGATGCATCTTCTTTTGGATATCCTCCAAGCTCCAGATCCCACATCCTTAGAGACGTTTCTTGGTAGAATACCTATGGTGTTTAATGTTGTCATTTTGTCCGTCCATGGCTACTTTGGCCAAGCAAAGGTTTTAGGATTGCCTGACACAGGTGGCCAGATTGTGTACATACTGGATCAAGTGCGTGCCCTGGAGAATGAAATTCTTATGAGACATAAGCAGCAAGGACTGGATGTCACTCCTAGAATTCTTGTTGTGACACGATTGATACCTGATGCAAAAGGTACTTCATGCAACCAGCGGCTCGAAAGAATAAGTGGGACTCAGCATGCCCATATTCTGCGAGTTCCTTTCAGAACAGATAAAGGAATTCTTCGTAAATGGATCTCAAGATTTGATGTATGGCCTTATCTGGAGAAATTTACAGAGGATGCTGCTAGTGAAATTGCTGCCGAGTTACAGGGTGTTCCAGATCTGATTATTGGCAACTACAGCGATGGAAATCTCGTTGCATCTTTGTTAGCTCATAAGATGGGAGTAACACAGTGCACCATTGCTCATGCCTTGGAGAAAACAAAATATCCTGATTCTGACATATATTGGAAAAAATTTGAAGATAAATACCACTTTTCATGTCAATTTACTGCTGACCTAATAGCCATGAATAGTTCAGATTTTATCATCACCAGTACATTCCAAGAGATTGCAGGAACGAAAAATACTGTTGGTCAGTATGAGAGCCATTCAGCTTTCACCCTTCCAAGCCTGTACCGAGTTGTTCACGGCATTGACGTTTTTGATCCAAAATTCAATATCGTCTCACCTGGGGCAGATATGTGCATTTACTTTCCATACTTTGAAAAGGAAAAAAGGCTTACAGCCCTACATGGTTCGATTGAAAAGTTGTTATATGATCCTGAGCAAAATGAAGAGCACATTGGAACACTGAGTGATTCATCAAAGCCCATAATCTTCTCCATGGCAAGGCTTGACCACGTGAAAAACATCACAGGGCTGGTAGAGTTCTATGCTAAAAATACCAAGCTGAGGGAACTGGTTAACCTTGTTGTGGTTGCGGGTTACAATGATGTGAAGAAGTCAAATGACAGAGAAGAAATTGATGAAATTGAAAAGATGCATAGCCTTATCAAAGAATACAACTTGGATGGCCAGTTTCGTTGGATATCATCCCAAACAAATCGAGCACGCAATGGTGAGCTCTATCGCTACATGGCTGACAAGAGAGGTGCTTTCGTGCAGCCCGCATTTTATGAAGCCTTTGGGCTTACAGTTGTGGAGGCCATGACCTGTGGGCTTCCAACATTTGCCACTTGCCACGGTGGTCCAGCGGAGATTATTGAGGATGGAATATCAGGGTTTCATATCGATCCATATCACCCTGATAAGGTTTCTGCAATTTTAGCAGATTTTTTCCAACGGTGCAAGGATGATCCCAGCTACTGGGAAAAAATCTCTAAAGCCGGCCTTCAAAGGATCCTAGAAAGGTACACATGGAAGATCTACTCGGAAAGGTTGATGACGTTGTCTGGAGTTTATGGTTTCTGGAAATATGTTTCAAAACTCGAGAGACGTGAAACTTTGCGTTATCTAGAGATGTTCTACATTCTCAAGTACCGTGATTTGGTAAAGTCTGTCCCTCTAGCAATTGATGGGGAAGACTAA

>AcSUS4

ATGGTGGCCCGCCCAAGGAAGAAAACAAGACGAGAGAAAAGTGCAAAACCATGGCTATGGATACCTTACGACTACTACGACTACTACGACTATTCTATTCGTCTAGTATTTGCATTTTTCAACATCTTTGACAAACAACACACTTCTAAGACACCGTATTCCGAGTTTGAGCACAAGTTCCAAGAGATCGGTTTGGAGAGAGGGTGGGGTGACACTGCTGAGAGAGTGCTTGAAATGCTTCATATGCTATTGGAGCTTCTTGAGGCTCCAGATCCATGCACTCTTGAGAAATTCCTTGGCAGAATCCCAATGGTTTTCAATGTTGTGATCCTTTCTCCCCATGGCTACTTTGCCCAAGAAAATGTTTTGGGCTATCCTGACACTGGTGGCCAGGTGGTTTACATTTTGGATCAAGTTCCTGCCATGGAGAAGGAAATGCTTAAGCGCATCAAGCAGCAAGGGCTTGATATCATTCCACGCATTCTCATTGTAACTAGGCTTCTCCCAGATGCAGTAGGCACTACCTGCAATCAGCGCATTGAGAAAGTTTATGGAGCAGAACATTCGCATATACTTCGAGTTCCCTTTAGGACTGAGAAGGGAATTGTCCGCCAATGGATCTCGCGTTTTGAAGTCTGGCCTTACATGGAGAGATTCACCGAGGATGTTGCACATGACATCGTCACAGAGTTGCAGGCGAAGCCTGATTTGGTCATTGGCAACTACAGTGAGGGCAACCTTGTTGCCTCATTGTTGGCTCACAAATTAGGGGTAACTCAGTGTACCATTGCTCACGCCTTGGAGAAAACAAAATATCCGGATTCCGACATCTATTTGAAGAAATTTGACGAGAAGTACCACTTTTCGTGCCAGTTCACAGCAGATCTCATTGCTATGAATCACACTGATTTTATAATCACAAGCACTTTCCAGGAAATTGCTGGAAGCAAGAACACCGTTGGACAGTATGAGAGTCATATGGCCTTCACTATGCCCGGACTCTACCGAGTTGTCCATGGTATTGATGTGTTTGACCCCAAATTCAACATTGTCTCACCAGGGGCTGATATGAACATCTACTTCCCTCACACTGAGAAGGACAAGAGACTGACCAAGTTCCACCCTGAAATCGAAGATCTTCTCTTTAGTGATGTGGAGAATAAAGAGCATATTGGGGTGTTGAAAGACCGTACCAAGCCAATCATATTCTCCATGGCAAGGTTGGACCGTGTGAAGAACTTGACCGGACTTGTCGAGTTGTACGGTAAGAATGCCAGACTTAGAGAGCTGGCCAACCTTGTGGTGGTGGGTGGAGATCGTAGGAAGGAGTCCAAGGATTTGGAAGAGCAAGCCGAGATGAAGAAGATGTACGATCTCATTGAAACCTACAAGTTGAATGGTCAGTTTAGATGGATATCTTCGCAGATGAACCGGGTGAGGAATGGGGAACTTTACCGCTGCATTGCTGACACAAAGGGTGTATTTGTTCAACCTGCCTTCTATGAGGCTTTTGGGTTGACGGTGGTGGAGTCAATGACCTGTGGTTTGCCAACATTTGCAACTTGCCACGGTGGTCCGGCTGAGATAATTATTCATGGCAAGTCTGGCTTCCACATTGATCCTTATCATGGTGATCAAGTGGCTGAACTCCTTGTCAATTTCTACGAGAAGTGCAAAGTTGATCCTTCTCATTGGGACGCTATTTCTGAAGGAGGTCTGAAGCGCATCCTGGAGAAATACACATGGCAGATTTACTCTGAGAGGCTAATGACTCTTGCTGGGGTTTACGGCTTTTGGAAGTACGTGTCTAAGCTTGATCGCCGAGAGACTCGCCGTTACCTGGAGATGTTTTATGCACTCAAGTACCGCAAGTTGGCTGAGGCAGTTCCTTTGGCTGTTGATCAGTAG

>AcSUS5

ATGATTCATATGCTGTTGGAGCTTCTCGAGGCCCCAGATCCATGCACTCTTGAGAAATTTCTCGGCAGAATCCCAATGGTTTTCAATGTTGTGATCCTTTCTCCCCATGGCTACTTTGCCCAAGAAAATGTTTTGGGCTATCCTGACACTGGTGGTCAGGTGGTTTACATTTTGGATCAAGTTCCTGCCATGGAGAAGGAAATGCTTAAGCGCATCAAGCAGCAAGGGCTCGATATCATTCCTCGCATTCTCATTGTAACTAGGCTTCTCCCAGATGCGGTAGGCACTACCTGCCATCAGCGCATTGAGAAGGTTTACGGAGCAGAACATTCGCATATACTTCGAGTTCCCTTTAGGACTGAGGAGGGAATTGTCCGCAAATGGATCTCGCGTTTCGAAGTCTGGCCTTACATGGAGAGATTCACCGAGGATGTTGCACATGATATCGTCACAGAGTTGCAGGCAAAGCCTGACTTGATCATTGGTAACTATAGCGAGGGTAACCTTGTTGCCTCATTGTTAGCTCACAAATTGGGGGTAACACAGTGTACCATTGCTCATGCCTTGGAGAAAACAAAATATCCAGATTCCGACATCTATTTGAAGCAATTTGACGAGAAGTACCACTTCTCATGCCAGTTCACAGCAGATCTCATTGCTATGAATCATACCGATTTTATAATCACCAGCACTTTCCAGGAAATTGCTGGAAGCAAGAACACCGTTGGACAGTATGAGAGTCATATGGCCTTCACTATGCCCGGACTTTACCGAGTTGTCCATGGTATTGATGTGTTTGACCCCAAATTCAACATTGTCTCACCGGGGGCCGATATGAACATCTACTTCCCTCACACTGAGAAGGACAAGAGACTGACCAAGTTCCACCCTGAAATCGAAGATCTTCTCTTTAGTGATGTGGAGAATAAAGAGCATATAGGTGTGTTGAAAGACCCTACCAAGCCAATCATATTCTCCATGGCAAGGTTGGACCGTGTGAAGAACTTGACCGGGCTTGTCGAGTTGTACGGAAAGAATGCCAGACTTAGAGAGCTGGCCAACCTTGTGGTGGTGGGTGGAGATCGTAGGAAGGAGTCCAAGGATTTGGAAGAGCAAGCCGAGATGAAGAAGATGTACGATCTCATTGAAACGTACAAGTTGAATGGTCAGTTTAGATGGATTTCTTCCCAGATGAACCGGGTGAGGAATGGGGAACTTTACCGCTTCATTGCTGACACCAAGGGTGTGTTTGTTCAACCTGCCTTCTATGAGGCTTTTGGGTTGACAGTGGTGGAGGCGATGACCTGTGGTTTGCCAACATTTGCAACTTGCCATGGTGGTCCAGCTGAGATAATTATTCACGGCAAGTCTGGCTTCCACATTGATCCTTATCATGGTGATCAGGTTGCTGAACTCCTTGTCAATTTCTACGAGAAGTGTAAAGTTGATCCTTCTCATTGGGACGCTATTTCCGAAGGAGGTCTGAAGCGCATCCTGGAGAAGTACACATGGCAGATTTACTCTGAGAGGCTAATGACTCTTGCTGGGGTTTACGGATTTTGGAAGTACGTCTCCAAGCTTGATCGCCGTGAGACTCGCCGCTACCTGGAGATGTTTTATGCTCTCAAGTACCGCAAGTTGGCTGAGGCAGTTCCTTTGGCTGTTGATCAGTAG

>AcSUS6

ATGTCGACTGCTAAATTGGCGCGAATCCCCAGCATGAGAGAGAGGGTCGAAGACACCCTATCAGCTCACCGCAACGAACTCGTCTCACTCCTCTCCAGGTATGTGGAGCAGGGGAAGGGGATATTGCAACCGCATCACTTGATCGACGAGCTTGATAAGATCGTAGGCGATGACGAAGCGAATCTTACTCTCAGCGATGGTCCGTTCGGCGAAGTCCTCAAGTCTACACAGGTGTTAATGTTAAACGATCGAATATATAGCATGCCCAGACTTCAGTCTGCATTGACTAAGGCAGAGGGTTATCTTGCTAAGCTATCAGCAGATACACCCTATTCTGAGTTTGAACATGACTTTCAAGTAATGGGTTTTGAGAGAGGTTGGGGTGATACTGCAGGACGGGTTTTGGAGATGATGCATCTTCTCTTGGATATCCTCCAAGCTCCAGATCCTACAGCCTTAGAGACTTTTCTTGGTAGAATACCAATGGTGTTTAATGTTGTCATTTTGTCTGTCCACGGGTACTTTGGCCAAGCAAATGTTTTAGGCTTGCCTGACACAGGTGGCCAGATTGTGTACATACTGGATCAAGTGCGTGCCCTGGAGAATGAAATTCTTGTGAGACATAAGCAGCAAGGACTGGATGTCATTCCTAGAATTCTTGTTGTGACACGATTAATACCTGACGCAAAAGGTACTTCATGCAACCAGCGGCTCGAAAGAATAAGTGGGACTCAGCACGCCCATATTCTGCGAGTTCCTTTCAGAACTGATAAAGGAATTCTTCGTAAATGGATCTCAAGATTTGATGTATGGCCTTATCTGGAGAAATTTACAGAGGATGCTGCTAGTGAAATTGCTGCCGAATTACAGGGTGTTCCAGATCTGATTATTGGCAACTACAGTGACGGGAATCTCGTTGCATCTTTGTTAGCTCATAAGATGGGAGTAACACAGTGCACCATTGCTCATGCCTTGGAGAAAACAAAATATCCTGATTCTGACATATATTGGAAAAAATTTGAAGAGAAATACCACTTTTCGTGTCAATTTACTGCTGACCTAATAGCCATGAATAGTTCAGATTTTATCATCACCAGTACATTCCAAGAGATTGCAGGAACGAAAAATACTGTTGGCCAGTATGAGAGCCACACGGCATTCACCCTTCCAAGCCTGTACCGAGTTGTTCACGGCATTGACGTTTTTGATCCAAAATTCAATATCGTCTCACCTGGTGCAGATATGTGCATTTACTTTCCATACTTTGAAAAGGAAAAAAGGCTTACAGCCCTACATGGTTCAATTGAAAAGTTGTTATATGATCCTGAGCAAAATGAAGTGCACATTGGAACACTGAGTGATCCATCAAAGCCCATTATCTTCTCCATGGCAAGGCTTGACCGTGTGAAAAACATCACAGGGCTGGTAGAGTGCTATGCTAAAAATACCAAGCTGAGGGAACTGGTAAACCTTGTTGTGGTTGCGGGTTACAATGATGTGAAGAAGTCAAATGACAGAGAAGAAATTGATGAAATTGAAAAGATGCATTGCCTTATCAAAGAATACAACTTGGATGGCCAGTTTCGTTGGATATCAGCCCAAACAAATCGAGCACGCAATGGTGAGCTCTATCGCTACATAGCTGACAAGAGAGGTGCTTTCGTGCAGCCTGCATTTTATGAAGCCTTTGGGCTTACAGTTGTGGAGGCCATGACCTGTGGGCTTCCAACATTCGCCACTTGCCACGGTGGTCCGGCGGAGATTATTGAAGACGGAATATCAGGGTTTCATATCGATCCATATCACCCTGATAAGGTTTCTGCAAGTTTAGCAGATTTTTTCCAACGGTACACATGGAAGATCTACTCGGAAAGGTTAATGACGTTGTCTGGAGTTTATGGTTTCTGGAAGTATGTTTCAAAACTCGAGAGGCGTGAAACCCGGCGATATTTAGAGATGTTCTACATTATCAAGTACCGTGATTTGGTAAAGTCCGTGCCTCTGGCAATTGATGAGGAACACTAA

>AeSPS1

ATGGCGGGAAACGACTGGATAAACAGTTACCTGGAGGCGATACTGGATGTGGGGCCAGGGATCGACGACGCGAAATCGTCGTTGCTACTTAGAGAGAGAGGCAGGTTCAGTCCCACTCGCTACTTCGTCGAGCAGGTCATCGGCTTCGATGAGACCGATCTCTACCGCTCCTGGGTTAAGGCGGCGGCGACGAGAAGTCCCAGGGAGGCGGAATACGAGACTCGAGAAACATGTGCTGGCGGATTTGGAATTTGGCTCGCCAGAAAAAGCAGGTTTGGCGTGTTATTTGAAATGGTTGTCACCCGAATAAGTGGGGGACTTAAGATCCTTGATGTGGAATTCTGCTTTGGATATGTCTATACATGTGGATACTTGTATGAGCACCAGCGGATTACTGTGCTTGAGGGAGAGGAAGCTCAAAGGATGGCTAAACGTCGTCTTGAACGTGAAAGAGGCCGCAGAGAAGCAACTGCTGATATGTCTGAAGACTTATCTGAGGGGGAAAAAGGAGATACAGTCAGCGATCTGTCGGCTCATGGTGAAAGCAACAGGGCGACGAGGTATACCTATAGGGTTAAAGTCTCACTAAGCCTTCATGGTCTAATACGGGGTGAAAATATGGAGCTTGGTCGTGATTCTGATACTGGTGGCCAGGGCTTTGGGTTCAATGCCAGGAGTTTGTATCGGGTTGATTTACTCACTAGACAAGTATCATCACCAGAAGTAGACTGGAGTTATGGTGAACCCACTGAAATGTTGCCTCCAAGAAATTCTGATGGTTTGATGGATGAGATGGGGGAGAGTAGCGGTGCTTATATTATTCGTATTCCATTTGGCCCAAGAGATAAATATGTACCGAAAGAACTTCTGTGGCCACACATCCCTGAATTTGTTGATGGTGCTCTTAACCACATCATACAGATGTCCAAAGTACTTGGTGAGCAAATTGGCAGCGGGCATCCTGTGTGGCCTGTTGCTATCCATGGGCATTATGCAGATGCAGGTGATGCCGCTGCTCTTCTATCAGGTGCTCTAAATGTACCCATGCTTTTCACTGGTCACTCACTTGGTAGGGATAAGCTGGAACAGCTTTTGAGACAAAGTCGATTATCAAAGGATGAAATAAATAAGACATACAAAATAATGCGTCGTATAGAAGCTGAGGAGTTATCGCTTGATGCCTCTGAAATAGTGATAACTAGCACTAGACAGGAGATAGAACAGCAATGGCGTTTGTATGATGGTTTTGATCCGGTGCTAGAACGGAAACTACGAGCCAGGATCAGGCGTAATGTGAGCTGTTATGGAAGGTTCATGCCTCGCATGGTTGTAATGCCCCCTGGGATGGAATTTCATCACATTGTTCCACATGAAGGTGATATGGACGGTGAAACTGAAGGAAATGAAGATCAGCCTACTTCTCCAGACCCACCCATTTGGCCTGAGATAGTGCGCTTCTTTACTAATCCACGCAAGCCGATGATACTTGCCCTAGCTAGGCCAGATCCCAAAAAGAATCTCGCAACTTTGGTTGAAGCATTTGGGGAATGTCGTCCATTAAGAGAGCTGGCTAATCTGACTTTAATAATGGGTAACCGAGGTGATGTTGATGAAATGTCAAGCACTAATTCTTCTGTTCTTCTCTCAATACTTAAGCTTATTGATAAGTATGATCTCTATGGTCAAGTGGCATACCCCAAACACCACAAGCAGTCTGACGTTCCTGATATCTACCGTCTGGCAGCTAAGACAAAGGGCGTTTTCATTAATCCAGCTGTCATTGAGCCTTTTGGGCTTACTCTGATCGAGGCAGCAGCTTATGGGTTACCAATTGTCGCCACAAAAAATGGAGGTCCTGTTGACATACATCGGGCTCTTGACAATGGTCTCCTTGTGGACCCCCATGATCAGAAGTCTATTGCTGATGCTCTTTTAAAGCTGGTTGCGGATAAGCAACTTTGGTCCAAGTGCCGCCAGAATGGGTTGAAAAATATTTACCTTTTCTCATGGCCAGAACATTGTAAAACTTACCTATCTCGAATAGCAGCTTGCAAACTGAGGCAACCATGGTGGCAAAGAAGTGACGATGGGGATGAAAATTCTGAGTCGGATTCACCAAGTGACTCCTTGAGAGATATATCCTTGAACTTAAAGTTTTCACTGGATGGAGAAAAGAATGAAGGCAGCGGAAATGCCGATAGTTCTTTAGAATTTGAAGATCGCAAGAGTAAGTTGGAGAATGCTGTTTTGACATGGTCAAAGGGTTTCCAGAAGGGCACACAAAAGGTTGGGCTTACAGAGAAAGCAGATTCGAACATCACTGCTGGAAAGTTCCCAGTGTTGAGGAGGAGGAAGAATATTATTGTTATTGCCGTGGATTTTGGTGCTATATCAGATTTTTCCGAAAGTATTAGAAAGATATTTGACGCTGTGGAGAAGGAAAGGACTGAAGGCTCTATAGGATTCATATTAGCAACATCCTTTACTTTGTCCGAAGTCCATTCTTTTCTCATCTCTGGGGGACTGAGCCCTTCTGATTTTGATGCGTTTATCTGCAATAGTGGTAGTGATCTCTACTATTCATCTCTTAATTCAGAGGATAATCCCTTCGTTGTTGACTTATATTACCACTCACATATTGAATACCGCTGGGGTGGAGAAGGGTTGAGGAAGACTTTGATTCGTTGGACGGGTTCTATCAATGACAAGAAGGGCGAAAATGAAGAGCAGATTGTTACCGAAGATGAAAAGATTTCAACCAATTATTGTTATGCTTTCAAAGTGCGAAATGCAGGGAAGGTTCCTCCGGTCAAGGAAATCAGAAAATTAATGAGGATTCAGGCTCACCGTTGCCATGTTATTTATTGCCAGAATGGGAATAAGATAAATGTAATTCCAGTATTGGCGTCTCGTTGCCAAGCCCTGAGGTATCTCTATCTCCGGTGGGGCATGGACTTGTCAAAAATGGTGGTTTTTGTCGGAGAAAGCGGGGACACTGACTACGAGGGCTTGCTCGGTGGCATACACAAGTCTGTAATATTGAAGGGAGTTTGTAGCGGTCCGACCAATCAACTCCATGCCAACAGAACCTACCCTCTTTCTGATGTCCTGCCGATTGACAGCCCTAACATTGTCCAGGCAGCTGAGGAATGCAGCAGTGCCGATCTCCGGACCTCGTTGTTGAAGCTAGGGTTCATCAAGGGCTAG

>AeSPS2

ATGGCCGGGAACGAGTGGATAAATGGGTACTTGGAGGCGATTCTTGACAGCGGCGCGGCGGCGATTGAGGATCAGAGCAAGCCAATCCCCGTGAATCTTAGAGAGAGAGGGAATTTCAATCCCACCAAGTATTTTGTCGAAGAGGTTGTTACTGGGGTCGATGAGACCGATCTTCACAGAACATGGATCAAGGTGGTTGCGACTCGCAATACTCGCGAGAGGAGCTCCAGATTGGAGAACATGTGTTGGAGGATTTGGCATCTCACTCGCAAGAAGAAGCAGTTGGAATGGGAGGATTTCCAAAGGATGGCACACCGAAGATGGGAACGTGAACAAGGACGCAGGGATGCGACAGAAGACATGTCCGAAGACTTGTCTGAAGGAGAGAAGGGAGATGTACTTGGAGAAACGATGGTGAATGAAAGTCCGAGGAAAAAGTTCCAGCGTAACTTTTCCAATTTGGAAGTATGGTCGGATAATAACAAGGAGAAGAAGCTTTACATTGTTCTTATCAGTTTGCACGGTTTGGTCCGTGGGGAAAATATGGAGCTTGGGCGTGATTCCGATACTGGTGGTCAGATCAAATATGTGGTAGAACTTGCTCGGGCCCTGGCTAGGATGCCGGGGGTATATAGGGTCGATCTGTTCACCCGCCAAATATCTTCTCCCGAAGTCGATTGGAGCTATGGAGAGCCCACAGAGATGCTCACCGCAGGTGCTGATGACGATGCTGATGTTGGAGAAAGCAGCGGGGCTTACATTGTAAGGATACCCTTTGGTCCACGTGATAAGTACATGAGAAAAGAATTGTTGTGGCCCTATGTTCAAGAATTTGTAGATGGAGCTTTGGCTCACATTCTCAATATGTCAAAGGCTTTGGGTGAACAAATTGGTGGGGGGCAGCCTGTTTGGCCATACGTAATTCATGGCCACTATGCAGACGCGGGGGATAGTGCTGCTCTTCTTTCAGGTGCTTTGAATGTTCCTATGGTTTTAACAGGACATTCACTAGGTAGAAACAAGTTAGAACAGCTTCTCAAGCAAGGAAGGCAATCAAAAGAGGATATCAATTCTACGTATAAGATTATGAGGAGGATAGAGGCGGAAGAGCTTTCCCTTGATGTTGCAGAGCTTGTTATCACGAGCACCAAACAGGAGATTGACGAGCAATGGGGGCTTTATGACGGGTTTGATGTCAAGCTTGAGAAAGTTTTGCGGGCACGTGTTAGACGAGGGGTCAATTGCCATGGACGATACATGCCAAGGATGGCGGTTATTCCTCCCGGAATGGACTTCAGCAATGTTGTGGTTCAAGAGGATACCGCTGAAGCTGACGGGGAGCTGACAGCACTAACTACTGCAGACGGGTCTTCTCCAAAAGCTTTCCCACCAATATGGTCAGAAATGATGCGTTTTCTAACAAATCCCCATAAACCAATGATCTTGGCCTTATCAAGACCTGACCCGAAAAAGAATATCACCACTCTTTTGAAAGCCTTTGGAGAATGCCGCCCGTTGAGAGAACTTGCTAATCTGACGCTTATAATGGGAAATAGGGACGATATTGATGAGATGTCCGCTGGGAACGCTAGTGTCCTCATCACAGTTCTGAAACTGGTTGACAAATATGATCTGTACGGGCAAGTGGCCTTCCCAAAGCATCACAAGCAAAGTGATGTTCCGGAAATCTACCGCCTTGCTGGGAAAACTAAGGGGGTCTTCATAAATCCAGCTTTGGTTGAGCCTTTTGGGCTTACCCTAATTGAGGCTGCAGCACATGGACTTCCAATGGTGGCAACTAAAAATGGTGGTCCAGTTGACATTCATCGGGCTTTGAATAATGGTCTGCTTGTCGACCCACATGATCAGGAAGCAATTGCTAATGCGCTGCTTAAACTAGTGTCAGAGAAGAATTTATGGCATGATTGCAGAAAGAATGGTTGGAAGAACATACACCTATTTTCATGGCCCGAACACTGCCGCACATACTTGACAAGGGTGGCAGCATGTAGAATGCGACACCCTCAGTGGCAAACTGACACCCCGGCAGATGAATTTGCCGCTGAAGAGTCCCTAAATGACTCACTCAAGGACGTGCAAGACATGTCCCTGAGGCTGTCAGTTGATGGGGAAAGGACATCACTTAATGAATCGCTTGACCATGTAACTGCAACTGGTGGTGGCCCTGAGCTCCAAGACCAAGTGAAACAGGTAGTAAGCAGGATGAGGAAACCAGAAACAAAAGCTCAAGATTCCGAAGGCAGTGGGAAACTTGTTGATAATATTGCAAGCAAATATCCGATGTTGAGAAGGAGGCGCCGGTTGATTGTCATAGCACTTGATTGCTACGACAGTGATGGAGCCCCAGAGAAAAAGATGATTCGGATAATTCAAGAGATATTTAGGGCTGTTAATGTAGTCTCTCAAACTGCTAGGTTCTCGGGATTTGCTCTATCGACAGCTATGTCAATGTCGGAGCTGAAAGCCTTCTTGAAAACTGGGAATATTCAAGTAAATGAGTTTGATGCTTTGATTTGTAGCAGTGGGAGTGAAGTCTACTACCCGGGTACTTATACACAAGAAGACGGGAAGCTTTATCCCGACCCAGACTATGCCACACATATTGACTACCGTTGGGGTTGTGATGGCTTGAAAAAGACTATTTGGAAGTTGATGAATTCACATGAAGGCGGGTCTTCTCATTCTAAAAGCCCAATTGAAGAAGATGTGAAATCTAGTAATTCGCATTGCGTCTCCTACTTGATAAAGGATCTCAGTAAGGCAAAGAAAGTGGATGATATGAGGCAGAAGCTGAGGATGCGGGGTCTCCGTTGCCATCTGATGTACTGCAGAAACTCAACAAGAATGCAAGTTGTCCCTCTTCTTGCATCTCGAGCACAAGCACTGAGGTACCTTTTTGTTCGTTGGAGACTAAACGTATCTAACATGTATGTGATTCTCGGTGAAACTGGAGACACTGATTATGAGGAACTGATATCTGGCACCCATAAGACGCTAATCATGAAAAACATGGTGGAGAAAGGTTCCGAAGAGCTGCTCAGAGCAGCAGGAAGCTATCTAAAAGATGACATTGTCCCCGAGGAGAGCCCACTGGTGACCTACACAAGTGGAGAAGCGAAAGCCGATGACATTGCAAATGCTTTGAAGCAAATCTCTAAATCTAGCCCGGGAATATGA

>AeSPS3

ATGGCGAATAACGAGTGGATAAACGGGTACTTGGAGGCGATTTTGGACGCGGGGAGTCGGAGAAGCGGATTGAGAGAGAATGGCTATGCTGATGAAGGAATTAGAAGCAACAATAGCATGAACAACATGAGTATAAGGAAAAGGTTTGAAGACAAGTTGAGAATTGAAAAGTTCGATGATGACAAAGGAAAGGAAGAGAAGCTGTTTAGTCCCACCAAGTATTTTGTTGAGGAAGTTGTTAATTGCTTTGATGAGTCTGACCTCCACAGGACATGGATCAAGGTAATAGCAACAAGGAATACTCGTGAACGCAGTAACAGGCTCGAGAATATGTGCTGGCGAATTTGGCATCTCGCACGTAAAAAGAAACAGGCTGCAAAAAGACGAATCGAGCGTGAGAAAGGTCGGAATGATGCTGCAGAAGACCTGTCTGAGCTTTCCGAAGGGGAGAAGGAAAAGGGTGATGCCAATCAGACAGAGCCTATATCAGAAAAGATTTCCAGAATTAACTCTGACATGAAAATATGGTCGGATGATGATAAATCTAGGCGCCTCTACATTGTCCTAATCAGTCTACATGGATTGGTGCGTGGGGAAAATATGGAACTCGGAAGAGATTCAGACACTGGTGGTCAGGTGAAGTATGTAGTAGAGCTTGCCCGAGCCCTGGCCAACATGAAAGGAATCTATCGTGTTGACCTTCTGACTCGACAAATCACCTCGTCGGAGATTGACTTTAGCTACGGTGAGCCCAATGAGATGCTCTCGTGCCCATCCGATGGCAGTGGTAGCTGTGGTGCATACATTATTCGGATCCCATGTGGACCTCGCGACAAGTACATTCCAAAAGAGTCACTCTGGCCTCACATACCAGAATTTGTAGATGGGGCCCTAAGCCACATCGTGAACATGGCAAGAGCTATAGGAGAACAAGTGGATGGGGGAAAGCCGACGTGGCCTTACGTAATTCACGGTCACTATGCTGACGCAGGGGAGGTAGCAGCACGCCTATCCGGGGCATTGAATGTGCCAATGGTGCTAACGGGGCACTCCTTGGGAAGAAACAAATTTGAGCAATTACTTAAACAAGGGAGGTTGTCTAGGGAGGATATAAATTCAGCGTACAAGATAATGAGGAGGATTGAGGCTGAAGAGTTGGGGTTGGATGCTGCTGAAATGGTGGTAACTAGCACGAGGCAAGAGATCGAAGAGCAATGGGGTTTATACGATGGGTTTGATATCAAGTTGGAGAGGAAGCTTAGGGTTAGAAAACGGCGTGGAGTGAGTTGCCTTGGTCGATACATGCCAAGGATGGTGGTTACACCACCGGGGATGGACTTCAGCTATGTCACAACACAAGATTCATTGGAAGGTGACGGAGATCTAAAGTCGTTGATTGGCTCTGATAGAACTCAAAACAAAAGGCACATACCTCCAATATGGTCCGAGGTAATGCGATTTTTCACAAACCCTCACAAGCCTATGATCCTAGCATTGTCACGTCCAGACCCCAAGAAAAATGTGACCACTTTGCTCAAGGCTTTTGGAGAATGCCGACCACTCAAAGAATTAGCCAACTTGACCCTAATACTTGGTAACAGAGACGACATAGAAGATATGTCTAACAGCAGTTCAGTAGTTCTCACAACAGTGCTTAAACTCATTGACAAGTACGACTTGTACGGTCAGGTAGCATATCCCAAACATCACAAACAATCCGAAGTTCCTGAGATCTATCGACTGGCTGCAAAAACAAAGGGAGTTTTCATCAACCCAGCCCTGGTTGAACCATTTGGTCTCACACTCATTGAGGCGGCTGCTTATGGTTTACCAATTGTTGCCACGAAAAATGGTGGCCCTGTGGATATTCTCAAGGCACTCAACAATGGCCTTCTCATTGATCCACATGACCAGAAAGCCATAGCAGATGCCCTCCTAAAGCTTGTAGCTGAAAAGCATCTTTGGCTTGAGTGCCGCAAAAATGGCCTAAAGTATATTCATCGTTTCTCGTGGCCAGAACATTGTCGTAACTATCTTTCCCATGTCGAGCATTGCAGGAACCGCCATCCTACAACCCGTCTTGAGATCATGCCAACTCCTGAAGAACCCATGAGCGAATCTTTAAGGGATGTGGAAGACATTTCTTTAAAATTCTCCATTGATGCAGACTTCAAGCTCAATGGAGACCTTGATGTACCAAATAGACAATGCAAACTCATTGAGGCCTTAACACAGATGGGTTCCTCCAATAGCCCTTCCAGCACTAGTTACTGTCCAGGAAGAAGGCAGGCACTATTTATAATTGCTACTGATTGTTATAACAGGGGTGGAATGTGCACCGAGACCTTTCCATTGGTCATCAAGAATGTGATGCAAGCTGCAGTCTCAAACTCAGGCAAGATAGGCTTTATATTGTCAACAGGTTTAACTTTACTTGAGACAAAGGAAATGTTAAGACATTGCCATGTAAATTTGGAAGATTTCGACGCATTTGTATGCAATAGTGGAAGTGAAATGTACTATCCATGGAGAGACTCCACAGCTGATATGGATTATGAAGCCCATATTGAATACAGGTGGCCCGGCGAGAATGTGAGATCAATGATAATGAGGCTTGGTCGGGTAGGAGATGGAGACGGAGATGGAGATGATATTATGGAAAGTACAGATGCATTTAGCTCCCGATGCTATTCTTATAGCATTAAACAAGGAAGCAAGACTCGAAGGATCGATGAACTGCGCCAGAGGCTACGCATGAGAGGTTTACGGTGCAATGTTATCTACACTCGTACTTCATCACGGTTGAAGGTAGTACCTTTATTTGCATCAAGAGCTCAAGCACTAAGGTATCTATCTGTAAGGTGGGCTATTGATCTTTCCAAAATGGTAGTGTTTGTCGGAGAAAGAGGGGATACAGACTATGAAGACCTGTTGGTTGGCCTACACAAGACTGTTATTCTAAGAAATTCTGTAGAATATGGCAGTGAGATGCTTCTGCGCAGTGAAGAAAGTTTTAAACGCGAAGATGTGGTCCCCCAAGATAGCCCTAGGATTGCCTTTGGTGCATGTTATGAAACCCATGATATCTCTGCAGCTTTAGATGCTCTACAGGTCATATGA

>AeSUS1

ATGGCAGGACAAGTCCTGACTCGTGTTCACAGCCTTCGCGAGCGTCTTGATGGAACTCTGTCTGCTCATCGCAATGAAATATTGCTGTTTCTCTCCAAGATTGAAAGCCATGGCAAAGGAATTCTGAAACCCCATCAGATTGAGGCTGAGATCGAAGCACTCTCCAAAGAGGTCCAACAGAAACTGTATGATGGAGCATTTGGAGAGCTTCTCAAATCAGCACAGGAAGCAATTGTTTTGCCTCCATGGATTGCTTTTGCGGTTCGGCTAAGGCCCGGTGTGTGGGAATACATGAGGGTGAATCTCAATGCCCTTGTTGTTGAAGAATTGAGTGTTCCCGAGTATCTGCAGTTCAAGGAAGAACTTGTGGATGGACCGTGCAATGGAAACTTTATTCTTGAGTTGGATTTTGAGCCCTTCACTGCATCATTTCCTCGGCCAACTCTTTCAAAATCAATTGGGAATGGAGTTGAGTTCCTTAACAGACACCTCTCTGCTAAAATGTTCCATGACAAGGAAAGCATGCACCCTCTCCTTGATTTTCTTAAAGTCCACAACTACAATGGCAAGACAATGATGCTGAATGACAGAATACAAAACCTCAATGCTCTCCAATTTGTGCTGAGGAAGGCTGAGGAATATCTCCTTACACTCCCTCTAGAGACACCCTATTCCGAGTTTGAGCACAAGTTCCAAGAGATCGGTTTGGAGAGAGGGTGGGGTGACACTGCTGAGAGAGTGCTTGAAATGATTCATATGCTGTTGGAGCTTCTTGAGGCCCCAGATCCATGCACTCTTGAGAAATTTCTTGGCAGAATCCCAATGGTTTTCAATGTTGTGATCCTTTCTCCCCATGGCTACTTTGCCCAAGAAAATGTTTTGGGCTATCCTGACACTGGTGGTCAGGTGGTTTACATTTTGGATCAAGTTCCTGCCATGGAGAAGGAAATGCTTAAGCGCATCAAGCAGCAAGGGCTTGATATATCCTCGCATTCCATTGTCGTAACTAGGCTTCTCCCAGATGCGGTAGGCACTACCTGCAATCAGCGCATTGAGAAGGTTTACGGAGCAGAACATTCGCATATACTTCGAGTTCCCTTTAGGACTGAGGAGGGAATTGTCCGCAAATGGATCTCACGTTTTGAAGTCTGGCCTTACATGGAGAGATTCACCGAGGATGTTGCACATGATATCGTCACAGAGTTGCAGGCAAAGCCTGACTTGATCATTGGTAACTATAGCGAGGGTAACCTTGTTGCCTCATTGTTAGCTCACAAATTGGGGGTAACACAGTGTACCATTGCTCATGCCTTGGAGAAAACAAAATATCCAGATTCCGACATCTATTTGAAGAAATTTGACGAGAAGTACCACTTCTCGTGCCAGTTCACAGCAGATCTCATTGCTATGAATCATACCGATTTTATAATCACCAGCACTTTCCAGGAAATTGCTGGAAGGTATTACTTTCTTGCTGTGGTGTTGTTTATAATGCTAATATGCTACACACATTGTGTTCTCCGCAAGAACACTGTTGGACAGTATGAGAGTCATATGGCCTTCACTATGCCCGGACTTTACCGAGTTGTCCATGGTATTGATGTGTTTGACCCCAAATTCAACATTGTCTCACCGGGGGCCGATATGAACATCTACTTCCCTCACACTGAGAAGGACAAGAGACTGACCAAGTTCCACCCTGAAATCGAAGATCTTCTCTTTAGTGATGTGGAGAATAAAGAGCATATAGGTGTGTTGAAAGACCGTACCAAGCCAATCATATTCTCCATGGCAAGGTTGGACCGTGTGAAGAACTTGACCGGGCTTGTCGAGTTGTACGGCAAGAATGCCAGACTTAGAGAGCTGGCCAACCTTGTGGTGGTGGGTGGAGATCGTAGGAAGGAGTCCAAGGATTTGGAAGAGCAAGCCGAGATGAAGAAGATGTACGATCTCATTGAAACGTACAAGTTGAACGGTCAGTTTAGATGGATTTCTTCCCAGATGAACCGGGTGAGGAATGGGGAACTTTACCGCTTCATTGCTGACACCAAGGGTGTGTTTGTTCAACCCGCCTTCTATGAGGCTTTTGGGTTGACAGTGGTGGAGGCGATGACCTGTGGTTTGCCAACATTTGCAACTTGCCATGGTGGTCCAGCTGAGATAATTATTCACGGCAAGTCTGGCTTCCACATTGATCCTTATCATGGTGATCAGGTCGCTGAACTCCTTGTCAATTTCTACGAGAAGTGTAAAGTTGATCCTTCTCATTGGGACGCTATTTCCGAAGGAGGTCTGAAGCGCATCCTGGAGAAGTACACATGGCAGATTTACTCTGAGAGGCTAATGACTCTTGCTGGGGTTTACGGATTTTGGAAGTACGTCTCCAAGCTTGATCGCCGTGAGACTCGCCGCTACCTTGAGATGTTTTATGCTCTCAAGTACCGCAAGTTGGCTGAGGCAGTTCCTTTGGCTGTTGATCAGTAG

>AeSUS2

ATGCTATTGGAGCTTCTTGAGGCTCCAGATCCATGCACTCTTGAGAAATTCCTTGGCAGAATCCCAATGGTTTTCAATGTTGTGATCCTTTCTCCCCATGGCTACTTTGCCCAAGAAAATGTTTTGGGCTATCCTGACACTGGTGGCCAGTTCTGCCCTGGAGAGGAAATGCTTAAGCGCATCAAGCAGCAAGGCCTTGATATCATTCCACGCATTCTCATTGTTAGTGTTACTATATGTACTAGGGCGTTCTTCTCCCCAGATGCAGTAGGCACTACCTGCATCAGCGCATTGATAAGTTTATGGAGCAGAACATTCGCATATACTTTTCGAGTTACTTTAGACTGGAAGGGAATTGTCCGCCAATGGATCTCGCGTTTTGAAGTCTGGCCTTACATGGAGAGATTCACCGAGGATGTTGCACATGACATCGTCACAGAGTTGCAGGCAAAGCCTGATTTGGTCATTGGCAACTACAGTGAGGGCAACCTTGTTGCATCATTGTTAGCTCACAAATTAGGGGTAACTCAGTGTACCATTGCTCACGCCTTGGAGAAAACAAAATATCCTGATTCCGACATCTATTTGAAGAAATTTGACGACAAGTACCACTTTTCGTGCCAGTTCACAGCAGATCTCATTGCTATGAATCACACCGATTTTATAATCACAAGCACTTTCCAGGAAATTGCTGGAAGCAAGAATACCGTTGGACAGTATGAGAGTCATATGGCCTTCACTATGCCTGGACTCTATCGAGTTGTCCATGGTATTGATGTGTTTGACCCCAAATTCAACATTGTCTCACCAGGGGCCGATATGAACATCTACTTCCCTCACACTGAGAAGGACAAGAGACTGACCAAGTTCCACCCTGAAATCGAAGATCTTCTATTTAGTGATGTGGAGAATAAAGAGCATATTGGGGTGTTGAAAGACCGTACCAAGCCAATCATATTCTCCATGGCAAGGTTGGACCGTGTGAAGAACTTGACCGGACTTGTCGAGTTGTACGGTAAGAATGCCAGACTTAGAGAGCTGGCCAACCTTGTGGTGGTGGGTGGAGATCGTAGGAAGGAGTCCAAGGATTTGGAAGAGCAAGCCGAGATGAAGAAGATGTACGATCTCATTGAAACCTACAAGTTGAATGGTCAGTTTAGATGGATATCTTCTCAGATGAACCGGGTGAGGAATGGGGAACTTTACCGCTGCATTGCTGACACAAAGGGTGTATTTGTTCAACCTGCCTTCTATGAGGCTTTTGGGTTGACGGTGGTGGAGTCGATGACCTGTGGTTTGCCAACATTTGCGACTTGCCACGGTGGTCCGGCTGAGATAATTATTCATGGCAAGTCTGGCTTCCACATTGATCCTTATCATGGTGATCAAGTGGCTGAACTCCTTGTCAATTTTTACGAGAAGTGCAAAATTGATCCTTCTCATTGGGACGCTATTTCTGAAGGAGGTCTGAAGCGCATCCTGGAGAAATACACATGGCAGATTTACTCTGAGAGGCTAATGACTCTTGCTGGGGTTTACGGCTTTTGGAAGTACGTGTCTAAGCTTGATCGCCGAGAGACACGCCGTTACCTGGAGATGTTATGCACTCAAGTACCGCAAGTTGGTAAGCTGAGGCAGTTCCTTTGGCTGTTGATCAGTAGAGGGATGATTGGCAAGTAA

>AeSUS3

ATGGCAGCCTTGAAGAGGTCTGAGTCGATGGCTGATAGCATGCCGGACGCCCTGAGAGAGAGCCGGTACCACATGAAGAAGTGCTTTGCTAAGTACATTGAGCAAGGAAAGAGATTGATGAAACTTCGACACTTAATGAGCGAAATGGAGAAAGTGATCGATGATAAGACTGAGAGAGAGCAGTTCTTGAACAGCCTACTCGGCTACATTTTGTGCACCACTCAGGAGGCGGTTGTTATTCCTCCATATGTTGCCTTTGCCATTAGACCAAATCCTGGGTTCTGGGAATTCATTAAGGTGAGCTCTACCGATCTATCGGTAGAGGGCATCACTGCCACGGACTACTTGAAATACAAAGAAATGTTGGTTGATGAGGACTGGGCAAAGGATGAAAATGCATTGGAAGTTGATTTTGGAGCGATGGACTTTTCCGCGCCTAGCCTGACCGTGTCTTCTTCGATTGGAAATGGAATCAATTTCGTTTCCAAATTCCTTTCTTCTAAACTACATGGTGGCTCACAGAAGGCTCAGCCTCTTGTTGATTACTTACTCTCACTAAATTACCATGAAGAAAAACTAATGATTAACGAGACCATCAACACCGCTGCAAAGCTTCAGAGCGCGCTAATAGTAGCTGAAGCGGCCCTTTTGACACTGCCCAAGGACACACCATACCAGGACTTTGAGCAAAGGTTTAGGCAGTGGGGATTTGAGAAGGGATGGGGCGATACTGCGGAAAGAGTGAGGGAGACAATGAGATCGCTTTCAGAGATATTCCAGGCACCGGACCCGTTAAATATGGAGAAGTTCTTTGGCAGGGTTCCAACGATTTTCAAAGTCGTTTTATTCTCGGTCCATGGGTATTTTGGACAATCTGATGTCCTCGGTTTGCCAGACACCGGTGGGCAGGTGGTCTATGTTTTAGATCAAGTAGTTGCTTTTGAAGAAGAACTGCTTGTTCGGATTAAGCAGCAAGGGCTTAATGTGAAGCCTCAAATTCTTGTGGTTACGCGACTCATCCCCGATGCCAAGGGGACTAAGTGCAACCAGGCGTTGGAACCGGTCGCCAACACCAAGCACTCTAACATCCTTCGGGTTCCATTTAGGACAGAAAATGGAGATCTTCCGCAATGGGACGCTACGGACAAAATCTTGGAAATCATGGAAGGGAAACCGGATCTAGTCATTGGAAACTACACAGATGGGAATCTGGTGGCATCACTCATGGCTAGCAAACTTGGCATAACTCTGGGAACTATTGCACATGCTTTGGAGAAGACAAAGTATGAGGATTCAGACCTAAAATGGAAAGAATTGGACCCCAAGTATCACTTCTCCTGCCAATTCACCGCTGACACGATCGCAATGAATTCTTCAGATTTCATCATCACTAGCACATACCAAGAAATTGCTGGAAGCAAGGATAGGCCGGGACAGTACGAAAGTCATGCTGCATTTACGCTTCCAGGGCTTTGCAGAGTTGTTTCAGGCATAAATGTGTTTGATCCCAAATTCAATATAGCTGCTCCCGGGGCTGACCAATCCGTCTATTTCCCTTACACCGATAGACCGAAGCGATTCACAAAGTTTCATCCTTCCATAGAAGAACTACTCTTTAGCAAAGTTGATAACATTGAGCACATGCTTGATATAGTGAAGAACATCAGCGGATTAACCGAGTGGTACGGGAAGAACAAGAGGCTTAGAAGTTTGGTTAATCTCGTTGTTGTCGCGGGGTTCTTCGACCCTACTAAATCCAAAGACCGAGAAGAGGCAGCCGAAATAAAGAAGATGCACATGTTGATCGAGAAATACCAACTTAAGGGTCAGATTCGATGGATAGCAGCACAGACCGACAGGCAACGAAACGGAGAGCTGTACCGTTGCATTGCTGACACGAAAGGAGCGTTCGCGCAGCCTGCACTTTATGAGGCGTTTGGCCTCACGGTTATTGAGGCGATGAACTGTGGATTGCCTACTTTTGCAACCAACCAAGGAGGCCCCGCGGAGATCATTGTTGATGGGCTTTCAGGGTTCCATATTGATCCTAATAATGGGGATGAGTCAGGGAACAAGATTGCTGATTTTTTCCAGAAGTGCAAGGACGATCCCGAGCACTGGAACAAGATTTCCAAGTTGGGTTTGAACCGTATCTATGAATGCTATACATGGAAGATTTATGCAAACAAGGTGTTGAACATGGGGTGTGTGTATAGTTTTTGGAGGCAGTTGAACAAGGACCAGAAGCACGCAAAGCAAAGATACATCCAAATGTTTTATAATCTCCAATTCAGGAACTTGCATCAAGCGCACACAAAGTCGGTTTCAGAGGTTGTTCGGATCGTGAAGACTCAAACACAAGACAGCTTGATTCAGTGCCTCCCTGTTGTTATTCTTTCTTTTTTTCTTAAAAAAAAATAA

>AeSUS4

ATGGCAGCCTTGAAGAGGTCTGAGTCGATAGCTGATAGCATGCCGGATGCCTTGAGAGAGAGCCGGTACCACATGAAGAAGTGCTTTGCTAAGTACATTGAGAAGGGGAAGAGGTTGATGAAACTTCACCACTTAATGAGCGAAATGGAGAAAGAGGCAGTTGTTATTCCTCCATATGTTGCCTTCGCAATTAGACCAAATCCCGGGTTCTGGGAATTCGTTAAAGTGAGCTCTACAGATCTATCAGTAGAGGGCATCACCGCCACGGACTACTTGAAATCCAAAGAAATGCTGGTTGATGAGGACTGGGCAAAGGATGAAAATGCTTTAGAAGTTGATTTTGGCGCGATGGACTTTTCCGCGCCTAACCTGACCATGTCTTCTTCGATTGGGAACGGAATCAATTTCATTTCCAAATTCCTTTCTTCTATACTATATGGTGGCTCACAGAAGGCTCAGCCTCTTGTTGATTACCTACTCTCACTAAATCACCATGAAGAAAAACTAATGATTAACGAGACCCTCAACACCGCTGCCAAGCTTCAGAGCGCGCTAATAGTAGCTGAAGCGGCCCTTTTGACACTGCCCAAGGACACACCATACCAGGACTTTGAGCAAAGGTTTAGGCAGTGGGGTTTTGAGAAGGGATGGGGCGATACTGCAGAAAGAGTGAGGGAGACAATGAGATCGCTTTCAGAGATATTCCAGGCACCGGACCCGTTAAATATGGACAAGTTCTTTGGCAGGGTTCCAACTGTTTTCAATGTCGTTTTGTTCTCGGTCCATGGGTATTTTGGTCAATCTGATGTCCTTGGTTTGCCAGATACCGGTGGGCAGGTGGTCTATGTTTTGGATCAAGTAGTTGCTTTTGAAGAAGAACTGCTCATTCGGATTAAGCAGCAAGGGCTTAATGTGAAGCCTCAAATTCTTGTGGTCACTCGACTTATCCCTGATGCCAAGGGGACTAAGTGCAACCAGGTGCTAGAACCGATCGCCAACACAAAGCATTCCAACATTCTTCGCGTGCCATTTAGGACGGAAGATGGAGTTCTTCCGCAATGGGTTTCTCGTTTCGACATCTATCCCTACCTCGAAAGTTCTGTTGTCAATCAACAGGACGCTACAGATAAAATCTTGGAAGTCATGGAAGGGAAACCGGATCTCATCATCGGAAACTACACAGATGGGAATTTGGTGGCATCACTCATGGCTAGCAAACTTGGGATTACTCTGGGAACTATTGCACATGCTTTGGAGAAGACAAAGTATGAGGATTCAGACCTAAAATGGAAACAACTAGACCCCAAGTATCATTTCTCATGCCAATTCACCGCCGACACGATCGCAATGAATTCTGCAGATTTCATCATCACCAGCACATACCAAGAAATTGCTGGAAGCAAAGATAGGCCGGGGCAGTATGAAAGCCACGCTGCATTTACACTTCCAGGGCTTTGCAGAGTTGTTTCAGGCATAAATGTGTTTGATCCCAAATTCAATATAGCTGCTCCCGGGGCAGACCAATCCGTCTATTTCCCTTACACAGATAGACAGAAGCGATTCACTTCGTTTCGTCCTGCCATAGAAGAACTACTCTTTAGCAAAGTTGATAACAATGAGCACATGCTTGATATAGTGAAGAACATCAGTGGATTAACGGAGTGGTACGGGAAGAACAAGAGGCTGAGAAGTTTGGCTAATCTCGTTGTTGTCGCAGGGTTCTTCGATCCTACTAAATCCAAAGACAGAGAGGCAGCCCGAAAAATAAACAAAAATGCACATGGTGTGATTGAGAATTACAAATGCAAGGCACAGACTGACAGGCAACGAAACGGGGAGCTGTACCGTTGCATTGCTGACACAAAAGGAGCATTTGTGCAGCCTGCACTTTACGAGGCGTTTGGCCTCACGGTTATTGAGGCAATGAACTGTGGATTACCTACTTTTGCAACGAATCAGGGAGGCCCCGCAGAGATCATTGTTGATGGGGTTTCGGGGTTCCATATTGATCCGAATAATGGGGATGAGTCGGGGAACAAGATTGCTGATTTTTTCCAAAAGTGCAAGGACGATCCCGATCACTGGGACAGGATCCAATGTGGCCTATACAGGAAGATTATGCAACAGGTGTTGAACATGGGGTGTGTATATAGTTTTTGGAGGCATTTGAAAAAGGACCAGAAGCAAGCAAAGCAAAGATACATCCAAATGTTTTATAATCTCCAATTCAGGAACTTGGTGAAGAATGTCCCCACTTCAAGGGTTGAACCTCAACAACAGCCTAAGGAAAAACAACCCAAAGCACAGCCCTCTCAAAACGTCAAGCGCACACAAAGTCGGTTTCAGAGGTTGTTCGGATCTTGA

>AeSUS5

ATGGCTTCCGCAAAAGTTCTTAGGAAGTCGGACAGTGCAATAGCCGAGAGCTTGTCGGATGCTCTAAAGCAGAGCCGGTACCACACGAAGAGATGCTTTGCTAGGTTTGTTGAAACGGGGAAGAGGTTGATGAAACCCCGTCATTTAATTGAGGAAATGGAGAAGGTGATTGGGGACAAGAGCGAAAGAGCCAAAGTTTTGGAGGGTTTACTCGGTCTCATCATCAGTTCCACTCAGGAGGCAGCTGTGGTTCCACCAAATGTTGCTTTGGCAGTGAGGAGGAGCCCCGGTTTCTGGGAGTTTTTCAAGGTGAATGTTGATGATCTAACCGTGGATGCTATTTCGGCTAAAGACTACTTGAAGCTCAAAGAAACAATCTGCGACGAGAATTGGGCAAAGGATGAAAATGCATTGGAATTGGATTTTGGTGCATTTGATTTCTCCAGTCGTCGCCTAACCCTTTCTTCTTCGATTGGAAATGGGGTCGATTTCATCTCAAAGTTCATGGCTTCAAAGACTAGTGGGGATCTTGAGCATTCGAAGCCTTTGCTCGAGTACTTGCTGGCGCTTAATCATCACGGGGAGAATCTAATGATCAATGAGACTCTCAACACATTTCCCAAGCTTCAAGAAGCGTTGATTGTAGCTGATGTTTACCTTTCTGCTCTCCCAAAAGACACACCCTACCAGAACTTTGAGAAAAAGCTTAAAGATTGGGGCTTTGAGAAAGGGTGGGGAGATAATGCAGAAAGAGTTAGAGATACAATGACAATCCTTTCGGAGATATTCCAAGCACCGGACCCGACTAAAATGGAGTCTTTCTTTAGAAGACTTCCAAATATATTCAATATCGTGATCTTCTCGGTCCACGGTTACTTTGGCCAAGCTGACGTCCTCGGTTTGCCTGATACTGGAGGGCAGGTGGTTTACATTTTAGATCAAGTGAAAGCTTTGGAGGAAGAACTACTACTCAGAATTAAGCAGCAAGGATTGAGTGTGAAGCCTCAGATTCTTGTGGTAACTCGTCTCATACCAGATGCACAAGGAACAAAGTGCAACCAGGAAATCGAGCCAGTCCTCAACACGGCACACTCCCACATTATTAGAGTCCCATTCATGACCGACAAAGGGGTTCTCCGCCAATGGGATGCTACTGCTAAGGTCCTTGGGCACTTGGAATGTAAACCAGACCTTATACTTGGGAACTATACTGATGGAAACTTGGTGGCCTCTCTAATGGCTAACAAACTTGGAGGAACCATTGCTCATGCTTTAGAGAAGACTAAGTATGAAGATTCTGACATCAAATGGAAGGAGTTGGATCCAAAGTACCACTTCTCATGCCAATTCACAGCTGACATTATTGCGATGAATTCGGCCGATTTCATAATTACCAGCACATATCAAGAAATCGCAGGAAGCAAGAATAGGCCTGGACAATATGAAAGCCATATGGCATTTACCATGCCGGGCCTTAGCAGAGTAGTTTCAGGCATCAATGTCTTTGACCCAAAGTTCAATATCGCTGCTCCCGGGGCTGAACAAGAAAAACCAATCATCTTCTCGATGGCAAGGCTCGATACGGTGAAGAACATTTCCGGATTGACCGAGTGGTACGGGAAGAACAAAAGGCTCAGGAACTTGGCAAATCTTGTTGTTGTTGCGGGATTCTTCGATCCATCCAAATCAAAAGATAGGGAAGAAATTGCAGAAATCAACAAGATGCACGCTTTGATACAGAAGTACCAACTCAAGGGTCAGATCAGATGGATAGCAGCGCAAACCGACAGGTACCGCAACGGAGAGTTGTACCGATGCATTGCTGATACGAAGGGGGCTTTTGTGCAGCCGGCACTGTATGAAGCTTTTGGTCTGACAGTTATCGAAGCAATGAACTGTGGATTACCCACATTTGCAACCAACCAAGGTGGACCAGCGGAAATCATAGTCGATGGGGTTTCGGGTTTCCATGTTGATCCGAACAATGGCGATGAATCGAGCAACAAGATAGCCGATTTCTTTGATAAGTGCAAGGGGGATGCTGAGTATTGGAATAGGATGTCTAAAGCAGGTCTCCAGCGCATATACGAATGCTACACATGGAAGATCTATGCAAACAAAGTCTTGAACATGGGGTCTTTATATGGCTTTTGGAAGCAGTTGAACATTGAACAGAAGAAAGCTAAGCAAAGATACCTTCAAATGTTTTATACTCTCCAATTCAGGAATCTGTTCATATGTCACCTTGATTTCAAACATGCTTCAACTCGTATGATAATTGTGCTCAGGGAAAACGGGTTAGAGCAGCAGAAGCAGCTTGCCTCACCGAGAGATGCTCACACTTCCTGCCCCTGCTCTTCGTGGTGTTTCCTGTTTCTCTCTGTTTCCATCATTATCTACGCTGCTATGAAGTACTATGGCTTCTTCAGACAACCATGA

>AeSUS6

ATGAAACCCCGTCATTTAATTGAGGAAATGGAGAAGGTGATTGGGGACAAGAGCGAAAGAGCCAAAGTTTTGGAGGGTTTACTCGGTCTCATCATCAGTTCCACTCAGGAGGCAGCTGTGGTTCCACCAAATGTTGCTTTGGCAGTGAGGAGGAGCCCCGGTTTCTGGGAGTTTTTCAAGGTGAATGTTGATGATCTAACCGTGGATGCTATTTCGGCTAAAGACTACTTGAAGCTCAAAGAAACAATCTGCGACGAGAATTGGGCAAAGGATGAAAATGCATTGGAATTGGATTTTGGTGCATTTGATTTCTCCAGTCGTCGCCTAACCCTTTCTTCTTCGATTGGAAATGGGGTCGATTTCATCTCAAAGTTCATGGCTTCAAAGACTAGTGGGGATCTTGAGCATTCGAAGCCTTTGCTCGAGTACTTGCTGGCGCTTAATCATCACGGGGAGAATCTAATGATCAATGAGACTCTCAACACATTTCCCAAGCTTCAAGAAGCGTTGATTGTAGCTGATGTTTACCTTTCTGCTCTCCCAAAAGACACACCCTACCAGAACTTTGAGAAAAAGCTTAAAGATTGGGGCTTTGAGAAAGGGTGGGGAGATAATGCAGAAAGAGTTAGAGATACAATGACAATCCTTTCGGAGATATTCCAAGCACCGGACCCGACTAAAATGGAGTCTTTCTTTAGAAGACTTCCAAATATATTCAATATCGTGATCTTCTCGGTCCACGGTTACTTTGGCCAAGCTGACGTCCTCGGTTTGCCTGATACTGGAGGGCAGGTGGTTTACATTTTAGATCAAGTGAAAGCTTTGGAGGAAGAACTACTACTCAGAATTAAGCAGCAAGGATTGAGTGTGAAGCCTCAGATTCTTGTGGTAACTCGTCTCATACCAGATGCACAAGGAACAAAGTGCAACCAGGAAATCGAGCCAGTCCTCAACACGGCACACTCCCACATTATTAGAGTCCCATTCATGACCGACAAAGGGGTTCTCCGCCAATGGGATGCTACTGCTAAGGTCCTTGGGCACTTGGAATGTAAACCAGACCTTATACTTGGGAACTATACTGATGGAAACTTGGTGGCCTCTCTAATGGCTAACAAACTTGGAACTAAGTATGAAGATTCTGACATCAAATGGAAGGAGTTGGATCCAAAGTACCACTTCTCATGCCAATTCACAGCTGACATTATTGCGATGAATTCGGCCGATTTCATAATTACCAGCACATATCAAGAAATCGCAGGAAGCAAGAATAGGCCTGGACAATATGAAAGCCATATGGCATTTACCATGCCAGGCCTTAGCAGAGTAGTTTCAGGCATCAATGTCTTTGACCCAAAGTTCAATATCGCTGCTCCCGGGGCTGAACAAGAAAAACCAATCATCTTCTCGATGGCAAGGCTCGATACGGTGAAGAACATTTCCGGATTGACCGAGTGGTATGGCAAGAACAAAAGGCTCAGGAACCTGGCAAATCTTGTTGTCGTTGCGGGATTCTTCGATCCGTCCAAATCAAAAGATAGGGAAGAAATTGCGGAAATCAACAAGATGCATGCTTTGATACAGAAGTACCAACTCAAGGGTCAGATCAGATGGATAGCAGCACAAACGGACAGGTACCGCAACGGAGAGTTGTACAGATGCATTGCTGACACGAATGGGGCTTTCGTGCAGCCGGCGCTGTATGAAGCTTTTGGTCTGACAGTTATCGAAGCAATGAACTGTGGATTACCCACATTTGCAACCAACCAAGGTGGACCAGCGGAAATCATAGTCGATGGGGTTTCGGGTTTCCACGTTGATCCAAACAATGGCGACGAATCGAGTAACAAGATAGCCGATTTCTTTGAGAAGTGCAAGACGGATGCCGATTATTGGAATAGGATGTCTCAAGCAGGTCTCAAGCGCATCTACGAATGCTACACATGGAAGATCTATGCAAACAAAGTCTTGAACATGGGGTCTTTATATGGCTTTTGGAAGCAGTTGAACAATGAACAGAAGAAAGCTAAGCAAAGATACCTTCAAATGTTTTATACTCTCCAATTCAGGAATCTGTACATATTTCACCGTGATTTCAAACATGCTTCAACTCGTATGATAATTGTTCTCAGGGAAAACGGGTTAGAGCAGCAGAAGCAGCTTGCCTCACCGAGAGATTCTCACACTTCCTGCCCCTGCTCTTCGTGGTGTTTCCTGTTTCTCTCTGTTTCCATCATTATCTACGCTGCTATGAAGTACTATGGCTTCTTCAGACAACCATATCCAATGTGTGGATGCTACCAAAATGTGCGATTCTTCTGA
