# Supplementary material for: Genome-wide identification and expression profiling analysis of sucrose synthase (SUS) and sucrose phosphate synthase (SPS) genes family in Actinidia chinensis and A. eriantha
Source: BMC Plant Biol. 2022 Apr 26;22:215. doi: 10.1186/s12870-022-03603-y (PMC9040251; doi:10.1186/s12870-022-03603-y)
Supplement: Supplementary file 3 — Additional file 3. [file 12870_2022_3603_MOESM3_ESM.docx]

Supplementary file 3 Genom sequences of members of the *SUS* and *SPS* gene families in *Actinidia*.

>AcSPS1

ATGGCGGGAAACGACTGGATAAACAGTTACCTGGAGGCGATACTGGATGTGGGGCCAGGGATCGACGACGCGAAATCGTCGTTGCTGCTTAGAGAGAGAGGCAGGTTCAGTCCCACTCGCTACTTCGTCGAGCAGGTCATCGGTTTCGATGAGACCGATCTCTACCGCTCCTGGGTTAAGGTATATCACAACATTTCTCTCTCTCTCTCTCTCTCTCTCTCTCTCTCTCTCTCTATCAGTGTGTATTGATGTATAATCGTGTGAATCGATACGTGTGTAATTGTTGATTGTCGGTGGTAGGCGGCGGCGACGAGAAGTCCGCAGGAGCGGAATACGAGACTCGAGAACATGTGCTGGCGGATTTGGAATTTGGCTCGCCAGAAAAAGCAGGTTTCTCTCGCCCCCCTTCTCTCTCTCTCTCTCTCTATATATATATATATATATATCTGTATATATGCCACAACTTGAAATGATTGTTGCCGATTAAGTGAAGGACTTAAGATTCTTGATGTGGAATCCTGCTTTGAATACGTATATACGTGTGTATAGTTGTCTAGGTTTGGAGTGTTAATTGAAATGGTTGTTGCCAATTAAGTGGAGGATGCAAGATTTTTGATGCGGAGTTTTGTTGTATACTTGCACACAGTGGCGGACCCAGGAATTTTTTTTAGGAGGGCGAAAATTTTTCGCTAATAAGATGATAAATTCTAGGATAATATAATGAATACGATAATATTCAAAATTCTATATATTAATAGCAAATGTTTCTTACACAAAATAATGTCCAAGTCCAAATAAAAATAAATGACAGATTACAATAATACTCTTCGTTGTTTCATATTTTGAAACCTTTGCAAGATAGCTTCATTGTCTATGGTTTTGAATATGTCCCTTTCAATATATGACACCAAGCAGTTATTCAACCATTCATCTCCCATGCGATTACGTAATGAAGATTTGATAATCTTCATTGCAGAAATTTTTTTTTCAATTGATACAGTTGCAACCGGTAAGATCAATACCAACTTCACAAGCAAATAGACTAATAGGTAGACAATATCCTTTTTAGTCTGAACTAACATTTGAGCAAGCCCACCAATCCCTATCACTTCTGAAAATTGACCACCAGATCTTACATCTATAATGTAGCTCTCAAGTTGATTATCAAGTATCATAAGCTCCAGTGAAAAAAAATCATTTAGATAAAACTCAGCAAACTGAAGCAACTTGCCCTTATCAAAATTAGAGAATGAACTGCTTGGATCAAGAGATGCCATACACAATAACAACTCTGTATTCACCTCATTAAAGCGGCTATTTAATGCATATTTATCACTGTATAAAATAACTCAACACGATAATGGTGAAGATGTGTAGTTTCTTGAGCATTCCGACGTGATCTTCCTCGAAGTACAAACAAATCATCCATGTTTGGAATAATTATCTTTGGAATAATTATATGTGTCAAAATAAATTTGTATATGTTTATTAAGTCAGCAACTTAAGCAAAAAAATAAGGGATTTACAGTAGTTGACTCACCCACCAAAGAAAAAATCGAATTTGACACGGGGAGAAAAGAGTCTGAGAGGGCTTGAACCAAATACAAAACAAAGCATTACCCACAAAACACAAAACAGAGACCCCGATATTGCTAGACATTTTCAATCATGAGTCATGACTAAAAAAAAGTTAGGAACCCCAAATAACTAAATCAAGGAACAATCATCACAAGTTCACAACGACACAATCCATATGAAAAACCCATAAAACTAGATCAAAACAGAACACAAAATAATAAATAAAAATTGAATATTTACTCAAAAAGTAGGGTTCTTACTTGCGCAATTGGGGATTCTGATAGAGACCCAGAAGCAGAAACGATATATTTGTGAATCCGACGGCTGGAGAACGAAGATCAGCTCCTTTGCAAGACTGCAACTGCGTGAGAGATCTAGGGTTTCGCTGAGCGGCTGAGAGGGGCGAGAAGAACAGAGGAGAAAGAGTCTGAAAGGGCTTGAAACAAATAAATTATAAACGTAATTGTTTCTTTTTTAGGAACATAAATTGCAATTTAGGCCCAGACCGAAATTTAGAAATTCAATTTTGGCCCAACCAATTAAATTTTTTTTTTTTTTTGCGTTTTTTTTTTCTTCAGGGGGCGGCCGCCTACCCTCGGCCCTTGGTAGGTCTGCCCCTGCTTGCACATGCACAAGTGAATTGCTGTGGTTTCAACTCTAGGTTTGGCGTGTTATTTGAAATAGTTGTCACCCGAATAAGTGGGGGACTTAAGATTCTTGATGTGGAATTCTGCTTTGGATACTTCTATACATGTGTATACTTGTATGAACACCAGCGGATTACTGTGGTTTGAATTCTAGGTTTGGAGTATTAATTGAAATGATTGTTGCCAATTAAGTGGAGGATCGTGAGATTCTTGATGCGGAGTTTTGTTGTATACTCGCACATGCACAAGTGAATTATGTGGTTTCAGTTATAGGTTTGGTGGTGTTATATGTTATGGCTGGTGCCGACTAAATGGTGGACTCAAGATTCTTCATGGGGGGATTTAAGATTCTTCATGTGGAATTGTGTATTTAATTTTCTGGTCCTTATTCTTAATTACGTATATATGCACGAATGAATGACTGTAGTTTGAATACTAGGTTTGGAGTGCTATTTGAATGACTTAAGCTTCTTGACGAGTGGATTTTGGTTGTTTAATTCTCCGGTCCCCGCGGATGATTGGGTGGGTGTGTTTTACTGATCGGCTATTGGTTCATTCTGTGGTTTGATTTTGCTTTGATGATTTGAATGCAATTAACCATCGTTTTACCATTGAACTTCTTTTGGTTTCAAATTTTACAATTTAATTATTGTGTTAGGGAACAAGATGCCACATGCGATTGCAAAAGAAACTTTTGTAGGCCAAAGGGAATGCATTCGATTATTAGGCTACTTTTTGTTTTTATTTCTGTCATTCTGTTATTAAAAATCAACTTACTTCTTTTGGAAGCCTATCTTAAACTCTTAGTGGTGTTTAAAAATTGGAAAAGCCTCGTTTCGTAGCGGGATGCTTTGTTGGCAGAAAACCAATAGTGTGTATTTGTCATAGCCATTTTAAGAAGGTCTTTAAACTTTTTCAATGTTTTTGGGTTGTCATTACTAATTTACTAAAAATGACGTGGCTGTAGTACCATGTTTTGTATCTTGCTGGCATTTTAATTTGATTTGCACATAAACGTATTGATGAAGATTTTACCGAGTTTAAGAGTGATACTCTAGCCATCTATTTTGCTTTGCTTTGTTTCAACCATTGTTGTTCCAGCATAAAATGTATTTCTTTTTCTACATTTGGAAGTCTATATAACTATATTGAATGTCTTTGTTTTTTTGGTAACTATGCTTCAAAAGGAGGCACCATATTCTGGTATTTGGATTCCTTGAATCTTGTCCTTCTTGGTGAAATTGTGAATAATTAATTCAGTTTTATTTGTTATCTACGTAGCTTGAGGGAGAGGAAGCTCAAAGGATGGCTAAACGTCGTCTTGAACGTGAAAGAGGACGCAGAGAAGCAACTGCTGATATGTCTGAAGACTTATCTGAGGGGGAAAAAGGAGATACAGTCAGCGATCTGTCGGCTCATGGTGAAAGCAACAGGGGCCGATTACCTAGAATTAGCTCCGTTGAGACAATGGAGGCATGGGTTAGTCAACATAAGGGGAAAAAGCTGTACATTGTCTTAATAAGGCATGAATTTGAATTCCATTCATTTGGCTTGCAAATCTTGTAATGAATTGCTATCTCAATTAATCAGTTGTTATTGTGATATTGCATATCGAGTACAAAAATTGGTTGTTTAATTGTGTTTTTAACCTGTAATTGTTGAATTAACTTTATGAGCTGTAGAGGAGAAACTTTTTTGAGGTCTAAACGTTTCCTGTAGTAGGTGTGCACTTTCTCCTGAATGTTATGGTGTAGAAGGATATGGTATCTCTTGCATGAGCAATGTCTTAATTATTCTTAACGGTTACTATTCTTGCAGCCTTCATGGTCTAATACGGGGTGAAAATATGGAGCTTGGTCGTGATTCTGATACTGGTGGCCAGGTAAATTGTAGTTGCAAGAGCTTATAAAGTCAGTATCCTAATCAATTAAAACTGCAAGTCAATGAATGCGAGGTTGCTTGTGGAGACTACTAAAGTTGTTTTTTTCCCCTTCTTTTCTTTTTTCTCTTAGGTTAAGTATGTTGTGGAACTTGCAAGGGCTTTGGGTTCAATGCCAGGAGTGTATCGGGTTGATTTGCTCACTAGACAAGTATCATCACCAGAAGTAGACTGGAGTTATGGTGAACCCACTGAAATGTTGCCTCCAAGAAATTCTGATGGTTTGATGGATGAGATGGGGGAGAGTAGTGGCGCTTATATTATTCGTATTCCATTTGGCCCAAGAGATAAATATGTACCGAAAGAACTTCTGTGGCCGCACATCCCTGAATTTGTTGATGGTGCTCTTAACCACATCATACAGATGTCCAAAGTACTTGGTGAGCAAATTGGCAGTGGGCATCCTGTGTGGCCTGTTGCTATCCATGGGCATTATGCAGATGCAGGTGATGCCGCTGCTCTTCTATCAGGTGCTTTAAATGTACCCATGCTTTTCACTGGTCACTCACTTGGTAGGGATAAGCTGGAACAGCTTTTGAGACAAAGTCGATTATCAAAGGATGAAATAAATAAGACATACAAAATAATGCGTCGTATAGAAGCTGAGGAGTTATCGCTTGATGCCTCTGAAATAGTGATAACTAGCACTAGACAGGAGATAGAACAGCAATGGCGTTTGTATGATGGTTTTGATCCGGTGATAGAACGGAAACTACGAGCCAGGATCAGGCGTAATGTGAGCTGTTATGGCAGGTTCATGCCTCGCATGGTTGTAAGTATTGGGATATCAGTTCTGAATCTGTTATGACTTGGTTCTTTTTGTTCTATGGTTAAAGTTATTAGTAACTATACACTGGAATCAACATGGATTGCTCTTATTTCTTGCTTATTACTATTGAGGCAGGTAATGAGGGTAACATGCTGGTCTTGAATTCTTCTTCTTCTGCTAGGGGCAAGACAGCGATAATTTTCTTGTTTGTCAGATCACAATACTTTGAGCATGAGTTACAGTAGCTTCAGTCTTACTTAGCTTGTCAAGATTCCATAAATGAACCTTTTTGTATCTTTGAGAGAACTCAACATGAAGCAACAAACACAGGGAACTCAACTGCGGCAAATTACATCTAATTGCACTGTTTGTACGTAGAGATAGAATTGTAAAAACGAATTTCATGATGCTTGATGACTTTGATCTCAATTGTAGAATTAAAGCCTTGACTTGTATATAGTAATTGTTAGCTTCATGAATAGTGACAGTATTCATGATAGAGATGCTGACTTGTCTAATTATGTAAATGATAAGTTAACATATGTGCTAAAATTGAACTGAGCTTGAACTTGCGAAGAATCTGGCTGTCTAGATTGGTTCGTTTTAAATTGTGGTCTCAAATGATAACCAAATGTAAATGGCTTGCTAGTTGGAATTACACTTCGTAATATTACAGCTTTCTGCAGGTAATGCCCCCTGGGATGGAATTTCATCACATTGTTCCACATGAAGGTGATATGGACGGTGAAACTGAAGGAAATGAAGACCAGCCTACTTCTCCAGACCCACCCATTTGGCCTGAGGTTCCAATCATTTCTCTGCATTTTCTTTGATGCGTTCATAGAGTGATAACATGACTGCAGTGCTAGTCCTTCGGGATATAACTTTTTCTCAATCCTCGAATGTATTGAGAAGTTCTTGCCAATATCTAAGTTGACGTTATGATATGCAGATAGTGCGCTTCTTTACTAATCCACGCAAGCCGATGATACTTGCCCTAGCTAGGCCAGATCCCAAAAAGAATCTCGCAACTTTGGTTGAAGCATTTGGGGAATGTCGTCCATTAAGAGAGCTGGCTAATCTGGTACTTTTGGATCACCTCTTGTTCTGTAGCACCTTTTATTTTGGTTGAATGAATTGTTTTGACTGTACTAAGCTGCTGAATCAGCTTTGACATTAATGTCCTTTGTCCTGTATTTTGCAGACTTTAATAATGGGTACTAAGCTGCTGAATCGGCTTTGACATTAATGTCCTTTGTCCTGTATTTTGCAGACTTTAATAATGGGTAACCGAGGTGATGTTGATGAAATGTCAAGCACTAATTCTTCTGTTCTTCTCTCAATACTTAAGCTTATTGATAAGTATGATCTCTATGGTCAAGTGGCATACCCCAAACACCACAAGCAGTCTGATGTTCCTGATATCTACCGTCTGGCAGCTAAGACAAAGGTACTCTTTCATAGGCTATATGACTTGGCAGGTTGGATATGGATCACTAGTTTAAAGCTGAAATAAAAATAGGACGGATGTTAGTTAATCTATTTATAGTACTCTTCTAGTTCCCTATGAGGTACATCAAGACAATTACTTCCCATGATACCTAAATAGCCAGCTCTCTCGGAACCTACTTATTGCTTTCTTTATTTTTTTTCTTATATGAGTCTGCTCTCCAGGAATCCTGCTTGTTACTTGGGCAATTCTAGAATCCTTTTTGCTACAGTAATCTGTATTTTTTCTACAATGATGAAACTTATCCCACCAAGTTTTGGTAGCTCCAGGAATCAAATTTTTTTTCTTCCATGCCGCTTTCTCTTTGGCCATACCTTCTTCCCTTTTGTGTTGTTTTAGAAAGTACGAAGGTTTATGTTCATGCACGTGTGCGTCTCTCCTAGTTGCAAGTTGTGTCCATTTGTACTTTTTCTTGCAGTTAAATTGTATTATTTTGCATTTATTGCATAATATGAAGAAATTTATTTTATTTTTTGTATTAGACAATTTTGTAGAAGTTTATGTGGCTACAAAAGATGAGACTTTTGTATGGTATGCTTCTATTAGAGATACAATGGTAGACAAATAGGTTGTTTCGATCGGACTTTCTGCAGAAATAATAATGCACCACTAGGTAGTAAGCAGGAGCTGTGAATCTGAGCGGGAAAGTACATTCATCTACGAATGGTTAAGGAATTTAAGTTGTACTTGTAATTGAAATGCATGATAATAGAAAAAAAATGACATTTCCTAGTTTTCGCTGGACATGAAAGCATTACAAATGTGGTTTTCAGTCAATGGACTGGCAATACTAGAAGATGCTGAACTATGAGTTTTATGGTTGTTTGAAAACATGAAATATTTTTCATTGAGGAAAAAGTAACTTGTCTTTTATTTGACCGACAAAAATAGGTTGAATCTGGGTAAATAATTTGTTTTAAGTTCTTTTCGTCTCTTGGTTTTATGTAATTGGCTACCATTGCACTGTCCTAGAAATCAACTCAGTGCTGGGAGAATATGAATTATAACACTTGAAGGGAAAGTTAAATAGCCTTTTCTGGGACAAATGAAGCCCAGATAACTATTAGAAGCGGCTTTGATCTCCTTAGTATTTATACCAATAGATGAATATGTCTGATGACTCTGATCCCTTTACCGTAGTTTCATCTTTTCTCTCACTACTAAACATGGTTTTAAAAATGGGCAGCATTTGTTACGTAACGGTATGGGTCACTCCCGCTACTTGTTGCGGCCGCTATAGCAGTGACACTGAAATTCATATGCATAATGGCTACAACCGTTATGTAACTCCCGTTACACCGCATTTACGACCGGTATGACGGTTAAGTAACCGTCAGCTAAGAAGCACGGAAAAGGACACGGGACAAGGCAATTTTGAAATATGGAGGACACAGATATGGCGGGGACACGACAAAAAATTATTATTATTTTATATATTTTTATTTTTTCCTGTATATAAATATAACATCAAAATACCAACAAAAATAGACAATAGGTCATCAATTCATCATCCATCAAGTATAGAGAAAGATACAAGTTAAAGAAAATCATCACCCGATACATCAGTAGATTGATTCATTAATGGCTAAAAGTATGAACTAGCAATTATCCTACTTTCACAAAAAAAATACGACCTCCCAAATAAAGAAACAAAAAGAGTAGTTTCAGACGTGTCCACTGCTTAAAAACGTGTCTCAGACGTGTCCTCGCCGTGTCTCAGACGTGCCCACTGCTTAAAAAATTGTTTAAAAAACAGGCCTGGCGTGTCCGACACATATTTTGGCGTGTCCGTGTCCGCGGTGTGTCCGACACGCATACGGCCCCCTAATCGGTGTGTCCGTGCTTCCTAGACCACCAGAGTATGTCCATTTTTCACTTTTAGCTTCTAGCTTATATTTATTTAATCTACTTTAAAGCTTATAAACAAAACAACTTCTTTTTTTTTGTGTGTGTATTTCTGTGTGTGCGCAGTTGGAACTTACAAGTTGTATGTTAATTTGTCAAATAGAGTATAATAATGGTATATTTTGCTTTTTATATTTTATTTTCATTAGTTACATTCACCTATTTTGTTTATTTAAAAACACCACATGAATCGCAATTGTATAATTATTAAATAAATAAAGTTCAATAGACTAGTGCATTATAAATTTATGGAACTTGAAACTATTCACCATTTATAGAAAATATCTAAATATCTCGGCTTAGTGACTTAAAAATATTGGAATGCAAATAAGATTCTAATAAACCCATGAAAAATATTTTAATCCCAAAAATTCTGAAATAAAATCATGCACATATCAGGAAGTTTATATGATAATGTGAAAAACAGAAAATCTCTGGTTGCGCTACTGTTACGCTACAGTCGTTACACCCTGAAACCGCGTCACCACCACCATTACTGTTACGGTAACTACAACCGATTTTTAAAACCTTGCTACTAGATAGTTGTCTATCGTTCTCATGCAGTCGTTACAATTAGTTGCCATGTGATTTCTTTTGTCCTGTCCAGTGAAGGCTCTGAAATTAGCTGATCTTATCATACTAGTTTGAATCTTGATGGCATTCTCAGATGATAATAACCTGTAAGTAATGAAATTTAAAGGGTTCCTGCCCCTAAAGTTTTCATCCTTATAATTCGCCGAACATTTGTCTATTGTACAGTGATCGTTTCGTTTCTCCACGTGTGTCTGAATCCTTTGTATTTTAGATCATTTGATAAGGCATCTTAACATTGCATGAAGTCCTCCGAAGAATATAAAAGGTGTTTTTGTGGGCTTTAATTAAAATTGGATTGCTTTCATGGTACTACCTTATCTTATCAAGAGATTTTTAACTGCATGAATTATGGATTAATTATTTCTTTAATTTGGTCTACTTTTTTTTTTATCGGATTTTGTCATGTTTAAGGTATGATGGTTTTATAGCTGGACACTGAATAAGAGAAAATAAATTATGTCTTCTGCTCAGTGGCATGAAAGCCATTTCATGTGGCTTTTTTTATACAGGATAGCCAAAGTAACGGTTGTCATTGTAATACAATAGTATGGTATAAAGTCATACATGCTAAGGTTGTTTTAGTTCGTTTTCTGCTGTATTTATGTTCTCTCTCTCTCTCTCTACCCACCTTCCCCCCGCCTCTCTCTCTCTCTGACTTATCATATTATTAGATATCTTATTCTGTTGCCAAATTTTGCTAGGGTGTTTTCATTAATCCAGCTGTCATTGAGCCCTTTGGGCTTACTCTGATCGAGGTACACTTAATATACCTGTGAAGCCCTTAGATTTCATTAGTTAGTTGACTATAATCAAGTATGACTTCTTTTCCATTGAACAGGCAGCAGCTTATGGGTTACCAATTGTCGCCACAAAAAATGGAGGTCCTGTTGACATACATCGGGTATGTCTACAGATCGGTTCCATTTTTTTGATCGTCGGACTTCTATCGATATAATAGTGACGTGTGAAGGTTATGCTGACAACAAGAACTCTACTTCTGAAGATCATTCTGATTACAATTACTGTATATCTGTTGCAGGCTCTTGACAATGGTCTCCTTGTGGACCCCCATGATCAGAAGTCTATTGCTGATGCTCTTTTAAAACTGGTTGCGGATAAGCAACTTTGGTCCAAGTGCCGCCAGAATGGGTTGAAAAATATTTACCTTTTCTCATGGCCAGAACATTGTAAAACTTACCTATCTCGAATAGCAGCTTGCAAACTGAGGCAACCATGGTGGCAAAGAAGTGACGATGGGGATGAAAATTCTGAGTCGGATTCACCAAGTGACTCCTTGAGAGATATATCCTTGAACTTAAAGTTTTCACTGGATGGAGAAAAGAATGAAGGCAGCGGAAATGCCGATGGTTCTTTAGAATTTGAAGATCGCAAGATTAAGTTGGAGAATGCTGTTTTGACATGGTCAAAGGGTTTCCAGAAGGGCACACAAAAGGCTGGGGTTACAGAGAAAGCAGATACGAACATCACTGCTGGAAAGTTCCCAGTGTTGAGGAGGAGGAAGAATATTATTGTTATTGCCGTGGATTTTGGTGCTATATCAGATTATTCCGATAGTATTAGAAAGATATTTGACGCTGTGGAGAAGGAAAGGACTGAAGGCTCTATAGGATTTATATTAGCAACATCCTTTACTTTGTCCGAAGTCCATTCTTTTCTCATCTCTGGCGGACTGAGCCCTTCTGATTTTGATGCGTTTATCTGCAATAGTGGTAGTGATCTCTACTATTCATCTCTTAATTCAGAGGATAATCCCTTCGTTGTTGACTTATATTACCACTCACATATTGAATACCGCTGGGGTGGAGAAGGGTTAAGGAAGACTTTGATTCGTTGGATGGGTTCTATCAATGACAAGAAGGGCGAAAATGAAGAGCAGATTGTTACCGAAGATGAAAAGATTTCAACCAATTATTGTTATGCTTTCAAAGTGCGAAATGCAGGGAAGGTAATTTTTTTTCTACTTGTGAATGATTTCCTTATGAAGTGGCTTGTTGAATTTACTTTACCTACCCTTAGGGGTGTCAGTCAAACCGAGTAGCGCGAGATTGGCTCGCTCAGTCACTCTTGACTCTGCTTGAGCTCATTTGAGCTCGGTTTTGAGATAAAGTGAGTCAACCTCAATTTTGGCTTGCAGGAAATGTGTTGGAAAGGGTAGAGCTTATATGCTATCGAAGAATACCAGAACGTAATGGACGCGTTTACATTAATGAGATAACGAGAGAGCTTGAACAGTACAAGGCACTTGCATAAACGTGTCAATTCGAGGATTTTTTTTTAAAAATGATTTCTTTCACTTTTACTGTCATTTTCCTGATTTTTGTTGCACGGGCACATAAATGAAAGTGGTCAGACTTTTTATTCTTCAACTTGCACTAAGAATTTAATGCCCATAACTTTTACCTATTTGACCATAATATTTCTATTTAAGTTTTGTACTAAGTTGGGCAACCAGATAAAAGCATCACTTTTTCGAGTCAAGCTGGGCCGTTTGTTGATCAGGGTGTTAAGAGAATGAGCTGAGCTGGAGCAAAATTTTGAATATTTACTTGAGCCATTCACTGAACATTATTGACATTTTCTCGAGCCAAGCCTGGCCAATAATGAATTCGGCTTGGCTCATTTACATCGCTACTCACCCCACCTCCTCATGCAACTGATTTTCATGCGAGGACTTATATATGAGTAGCGGTGTAAATGAGCCAAGCCGAGTTCATTATTGCCCAGGCTTGGCTCGAGAAAGTGTCAATAATACATACATATATATATATATATATCTTAGCTTTATGAGACATAAAGCTGGGGTCAGTGGTGGCACAAGCATAAGGCCAACAAAGTCATCGCTTTTGCTTTTGCCCCCCGATTTTAGAAGACCCCAAATTTTTATTAGAGTTATGTAGGTTTATAAAATTTCTTATAATTTGCTATAGTTAAAGAGCCCCGCCGCTGTTAAAAAAAAAGTTTAAAAATGCCCACCAATGAAGACAATGCTAAAAAATAATAACCACTATAAAAAAATCCAAAAGACACAAATCCCCAAAATTTGTATAAACCGTATAAATATCACTTCCAAAAGAAAAAAACAATATATTTCTCCACAACGATGATGCATGAAATTCTAGACAATCACCGGAAGAATCTTTAAAAACTAATTATTTTATATTAATTGTCGATCAATTTGTATCTTCACTTGAAAGTAGATTTGAATAATTGCTAATATATAGTATTTTTTTGATTTTTTTTAAAAATTTTAAAAAATTAAAATCATCAGATGACGGTATTCATTAATTACTCAAACTAAAAACAAGATTCATTTGAAAATACATGCATTGCATATTGCATCTTATTGACAATATCTATTACAATTGCTTCTGCAGAAAGAAGGTTTTTGAAATTAAAACTGATAAAATTTAAGATGAACATTGTGACAAGAAAAATTAAGTGGATTAGTTGTACTATCTATTAGAAATAACTTGTGAGCAATACTAAAATACAAAGTCTTAAATCAATAACTTTACATCTAAAAGGCAAAATAAATTTTTAAATAAAATAGTATATAGGCCTCATTTAAAGTTACGCTTGAGGCCTCGCATGTGTCTGAGCCGGCCCTGCTTGGGGTGGGTTGATATTGCTAAATATCATAGGCAATATTCTAGTTCCGGTAAGATAGTTAAATAAATTTAGAATTGTAAAGTTCTCTGCAACTATTTCTTTGTGTGTGTGTATTTATGATCTTTGTGCATCATCGCGTATCATTCTTCTTTATCTTATTATTGTTACATCTTCACAGGTTCCTCCTGTCAAGGAAATCAGAAAATTAATGAGGATTCAGGCTCACCGTTGCCATGTTATTTATTGCCAGAATGGGAATAAGATAAATGTAATTCCAGTATTGGCGTCTCGTTCCCAAGCCCTGAGGTATTGCGCTTATAGTTTCAATTAATTAGTTGTTAGCTTATTATCCTTTTTTTTGGTGGTGGTGGTGAATATTCCATGATATGCTGACTTTAGTTGATCTTTTAATCAATACACAAAATCCTCAGTATTTTGTTCTTGAAATATGATGGGAAATAGTAATTGTTAATTAGGTCCTACTAATCTTATTTAGGCACAAATAATGAAATTCTACCGTTCGTCGGTGTTCGGTATTGTGTACACAATTAGCTAGTCATTAAAACCAAAACATGTGACAGGAACAAGGTGCATATAGTTTGAGCACTGCCACAGTCCAAGAATTTGGACAAAGAAAAGTATTCAGAATTCAAAATAAATTATTGAAATTCACTTGGTTATGTGGAATCCACACACTCAAATGAATATAAACCAATCATTTTGCATTATGGGTACTTTTCTTGGTTCAAATTCTGAGAACCTAGGGTGTCTCGTACAGTTTTAGAGCAAGTTGGGTAATATAAAACTTAAATGTCGCACTATGATTGCACCTCCAATATAAATGCCAAAGATTACATTTTTTATGGATATCTTACTGAGTTTACCCCTCATCTTTTTGGTATGAGGTGTTGTAGTTGTTCCACTTAATATGTTTTACATATTTTTTAGAAAAGGAGAACTTGACCCTTCCCTAAATTTGTTGTAGATAAACAGCAGTAAAAAATTCGGTGTCAATGTTATATATGTCTACAGCTAACACACTGAATGATGAGGCGTCAACATTTTTTTTGGCAAAGATTTTATGTATGTGAATTTTTATCATAAGATTATCATTAGCCATTTGAACCAATTCATGTTTATGTAGCTTTCCAGTTGCCACTATGTGGGAACTTTTGCATTGGATTTAGTTTTAGCAGGATATGGTATCCAAACATTGAAACGGACTTAAAGTTGCCCAATCAGTCATATCGCTGTTAGCAGCAGGAAGATCCACAGCACTCTTTTAACTGCAGAAACCTTTTTCTCTTTCCTTTGTGGTTGGAGTGCTACTGTGCTGGTAATTGGAACCACTTAGACTGTCAATGTGAGCAAATTGGGCTGTCTAGAAGATGACACATTGTGTGACCTAGATGCACCATTTGACCTGATTTGCCATGACATTTTAGCCAGAGTCCAATTAACTTCAGATTTGGAGTGAGTGAGAGAGGTTGCCTCGGTGATTATTAAACACTTCCACATGTCACTAGTTGTGTTGAACCCATCAATGGATCCATCCTTTGGCTAGATCACTATATAACCTAATACGATGAGCTCATAGTGTTAAATTTTAAATAGAGAAAGATGAGAAGGAGAAATAGAAGACATAAATTAACTTGGTTTGGTCATATTGACCTACATCCACGGACATAAATGCCCATAAAGGCTTTTTCTTCTTTATTGATAGATCATTATGTGTCTAATACATAAAATACACACCTATTTATATAGGTGGAGATTGAATAAAATCACATGTTCAATGGTATTGAATGATATCGATAATCCCACTAATTGTGTCTTTGGCCTACCACATGCCAATAATCAAAAGTCAATTAACCCACTAATTATGGGATCTTCAAACTCTAACACATAGGTTCAGTAGGCTCCTGTGGATGGTGGAGGATATAAATTTCATCACCCCTTTAGTTAGAAGCTCATAGATCTCAATACTATAGCATCAGACGTTATGCTGCCTCGTGGAAGATATAATTGTTTAGTTCATATCCTTTTTAAAAAATTAAGTGCGTGTTTTTCTATTGTTATTGTACTTTTTGATCTTCTTAATTTTGAAATAAAAATGGCGAGAATTAAGAAAAAAACAATCCTCTCCATCATTTTCGATTTTATAATGAATCCAGTTTTTAGAAAAGAGAAAGTTAAAACTGCTTTCTTTAAATGCACTGTTTCAAAGGATTATCTTGAAATTAGTTTTGCTACAATGAATTTTCTGCATCTTTTCCCATTTTTGTCTGTTTCTGAAAACTGGAGTCGTTTGAAAATGAAACCAAATCTGCTTTAAGTTTTTGGGGCAATTGTTCTCCTGATTTGCTGTATCCTTTTTTGTTTGTCGGAAACCTTCTAAAATTGCAGCGAAGTGGGTACAACGGGGATATGTCAATAATAAAGTATAGTTTATATTTTGTTACTATAGATATGACCATAATAGATGCAATTTGTGCTATTCTTTAAATGTTGAAGTTAAAACATTTATCAAGAGGAGAGTTATTCATGTTTTCTTTCTTATGAAGTGTAGATATGAATATAAGACGCGTATGAGGCCTTAAGAATATGCAAGTACGATAATCTGAAAACATAAGGTACTTTTAAATTCGCTATTTTTATCGTTCTTATAAATAAACCAAATATGGCAACTATTCTTGTATTTATCCTCTCAACTGGAGTATAGTCCAATACTGTGAAAGAAATGCTATAATCTCAATATAGTAATAGTTAGGAAATGTGATGGAGAGTATCTGAGAAGTATCCGATACTTCTCAAACTAATGAAAATAAAGTTTAAATTACGTATACTTTTAAGAAGTTTCAGATATATATTTGCAAGTATCCAAAGAGTATCCATATCTGATACGGCTATGGTACGGGTACAAAACCCTCCCAGAAGTATCCGTGCTTCATAGCTTAAAACTTGTTATTTTGACTGAGCTGTTTCTTTGAACTTGGTATGAAACGCTGTTACTGATAACTCATTATATTCAGGTATCTCTATCTCCGGTGGGGCGTGGACTTGTCAAAAATGGTGGTTTTTGTCGGAGAAAGCGGGGACACTGACTACGAGGGCTTGCTTGGTGGCATACACAAGTCTGTAATATTGAAGGGAGTTTGTAGCGGTCCGACCAATCAACTCCATGCCAACAGAACCTACCCTCTTTCTGATGTCCTGCCGATTGACAGCCCTAACATTGTCCAGGCAGCTGAGGAATGCAGCAGTGCCGATCTCCGGACCTCGTTGTTGAAGCTAGGGTTCATCAAGGGCTAG

>AcSPS2

ATGGCGGGAAACGACTGGATAAACAGTTACCTGGAGGCGATACTGGATGTGGGGCCAGGGATCGACGACGCGAAATCGTCTTTGCTGCTTAGAGAGCGAGGCAGGTTCAGTCCCACTCGCTACTTCGTCGAGCAGGTCATCGGCTTCGGTGAGACCGATCTCTATCGCTCCTGGGTTAAGGTATATCACAACATTTCTCTATACGCTTCTCTCTCTCTCGCTATATATGTATCAGTGTGTATTGATGTATTGTTATGTGAATCGATACGTGCGTGTAATTGTTGATTGGCGGTGGTAGGCGGCTGCGACGAGAAGTCCTCAGGAGCGGAATACGAGACTCGAGAACATGAGCTGGCGGATTTGGAATTTGGCTCGCCAGAAAAAGCAGGTTTCTCTCTCTCTCTCTCTCTCTCTCTCTATATATATATATATATATATATATATATATACACACACACAACTTGAAATGATTGTTGCTGATTAAATTGAGAACTTAAGATTCTTGATGTGGAAATCTGCCTTGAAATGTATATACGTATGTATACTTATATAAGCACAAACGAATTGCTGTGGTTTGAATTATAGGTTTGTAGTGTTAATTGTAATGGCTGTTGCCAATTAAGTGGAGGACGTAAGATTCTTGATGATGAGTTTTGTTGTACACTTGCTCATGCACAAGTGAATTTCTGTGATTTCAATTCTAGGTTTGGGGTGTTATTTGAATTGGTTGTCGCCGAATAAGTGGGGGACTTAATATTCTTGATGTGGAATTCTGCTTTGGATATGTATATACGTGTGTATACTTGCATGAGCTTGGGTGGATTACTGTGGTTTGAATTCTAGGTTTGGAATATTAATTGAAATGATTGTTGCCAATTAAGTGGAGGACGTGAGGTTCTTGATGCGAAGTTTTGTTGTATACTTGCACACGCACAAGTGAATTGCTGTGGTTTCAAATTCTAGGTTTGGGGTGTTATTTGATTTGGCTGGTGCCAACTAAATGGGGGACTTAAGATTCTTCACGTGGCATTGTGTATTTTAATTTCCTGGTCCCTATACTTAATTACTTATACATGCACTAATGAATGGCTGTAGTTTGAATTCTAGGTTTGGAGTGTTATTTGAAATGGTTGGTGCCTGTTGAGTGGGGGACCTACACTTCGTGATGTGGAGTTTGTTTTTTTAATTCACTGATCTCTGCGATGATGGGATTGGGTATGCTTTACAGATTGGGGATTGGTTCATTATGTGGTTTGATATTGTTTTGATGATTTGAATGTAATTAACCATCGTTTTACCATTGAACTTCTTTTGGTTTCAAATTTTACAATTTAATTAGTGTTAAGGAACTAGATGCCACATTCGATTGCAAAAGAAACTTTTGTAGGCCAAAGGGAATGCATTTTATTGTTAGGCTACATTTTTTTTTATTTCTCTCATTCTGTTATTAAAAATCAACTTACTTCTTTCGGAAAAGCCTATCTTAGACTCTTAGTGGTGTTTAAAAATTGGAAAAGCCTTGTTTCGTAGCGGGATGCTGTGTTGGTAGAAAACCAATAGTGTTTTTTTGTCATATCCATTTTTAGCAGGTCTCTAAACTTTTTCAATGTTTTTGGGTCGTCATTACTAATTTACTAAAAATGAAGTGGCTGTAGTAGCATGTTTTGTATCTTGCTGGCATTTTAATTTGATTTGTACATAAATGTATTAATGAAGATTTTACCAAGTTCATCGTCGTCAAGAGTGATACTCTATCCATCTATTTTGCTTTGGTTTGTTTTAATTTTTTGCTGTTCCAGCATAAAATGTATTTCTTTTTCTACATTTGGAAGTCTATATAACTACATTGAATGTCTTTTTTTTGGAAACTATGCTTCAAAAGGAGGCGCCTCATTCTGGTATTTGGATTCCATGAATCTTGTCCTTGTTGGTGAAAAAAAAGTGAATATTTAATCAGTTTTATTTGTTATCTACATAGCTTGAGGGAGAGGAAGCTCAAAGGATGGCTACACGTCGTCTTGAACATGAAAGAGGCCTCAGAGAAGCAACTGCTGATATGTCTGATGACTTGTCCGATGGGGACAAAGGAGATACGGACAGCGATTTGTCTGCTCATGGTGAAAGCAACAGGGGCCGATTACCTAGAATTAGCTCCGTTAAGACAATGGAGGCATGGGTTAGTCAACAGAAGGGGAAAAAGCTGTACATTGTCTTAATAAGGCACGAATTGAATTCCATTCATTTGGCATGCAAATATTGTAATGAATAGCTATCTCAATTAATCAGTTGTTATTGTGATATTGCATATCGCGTACAAAAATTGGTTATTTAATTGTGTTTTTGACATGTAATTGTTGAATTAACTTGATGAGCTGTAGAAGGGAAACTTTTTTGAGGTCTAAATGTTTCCTGTAGTAGGTGTGCACTTTCTCCTGAATGTTATGGTGTAAAAGGATATAGTATCTCTTGCATGCACAATGTCTTAATTATTCTTAACCGTTACTATTCTTGCAGCCTTCATGGTCTAATACGGGGTGAAAATATGGAGCTTGGTCGTGATTCTGATACTGGTGGCCAGGTAAATTTTAGTTGCAAGAGCTTATAGAATCAGTATCCTAACCAATTAAAACTGCAAGTCAATGAATGCGAGGTTGCTTGTGGAGACTGCTAAATATGTTTTTTTTTTCCTTCTCTTCTCTTTCTATTAGGTTAAGTATGTTGTGGAACTTGCAAGGGCTTTGGGTTCAATGCCAGGAGTGTATCGCGTTGATTTACTCACTAGACAAGTATCATCACCAGAAGTAGACTGGAGTTATGGTGAACCCACTGAAATGTTGACTCCAAGAAATTCTGATGTTTTGATGGATGAGATAGGGGAGAGTAGCGGTGCTCATATTATTCGTATTCCATTTGGCCCAAGAGATAAATATGTACCGAAAGAACTTCTGTGGCCACACATTCCTGAATTTGTTGATGGTGCTCTTAACCACATCATACAGATGTCCAAAGTACTTGATGAGCAAATTGGCAGTGGGCATCCTGTGTGGCCTGTTGCTATCCATGGGCATTATGCAGATGCAGGTGATGCCGCTGCTCTTCTATCAGGTGTTCTAAATGTACCCATGCTTTTCACTGGTCACTCACTTGGTAGGGATAAGCTGGAACAGCTTTTGAGACAAAGTCGACTATCAAAGGATGAAATAAATAAGACGTACAAAATAATGCGTCGTATAGAGGCCGAGGAATTATCTCTTCATGCCTCCGAAATAGTGATAACTAGCACTAGACAGGAGATAGAAGAGCAATGGCGTCTGTATGATGGTTTTGATCCTGTACTTGAACGGAAACTACGAGCCAGGATAAGGCGTAATGTGAGCTGTTATGGCAGGTTCATGCCTCGCATGGTTGTAAGTATTGGAATATCAATTCTGAATCTGTTGTGACTCGGTTCCTTTTTGGTTTATAGTTAACGTTATCAGTAACTATTCACTGGAATCAAAATGGATTGCTCACATTTCTTGCTTATTACTATCGAGACAGTAATGAGGGTAACAGCTGGTCTTGAATTCTTCTTTCTTCTTCTTCTTCTGTTTGGGGCAAGACAGCGATAATTTTCTTGTTTGTCAGATTATAATACTTGAACTTGAGTTACTGTACTTCACTCTTGCTTAACTTGTCAAGACTTCATACATGAACCTTTTTGTATCTTTGAGAGAGACAACTCAACCGGAGGCATTAAACATGGAGAACTGAACTGCGGCAAATTACATCTAAATACACTGTTTGTACATAGAGATAGAATCGGAAAAACGAATTTCATGGTGCTCAATGACTCTGATCCCAATTGTAGAATTAAAGCCGTTACTTGTATATAGTAATTGTTAGCTTCAAACATCAAATAATTAATAATGTTCTGTGGAATGTATCTATCAAAATTTTATCAGTAATTCCTCCATTCTTGCTGATATATCCTTAATAGTGACAGCATTAATGATAGAGAATTAGAGATGCTGACTTGTTTAATTATTTCAAATGATAAGTTAACATATGCGCTAAATTTGAACTGGGCTTGAACTTGAGAAGAATCTGGTTTTACAAAGCCAAATGTAACTGGCTTGCTAATTGGAAGTACACATAGTAATATTTCAGATTTCTGCAGGTAATTCCCCCTGGGGTGGAATTTCATCACATTGTTCCACATGAAGGTGATATGGACGGTGAAACTGAAGGAAATGAAGATCAGCCTACTTCTCCAGACCCACCCATTTGGCCTGAGGTTCCAATCATTTCTGTTCATTTTCTTTGATGCGTTCGTACGGTGATAACATGCCTGCAGTGCTAGTTCTTTGGGATATAACTTTTTCTCAATCCTCGAATTTATTAAGAATTTCTTGCCAATATCTAAGTTGACGTTATGGTTTGCAGATAATGCGCTTCTTTACTAATCCACGCAAGCAGATGATACTTGCCCTAGCTAGGCCAGATCCCAAAAATAATCTCACAACTTTGGTTGAAGCATTTGGGGAATGTCGTCCATTAAGAGAGCTGGCTAATCTGGTACATTTGTATCACCTCTTGTTCTGTAATATTTTTTATTTTGGTTGAATGAATTGTTTTGACTGTACTAAGCTGCTGAATCGGCTTTGACATTAATGGCCTTTGTCCTGTATTTTGCAGACTTTAATAATGGGTAACCGAGATGATGTTACTGAAATGTCAAGCACTAATTCTTCGGTTCTTCTCTCAATACTTGAGCTTATTGATAAGTATGATCTCTATGGTCAAGTGGCATACCTCAAACACCACAAGCAGTCTGACGTTCCTGATATCTACCGTCTGGCAGCTAAGACAAAGGTACTCTTTTATCGGCTATATGACTTGGCTGGTTGGATATGGATCACTAGTTTAAAGCTGAAATAAAAACAGGACAGATGTTAGTTAATCTATTTATGGTACCTCTTCTAGTTCCCTATGTGGTAAATGAAGACAATTACTTCCCATGATACCTAAATAGCCAGCTCTCTTGGAACCTACTTATTGCTTTCTTTATTTTTCTTCTTATATGGGTCTGCTCTCCAGGAATCCTGCTTTTTACTTGGGCAATTCTGGAATCCTTTTTTCTACAGTAATCTATATTTTTTCTACAATGATGAAACTTATCCCACCAACTTTTGGCGGACCCAGGAATCAATTTTTTTCTTCAATGGCGCTTTCTCTTTGACCATACCTTATTCCCTTTTGTGTTGTTTTACAAAGTATGAAGGTTTATGTTCATGCACTTGTATGTCTCTCCTAGTTGCAAGTTGTGTCCATTTGTACTTTTTCTTGCAGTTAAATTTTATATTTTGAATTTATTGCATAATATGAAGAGTTTTATTTTACTTTTTGTAGTAGACGATTTTGTAGAAGTTGTGGCTACGAAAGATGAGACTTTTGTATGGCATACTTCTATTGGAGAGACAATCGTAGTTGTTTTGTTTGCACTTCTGCAGAAATAATAATGCACCAACAGTTAAGCAGAGCTGTGAGTCAGAGAGGGAAAGTAAATTCAGCTATGGATGGTTAAGGAATTTAAGTTGTACTTGTAATTGAAAAAAGTAAATTCAGCTATGGATGGTTAAGGAATTTAAGTTGTACTTGTAATTGAACTGCATGATAATAGAAAAAAATGACTTTTCCTAGTTTTCTCTGGATATGAAAGCATTACGAATGTGGTTTTCAGTCAATGGATTGGCAATACTAGAAGATGCTGAACTATGAGTTTTATGGTTGTTTGAAAACATGAAATAATTTTCATTGAGGAAAAAGGAGCTTGTTTTTTATTTGATCAACAAAAATAGTTTGAATCTGGGTAAATAATCTGTTTAAGTTATTTTCATCTCTTGGTTTTATGTAATTGGCTTCCGTTGCACAGTTGCACTGTCCTAGAAATCAACTCAATGCTGGGAGGATATGAATTATAACACTCAAAAGAGAAAGTAAAATTGCCTTTGCTGGGACAAATGAAGCCCAGATAACATATTAGAAGCAGGTTGGATCTCTTTAGTATTTATACCAATAGACAAATATGTTTGATCCCTTTACCGTAGTTTCATCTTTCTCTTACTAAACGTGGTTTTAAAACTGGGCAGCTTTTGTTACGTAACGGTTGGGGTCACTCTCGCTACCTATTGCGGCTGCTATAAAAGTGAGACTGAAATTCATATGCATAACAGCTGCAACTGTTACGTAACTCCCGTCACACCACATTTATGACCGGAATGACTGTTATGTAACTGTCAGAGTATGTTCATTTTTCAATTTTAGCTTCTAGCTTATATTTGTGTGTGTGTGTAGTTGAAACTTACCAAGTTGTATGTTAGTTAGTCAAATAGAGTGTAATAATGGTATGTTTTGATTTTTATATATTATTTTTACTAGTTACATTCACCTATTTTGTTTATTTAACAATTAACACTGGTAACAAGTTTGAAACACCTCATGAATCGCAATTGTATAATTATTAAATACGTAAATAAAGGTCAATAGACCAGTGCATTATAAATTTATGGAACTTGAAACTGTTCGCTATTTATAGAAAATATCTAAATATCTCTTCTTAGTGACTTAAAAATATTGGAATGCGAATAACATTCCAATAAAACTGTGAAAGACTATTATAATCCCAAAAAATAAAATCTGAAAAAAATCATGCATATCAGTAAGTTTCATCATGATGCTAAAAACATAAAAAATCACGACTTTGGTTGCTGCTACAGTCATCATTACACCCTGAAACAGCATCACCACCATTACTGTTACGATAACTGCTACGAATTTTTAAAACCTTGCTACTAGATAGTTGTCTATCGTTCTCATGCAGTTGTTACAATTAGCTGCCACGTGATTTCGTTTGTCCTGTCCAATGAAGGCTCTGAAATTAGCTGATCTTGTCATACTAGTTTGAATCTTGATGATGTTCTTGCATGATAATAACCTGTAAGTAATGAAATATAAAGGGTCCTGCCCCTAAAATTTTCATCCAGACTTGCTACTTAAAAAACCTTTGTCCTGCAAACTTTTTGCACCCGCTTATGATTCGCTGAACATTTGTTTATTGTACAGTGATCATTTCATTTCTCCATGTGTTTCTGAATCCTTTGTATTTTAGACAAGGCATCTTAACATTGCACGAAGCCCTCTGAAGAATATAAAAAGTGTTTTTGTGGGATTTAATTGAAAGTGGATTGCTTATGGTATTACCTTATCTTATCATGAGATTTCTAACTGCATGAATTATGGATTAATTATTTCTTTAGTTGGGTCTACTTTTTTTTAAAAAAAATTTTATGGGATTTTGTCATGGTTAAGGTATGATGGTTTATAGCTGGACACAGAATAAGAGAAACTAAATTATGTCTTTTGCTCTGTGGCATGATAGCCATTTAATGTAACTGTTTTATACAGGATAACCCAAGGTAATGGTTGTTATTGTAATACAGTAGTATGTTATAAATTCATACATGGTAAGGTTGTTTTAGTTCGTTTTCTGCTGTATTTATGTTCTCTCGCTTTAACCCCCCCCCCTTCTCTCTCTCTGAGACTTATCATAACATTAGATATCTTATTCTGTTACCAAATTCTGCTAGGGCGTTTTCATTAATCCAGCTTTCATTGAGCCGTTTGGGCTTACTCTGATCGAGGTACACTTGTTATACCTGTGAAGCCCTTAGATTTCACTAGTTAGGTCACTATAATCAACTATGGCTTCTCTTCTATTGAACAGGCAGCAGCTTATGGCTTACCAATTGTCGCCACAAAAAATGGAGGTCCTGTTGACATACATTGGGTATGTCTATATTGGGACTTCTATTGATATAATAGTGAAGTGTGATGATTATGCTGATAACAATTAGTTCTGAAGATCATTCTGATAACAATTACTGTATATCTGTTGCAGGCTCTTGACAGTGGTTTCCTTGTGGACCCCCATGATCGGCAGTCTATTGCTGATGCTCTTTTAAAGCTGGTTGTGGATAAGCAACTTTGGGCCAAGTGCCGTCAGAATGGGTTAAAAAATATTCACCTTTTCTCATGGCGAGAACATTGTAAAACTTACCTATCTCGAATAGCAGCTTGCAAACTGAGGCAACCATGGTGGCAAAGAAGTGACGATGGGAATGAGAATTCAGAGTCGGATTCACCAAGTGACTCCTGGAGAGATATACAGGATATATCCTTGAACTTAAAGTTTTCACTGGATGGAGAAATGAATGAAGGCACCGGAAATGCTGATAGTTCTTTCGAATTTGAAGATCGCAAGAGGAAGTTGGAAAAGGCTGTTCTGACATGGTCAGTGCAGAAAAGCACACAAAAGTCTGGGCTTACAGAGAAAGCAGATCAGGACAGTACTGCTGGAAAGTTCCCAGCGTTGAGGAGGAAGAATATTATTGTTATTGCCGTGGATTTTGGTGCTATATCGGATCTCTCCGAAAGTATTAGAAAGATATTTGACGCTGTGGCGAAGGAAAGGACTGAAGGCTCTATAGGATTCGTATTAGCTACATCCTTTACTTTGTCTGAAGTCCAGTCTTTTCTCATCTCTGGGGGACTGAGCCCTTCTGATTTTGATGCTTTTATCTGCAATAGTGGTAGTGATATCTACTATTCATCTCTTAATCCAGAGGATAATCCCTTCGTTGTTGACTTGTATTACCACTCACATATTGAATACCGCTGGGGTGGAGAAGGGTTGAGGAAGACTTTGATTCGTTGGGCTGGTTCTATCACTGATAAGAAGGGTGAAAATGAAGAACAGATTGTTACCGAAGATGAAAAGATTTCCACCAATTATTGTTATGCTTTCAAAGTGCGAAACGCAGGAAAGGTAACTTTTTTTTCTACTTGTGAATAATTTTCTTATGAAGTGGCTTGTTGAATTGACCTTACTTGCCCTTAGGGGTGTCAGGCAAAACAAGCAGTGGGAGATTGGCTCACTTTTGACTCGGCTTGATCTTATTTGAGCTCAGTCTTAAGATAATGTGAGTCAACCTCAATATTGGCTTGTAGGATGTGTGTTGTAAAAGTTAGAGCTTATATGCTATCTAAGAATAACAGAACCCAATGTACTCGTTTACATTAATGAGATAACGTGCTAGCATGAATAATACAAGGAACTAGCATAAATGTGTCATCTCTAGGTTTTTTAAATGATTTCTTTCACCTCCACTTTCCTGATTTTTGTTGCACATGCACATAAACGAACGTGGTCAGGCTTTTTATTCTTCAACTTGCACTAAGAAATTAATGCCCATAACTTTACCTATTTGACCATAATATTTCTCTATTGTACTAAGTTGGGCAACCATATAAAAGCATTACTTTTTCGAGTCAAGCTGGTCCGTTTGTCCGTTTGTTAGAGAATGAGCTGAGATGGAGCAAGATTTTGAATATTTAGGCTATTGGAAGCCAAATCCCAGGATCACTAAAAACATGTACACATTAAACCAAAAAAAAAAATTCTTTTTGTGAAATCGTATACTACGGAATTAGGGTTATACATGCGAGATCTCTCTGGCTTGATCTCGACTAAACTTTGAAACCAAAATCTGAAGCAGAACGAACAACGAATACAAACGCGATATGACCTCGCGATCTTCTATTGTATTCCATCCTTGTGTTCTGATGACGAATAGAGTGTGAGCTCTAAAACATGATCTATTTCGCTCTCTTTTTTACAAAATCGTATATATATGTGTGTATGTATATATTTTCAGAATATCTGTGTGTAACCCTAGAGAGTTGTGAATGCACACATAAATATAGGCTACGGTAGAGACCTCAATAAGGTTTGACTTCCTACGGAAAGAAAGAAAGAAAACCAAATTAAACCTGAATTAAATTTTTATTTTTCATAAATTATAATTCATACCACTAAAGAATTATAATTGCACTTCCGCCCTTATTTGAAAATTATATTTCGAGGTCCATATTACTAAATGCTCATTAATCTCACCGCGTTAAGATTAAAATAATTACTGACAATTATAATTAATTGACATCTTCAAAAATTTCGTGAGACCCTCCACTTAACTATTTGATATGTCGGATATAAAATTTACCTGTAGGATTTGACGTAATCGAAACTTATAAGCTTCCTCAAGGGGGCATGATTAATCTCTAAACCAGAACGCGGATTCCGTCAATAATTAATATTCATCATACACGTAATGTGACCATCCAACTCACCAAGTTTGTTGACCCACGAAGGTATCTCACTTCACGAGAATCAAAACAATCAACATCATGTGTCTAAAAATTATTTTGGGATTAAGAGTATAAGCATTCATAATAACCATGGGGTACTAATTGTTTTATAAAGTTAGTACAAAAAGACAATATTCCCAAAGTGTAATAGCACAATGAGTCAAAATTATACCATTTTCCATAGTCAAGAAAAACCTACTAAAACATTGTGCTACAGCCCTACCGATGGTTTGTCCAATTTTATCTAAAACTGTGAACTAAAAACTTATATTCCACAAGAACCGATGATTAGATCTTCCGTGTGTAAGCCGAACTCTACACACCTAATCATCTACTTCGTAGAATAAAAGACACAAGCATAATAGAAATATTATGCTACAAACATTGTCCACATAGATTCTCATTAAAATGGAATGACAAATTTTATTAATAAATAAAATTAATATTTTTTTTTGAAGCTAAATAAAATTAATATTAAGTTCCATAATCATGAATTTTAGTATATTTCTCCAACCTCGGCATGCTTGAGCCATTAACTGAACATTATTGACACTTTCTCGAGCCAAGCCTGGACAATTATGACCTCGGCTTGGCTCATTTACACTGCCTACTCACCCCACCTCCTCATGCAACTGATTTTCATGCGAGGACTTACAGATATCTTAGCTTTATGAGACATAAAGCTGGGGGCGGGTTGATATTGCTAAATATCATAGTCAGTATTCTAGTTTCGGTAAGATAGCGAAATGAATTTAGAGTTATAAAGTTCTCTGCAACTATTTGTCTATTTATGATCTTTGTGCATCATCGCGTATCATTGTTCTTCATCTTATTACTGTTACATCTTCACAGGTTCCTCCTGTTAAAGAAATCAGAAAATTAATGAGGATTCAGGCTCTCCGTTGCCATGTTATTTATTGCCAGAATGGGAATAAGATAAATGTAATTCCGGTATTGGCATCTCGTTCCCAAGCCCTAAGGTATTGTTCTTATAGTTTCAATTAATTAGTTGTTAGCTTATGTTCTTCTTTTTTTGTGTGTGTGCGCGTGTGTGTGTGAATATTCAATGATATGCTGCAGTTGATCTTTTAATCTTTGCACAAAAACCTCAGTATTTTGTTCTTGAAATATGATGGGAAATAGTAATTGTTAATTAGATCCTACTAATCTTATTCAGCACAAATAATGAAATTCTACAGTTCGTCAGTGTTTGGTATTGTGTACGCAACTAGTTAGTCATTGAAACCAACACGTGATAGTAACAAGGTCCATACAGTTTTAGAGTAGGTTGAGTAATATGGAAACTTAAAGGTTGCACTATGATTGCACCACCAATATAAATGCCAAATATTACATTTTTATGGGTATCTTACTGAGTTTATTTCCACTCATTAAGTTTTACATATTTTTTAGAAAAGGATAACTTGACCCTTCTCTAAATTTGTTGTAGCTAACCAGGTGTATAAATTCAGTGTCAATGTTATATATCTTTACATCTAACACACTGAATGATGAGGCGTTCACATTTTTTTGATCTTTTTTTTTATATATGTGAATTTTTATCATAAGATTATCATTTGCCATTTGAACCAATGTCATGTTTATATAGTTTGCCGGTATGTGGGGAACTTTTGCATTGGATTTGGTTTTAGCAGGATACACTATCCAAACATTGAAAGGGACTTAAAAGTTGCCCAGATCCACAGTACTCCTTACTGCAGAAACCTCTCTCTTTCCTTTGTGGTTGGAGTGCTGCTGTGCTGGTAATTGGTACCACTTATGACTATCAATGTGAGCAAATTGGGCTGTCAAGAAGATGACACATTGCATCCATTGGCTAGATCACTTTATAACTTAATTCAATGAGCTCATAGGTTAACTAGGCTCCTGTGGACAGTAAAGGACATATATGTTCATCACACCTTTCGTTATAAGCTCATAGACCTCAACACTATAGAATCAGAGGTTAGACTCTCTCATGTGGAGGATATGATTGTTTAGGTCATATCCTTTTTAAAACCCTAAGGGCATGTTTGGTTTCTTTTCTATTGTTATTTTATTTATTTTTCTAATTTTGAAATAAATATGACGAGGAGTATAAAAAAACAATCCCCTCCAATGTCACCCTCATTTTCGATTTTTAATGAATCAATTTTTTGAGTAAAGAGAGTTAAAACTTCTTTCTATAAATGCACTGTTTCAAAAGATTGTCTTGAAAGTAGTTTTGCTTCAATGATTTTTCTGTATCGTTTCCCATTTTTTTCTGTTTCTGAAAACCGAAAGTTGTTTTAAAACAAAATCACCTGATTTGGTAATAAGAGCTGGCAAAGGCTTGCAGTTGAATCTGTTCACCAATTAATTGCAGAAATCCATGTACTTGGTCCTCCCATGTGGGGCGTGTGGATCCCTCACTTAGGCAGTATCATTTTTTGTTTGTCGGAAACCTCCTAAAATTGCAGCGACGTGGATGTGAGCTTCATAATTTGTTAATCCAATGCTATGCCGCTGATGTTGCTTTTTCAAGTGTTACTGCAATAACTCTGTGCTTGGCAACCTTTTTTTCTGTTTATTCACCATGCATTTATGATTCAAGATTCCTCGTCGGAAAAAAGTATGGAAGGATGGTGATTCTAAGGTCACCATATTGAATCAACTTGTATTTGGCATTACGGATTTCAGAGACAGCTATGTTGTGTGTGTCTATTCCTTGAGTTAATGTTCAACAACTAGAGACAGTTTCTTGAATCTGAATCTGAGGTCTTTTTGGAAATAATTTGTTAAGTTATGGGGGCAACTGTAATTTATGTATAATTAGTAGATAATTTATATTCACTCATGTAATTAGTTTGTAGTTATGGGTTTATTGTAATATTTTCTTTTTCCTTTTCTTCACTTGTATATATTTTTACGTAATGAGAAGAAAATTTAATTTTCTTTTGCCTCACAATCAAACTTTACTTGGTATCAGAGCGGGTGGTCTTGTGCCCTAACTGATTTATCGATCTCCTTCATCGCGGCACTGTTCGTTGGTACTATTCATCGACACTGTTTGATTTTTTTTGGCTTGAGATATATCTCTTGCAAGTATTATTCTTATTATCTAGCCCCTTAATACTCCTCGGTATCTGAATTTTTGCATTTCGATATGTCTGACGGTTCAAAGATTGTTTCGGCTAAAGTTTCTAGTAATAAGGATGTGTCGAATCCATATGACAGGCAGTTGGAGAATGTTCTTTTGGATATTTGCGGCATTAAATTGAATGGTACCAATTATTTGATTTGGTCTCGCACCTTCACTCTGGCCATTGAAGTAAGAGGGATGTCTGAATTCATTGAGGGGCCTGCTACTTAGTTGGAGGAAGTTGTTACCCTTAAGAAATTTAAGTCTCAAAAATCGTTAGTTATGACATGGTTGTTTAATTCTATGTGAGCTGATATACGTCACACTTTCTTGCTTTTGGATACCACATATAAAATTTGGACCACAGCAAAACAAACTTATTCTCGGCAAGGTAATGATGCCCAGTGTTTTGACTTAAGAAAGAAGCTTTGCACGTTGGACCAACACCATCGATCTGTTGCTGAGTATTTTGCTGAGTTGAGTGGGGTGTGGCAGGAGTTTGACTTTTATCAGGGATTTCAGGTTGTTTGTACTGCTGATACTGCAAGCTAGTTGAAGAGGTTGGAGAATGAACGTGTTTATGATTTTCTTCCTGGCCTTAATGTGGAATATGATCAAATTCAGGTGCAGGTATTAGGTCGTGTTCCATTTTGGTGTTTGGGAGAGGCTTCCGCTATTATGCAGCAGGAGGAGAGCAGGAGAGGTGCTATGTTGCATACTCCCTCTGATAATTCTGCCTTGATTGCCACTCCGTAAGGGGGTAACTTACAGACTGGTACTAGTAATGGGGCTAACAATCATAAGTCACTCAGGTGTGATTATTCCCAGAACACCGGTCATACCAGTGATTTCTGTTGGAAGTTACATGGTCGTCCTCCTCGTGGGTGAGGGAGTGGGCGCAGTGGTCGTGGTCGAGGTACCGTGCGTCCTCAGGCTCAAGCCCATGTTTCAGAGTCTGAAGTTTTTGCTTCTTTGCCTGGTTTTAGGTTCATATCGTCTGAGCAGGTTAGTGGTTTTTCTCAAGGGAAGATGTAAGCTCTCAGGTGTCTGATGGCTCAGGCTGATTATTCATCTATTATTGTTCTCACTTTCACTTCATTTTATTTTGTTCACACAGGTATCTCAGCTAATGCATTTACTGCCTCTTCTAGTGTTCTTTGGATTATCGATTCTGGTACTTCAAATCATATGATAGGTTGTTCCTATATATTTGATTCTTACTTGACATGTTTTGGTAAGGATAATGTCAGAATAGCAAATGGGTCACTCTGCTATTTTAGGAAAATGTTATGTTTGCTTTTCTCTTTCTCTTTATCTCTTATGTGCTTCATATTCTAAATTTTGCTACTAACCTTCTGTCTATTAGTAGCCTTACTCATTCTGGTAAGGATAAGGTCAGAATAGCAGATGGGTCATTCTGTTATTTTAGGAAAATGTTTCGTTCACTTTTCTACTTCTATTTCTATCTTTTGTGCTTCATATTCCAAATTTTGCTACTAACCTTCTGTCCGTTAGTAGCCTTACTCATTTTGCAAATTGTTCTGTAACATTTTTTCCTACCCGTTGTGTTTTTTAGGAACTGGAAGCAGGAAAGGTGATTGGAAGTGGTAAAGCACATGGTGGCCTCTATTTTTTGGAGTCTGCACCTCATTCACTTATGTCATGTGGTCTAAATCTGCAAGCAGATAAAGGTTCAACTATTTCTATGTTACATCAATGGCATCGTAGATTTGGTCATCCCTCCTTTGGGATTTTAGAGAAACTTTTTTCTAATTAGTGAAACATTATTCTAGGAGTTAGTTTTTTTGTGAGACCTGTGAGCTTGGTAAACGTAAATCTTTTTATCTACCTATTAATAAAAAGAGTGATTCTCCATTTATGGTTATTCATTCTGATATTTGGGTTTTTCCTATTACATCTCTTAAAGGCCATCGTTGGTTTGTCACTTTTATTGAATGTTTTTCTCGTGTTACTTGTGTGTATTTACTTAAGTAAAAAAATAAAGTTTTTTCTTATTTTAAGCCTTTTCATGCAATGATTCGTACTCAGTTTGATTCAAATATTAAAATTTTCCATAGTAATAATGGCACCAAGTATATTGACAAGAGTTTTAGAGTTTTTTTGGATGACAATGGTATTCTTTTTCAGACGACTTGTGTTGGTACTCCACAAAAAAATGGAGTTCTTGAACGCAAAAATCGTCATCTTGCTGAGGTAGCTCGCTTTCTTTTGTTTAACATGAATATTCTCAAGTATCTATGAGGTGAAGCAATTTTGACTGTAGCCTACTTTATTAATCGCATGCCGTATGGTGTCCTTAACTTCAAAACATCTATTGAGTGTCTTCCTAGCCATTTTCATGTCACAAATCTTCCCCCACGAGTTTTTGGTTGTGTGTGTTTTGTGCATGCTCTACAACCCTCTGATGGCAAGTTCGATCCTAAGGCTCACAAGTGTGTTTTCATAGGGTACTCCCTTACCCTACCCCAAAAGGGCTATAAATGTTATGATCCTACATCTGGGAAGTTTTTTGTCTCTATGGACGCCACTATTTGAGAAATCGATTTTTTTTTTTTCTCCCTCTACTCCATCTCTTCAGGGGGAGCATAGACAGGAAAAAAAGATGTTTTCTGGTGATGGGAAGAAATTGTTTTATGACCATGGTGAGGGGGCTGGTGATAGGAGGGAAGGAAAAGTCTGGAGAGGAATCAATATCTCCTGAAAGAGCAACGCGTGACATTGGTGGTGATGAGGAGGCAAGTCCCCACCGGTTGCAAAGGTCTGACTTGAAGACCTACGCTCGCAGGGAAAAAGAAAAGTCCTCCTATGTGATACCACTGTGCCAATTACAATTGCTTGCTTTAGTTCTGAACTCTCCAGCTAACTCATCTGGTAATGACTCTTCTTCTATTGAACCTCCTCTCATTAATTATGTTGATCTTTCTATTTTTTCTTCGAAAAGGTGTTGGATCATGTACTCAACATACCATCTCCCAATTTGTCTCGTATAATCGTTTGCCTCCTTCGTATCATGCCTTTGTCTCGTCTCTTTCATCTATTTCTATTCCACAGAATTGGCAAGATGCAATTATGATACCTAAGTGAAAAAGAGCTATAGTAGAGAAGATGATGGTTTTAAAAAAAATGGCACTTGGGAGTTGGTATCACTCCTGAAAGAAAAAAACCAGTGGGGTACAAATGAGTTTTCACCGTTAAGCATAATGCTGATGGTACCGCTGAGCGATATAAAGCGAGACTTGTTGCCAAGGGTTTTACCTAAACCTATGGTATTGACTATGAGGAGATGTTTGCTCCTGTAGCGAAGATGAACTCTGTGCGGGCTCTACTTTCTTGCGCTGCTAATTTGAAATGGCCTCTATATCAGTTTAATGTGAAGAATGCATTCCTTTATAGATAGTTAGAGGAGGAGATGTATATAAATATCTCACGAGGTTTTTTTTCATCTAAGACTGAGGGAAATGTGTGCATATTGAAGAAGGCTCTTTATAGGTTGAAGCAATCACCTTAGAGCTTGGTTTGGTAGATTTTCAAAGGTAATGTTGGATTTTGGATACAAATAAAGTCATGCAGATCATACTATGTTCATCAAGAAAGGTGCAGGTAAAATTGTTGTTCTTATTATGTATGTTGATGATATGATAGTGACAGAGGATGATGTGGATGAGATTCTCAATCTTAAATCTCGTTCAACTCAAGAATTCGAGATTAAAGATTTGGGGTCGCTGAGATATTTTCTTGGGATGAAAGTGGCACAGTCTGATAGAGATATTTTTATCTCCTAACGAAAGTATATTCTTGATCTTTTGGAAGAAACATGTATGTTGGGTTGCAGGCCTGTAAATTCTTTTATTGAGGCGAATCATCATCTTAGTGGAGACATGGGGGAACGCACCACCAAGGAGAGGTATCAGCGATTGGTAGGTCGGCTAATTTACTTAGCTCATACGAGACTAGATGTTTCTTATGCAGTGGGCGTTGTCAGTCAGTTTATGCATGATCCTCGTACTTCACATCTGTATGCAATTTATCGTATTTTGAGGTATCTCAAATTAACTCCAGAGAAAGGGATTTTGTTTTCTAATCACAATCACTTGTAACTGGAGGTATTCACTGATGTAGACTGGGTTGGTTCTGTGGATGATAGATGCTCTACATTTGGCTATTGTACTTTTCTTGGTGGAAATTTGGTTACTTGGCATAGTAAGAAGCAATCGATGGTTGCTAGATCTAGCGCCGAAGCTGAGTATAGAGCTATGGCTCATGGTATTTGTGAGCTCTTGTGGTTCCAAATCTTGTTGCATAATTTGGGTGTTTTGATTGATGGCCCTATGAAATTTTACTGCGACAATAAAGCTGCTGTAAATATTGCTAACAAGCATGTTCAACACGATAGAACGAAGCATGTAGAGATTGATAGACACTTCATCAAGGAAAAGTTGAGCGAGGGGTTGATCTGTATGCCTTTTGTGAAATCCGAGAATCAATTGGCCGACATTTTCACAAAAAGGCTAGGCAACAAGAGTTTCAACCCCATTGTGTTCAAGTTGAGCATGATCGATATTTTTACACCAACTTGAGGGGGAGGGTTAAGTTATGGGTGTAAATATAATTTATGTATAATTAGTGGGTTATTTATATTCACTCATGTAATTAGTTTGTAGTTATAGGTTTATTATAATTTTTCATTTTTTCTTTCTTCACTTGTATATATTATTTTTACGTAATGAGAAGAAAACCTTTTTTTTTTGCCTCACAATCAAATTTTACATAATTTTCATAATCTTGATATGACTTAAAACCTAGGAAGCATGGATACGGGATACAGGTACAATACGACATGGGTACGACGGCACGCCTAAATTTGTGCTATTCTTTAAATGTTTTGTAGTTAAAACATTTATCAAGAGGAAAGTTATTCCTGTTTGTATAGCTATGAATATAAGACACAAATGTGGCCTTAAAAATATGTAAGTATGAATTGAAAACATAAGGTACTTTAAATTTGCTATTTTTATCGTTCCTATAAATAAACCACATATGGTGCAACTATTCTTGTATTTATCCTCTGAACTATAGTCTAATATTGTGCAAAAAATGCTATAATCTTAATTTGCTCATACTTAGGAAATGTGATCGAGAGAATCCGATACTTCTACGAAACTAATGACACATTGACAATAAATTTTAAAATACGGATATGTTTAGGAAGCATTAGATATACATTTGCAATTATCGAAAGAGTATTGGTATCCGATGTAGCTACGATATGGGTACAAACCCCTCCTAGAAGTATCCTTGATTCATAACTTAAAACTTGTTATTTGACTGAGCTGTTTCTTTGAGTTTTTTATGAAACTTTGTTACTGATAACCATTTCTATTCAGGTATCTCTATCTCCGGTGGGGCGTGGACTTATCAAAAATGGTGGTTTTTGTCGGAGAAACTGGCGACACTGATTACGAGGGCTTGCTCGGTGGCATACACAAGTCTGTAATATTGAAGGGAGTTTGTAGCGGTCCGACCCATCAACTCCATGCTAACAGAACCTACCCTCTTTCCGACGTCCTGCCGATCGACAGCCCCAACATTGTTCAGGCAGCTGAGAAATGCAGCGGTGCCGACCTACGAACCTCGTTGGGGAAGCTAGGGTTCATCAAGTGCTAG

>AcSPS3

ATGCCTGGAGTTTATCGGGTTGATTTGCTGACAAGACAGGTATCAGCACCAGATGTCGATTGGAGTTATGGTGAACCAACAGAGATGCTAAATACAAGGAGCTCTGAAAATGATATGAAAGAAACTGGGGAAAGTAGCGGTGCTTATATAATTCGTATACCATTTGGTCCGAAAGATAAATACATTCCCAAAGAGTTCCTTTGGCCTCACATTTCTGAATTTGTGGATGGTGCACTTAGTCATGTTATACAAATGTCCAAAGTTCTTGGTGAGCAAATTGGTGGCGGTGAACCAGTCTGGCCTGTTGCCATCCATGGACATTATGCAGATGCAGGTGACTCTGCCTCTCTTCTTTCTGGGGCTCTAAACATCCCAATGCTTATTACTGGCCACTCGCTTGGACGAGACAAGCTTGAACAAATCTTGCAACAAGGACGGCAATCTAAGGAAGAAATAAATGCTACATACAGAATAATGCGGCGTATAGAGGCCGAGGAAATATCTCTTGATGCTTCCGAAGTTGTTATTACCAGCACTAGACAGGAAATAGAAGAGCAATGGCGTCTGTATGATGGATTTGATCCCATAATAGAGCGCAAACTAAGAGCAAGAATCAAGCGCAATGTGAGCTGCCATGGAAGGTTTATGCCTCGCATGGTTGTAAGTACCAAGATTGTCTTCCCATTAAAACCAATAAATCTCCTTTTAGCAGCTGAATCAAGTTTTATTTGATTATTGTTGGTTTTGCCTAGGTTGTTTCGTCATTCACTACTGATATTTGTGTGTTCATATTTTTCGTGATGGTATTAGGTGATTCCTCCTGGAATGGAGTTCCATCATATTGTTCCACATGATTCCGATATGGACCGAGAAACAGAAGGAAACGAGGATAATCCTGCTACTCCGGATCCACCGATTTGGTCTGAGGTGTTAACTATTCCTTGTCGCTATCTCTCCGCTCAACGAACACCTTGTTTGTCTAATGAAGGGCTCCCTTATATTTTTTACCGGGGTAATTTTCTATTTTTGCAGATTATGCGTTTCTTCACCAATCCTCGCAAGCCCATGATACTTGCACTTGCCAGGCCAGACCCCAAAAAGAACATCACGAATTTAGTCAAAGCTTTTGGAGAATGCAAGCCATTGAGGGAACTTGCTAACCTTGTATGTTCAAGCTTTTATTTGCACTTGATGATTAATCATAAATGATTCCTCTGAGAAGTTGAAAAGGCTTAGTCATTGATGTACTTTTTTTCCAGATCACTGACAATATCTCTGCACTTCTGTTGTTGCAGACATTAATAATGGGTAACCGTGATGATGTCGATGAAATGTCTGGCACAAATGCGTCTGTTCTCCTTTCAATACTTAAATTGATTGACAAATACGATCTGTATGGTCAAGTGGCATATCCTAAGCACCACAAGCAGTACGAAGTTCCTGACATATATCGACTTGCAGCAAAATCGAAGGTATGGGTTTGAAATCTCAAGGGAATTGCAATGAGAGAAGGTCATTCTGTTGCATTACCATGTATCAAAACAATGAAGCATTTTAGATCAGCTTAGGCATTGTGTCAAGGAAGAAGTTTTATGGTTTTGAGAGCGTTGTATGATGATGTCCTCTAAATTCTATTCTTTTCCCCTTTATTTTTTTCCATGAACTTTGATCCTCTGTTTCCCTCTGTGCTTTATTTTGTTTGGATTGTTTACACACAGGAAAGGATTGGGAATATGAAATGTAGATGATATTAGAGAAATTTTGATTAATACGATTTTTTCCCCGCATAATAGAACCAATATTTGCCCGTTACATGGCGTGACTGGTCATGCTAATGACATGACAACTTCACTCCATTGGCAAACGTTATACACCCACACACACACCATTCGAGTTTTGTATCTTACCCAAGAAGAAAAAAGGCATAAACATTCTGTTCTAAGTTAAATGTTTCAAGACCAAGTCACCAAGTGGCTTATTCTGGTCTGACAGGTTTCCATAACCTTTTCATTTTGAAACTTGTCTGATATATTTGAATTTGGTCGATAGGATTCTCTAATTTCTTGACCACTTACAAAAGAATTCTGCATTATTAGGTTATAATGTTGATTCCATGCAAACTGTGAATGTGTTTAGGGGGAAATATAGCTGGTTATTGGGCTGTTTAAGAATGAGAAACACTACCCTTATCTTCAAGCCCTAATCGTATCAGTAGCTGTAAGTGCCAACATATTTAAGGAATATGATTGGAGAAAGTGAGTTGCTGCAATTACCAGATCATGTTTCTTTTTCTTATCTTCTTCTTTACACAAAATGTCAATTGAGGTGGTATTACTGTATTTTCTATCAAGTTTGACTTCTCATTCTATCGAACTTCTTCTTTCTTAGGGTGTCTTCATTAATGCAGCATTCATTGAGCCATTTGGGCTCACTCTTATTGAGGTGCTTTCTCTTTTCTATTGTTTGCAAAGCTTTTTATTTTTTTGTACATGTTCAATCATTAGCTAATCTTATGTCAACTCTTTTGCTGAACAGGCAGCAGCTCACGGTTTGCCTACGGTTGCCACAAAAAATGGAGGTCCGGTTGATATCAATCGGGTGTGTTCTTTGTTGGAACCTTCTTATTGCAAGGTGAACTATCATCCTAGTTTCCAAATTTCCAATACTGAACATAAGCTTATTTCAGGTTCTCGATAATGGTCTGCTAGTTGACCCTCATAATCAGCAGTCTATTGCCGATGCTCTTTTGAAACTTGTTGCAGATAAGCAACTTTGGGCAAAATGTAGACATAACGGATTAAAAAACATTCACCTTTTCTCGTGGCCAGAGCATTGTAAGACGTATTTATCTCGAATTGCCAGCTGCAAACCAAGGCAGCCACGGTGGCAAAAAAGTGACGATGGGTATGAGAAGTCAGACTCGGACTCACCGGGTGATTCGTTGAGGGACATACAGGATCTATCTCTAAACTTAAAGATTTCTCTGGACGGGGATAAGGGAAGTGGCACGCTTGATAATGCCTTAGATTGTGAAGAGAATGCGTCTGGTGGAAAGAACCGATTAGAGAATGCAGTTTCGGTATTATCAAAGGGTGAAGAACAAAACCCACGAAAGGCTGGCTCCATGCCGAAACTAGACTACAACAATAGTAAATTTCTAACATTGAGGAGGCGGAAGTTCGTCTTTGTCATTGCTGTGGATTGTGATATGACTTCCGAGTATCTTAAAATGGTCAAAGTGATTGTTGAGGCTGCGGGGGAGAATAAGTCAGGCTTTATAGGCTTCATATTGTCGACAGCCTCGAGCATACCGGAGATATTCTCTTGTCTGAAGTCAGGAGGCTTGAATCCTATGGACTTCGATGCTTTTATTTGCAATAGCGGTAGTGAACTCTACTATCCATCTTCAACTTCTGAAGTTGGTCCTTTTGGGCTTCCAGTCGTAGCAGACTCAGACTATCATTCCCACATTGATTACCGTTGGGGAGGAGAAGGTTTGAGGAAAACTTTGGTCCGCTGGGCTGCTTCTATGAATGACAAAAAAGGAGCAGGAGGAGGACAAGTTGCTGAAGACGAATCCAGATCGGCTACGCATTGCTGTGCATTCAAAGTGACGAACCCAGCAATGGTAAATTTTCTGTACGTTTTGTTTGGAGAAACATTACGGTCCCGTAATTTGAACCAAGAAAAGTTTCTAAAATTTAAAGTGCATTATTCAGATTCACTTGAGTGTGTGGATCCCACATAATCAAGTGAATATGAGCCATATATGAACCATTCATTTTGAATTTTGGGAACTTTTTTGAGCCCAAATTCTGGGACCATAGCATGCTCTTTTGTTTGTGGTAGTGCTTCCTCGCTCCACTTATCAACGTTTTGAACTTTTACTTCTCTTGTCAATTGACGTATTGGTTGCAGTGGGATTGTATTGTTTTGATTATTATTTTGTTTTTTTAGCTTCCTCCTGTTAAGGAACTTCGGAAATTGATGCGGATTCAGGGTCTTCGATGCCACATTATTTATTGTCAGAATGGTATAAAGATGAATGTGATTCCCGTGGTAGCTTCCCGATCCCAGGCCCTCAGGTATTTTCTGGTTATTTTCTTTATGTGCCTGGTTGGTTTTTTCCCCTATTCTCGTGGACGGTCGAGTTAAATTCACCGATAAATCCAGGTGTTTCTATTTTCGTTTGGTTGATATGTCAATCACCGTTTTGTACTTGTTCCTTCTAAGCATCTGAAGTAGTGTTTCCCACCGTGGATTTGGACCTTCCAGTCTGTTGAAATGAAATGTTACTAGAAAATGCTTAGGTATTCCATGCATTTTTATGCATTCACGTGTTAATGGAAGAAGACCGATATGTGATTTCAGTTGCACTAGGTGAATGTTAAGGTTCAGCAGTTGTGTTTACAACGGAAACTACTATATGTGTGGGATGTCTCATTTATACACTTTAACCGGTCATGAGACTTATGTATGACCTCATTTCCAGGTGCATTCAGACTTTTACACAACATAGTTAAGTGTCTAAATTTACTGAGTACAATGAAACTAAATTCTCACTGAAAACTTTTTGGAAACAAATAAGATTTCTGTACTCCATTGTAGAAAATGGTATGTTTTTTACATTTCTATGCTTCTAAAAACAAATATGCTAATAGTTGGATCATTTTTGGAATCATTGTATCAATGTTTATAAATGCGCAACATAAGATGGTAAATAATTAAAAATTGATAAAAGCAAATGGTCAGTATAACATTGTAAAAACAAAAATGATCTCTTTCATTTCTGTTTCGTTTGAATGCATCGATCTTTCTTGCACTTGATTCGGATTTCCACATTTCTGTTTTCGTTTTAACAAAACCCATCTACTTTCTTCCAGGTACCTGTATGTTCGATGGGGCGTGGACTTGTCGAGTATGGTGGTCTTCGTAGGAGAATGCGGGGACACGGATTACGAAGGATTGCTCGGTGGAGTACACAAAACTGTGATACTAAAAGGTGTGTGTGTCGATGCACGTAAACTACATACTAACAGAAACTATCCTCTCGAACACGTCGTGCCGTCCGACAGCCCTAATCTTGTGGAGTCTGAAGGTTGCAACAACACCAACATTAGAGCAACATTGGTAAAGCTAGGTGTTGTGAAGGGCTAAGGTGTGTCTGGGTCTTGCATTTGCGAAGAGCTTTGTGGAGGGAAAAAAAAAGAGATTCGAATTATCCGAAATTTTCTTTATCTTTTTCCCTTCACAAATTTCTCCCTAGATTCAAGATACAAATGCAGCCTAAAAGTTACAGTTTTAGAATTCTTTTTATCTAGATTTGTCCTGCTTTCCTTATATTCCACGAGATCCTAACCTTAGGGCTACGGCTAAGGTTAGGATTAGATTTGGCTTATGTGTCATGGGTTTGGCCGAGCCGGATGGTTTTATGTATTGGTTTAGTCGTGTCCACGTGTCACCCATGTGGCTCCCACATGTAATATTCTGTTGTTAATTTGGAGGGTATTAATGACTGGTCCTAATTACAACCAATTCAAGAGTTGAAAGTCTAAAAAAAAAAATTTTGTTTGTTTAAGGACCAAAGTGATAAAATTTATCAACCACATAAATCTTCAAATTGCATTGCACACGCTCAAGTTAATCTAAATCATTCATTTTGAATTATGAGGGAAAAAAAATGAATCCAAATCTGGAACCATAAAGTTCTCAACTAGTTTTAATCAAGTCGTTCATGAGAAGAGTTCAACTAAGTTAGTTTTTTATATAGTGATAAGGTTTTTCAAGCTAAACTCAGAAAATTAATGAGATTCAAAATATGTTGAAGTTTTATTTATTTATTTATTTATTTTTACGTATTGAGGCAATCTCCACCTTCTAATAATAATAACAACAACTTCCACATTAGGAAATGAAGTCTACATGGATGGTTATTTATTTATTTCCTTTACAAAATAAATTAAAATTAAAAACAAAATTGTGCTTTTAATTTTTGTACATTTCTATTCACTGTCCCTATCTCATGCGACATGCTAGGCGCCAGATGTGTCCACTTCCAACCCCAAGCTATTCAAAAACTTAACCAGGGTTCTTTGCAATGCCAACACTTCAGAAGGTTTCCAACCACCGAGAGATGTCTCCTTGCGGGAAATAAAAATCCCACACAACCACCTTGGGCCACCCCGCCTTGGCCAGCCTCCAAACAACCACTCCATCCTCGCCTTCTTTGCAGGCGGTGACCACAATCACGCACGAAAATCCTACTCAGATACTGGAAAGACAAAGATAACAATATTCAAGTCCACGACTACCTCCCAAAACCCTAAATTACTTCGAGCTAATGGGTCGAGTCAAGTTGTGCTTATGTGCTAG

>AcSPS4

ATGGCCGGGAACGAGTGGATAAATGGGTACTTGGAGGCGATTCTTGACAGCGGCGCGGCGGCGATTGAGGATCAGAGCAAGCCAATCCCCGTGAATCTTAGAGAGAGAGGGAATTTCAATCCCACCAAGTATTTTGTCGAAGAGGTTGTTACTGGGGTCGATGAGACCGATCTTCACAGAACATGGATCAAGGTGGTTGCGACTCGCAATACTCGCGAGAGGAGCTCCAGATTGGAGAACATGTGTTGGAGGATTTGGCATCTCACTCGCAAGAAGAAGCAGGTTTCAAGATTTCATTCTCTCTCTCTCTCCATCTATGTGTATATATATATATATATATATATGAAATGTCTATTTGTTTGTGTATGTGGGTATGCGTGCTTGTAGGTAGAAATGGTGTTTCTTATTTGGATTTTTGGAGAATTGGGTTTTTCTTGAATTCTGAATTATCTCCCCCCACACCCCACACCCACACCCACACACCCCACACACACACACCCCAACACACACACACCTCTCTCTATGAAATGTCTATTTGTTTGTGTATGCGGGGTATGTGTGAAAATATGCAGAACTGGTGTTTGATATGGAGATTCTTGGTTTTTTCAAGAACTGGGTGTTTCTTATAATCCATGTTTGTTTAAGTTCGTGCGTAGAATTGGCTGATTGTCAATTGGTGAATATGTACTTGTTTGTGGGTATGTGAGCTTGTATGTAGACCTGGTGTTTGTCATTGGGTTTCTTGGATTTATTGATAATTGGGTTGTTTTTACTTTGTTATTAGTATAAATTGTGTGGAATTTGGTTGATTAACAATGGATTTCTTGAATATTAGAGTCTATATACATGCATATTTTTTTCTCTGTGTGGTTGTTAACTTGTTATTGAGCTTCTTTAAGATTTCAAGAATTGGCTTCTCTGTACATCAGTATTAGTATAAGTTTTTGTGTGGAATTGGGTGATTATCAGTGGGTTTCTTGAATTTTAGACCATTCTCTTCCTCTTTTTTTGCATATAATATTATATATATGTGTGTGTGTGTGTGTGTGTCTCTCTCTCTCTCTCTAGGTATAGGGGCGCCTATGTATGTTTAAGCCTCTTTGTGTAAGGGAAAAAAAAAAGACTGGAGAAACTTCTAGGCCATTTTTAGATAAAAATTTATAGAGAGATTTGTGGAGAGAAAATAAAAGGTAAGAGAAACACGTCTCAGACTTGGTGAGAGAAATCTGTTGCATAATCTTTTCCCCCCTAATGCTACTACCACACACACACACATTTGTATAATCTTCTTTCATGGTTGTGTAGGACCCACATAGTCATAATGTGCAGGCTCCACACGTATGTATATGTGGGTGTGCAAATGTTTTTGTTTGTAACGTTTCTCTTCTTTTCCCTCCACGCTTGATCCTGAAAGAGCTTTAAGGGATTTTTTTATTTTTTGACTCTCTCTCTCTCTCTCTCTCTCTCTCTCTGAGATTGTTGACTACTGATGGTGTTTAGTCAAATTACAGAGAATTGGAAAGCCTTAATGGGATTCAGTGCACTTGTGTGAGTTTGTGAGTGTGTGTTTACACATTTAATTAGGTGGTGACTTATAGTGGGGTTTTCATGACTTTTCCTAAGAATTTGACAGTTGAAGGGTCTCTATCACCATATCTGGAGAATTAGGTTCATAGTGAATACATGACTGCCTTGATATCCCTATTAAAGATGAATGAATACGCTTGCTCTATTAGGTATATTTTTTATGGGTTAATATGAATAAGCTTTTTTCTAACAATTTTTCACAATAAACTCTTTTAGCTTTATCCACCAACCCTCAAGGAAAGATCTTTCTTTGATGAAAACTTTCTTTATAGTTAGACTTCATTGAAACTATTTCTATAAACACGTCCTAAAGGAAAAGGCACAATTCAATTCAGCAGTTGCCGCCAATAGTCTAGAAGGATTCACACTATCATACAAGCTACCAGGAACTTTCAAAATTAAGAAGGTTTGACTTTGAATTAGAAAAACAAAAACCTTTTAGTTTTAGAAGTTCCTCCACGTCACACTTTGTTCATTTTATTTTAAGAAATTGATATTTCTTTTTTCCGAATTGTCGTGCACCCCGCAGATGATGTGGCAGATGCTCAGGTTGAATCCATAAACATGTAAATTGTATAGAATTTTTCATCTCATTTGATGATTGAGGCAATTGCCTAGTATAATATAATATGGAGGTTCTTAGGTGTTAAGAAGTTTACACGTATATAAATATGATTTGGTACTTCCCTCACATGCCACATGAATAACAAAAAATCAAAACCAGGAATGATATTTGATTTGATCAAAACATATTTGATCTGAAACTTGTGTATGCTTTTAAGAGAAAATGCTATAAAAAAAAAATGTTTCTAACATGTATTCCAAATGATTCCATGACTAAATAGTGGAGCTTTTTTTGGTTTTATGTTAGTTGGAATGGGAGGATTTCCAAAGGATGGCACACCGAAGATGGGAACGTGAACAAGGACGCAGGGATGCAACAGAAGACATGTCCGAAGACTTGTCTGAAGGAGAGAAGGGAGATGTACTTGGAGAAACAATGGTGAATGAAAGTCCGAGGAAAAAGTTCCAGCGTAACTTTTCCAACTTGGAAGTATGGTCGGATAATAACAAGGAGAAGAAGCTTTACATTGTTCTTATCAGGTACAGTTCTTTATGTATGTTCATCACCGTATTGGATTTGAACCACTCTTTGGATATCAGTCACAGTCACACTACTGATCTATTAATATTGAAAGAATATCAAGTTGACAAGATTTTAGGCAGCTTCCTTTTAATCATTATTAGAAAGCTGTCTCTGAGTTTAACAATTTTATTCAGCATCCCAAGTCTTATTTACCTAATCTGTAGAGAAATTGACTTCACATATAATGGTTTAAGCATTCACTGAAACGATATCTCTTATGCTTGCTTTTGTTGCACAGCGCAGACTTTCCAAATATTGTAATAATTCTAGGTCATCTAAAAATGACAAGGGTTCTCACTTCTCATCTTTATGAGGTGGGTCTTTTTAGATAGCACAAAAGACTAACTTATTTACGCAAAGTTTCTTGTTAGTCAATTCAACGGTGGCACAGGTCAATTGATAACGTAAAGCATAGAAATATACAAGTTTAGGAAGAAATTCATTGATGCAAAGAATACATTTTTTAAGCCACTTGTTTTAAGCTAGTTACGGAATTAAATAGAATGAATTTTAGAACTCACAATCACAATTGATCTTCTTGACCAGTTTGCACGGTTTGGTCCGTGGGGAAAATATGGAGCTTGGGCGTGATTCCGATACTGGTGGTCAGGTAAATACTTAATTTTTCTTTAAACGAACAATCCTTTAACTATATTTTGTTCTTCTTGTTTGCTTTTTTATCTAGTATCTCCTGTTTTCATGACAATTCCAACTCCAGATCAAATATGTGGTAGAACTTGCTCGGGCCCTGGCTAGGATGCCGGGGGTATATAGGGTCGATCTGTTTACCCGCCAAATCTCTTCTCCCGAAGTCGATTGGAGCTATGGAGAGCCCACAGAGATGCTCACCGCAGGTGCCGATGACGATGCTGATGTTGAAGAAAGCAGCGGGGCTTACATTGTAAGGATACCCTTTGGTCCACGTGATAAGTACATGAGAAAAGAATTGTTGTGGCCCTATATTCAAGAATTTGTAGATGGAGCTTTGGCTCACATTCTCAATATGTCAAAGGCTTTGGGTGAACAAATTGGTGGGGGGCAGCCTGTTTGGCCATACGTAATTCATGGCCACTAAGCAGACGCGGGGGATAGTGCTGCTCTTCTTTCAGGTGCTTTGAATGTTCCTATGGTTTTAACAGGACATTCACTAGGTAGAAACAAGTTAGAACAGCTTCTCAAGCAAGGAAGGCAATCAAAAGAGGATATCAATTCTACGTATAAGATTATGAGGAGGATAGAGGCGGAAGAGCTTTCCCTTGATGTTGCAGAGCTTGTTATCACGAGCACCAAGCAGGAGATTGACGAGCAATGGGGGCTTTATGACGGGTTTGATGTCAAGCTTGAGAAAGTTTTGCGGGCACGTGTTAGACGAGGGGTCAATTGCCATGGACGATACATGCCAAGGATGGCGGTAAGGAGCTGTTTTGAACATCTTATTGATCTACTTGTAGAGAAAAATTGGAATGTTGGAAATTTGAGAAAAAATCATTTTGTTTTGGAACGTGAAGACCCCATCAGATCCTTAATGAACTTCACTTTCGAATTGCCTTTCCCAACAGACGTAATATAATAACATATTCTACAACTCCCCTGTCACCTTTACATTCTTGAGAAGCAAACTCTCTTGTAAGTATGATCTTGGTTTCTTATTTTGTCTGCTATCAGTGTGTAAAGGTCTATTGAAGCTAATTTTCATTGGTTATATCTTTGTGGGGAGTGAGTTCTATTCTGTTTATCCGTGAGAGGGCCCCATTTCATATGCACATATATTAGAAGTAATATTAAAACTAGAAAGCTCTTTTGATTTGAACAATTATGTCAGCATGAAGGCCTGAGAGAAACTGAGTTTCCTTTCAATTCAAAAACCGCCTTACATGTTGTTTTCCTAGACTAAATTCTAATTTTTCCCAGATCATCAAGTCATAACGGTAATTTGAAACTATGAATATCCTACCAGATTTTTTAATAAGAGATTCGTATAGTATTGATTTGAATAACAACGTGCAAGTGAACAATGTGTCATCTGCCAACAATGCTTATTGTGGGTAATCCTGATTGAATTACATTGATCTTTCAATGTCCAGAATAGCCCTTTGTAACGAAATACATTTACTTTTCAGGTTATTCCTCCAGGTATGGACTTCAGCAATGTTGTGGTTCAAGAGGATACCGCTGAAGCTGACGGGGAGCTGACAGCACTAACTACTGCAGACGGGTCTTCTCCAAAAGCTTTCCCACCAATATGGTCAGAAGTGAGTATCTAATTTCTATGCTTCCTCGTCAATACCCATCCTGCTACTTTTTGTAACTACCCAACTGAACTTTCTTTGCAGATGATGCGTTTTCTAACAAATCCCCATAAACCAATGATCTTGGCCTTATCAAGACCTGATCCGAAAAAGAATATCACCACTCTTTTGAAAGCATTTGGAGAATGCCGCCCGTTGAGAGAACTTGCTAATCTGGTAAGTAAGTTCTCACGTTTTCTCGTTGGACCACCAGAGAGTGTAATCAAATGCCTCATGTCTCCTAACATTGTGCGCAGACGCTTATAATGGGAAATAGGGACGATATTGATGAGATGTCCGCTGGGAACGCTAGTGTCCTCACCACAGTTCTGAAACTGGTTGACAAATATGATCTGTACGGGCAAGTGGCTTTCCCAAAGCATCACAAGCAAAGTGATGTTCCGGAAATCTACCGCCTTGCTGGGAAAACTAAGGTTCGTCGAAGTTCACAGTTTTCATACTTGCTTTACATGGTTTCATTTGTGTTGGTTTTAGTAATTCCTGCAACTATTGCAGGGGGTCTTCATAAATCCAGCTTTGGTTGAGCCTTTTGGGCTTACCCTAATTGAGGTTGGTTTGAACTATTTAGGTTTTTTTCATCTTTTATAGTCTTATCTTAACTTGGTCTTGAGAAGGTGGTTCAAAAGCCATTATTTTTATGATGTAGGCTGCAGCACATGGACTTCCAATGGTGGCAACTAAAAATGGTGGTCCAGTTGACATTCATCGGGTAAGAATATCTATGCTCCACGCCCACTAATTTCTATTTGTTAAAAATGTGGAATTGTGTTTCACTCTGTTTGATTGTCAAGAAGACAAAAGGGGAGGTAAACTCTTAATAGTTGATGGCAGTTATTGGGGGTCAAAATGGATTTTCTACATTTTTCATTGTATCCTTCGCAAGTCCCAACTCATTTGAGTAACATACAAACAATGATTTGATTCCCTAAAAGTGCTCTTAGTGTTTCTTTTCGTTGTTTCAGGCTTTGAATAATGGTCTGCTTGTCGACCCACATGATCAGGAAGCAATTGCTAATGCGCTGCTTAAACTAGTGTCAGAGAAGAATTTATGGCATGATTGCAGAAAGAATGGTTGGAAGAACATACACTTATTTTCATGGCCCGAACACTGCCGCACATACTTGACTAGGGTGGCAGCATGTAGAATGCGACACCCTCAGTGGCAAACTGACACCCCGGCAGATGAATTTGCCGCTGAAGAGTCCCTAAATGACTCACTCAAGGACGTGCAAGACATGTCCCTGAGGCTGTCAATTGATGGGGAAAGGACATCACTTAATGAATCGCTTGACTATGTAACTGCAACTGGTGGTGGCCCTGAGCTCCAAGACCAAGTGAAACAGGTACAAGGATGGAGCCAAATATTATAGTAGGTTTATTTTTTTTTAAATTTAGTACTTGTCCCCTTAATGACATTTTTTTCCCCAGTTTTATCGATTGTATGTTCATTGTTTTCCGAAATAAAATCTGTGTCCCCTTGAACAGCTACTAAGCAGGATGAGGAAACCAGAAACAAAAGCTCAAGATTCCGAAGGCAGTGGGAAACTTGTTGATAATATTGCAAGCAAATATCCGATGTTGAGAAGGAGGCGTCGGTTGATTGTCATAGCACTTGATTGCTATGACAGCGATGGAGCCCCCGAGAAAAAGATGATTCGGATAATTCAAGAGATATTTAGGGCTGTTAATGTAGTCTCTCAAACTGCTAGGTTCTCGGGATTTGCTCTATCGACAGCTATGTCAATGTCGGAGCTGAAAGCCTTCTTGAAAACTGGGAATATTCAAGTAAATGAGTTCGATGCTTTGATTTGTAGCAGTGGGAGTGAAGTCTACTACCCGGGTACTTATACACAAGAAGACGGGAAGCTTTATCCTGACCCAGACTATGCCACACATATTGACTACCGTTGGGGTTGTGATGGCTTGAAAAAGACTATTTGGAAGCTGATGAATTCACATGAAGGCGGGTCTTCTCATTCTAAAAGCCCAATTGAAGCAGATGTGAAATCTAGTAATTCGCATTGCGTCTCCTACTTGATAAAGGATCTCAGTAAGGTGCGCAAAAGTTTTGGATGCAAGCTTTGAAGCATAAGAAGTAACTGTTAGTTCGGTTACGTTATATTCATGATGGTTGGATTTTGCAGGCAAAGAAAGTGGATGATATGAGGCAGAAGCTGAGGATGCGGGGTCTCCGTTGCCATCTGATGTACTGCAGAAACTCAACAAGAATGCAAGTTGTCCCTCTTCTTGCATCTCGAGCACAAGCACTGAGGTAATATCTCATAAACTAATGGGATTTTTGGACGCCCCTATGAACTTGGGGTTTAGAGTGTGCCAATTTGAGGTTCTCCAGCTTGCAATGTTTCTCAGATCTTTCTTAGGAGATCACTTTTGGAAATCATGCGACTGCTAGTTCTAAGCAAATTGGGTTCACTTGATTGAATTAAAGTGTTTTCTTCTTTAAACACACTGTTATGGTATCTAAAGAGACATAATGTACTCGGTCCATTCTTTTAAGTCTTGTTCACGCTTAAAAAGTACTGGACCCTCTCTCCCCAAAGCCTTTTGTCCCAATCCACTTCCAACAACACAATCTTACCTTGAGATCTGTGTGTTTCGAGTTTTTCACCCATCTTGCGACCGCCAGTGCATTGGGACATGGGACAGAGTTTTTAGAGAGAAGAGGATTGCTAGCCCACTTGAAATGCTTTCAATCCATTTTCCATTGCATTTGAAACCATCTCTTTGATTGCAACGTGGAATCCTTTTGCCTACTCGTTTTCTTTTCGTAGTCTGTCTTTTTCTCTCTCATTAGTAGTGTGATGTAACGAAGGAATTTATTATTGATTGGATTGTCTAATGAGTTCTCTCTTCGTGGTCGTAGCTCTTATCTAGCATGATTTGTCTTTCTGATTGCCTCGTGTTTTCTCATCTTCTGGTATCATTAGGTACCTTTTTGTTCGTTGGAGACTAAACGTATCTAACATGTATGTGATTCTCGGTGAAACTGGAGACACTGATTATGAGGAACTGATATCTGGCACCCATAAGACGCTAATCATGAAAAACATGGTGGAGAAAGGTTCCGAAGAGCTGCTCAGAGCAGCAGGAAGCTATCTAAAAGATGACATTGTCCCCGAGGAGAGCCCACTGGTGACCTACACAAGTGGAGAAGCGAAAGCCGATGACATTGCAAATGCTTTGAAGCAAATCTCTAAATCTAGCCCGGGAATATGA

>AcSPS5

ATGGCGAATAACGAGTGGATAAACGGGTACTTGGAGGCGATTTTGGACGCGGGGAGTCGGAGAAGCGGATTGAGAGAGAATGGCTATGCTGATGAAGGAATTAGAAGCAACAATAGCATGAACAACATGAGTATAAGGAAAAGGCTTGAAGACAAGTTGAGAATCGAAAAGTTCGACGATGACAAAGGAAAGGAAGAGAAGCTGTTTAGTCCCACCAAGTATTTCGTTGAGGAAGTTGTTAATTGCTTTGATGAGTCTGACCTCCACAGGACATGGATCAAGGTATGTTTCTAAGCAATCCTCCCATATATAGGAACATATATGTGTCTATATATACACACATATATGTGTGTATATATATATATATATATTAACGGAAAATTCATACGTGACTCATGTGTAATATCTGTAACAGTTATTGTGAACGTGGTTGTGAAGCAGTTACCAGAATAGAATAATTTTTTAGTTTTACCAAATGAGGTATTTTCTTTAAATAATAGAATATATAAAAGAGAGATCAAGATTAAACACAAACCAAATGTGAAAAGGGAAAAAAGGAAGGGAAAAAAAACTTCTAATCAAAATGGAACTTGAACTTTGAAAAATAGAAATAGTTCATAATTTTTTCACCTTTCAACTTTATTAGCAACTTCTTTTTTAGGTATATATATTTCCACTTCCTACAACAAAAAAGCTTTTCTTTGCTTTCTCTAGAAATTGCCTTATTAATTTCCTTCAATTCTTCTCCACAGAAGAAAATAAAACAAATAATTAAAATGAATTAGTCAAGAAAATAAAAGAGAGAGTGTATTGTGTGGAATGGGGCTCCCTCCAAGTGGAAAAACTTTGAATTGATGCTCAAAACCATTGCCATGCACAAGAAAAACAATATGACACTAAGGATTTAGAGATTGATTTTTGGAGAGAGAAGAAAATTAAAGAAAAAAAATTAAATATAGACTGTTGAATCCATTAAACTTTTCTCTTTTCTCGTTAGCCTCATTTTTTCCGTACACCTACCAAGGAACTAGTAACTTCTTACACCCACCTATATAGCAACAATAACCTCTTGTTCTTTGCACACGCACCTTCTTCTCCACGGCCGACATCACCATAACTCGTCGCCTCCTTAGTCACTTGTCCGACAACCTCGAATCCCTTCCCTACACACACCCAAAACCACGATTACCCCGTCATCTGAACCTTTCATTGGTTAGTTTTTCGACGCTACAAATATGGTTATGAATCTAGGATTATTGTTTTTCAACATTATGCGTGAGAGAGAGAGTGGTGTAGGGATGGGGAGTTGAGGTGGCTGGGTTCTTGATTATGTGGGAGGGGTGGCAGGTTGGGGAAGGGACGTGGCTGGGGATGCGATGAGGACGGGGGCAGGGGCTTGGAGGGTTCACACAAGAGGGGACTGGGGTGGAGGTGGGAGATTGGAGGTGCGGCAGTGGCAGTCGTGCAGGTTACTGGCGACTACGAGACAGTCTGTGATGAGAGATTGATTTTGAGTCTAGATTGATTTTTGAGCCTAGAGAGAACAAATTCGAGTTAAATTCTCCTAAAATAACATCTCCTGAGCATTTTAACTCCTTAAAAAGTTAAATTCTCCTAAAATTAGCAAATAAATTGGAATGGAGGATTCTTATTTGGAAAAATTATGGTACATCGCATTTCTTGCAAAAATTCTATAACCGTTGGCACTGGTGGAAGGCTGTTTTGATAATCCCACGTACTGCGGGGTCCAGACAATTTGGAGGCACTATAACGGGTTCATTGGAACCCCAAAGGCCACCATCACTCACCATCCCTCCACCATATCATTTTTTTGTCCTCTTTTTTCTTTGGAAGTATATATGCATTTGAGGCTTAGTTGAGTCTATTACTTGAATTGAGATTACAACTACAAAAATAACTTTAGTAACCCAGATCATTAAGGTTGTGATTTGGACCAATTTAAGTGAGGAAGTCATATATAAATAGCACTGAGAAACGAGCGTAAGAGTTGAATAATATATCATTCAAGTTTGTTTTAAAACTTAACAAGTTTTAAAAATATGAGAACTAAACTCGTGTAGCTTGAGTTCTTTAAACATTATAAACTTATCGAGTCAATTAGTAAATTATTCAAATTTGGTTTGAAAACTTTAAAAACTTTTAAAATATTTTCGAGATTTATTTATTTATAAAACAAACTGATCTTAAAAGAGTTTTTAATGAGTCGACTACGAGTCAAACTCAAAATTCATTTATCATCCTACTTGCATAAGAATATAAAATAATAGAGAATGATGCAAAATGAATAGTAACTTAAAAACCAACAAATGAAAGTGCTAGTTATCTTGACTCATAAAGTGCACACCACCCACCACCCCCACAAAAAAAATGGAAAAATCTTAAATTTTCATAGTACCTATTTGGGAAACTTAGACCTAAAACAGTTGTATATATTCCTGGATGGGGTGCAGAGACGTACCAAATGAAAAGCTTCTGCTAAGCTAATAGCAACTCCAACAATAGTAGTTAAAATATTATTTTCTTTATCTTTACTAATTATTTTTCATTGCATCTTCTACACAATTATCAACACCACTGCATAAACACAGCCGCCACCACCGGTGCTCCACCAAACCCGCCATTCCCTCAATCCAACCAAACCCAGTACACACACATACAGAATCAAACTCTTCTGATGTTGAATCGAACCACTCTAAAAGTAAAATATATGTGAATCTAGAAACTTCATCCGCGTTGGCAATGGCTTTGGATGTGATTCAAAGGGCTTCAAGTGCCCATCAATGGCCAAACATCACCGTTGCACAACCACAGCAATTTTTAGATAATATTTCAAAAAACCTAATGAAAGCATAAAGAAATAATTTCTAAAAGTCCACTAAATAATAATTTTTTTGACAAACACCACTTTCATGCAAAATTGCGAACTAAATGATTATTTTTTTTTAATTCTTTTGACTCGGAACGAATCCTAAACAGCAGCTTCCAATTTTTTCGCAAGTTAATTGTCAAGTGAAGATTGATATTAACTGAAATTCTAACAGGCCTTAGTGACGAGCTCATGAAAATTATAAAGCGAAATATATTTTTTGGTATATTATAAGGGTAGCCCTATGATACACATTAAACCACGTTTCTCACAAATATTTTTATAACTAGCTATAATAATTCATAACATTTTCATGCATATCATACACACCTGTTCGTAACTAACCAAAATAACATATAACGAACACTACAAAAAGTCCAAAAAAAAAGCCAAAAAAAAATACGCAGCAAACCATACAACAGAAATGCTTACCAAATGAACCATAAAATTTATAACTATCACAAAAATGCACAACTCTACTATTCTATAGTAGACGCATACGCATAACTCGTCACAATAAATCATTACCAGCACTCGCAAAGCATGTGTACCGTAGGTTTTTGTCCTTCAAAGTATGTGTGATTCTTATTCTGATAGTGTTAAATGGAAAAGAGAAATGTAAAAGTGGGAAATACATGTGAGTTTATGGTGCTATTATGCTATATATTGTGAATTGACAGGTAATAGCAACAAGGAATACTCGTGAACGCAGCAACAGGCTCGAGAATATGTGCTGGCGAATTTGGCATCTCGCACGTAAAAAGAAACAGGTCTCGCTCTCTCGCCAAATACATATAAAAATTGGCTCTCTTTTTTATGCCTAAAATATTCCAGAATCAATTTCACAATAATAACAGTTAGATAATAATAAAAAACTAATAAATTCACAACAATAACAGCTAGATAAAAAAAATCAATCGATCTTCAAGAATTAGTTTTCATTAAATATTCTGCTCCTGCACTTGCATCGCGTGAGAGAGCACCGATCACTAGTATCTATATGTGCATGACAATGGCATGAAAGCATAGAAACACATATATCATCGCAGTGGAATCAATTTCTGATGATAACAAAAGGAAGAGAAAAAAGCACGAAAAATGGAGGAATTGGTATTGGTGCCGTGAGTCTGGGGCCCACTTAGGGTTCCAAATCTTTTATTTTACAAGATCAGATTATCCAAACAAAAAATAAGATTGATGAGACATTAATAACCACATTGTTAGATTACGTTTGTATAGCAAAAAATAAATAATCCATTTCTAAAATAGTTACTAAAAAATAAACGTGATTCATTTATGTACTTGGTGATGTCCAATCCAAATTAGTGGAAGCTGCCAAAAAGAGTGGCAAGGCCATAACCGAAAAGTAAGACACCCACAATGTGGGAACTCAGGATTAACCTCCTAATTACGACAATATGTTTGTGGATAATAATGCTATACGAACAGAAAATATATACAGGAATATGGTATACGTAATCATGCGTTTCTCTAAAACAAATAAATCATCAAGATGCCTTCTAATTACAAGAATACCGCTCCACGTTATATTGGTGGAAGATTCCGTATCCAAATTCTGGAAGAATCTCATTTGCCTGTGTTTAATTTGACAACGCAGAGCGGGCATGATCAACAGACATGCGAATGCACACACATATATGGGCATTGTAGCTCTTCTTTTTCCACGTATATGTTTAATTTATCCTCCAATAGCCCAAAGGCCCTTATGCTCTTCAACTTTTCTGAGATTCCTAAAAATTTCACATGGACTGAACCAAAACAGTACTAGAATAGCTATTCCAATGAGTAAAACAAAACTGTAAAAATGCTAGAAATACCAGCAATAGCTTTTTTTCCCCGCCCCCCTTTTGTGGTAATCAACTTTGAGACATTCATATGATCCTTTGGATGAACGCACCGACGAACACAAGATTTTAACTTTAGGGAGCAAATATAGAAATAAAATATAAACTTTGGACCCACCCTATACATGAAATACAATGGTGATTTCAATCTTAGTGTGGCGAACCTACGTAGTTTTGTAATATTTAGAGCTAAAAATACTATAAAACTAGTCCTAGCCACCAGGGGTGCACCCCTCAAAGAACTCATTCGTCGATATGTAGTTAACTTAGCACATAAAACTAAATTAAAACTGCAAAAAGTAAGGAGTAAAGCCTACCAACTTGATGTGATTCTGTTAATTATGTAGTTTTTCTTCATAATTTAATGCTTCGAGACTTGAAATCTGGGAGAAAGCATTATCTTGCAGATAGCTTGGGATGATGCAAAAAGGCTTGCAAAAAGACGAATCGAGCGTGAGAAAGGTCGGAATGATGCTGCAGAAGACCTGTCTGAGCTTTCCGAAGGGGAGAAGGAAAAGGGTGATGCCAATCAGACAGAGCCTATATCAGAAAAGATTTCCAGAATTAACTCTGACATGAAAATATGGTCAGATGATGATAAATCTAGGCGCCTCTACATTGTCCTAATCAGGTAATTCATAAAAACTACCATGTATTATTTGTGTTCCAAACAAAGCCTTAAGGTAATCGTGTCATGCACTGTACAACTAGTTATACGATACCAAGACAATAAACTAAGGTCTCCATTAATTCCTATACTATACCAATCCAAGCTTTGGTACATAAATTCCAATAGGAAAATGAGGCATTCATATTAACAAATGTTAGTATTGTCGGGGCATTATTCACGGTGATAGATGCATATAAGTCATTATATACAAAAGGTAGTAGTGTCTATAATTTTCATCAATTGTCAAGTACTTTCTTATTCTATTTTTGAACCTTGATGAGTTAAAGCAAAGGAATATGAAAGGGAAATGAAATTGTGCCCGGGAAATCAGACAGAGAAAATGATTCTTTTGACCTCTACCTATTGATTTTCTTCTCATGTTCTTAATCCGATGAAATAAAAGAAAAGAATCTCGCATCTTTCTATCCATTTTTGTTCCGATCTTTTCCTATCCCTTTCCCTTTCCTCCACAATCCAAGATCCAAACATGACATCAATGTCAACAAGATGGTATAGGAAAGATACTGAAAAGTGTACTGTTTATGGAGGGCAAAGAATAGGAAAGGGAAATGAAATTATGCAGGGTACCAGTTTGATATCATGTTTATTGTTTATGTAGTCTACATGGATTGGTGCGTGGGGAAAATATGGAACTCGGAAGAGATTCAGACACTGGAGGTCAGGTAGTAACATAATCCTGGGAAGGAAACCTGTATCGCCCTTAATTGATGCCATCACTAGCCGAAACTGAATGGATAATTGGTTTATATGTTCAGGTGAAGTATGTAGTAGAGCTTGCCCGAGCCCTGGCCAACATGAAAGGAATCTATCGTGTTGACCTTCTGACTCGACAAATCACCTCGTCGGAGATTGACTTTAGCTATGGTGAGCCCAATGAGATGCTCTCGTGCCCATCCGATGGCAGTGGTAGTTGTGGTGCTTACATTATTCGGATCCCATGTGGACCTCGCGACAAGTAACTACATGCATTCATTTCTTCTCTAGCTATGTTTACTTATATCACTATCACGGATGTTATATTTGTAATTTATAACCTTCACTGTAAGTTTTTCGCACTCCTAGAGGTATAATATACCAAAACATGTGTCTAACTTCGAATGCCATGACTACAGGTACATTCCAAAAGAGTCACTCTGGCCTCACATACCAGAATTTGTAGATGGGGCCCTAAGCCACATCGTGAACATGGCAAGAGCTATAGGAGAACAAGTGGATGGGGGAAAGCCGACGTGGCCTTACGTAATTCACGGTCACTATGCTGACGCAGGGGAGGTAGCAGCACGCCTATCCGGGGCATTGAATGTGCCAATGGTGCTAACGGGGCACTCCTTAGGAAGAAACAAATTTGAGCAATTACTTAAACAAGGGAGGTTGTCTAGGGAAGATATAAATTCAGCTTACAAGATAATGAGGAGGATTGAGGCTGAAGAGTTGGGGTTGGATGCTGCTGAAATGGTGGTAACTAGCACGAGGCAAGAGATCGAAGAGCAATGGGGTTTATACGATGGGTTTGATATCAAGTTGGAGAGGAAGCTTAGGGTTAGGAAACGGCGTGGAGTGAGTTGCCTTGGTCGATACATGCCAAGGATGGTGGTATGTACAATCTCTTTTTGCTTGTCATTCTAATAAACCTAGGCTTAAGTTCCTTTTCTGATATTTACATGAAACTAGGTTACACCACCGGGGATGGACTTCAGCTATGTCACAACACAAGATTCATTGGAAGGTGACGGAGATCTAAAGTCGTTGATTGGCTCTGATAGAACTCAAAACAAAAGGCACATACCTCCAATATGGTCCGAGGTCACGTATCATTTTCTTCCAGACTTGTATAATGTCAGCCCTTTGGAATAATACGACCAATCAGATCACAAGCCTAATGCCATTTGTCATTTCTATGGACATCATGTCTACATTAAGTTAACTATTAACCCTTTCACTATATTTAATGCAGGTAATGCGATTTTTCACAAACCCTCACAAACCTATGATCCTAGCATTGTCACGTCCAGACCCCAAGAAAAATGTGACCACTTTGCTCAAGGCTTTTGGAGAATGCCGACCACTCAAAGAATTAGCCAACTTGGTAAAACTATATTCACCGCAATAATCTCGTACTTTATACAAGTCATAGTTCCACACATAATTAGTGAATGACGCCAACTTATTATTGCAGACCCTAATACTTGGTAACAGAGACGACATAGAAGATATGTCTAACAGCAGTTCAGTAGTTCTCACAACAGTGCTTAAGCTCATTGACAAGTATGACTTGTATGGTCAGGTAGCATATCCCAAACATCACAAACAATCCGAAGTTCCTGAGATCTATCGGCTGGCTGCAAAAACAAAGGTAAAATGCATGTCCTAGTTTACCTCCAAGCAAATTGTGAAGTCTTAATTATGAGATGGTACTACGACTCTGAAGTAATCACCAAGCTAGGTTCTGATGAATAGTGGACCATGATATAGTAATCGCCAGTAGAGCCTATATAGATTTCAGATGCAATATCTATGTGAATGTCGGGCCATAGTTCATCAGCCTATATCATTATTTAAGAAGTTACTAATTACTTAACGTTCCTTTCGTTGCAACATTTACACTGCTAACAGTAACACTTCTCTGGCGGATTTGTCTCTTGAAGGGAGTTTTCATCAACCCAGCCCTGGTTGAACCATTTGGTCTCACACTCATTGAGGTTAGTTACCACTATCTGACTCGGCTATTGAACGGACTTAATGCAGTTGAAATTCCTGGAAAAAGAACTATTACAAAATCTTAAAGTTCATATATCTCTATATTTTGCAGGCGGCTGCTTATGGTTTACCAATTGTTGCCACGAAAAATGGTGGCCCTGTGGATATTCTCAAGGTGAAACACATAGATAATTTTAAGTTTCTTCAGTCAGTTGTTTGAGTCATACAAAAACAAAGTCTAACTTATCAGGTGACTCATTGCCCACAACCTAATCAATTTGAGATTTTAATGGGTCAATTAACCCCATCTGTTGACGTTCAACGCCATGATTACCCAAAACTATACACTAATTTGCAGAAAGTGACACACAAGAAACTACGCAAACAAATCTAATTGGGAGTTCCTTGTCAATCAGGCACTCAACAATGGCCTTCTCATTGATCCACATGACCAGAAAGCCATAGCAGATGCCCTCCTAAAGCTTGTAGCTGAAAAGAATCTTTGGCTTGAGTGCCGCAAAAATGGCCTAAAGTATATTCATCGTTTTTCGTGGCCAGAACATTGTCGTAACTATCTTTCCCACGTCGAGCATTGCAGGAACCGCCATCCTACAACCCGTCTTGAGATCATGCCAACTCCTGAAGAACCCATGAGCGAATCGTTAAGGGATGTGGAAGACATTTCTTTAAAATTCTCCATTGATGCAGACTTCAAGCTCAATGGAGACCTTGATGTACCAAATAGACAATGCAAACTCATCGAGGCCTTAACACAGATGGGTTCCTCCAATAGCCCTTCCAGCACTAGTTACTGTCCAGGAAGAAGGCAGGCGCTATTTATAATTGCTACTGATTGTTATAACAGTGGTGGAATGTGCACTGAGACCTTTCCATTGGTCATCAAGAATGTGATGCAAGCTGCAGTCTCAAACTCAGGCAAGATAGGCTTTATATTGTCAACAGGTTTAACTTTACTTGAGACAAAGGAATTGTTAAGACATTGCCATGTAAATTTGGAAGATTTCGACGCATTTGTATGCAATAGTGGAAGTGAAATGTACTATCCATGGAGAGACTCCACAGCTGATATGGATTATGAAGCCCATATTGAATACAGGTGGCCCGGCGAGAATGTGAGATCAATGATAATGAGGCTTGGTCGGGTAGGAGATGGAGATGGAGATGAATATGATATTATGGAAAGTCTAGATGCATTTAGCTCCCGATGCTATTCTTATAGCATTAAACAAGGAAGCAAGGTGAGATTTTTTATAAACCAGTGTATAAGATGTTGTATAATTAAGAAAAAGAAGTTAGGGAGAGATCATAAGGCATGCATTATGCTGGTATAATCTGTCTACTTATCACCCAAATGTGTGGTTGGCAGACTCGAAGGATCGATGAACTGCGCCAGAGGCTACGCATGAGAGGTTTACGGTGCAATGTTATCTACACTCGTGCTTCATCACTGTTGAAGGTAGTACCTTTATTTGCATCAAGAGCTCAAGCACTAAGGTTAGAACTTCTGTTCTTAATTACTGAATTCTATGAATGGTGTATGTGTGCACTCGTGGTTTACGTGCAGTCAACATGAGAGAGAGAGAGAGAGAGAGAGAGAGAGAGAGAGAGAGAGATCAGCCAACCACTCATGTTCCTGCATGTCTTTCAGGTATCTATCTGTAAGGTGGGCTATTGATCTTTCCAAAATGGTAGTGTTTGTCGGAGAAAGAGGGGATACAGACTATGAAGACCTGTTGGTTGGCCTACACAAGACTGTTATTCTAAGAAATTCTGTAGAATATGGCAGTGAGATGCTTCTGCGCAGTGAAGAAAGTTTTAAACGTGAAGATGTGGTCCCCCAAGATAGCCCTAGGATTGCCTTTGGTGCATGTTATGAAACCCATGATATCTCTGCAGCTTTAGATGCTCTACAGGTCATATGA

>AcSPS6

ATGAGTAGGAGAGCTGAAGAACAAAAGAAAGAAGGTACAACAACCCATATTTTTATCAGGAGAGGTGTTTTTCTCATATACCCCCAATCTCCATTTTTTGGTAATTAAGATAGGAGATGATCTCGAAATCCCTCTCAAGTCTCTACCCCTGTTGCGCTTTTGAAAAAATATTGTTACTTTCCGGATATTAACGTTCTTCCTTCCGCTATGCTGATATTTCCCCGAACACTTCCCCTGTAAAACAAAAAACAAAAAACAAAAAACAAAAATAAAAATATTTTTACCGTTTAAAACATCACAAAAACTCCCTTCCCCTATATATCAGCACTTTGTCGCTCTGTACATCTCTCAATCTCTCTCTTTCTCTCTCTCTCTGTCTGCAAATCTTACAGTCGCTACTGTCCTCATCGCATACCGACCCCCTCAGCCAGAATCCTCTCTCTCTTTAGATTCTTACCGTCAGTGTCCTCGTCGCATCACCGAATCACCAACCTGCGAACCAGAACACTCTCTCTCTCTCTCTCTCTCTCTCTCTCTCTCTCCATATATAGGGGGTGGTGATGGCGGGAAACGACTGGATAAACAGTTACCTGGAGGCGATACTGGATGTGGGGCCAGGGATCGACGACGCGAAATCGTCGTTGCTGCTTAGAGAGAGAGGCAGGTTCAGTCCCACTCGCTACTTCGTCGAGCAGGTCATCGGCTTCGATGAGACCGATCTCTATCGCTCCTGGGTTAAGGTATATCACAACATTTCTCTATACGCTTCTCTCTCGCTCGCTATATATGTATCAGTGTGTATTGATGTATTGTTATGTGAATCGATACGTGAGTGTAATTGTTGATTGGCGGTGGTAGGCGGCTGCAACGAGAAGTCCGCAGGAGCGGAATACGAGACTCGAGAACATGTGCTGGCGGATTTGGAATTTGGCACGCCAGAAAAAGCAGGTTTCTCGCTCCCTCCCTCCCTCTCTCTCTCTCTATATATATCTATATCTATATCTATATCTATATCTATATATATATCTATATATATAGATATGCACACACAACTTGAAATGATTGTTTCTGATTAAGTTGAGGACTTAAGATTCTTGATGTGGAAATCTGCCTTCAAATGTTTATACGTATGTATACTTGTATAAGCACAAACGAATTGCTGTGGTTTGAATTATAGGTTTGGAGTGTTAATTGTAATGGCTGTTGCTAATTAAGTGGAGGACGTAAGATTCTTGATGACGAGTTTTGTTGTACACTTGCACATGCAAAAGTGAATTTCAGTGATTTCAATTCTAGGTTTGGAGTATTAATTGAAATGATTGTCGCCAATTAAGTGGAGGATGTGAGATTCTTGATGCGGAGTTTTGTTGTATACTTGCACATGCACAAGTGAATTGCTGTGGTTTCAAATTCTAGGTTTGGGGTGTTATTTGATATGGCTGGTGCCAACTAAATGGGGGACTTAAATTCTTCACGCGGCATTGTGTATTTTAATTTCCTGGTCCCTATACTTAATTACTTATACATGCACTATTGAATGCCTGTAGTTTGAATTCTAGGTTTGGAGTGTTATTTGAAATGGTTGGTGCCAGTTGAGTGGGGGACTTAAGCTTCTTGATGTGGAGTTTGTTTTTTTAATTCGCTGATCCCTGCGATGACGGGATGGGTGTGCTTTACAGATTGGGGATTGGTTCATTATGTGGTTTGATATTGTTTTGATGATTTGAATGTAATTAACCATCGATTTATCATTGAACTTCTTTTGGTTTCAAATTTTACAATTTAATTAGTGTTAAGGAACAAGTGCCACATTCGATTGCAAAAGAAACTTTTGTAGGCCAAAGGGAATGCATTTGATTGTTAGGCTACTTTTTGTTTTTATTTCTCTCATTCTGTTATTAAAAATCAACTTACTTCTTTTGGAGAAGCCTATCTTAGACTATTAGTGGTGTTTAAAAATTGGAAAAGCCTCATTTCGTAGCGGGATGCTGTGTCGGTAGAAAGCCAATAGAGAGTTTTTTTGTCATATCCATTTTTAGCAGGTATCTAAACTTTTTCAACGTTTTTGGGTCGTCATTACTAATTTACTAAAAATGAAGTGGCTGTAGCAACATGTTTTGTATCTTGCTGGCATTTTAATTTGATTTGTACATAAATGTATTAATGAAGATTTTACCAAGTTCATTGACGTTAAGAGTGATACTCTATCCATATATTTTGCTTTGGTTTGTTTTAATATTTTGCTGTTCCAGCATAAAATGTATTTCTTTTCTACATTTGGAAGTCTATATAACTACATTGAATGTCTTTTTTTTTGGTAACTATGCTTCAAAAGGAGGCGCCACATTCTAGTAATTGGATTCCATGAATCTTGTCCTTGTTGGTGAAATTGTGAATATTTAATCAGTTTTATTTGTTATCTACGTAGCTTGAGGGAGAGGAAGCTCAAAGGATGGCTAAACGTCGTCTTGAACGTGAAAGAGGCCGCAGAGAAGCAACTGCTGATATGTCTGAAGACTTGTCCGAGGGGGAAAAAGGAGATAAGGTCAGTGATTTGTCTGCTCATGGTGAAAGCAACAGGGGCCGATTACCTAGAATTAGCTCCGTTGAGACAATGGAGGCATGGGTTAGTCAACAGAAGGGGAAAAGGCTGTACATTGTCTTAATAAGGCACGAATTGAATTCCATTCATTTGGCATGCAAATATTGTAATGAATTGCTAACTCAATTAATCAGTTGTTATTGTGATATTGCATATCGCGTACAAAAATTGGTTATTTAATTGTGTTTTCGACCTGTAATTGTTGAATTAACTTGATTAGCTGTAGAAGGGAAACTTTTTTGAGGTCTCAATGTGTCATGTAGTAGGTGTGCACTTTCTCTTGAATGTTATGTTGTAAAAGGATATGGAATCTCTTGCATGCGCAATGTCTTAATTATTCTTAACTGTTACTATTCTTGCAGCCTTCACGGTCTAATACGGGGTGAAAATATGGAGCTTGGTCATGATTCTGATACTGGTGGCCAGGTAAATTTTAGTTGCAAGAGCTTATAGAGTCAGTATCCTAACCAATTAAAACTGCAAGTCAATGAATGAGAGGTTGCTTGTGGAGACTGCTAAAGATGCGTTTTTTTTCTTCTTTTCTTTTTCTATTAGGTTAAGTATGTTGTGGAACTTGCAAGGGCTTTGGGTTCAATGCCAGGAGTGTATCGCGTTGATTTACTCACTAGACAAGTATCATCACCAGAAGTAGACTGGAGTTATGGTGAACCCACTGAAATGTTGCCTCCAAGAAATTCTGATGTTTTAATGGATGAGATGGGGGAGAGTAGCGGTGCTTATATTATTCGTATTCCATTTGGCCCAAGAGATAAATATGTACCGAAAGAACTTCTGTGGCCACACGTTCCTGAATTTGTTGATGGTGCTCTTAACCACATCATACAGATGTCCAAAGTACTTGGTGAGCAAATTGGCAGTGGGCATCCTGTGTGGCCTGTTGCTATCCATGGGCATTATGCAGATGCAGGTGATGCCGCTGCTCTTCTATCAGGTGCTCTAAATGTACCCATGCTTTTCACTGGTCACTCACTTGGTAGGGATAAGCTGGAACAGCTTTTGAGGCAAAGTCGACTATCAAAGGATGAAATAAATAAGACGTACAAAATAATGCGTCGTATAGAGGCTGAGGAATTATCTCTTGATGCCTCTGAAATAGTGATAACTAGCACTAGACAAGAGATAGAACAGCAATGGCGTCTGTATGATGGTTTTGATCCTGTACTTGAACGGAAACTACGAGCCAGGATCAGGCGTAATGTGAGCTGTTATGGCAGGATCATGCCTCGCATGGTTGTAAGTATTGGAATATCAATACTGAATCTGTTAAGACTAGGTTCCTTTATGGTATATAGTTAACATTATAAATAACTATTCACTGGAATCAAAATGGATTGCTCTCATTCTTGCTTATTACTATCGAGGCAGGGAATGAGGGAAACAGCTAGTCTTGAATTCTGTTTAGGGCAAGACAGCGATAATTTTCTTGTTTGTCAGATTATAATACTTGAAATTGAGTTACTGTACTTCATTCTTGCTTAACTATGTTTGGGGCAAGACAGCGATAATTTTCTTGTTTGTCAGATTATAATACTTGAAATAGAGTTACTGTACTTCACTCTTGCTTAACTTGTCAAGACTCCATAGTACCTTTGAGAGAGACAACTCAACAGCAGGCATTAAACATGGAGAACTTAACAGCGGCAAATTACATCTAAATACACTGTTTGTACATAGAGATAGAATTGGAAAAACGAATTTCATAGCGTTCAATGACTGATCCCAATTGTAGAATTAAAGCCGTTACTTGTATATAGTAATTGTTAGCTTCAAACATCAAATAATGTTCTGTGGAATGTATATATCAAAATTTTATCAGTAATTCCTACATTCTTGCTGATAAATCCTTAATAGTGACAGCATTCATGATAGAGAATTAGAGATGCTGACTTGTTTAATTATGTCAAATGATAAGTTAACATATGTGCTAAATTTGAACCGAGCTTGAACTTGAGAAGAATCTGGCTGATTGGTTCTTGTTTTACAACCCCATTTTAAATTGTGGTCTCTAATGTTAACCAAATGTAACTGGCTTGCTAATTGGAAGTACACGTCGTAATATTACAGATTTTTGCAGGTAATTCCCCCTGGGATGGAATTTCATCACATTGTTCCACATGAAGGTGATATGGACGGTGAAACTGAAGGAAATGAAGATCAGCCTACTTCTCCAGACCCACCCATTTGGCCTGAGGTTCCAATCATTTCTCTTCATTTTCTTTGATGCGTTCCTAGAGTGATAAAATGCCTGCAGTGCTAGTCCTTCGGGATATAACTTTTTCTCAATCCTCGAATTTGTTAAGAATTTCTTGCCAATAACTAAGTTGACGTTATGGTATGCAGATAATGCGTTTCTTTACTAATCCACGCAAGCCGATGATACTTGCCCTAGCTAGGCCAGATCCCAAAAAGAATCTCACAACTTTGGTTGAAGCATTTGGGGAATGTCGTCCATTAAGAGAGCTGGCTAATCTGGTACATTTGAATCACCTCTTGTTCTGTAACATTTTTTATTTTGGTTGAATGAATTGTTTTGACTGTACTAAGTTACTGAATCAGCTTTGACATTAATGGCCTTTGTCCTGTATTTTACAGACTTTAATAATGGGTAACCGAGATGATGTTGATGAAATGTCAAGCACTAATTCTTCGGTTCTTCTCTCAATACTTAAGCTTATTGATAAGTATGATCTCTATGGTCAAGTGGCATACCCCAAACACCACAAGCAGTCTGAAGTTCCTAATATCTACCGTCTGGCAGCTAAGACAAAGGTACTCTTTATCGGCTATATGACTTGGCTGGTTGGATATGGATCACTAGTTGAAAGCTGAAATAAAAACAGGACGGATGCTAGTTAATCTATTTATGATACTCTTCTAGTTCTCTATGTGGTAAATCAAGACAATTACTTCCCATGATACCTAAATAGCCAGCTCTCTTGGAACCTAATTATTGCTTTCTTTATTTTTCTTCTTATATGGGTCTGCTCTCCAGGAATCCTGCTTTTTACTTGGGCAATTCTGGAATCCTTTTTTCTACAGTAATCTATATTTTTTCTACAATGATGAAACTTATCCCACCAACTTTGGGTGGCTCCAGGAATCAATTTTTTTCTTCAATGACGCTTTCTCTTTGACCATACCTTCTTCCCTTTTGTGTTGTTTTAGAAAGTATGAAAGTTTATGTTCATGCACGTGCACGTCTCTCCTAGTTGCAAGTTGTGTCCATTTGTACTTTTTCTTGCAGTTAAATTTTATATTTTGTATTTCCTGCATAATATGAAGAATTTTATTTTATTTTTTGTTTTAGACGATTTTGTAGAAGTTGTGGCTACGAAAGATGAGACTTTTGTATGGCATGCTTCTATTAGAGATACAATGGTAGTTGTTTTGTTTGCACTTATGCAGAAATAATAATGCACCAACAGTTAAGCGGAGCTGTGAGTCAGAGAGGGAAAGTAAATTCAGCTTCGGATGGTTAAGGAATTTAAGTTTTACTTGTAATTGAACTGCATGATAATAGAAAAAAATGACTTTTCCTAGTTTTTTCTGGATATGAAAGCATTACGATTGTGGTTTTCAGTCAATGGACTGGCAATACTAGATGATGCTGAACTATGAGTTTTATGGTTGTTTGAAAACATGAAATATTTTTCATTGAGGAAAAAGTAGCTTGTTTTTTATTTGACGAACAAAAATAGTTTGAATCTGGGTAAATAATCTGTTTAAGTTCTTTTCGTCTCTTGGTTTTATGTAATTGGCTTCCGTTGCACTGTCCTATAAATCAACTCAATGCTGGGAGGAGATGAATTATAACACTTCACGGGAAAGTAAAATAGCCTTTGCTGGGACAAATGAAGCCCAGATAACATATTAGAAGCAGCTTGGATCTCTTTAGTATTTATACCAATAGACAAATATGTTTGATCCCTTTACTGTAGTTTCATCTTTCTCTTACTATTAAACATGGTTTTAAAAATGGGCAGCTTTTGTTACGTAACGGTAGGGGTCACTCTCGCTGCCTATTGCGGCTGCTATGCAAGTGAGACTGAAATTCATATGCATAACAGCTACAACCGTTACGTAACTCCCGTTACACCACATTTATGACCGGTATGACTGTTATGCAACTGTCAGAGTACATCCATTTTTCACTTTTAGCTTCTAGATTATATTTGTTTAATCAACTTTAAAGCTTATCTCTCTCTCTCTCTCTATCTCTATCTCTATCTATCTATCTATCTATCTATCTATATTTTTATGTGTGTGTGTGTAGTTGAAACTTACCAAGTTGTATGTTAGTTTGACAAATAGAGTGTAATAATGGTATGTTTTGATTTTTAAATTTTATTTTCACTAGTTACATTCACCTATTTTGTTTATTTACCAATTAACACATGTAACAAGTTTGAAACACCACATGAATCGCAATTGTATAATTATTAAATACATAAATAAAGGTCAATAGACCAGTGCATTATAAATTTATGGAACTTGAAACTGTTAGCTATTTATAGAAAGTATATAAATCTCTTCTTAGTGACTTAAAAATATTGGAATGCGAATAACATTCCAATAAAACCGTGAAAAACTATTATAATCCCAAAAAATAAATTATGACAAAAAAAATCGTGCCTATCAGTAAGTTTTCATCATGATGTGAAAAACAGAAAAAAACACGACCCTGGTTGCCGCTACAATCATTACACTCTGAAACCACATCAACAACATTACTGTTACGGTAACTACAACCGATTTTTAAAACCTTGCTACTAGATAGTTGTCTATCGTTCTCATGCAGTCGTTACAATTAGCTTCCACATGATTTCATTTGTCCTGTCCAGTGAAGGCTCTGAAATTAGCTGATCTTGTCATACTAGTTTGAATCTTGATGATGTTCTTGGATGATAATAACCTGTAAATAAAGGGTCCTGCCCCTAAAATTTTCATCCAGACTTACTAATTAAAATACCTTTGTACATTGCCATAAATGATAGGAACAAGGAACTCGACCTATATCCTGCAAACTTTTGCACCCGCTTATGATTCGCTGAACATTTGTTTATTGTACAGTGATCATTTCATTTCTCCATGTGTTTCTAAATCCTTTGTATTTTAGACCAGTTAATAAGGCATCTTAACATTGCATGAGTCCTCTGAAGAATATAAAAGGTGTTTTTGTGGGATTTAATTGAAACTGGATTGTTTTCATGGTATTACCTTATCTTATCATGAGATTTCTAACTGCATGAATTATGGATTAATTATTTCTTTAGTTGGGTCTACTTTTTTTAAAAAATCTATTACGGGATTTTGTCATGGTTAACGTATGATGGTTTATAGCTGGACACAGAATAAGAGAAACTAAATTATGTCTTTGGCTCTGTAGCATGATAGCCATTTAATGTAGCTGTTTTATACAGGATAACCCAAGGTAATGGTTGTTATTTTAATACAATAGTTTGTCATAAATTCATACATGCTAAGGTTGTTTTAGTTCGTTTTNNNNNNNNGCTCTCTCTGTGACTTATCATATTATTAGATATCTTATTCTGTTACCAAATTTTTCTAGGGCGTTTTCATTAATCCAGCTTTCATTGAGCCATTTGGGCTTACTCTGATCGAGGTACACTTGTTATACCTGTGAAGCCCTTAGATTTCACTAGTTAGTCCACTATAATCAAGTATGGCTTCTTTTCTATTGAACAGGCAGCAGCTTATGGTTTACCAATTGTCGCCACAAAAAATGGAGGTCCTGTTGACATACATCGGGTATGTCTACAGATCAGTCCCAATTTTTTTTTGATCTTGGGAATTCTATCGATATAATAGTGAAGTGTGACAGTTATGCTGATAACAATTAGTTCTGAAGATCATTCTGATAACAATTACTGTATATCTGTTGCAGGCTCTTGACAATGGTCTCCTTGTGGACCCCCATGATCGGCAGTCTATTGCTGATGCTCTTTTAAAGCTGGTTGCGGATAAGCAACTTTGGGCCAAGTGCCGTCAGAATGGGTTAAAAAATATTCACCTTTTCTCATGGCCAGAACATTGTAAAACTTACCTATCTCGAATAGCAGCTTGCAAACTGAGGCAACCATGGTGGCAAAGAAGTGACGATGGGAATGAAAATTCAGAGTCAGATTCACCAAGTGACTCCTGGAGAGATATACAGGATATATCCTTGAACTTAAAGTTTTCACTGGATGGAGAAAAGAACGAAGGTAGCGGAAATGCTGACAGTTCTTTAGACTTTGAAGATCGCAAGAGTAAGTTGGAGAATGCTGTTCTGACATGGTCAAAGGGAGTCCAGAAAGGCACACAAAAGGCTGGGCTTACGGAGAAAGCAGATCAGAACAGTACTGCTGGAAAGTTCCCAGCGTTGAGGAGGAGGAAGAATATTGTTGTTATTGCCATGGATTTTGGTGCTATATCAGATCTCTCCGAAAGTATTAGAAAGATATTTGATGCTATGGCGAAGGAAAGGACCGAAGGCTCTATAGGATTCATATTAGCTACATCCTTTACTTTGTCTGAAGTTCAGTCTTTTCTCATCTCTGGGGGACTGAGCCCTTCTGATTTTGATGCTTTTATCTGCAATAGTGGTAGTGATCTCTACTATTCATCTCTTAATTCAGAGGATAAACCCTTCGTTGTTGACTTATATTACCACTCACATATTGAATACCGCTGGGGTGGAGAAGGGTTGAGGAAGACTTTGATTCGTTGGGCTGGTTCTATCACTGATAAGAAGGGTGAAAATGAAGAGCAGATTGTTACCGAAGATGAAAAGATTTCCACCAATTATTGTTATGCTTTCAAAGTGCAAAATGCTGGAAAGGTAAATTTTTTTCTACTTGTGAATGATTTTCTTATGAAGTGGCTTGTTGAATTTACCTTACTTGCTCTTAGGGGTGTCAGTCAAACCAAGCAGTGGGAGATTGGCTCACTCTTGACTCGGCTTGAGCTTATTTGAGCTCAGTCTTAAGATAATGTGAGTCAACCTCAATATTGGCTTGTAGGACGTGTGTTGTAAAAGTTAGAGCTTATATGCTATCTAAGAATAACAGAACCAAATGTACTTGTTTACATTAATGAGATAACGTGATAGCATGAATAATACAAGGCACTTGCATAAATGTGTCATCTCTAGGTTTTTTAAATGATTTCTTTCACTTTCACCTTCACTTTCCTGATTTTTGTTGCCCAGGCACATAAACGAAGGTGGTCAGGCTTTTTATTCTTCAACTTGCACTAACAAATTAATGGCCATAACTTTACCTATTTGACCATAATATTTCTCTTTTATTGTACTAAGTTGGGCAACCATATAAAAGCATTACTTTTTTGAGTCAAGCTGGGCCGTTTGTTGATCAGTGTGTAAGAGAATGAGTTGAGATGGAGCAAGATTTTGAATATTTAAGCTGATGGAAACAGAATTCCAGGATCACTAAAAACACGTAAACATTAAACCAAAAAACAATATTCTTTTCGTGAAATCGTATACTACGGAATTAGGGTTATACCTGCGAGATCTCTTTGGCTTGATCTCGAAAGCCAAAATCTGAAGCAGAACGAGAGGGAGTTTTAGACAAAAACCTTCCTTCATCTCTCTTTCATAAGCAGTAGTCCATTCATTATCTCATTTAGAGGCTGTAGGCACTAGCTTAGCTGGTGATAGGAGGCACTGGCTTATGGCGGTAAGAACTACTGACACTGTCATCTTCCTGCGCTAGTGTGTCTGCTCATCCGGTTAGAATCAATGGGTGTCGAGTGAGTATTACACCAAGAATTGACAAGCGGATGACTTTTCTAGTGCGAACAAACCTGTAGTTTTCTTTGCTCCGCCTTTTTGAATAAGCCCCGCTCCCTAGGGGGCCCCTCTCTGGTAAAGAAAGAAAGAATCTCATTATGAGAAAGATGGTACAATGGCTTAAAAGACTTTCTATAGTTCTATTTCCATTTTTTGTCTCCTACTTATGGAGACTCTTGCTTTTCAAACTATTTGCGGGAAACATTTTTTTAGATCCTTTGTTCGATGCCCAATTATATATGAATAACGAAAATAGAGTGGATTTGGATCTCGAGTTGAGACTGGCCCCCCCGGTTCCTCCGAGAGCAGATGAGGGCTATATTGTGCCTAAAATATCTTTCGATTCCCTGCCCTTCCAATGGATATGGGATCACGAGCACAGTATTGGGAATCTCATCCGCGAATTGCGGAACGAGGAGCATCTTAGGCAACCTGCTCCTGAACAGGTGGAGCAGATCGTGTCAATGTTGCAGAAGAAGAATGGAGGAATGGAAAAACTTCCCCAGATTCTTTAGGAAATGCAGGAAAATAGACTCAAAAGTCTATACTATAAGGAGAGTCAAAATCTTTATAGCCAACTTATTAGTAAGCCTAAGCCCCGCTGAAGCTTTCAATATAGTATAGACTTTTGACAAATTCCGTCGTCAAGGCATCTCTCCTTATGTCTCAAGTCCGTGTCACATGATGATTACTAGAAAGTGTGAGCTCTAAAACGTGATCTATTTCGCTCTCTATTTTAAAAAAAAATATATATATGTGTGTGTATATATATATTTTCAGAATATCCTTAGAGAACTGTGAATGCACACATATATATAGGCTACGGTAGGGACATCAAGGAGTCAATAAGGTTTGACTTCCTATGGAAAGAAAGAAAACCAAATTCAACCCGAATTAAATTCCAATTTAGTATTTTTCATAAATTATAACTCATACATCTAAAGAATTATAATTGCACTTCCTCCCTTATTTGAAAATTACATTTCAAGCTCCATGATACTAAATGCTCATTAATCTCGCCACGTTAAGATTAAAATAATTACTGACAATTATAATTAATCGACATCTTCAAAAATTTCTTAAGACCCTCCACTTAACTTATTTGATATGTCAGATATAAAATCTACGTGTAGGGTTTGACGTGATCGAATCTTATAATCTTCATCAAGGGGCATGATTAATCTCTATACTGGGATGAGGATTCCGTCAATAATTAATATTCATCATACACGTAATGCGACCATCCAACTCACCAAGTTTGTTGACCCACAAAGGTATCTCACTCCATGAGAATCAAAACAATCAACATTAAATGCATGTGTCTAAAATTTATTTCGGGATTAAGGGTATAAGCACTCATAATAACCATGTGGTACAAATTGTCTTATAAAATTAGTACAAAAAACCAATATCCCCAAAGTGTAATAGCACAATGAGTCAAAATTATACCATTTTTCGTAGTCAAGACAATGTTACAGCCCTAACGATGGTTTGTCCAATTCCATCTAAAACTGTGAACTAAAAACTTATATTCTACAAGAACTGATGATTAGATTTTCCGTGTGTAAGCCGAACTCTAAACACCTAATCATCTACTACGTAGAATAAAAGACACGCAAGCATAATAGAAATATTATGCTACAAACATTGCCCACATAAATTCTCATTAAAATGGAATAACGAAGTTTATTAATAAATAAAATTAGTACTTTTTTGTAAGCTAAATAAATTAATATTAAGTTCCATAAAAATCATGGCTTTTAGTATATTTCTCCAACCTCGGCATGCTTGAGCCATTAACTGAACATTATTTACACTTTCTCGAGCCAAGCCTGGGCAATTATGACCTTGGCTTGGCTAATTTACACAGCTACTCACCCCACCTCCTCATGCAACTGATTTTCATGCGAGGACTTAACAAAATATCTTAGCTTTATGAGACATAAAGCTGGGGGTGGGTTGATATTGCTAAATATCATAGTCAGTATTCTAGTTTTGGTAAGATAGTGAAATGAATTTAGAGTTATAAAGTTCTCTGCAACTATATCTTTGTGTGTCTAATTATGATCTTTGTGCATCGTCGCATATCATTCTTCTTCATCTTATTACTGTTACATCTTTACAGGATCCTCCTGTTAAAGAAATCAGAAAATTAATGAGGATTCAGGCTCTCCGTTGCCATGTTATTTATTGCCAGAATGGGAATAAGATAAATGTAATTCCGGTATTGGCATCTCGTTCCCAAGCCCTAAGGTATTGCTCTTATAGATTAATTAGTTGTTAGCTTATGTTCTTTTTTTTGGGGGGGTGAATATTCAATGATATGCTGCAGTTGATCTTTTAATCATTGCACAAAAACCTCAGTATTTTGTTCTTGAAATATGATGGGAAATAGTAATTGTTAATTAGGTCTTACTAATCTTATTCAGGCACAAATAATGAAATTCTACAGTTTGTCAGTGTTTGGTATTGTGTACGCAACTAATTAGTCATTGAAACCAATACATGTGATAGTAACAAGGTCCATACAGTTTTAGAGTAGGTTGAGTAACATGGAAACTTAAAGGTTGCACTATGATTGCACCGCCAATAGAAATGCCAAATATTACATTTTATGTGTATCTTACTGAGTTTATTTCCACTCATTAATTTTTACATATTTTTTAGAAAACGAGAACTTGACCCTTCTCTAAATTTGTTGTAGCTAGCCAGGTGTATAAATGCAGTGTCAATGTTATATAACTTTACATCCAACACACTGAATGATGAGGCGTCCACATTTTTTTTTTGATTTTTTTTTTTATATAAGTGAATTTTTATCATAAAATTATCATTTGCCATTTGAACCAATGTCATGTTTATATAGCTTGCCAGTATGTGGGGAACTTTCGCATTGGATTTGGTTTTAGCAGGATACATTATACAAACATTGAAAAGGACTTAAAAATTGCCCAGATCCACAGCACTCCTTACTGCAGAAACCTCTCTCTTTACTTCGTGGTTGGAGTGCTGCTATGCTGGTAATTGGTACCACTTAAGACTGTTAATGTGAGCAAATTGGGCTGTCAAGAAGACAACACATTACATGTTCTAGAGGCACCATTTTGACCTGATTTGCCATGACATTCCATCCAGAGTCCAATTAACTTCAGACTTTAGAGTGAGTGAGAGGTTGCCTCGGTGATTATTACACAATTCCACATGTCACTCATAGTGTTGACCCATCAATGGATCCATCCTTTGGCTAGATCACTTTATAACTTAAAACGATGAGCTCATAGGTTAACTAGGCTCCTGTGGACAGTATAGGATATAAATGTTCATCGCACCTTTAGTTATAAGCTCATAGACCTCAACACTAAAGAATCAGAGGTTAGGCTCTCTCACAGTCTCATGTGGAGTATATGATTGTTTAGGTCATATCCTTTTTAAAACCTTAAGGGCATGTTTGGTTTCTTTTCTATTGTTATTTTTTTTCTTTCTAATTTTGAAATAAATATGACGAGGAGTATGAAAAAACAATCCTCTCCAATTTCGCCCTCATTTTTGTTTTTTTAATGAATCAATTTTTTGAGTAAAGAGAGTTAAAACTGCTTTCTATAAATGCACTGTTTCAAAAGATTATCTTGAAAGTAGTTTTGCTTCAAAGATTTTTCTGTATCGTTTCCCATTTTTTTCTGTTTCTGAAAACTGATAGTAGTTTTAAAACAAAACCAAATCTGCTTGAAGATTTCTCACCTGATTTGGTAATATGGGCTGGCAAAGGCTTGCAGTTGAATCTGTTCACCAATTAATTGCAGACATCCATGTACTTGGTCCTCCCATGTGAGGGGCGAGTGGATCCCTCACTTAGGCAGTATCATTTTTTGTTTGTCGGAAACCTCCTAAAATTGCAGCGATGTGGATGTGAGCTTCATAATTTGTTAATCTAATACTATGCCGCTGATGTTGCTTTTTCAAGTGTTACTGCAATAACTCTGTGCTTTGCAACCTTTTTTTTCTGTTTATTCACCATGCATTTATGATTCAAGATTCCACGTCGGAACATAGCATGGAAGGATGGTGATTCTAAGGTCACCACATTGAATCATCTTGTATTTGTCGTTACGGATTTCAGAGTGTGTGTGTTTGTGTGTGTCTATTCCTTGAGTTAATGTTCAACAACTAGAAAGTTTCTTGAATCTGAGGTCTTGTTGGAAATAATTTCATAATTTTGATATGACTTAAAACCTAGGAAGCATGAATATGGGATACGGGTACGATACGACATGGGTACGACAACACGTCTAAACCTAAAAAACTAGAATACTGGTACGACGGGATATGTCAATAATAAAATATAGTTTATATTTTATTACTACAAATATAACCATAATAGATACAATTTGTGCTATTCTTTAAATTTTTTGAAGTTAAAACATTTATCAAGAGGAAAGTTATTCATGTTTTCTTTCCTATGGAGTATAGCTATGAATATAAGACACGAATGTGGCCATAAGAATATGCAAGTATGATAATCTGAAAGAATAATGATTGGTACACACCCAAAAGTGGGTGTGTACAAGACACACTCATTTTGCCCTCTCACAAATAAGTGGGACCCGCCCTAATTTGCCCTCTCACAAAATAAGTGGGACCCGACCTAATTAGGGCGGGTCCCACTTATTTTGTGAGAGGGCAAAATGGGTGTGTCTTATACACACCCACTTTTGGGTGTGTACCTATCACTACTCAATCTGAAAACATAAGGTACTTTAAATTTGCTATTTTTATTGTTACTATAAATAAACCACATATGGTACAACTATTATTGTATTTATCCTATGAACTATAGTCTAATACATGACACATTGACAATAAATTTTAAATTACGGATACGTTTAGGAAGCATTAGATACACATTTGCAAGTATCGAAAGATTATAGGTATCCGATGCAGCTACGATATGGGTACAAACCCCTCCTAGAAGTATCCGTGCTTCATAGCTCAAAACTTGTTCTTTGAATCAGCTGTTTCTTTGAATTTTTTATGAAACTTTGTTACTGATAGCGCACTCTATTCAGGTATCTCTATCTCCGGTGGGGTGTGGACTTGTCAAAAATGGTGGTTTTTGTTGGAGAAAGCGGCGACACTGACTACGAGGGCTTGCTTGGTGGCATACACAAGTCTGTAATATTGAAGGGAGTTTGTAGCGGTCCGACCCATCAACTCCATGCCAACAGAACCTACCCTCTTTCCGACGTCCTGCCAATTGACAGCCCTAACATTGTTCAGGCAGCCGAGGAATGCAGCGGTGCCGACCTCCGGACCTCGTTGGGGAAGCTAGAGTTCATCAAGGGCTAA

>AcSUS1

ATGGCAGCCTTGAAGAGGTCTGACTCGATAGCTGATAGCATGCCGGATGCCTTGAGAGAAAGCCGGTACCACATGAAGAAGTGCTTAGCTAAGTACATTGAGAAGGGGAAGAGGTTGATGAAACTTCACCACTTAATGAGCGAAATGGAGAAAGTGATCGATGATAAGACCGAGAGAGAGCAGATCTTGAACGGCCTTCTTGGCTACATTTTATGCACCACTCAGGTATATTTTCATCTCGAGTAATGATATACAAACACCAACATCATTCCCCAGATATATACAAACTCTTTTAGCAGATTGCTGTCTAGTCGCATCATTCTGGTCGATAATTATGTATCCCTATAGACTAATCCTGTAATATCATATTTTTTTCTCTCAACAGGAGGCAGTTGTTATTCCTCCATATGTTGCCTTTGCAATTAGACCAAATCCCGGGTTCTGGGAATTCGTTAAAGTGAGCTCTACAGATCTATCAGTAGAGGGCATCACCGCCACGGACTACTTGAAATCCAAAGAAATGCTGGTTGATGAGGACTGGTATACCGATTACTTGCAGCCTGTTACTAAAATTTATTTCATTTTAAAGCTGAAGATTTACTAAAATTTAGGTATTTGTGTTTGCTGATATTGCAGGGCAAAGGATGAAAATGCTTTAGAAGTTGATTTTGGCGCGATGGACTTTTCCGAGCCTAACCTGACCATGTCTTCTTCGATTGGGAACGGAATCAATTTTATTTCCAAATTCCTTTCTTCTATACTATATGGTGGCTCACAGAAGGCTCAGCCTCTTGTTGATTACCTACTCTCACTAAATCACCATGAAGAAGTATGGAAACAACTTTTTTTAATTGCAAACAAAATTAGTTAGTACAAGTAATTTTAAAATTTCTACAGAAAAATACTAAGAATATTTGTTTTTATTAAGGTTTGGTCTTAAAATTTGCAGAAACTAATGATTAACGAGACCCTCAACACCGCTGCCAAGCTTCAGAGCGCGCTAATAGTAGCTGAAGCGGCCCTTTTGACACTGCCCAAGGACACACCATACCAGGACTTTGAGCAAAGGTGATTGATACTAATTTTTATTTCAAAAAAATTTATGGTTTGTGTCGAATTGATAGCTAATACCATGCTTTTTTTACAGGTTTAGGCAGTGGGGTTTTGAGAAGGGATGGGGCGATACTGCAGAAAGAGTGAGGGAGACAATGAGATCGCTTTCAGAGATATTCCAGGCACCGGACCCGTTAAACATGGACAAGTTCTTTGGCAGGGTTCCAACTGTTTTCAATGTTGTTTTGTTTTCGGTCCATGGGTATTTTGGTCAATCTGATGTCCTCGGTTTGCCAGATACCGGTGGGCAGGTACACATTTTTTCCAGCACATCAATCTGATGTTTCCTAATTTTACCAGAAAAGTACCCAGAATTTGAAATGTACGGATCAGATTCATTGTGGCTGCGTTAAATCTACACCTTAGACTTTGAAACTTCGCTTTGTTACAAACAACAGGTGGTCTATGTATTGGATCAAGTAGTTGCTTTTGAAGAAGAACTGCTCGTTCGGATTAAGCAGCAAGGGCTTAATGTGAAGCCTCAAATTCTTGTGGTGAGTATCAAATTTTTCTCAAAATTCTTTTGCTAACTATAGCATTAATCATATATACTCTCTTCTTGGTAATAATAGGTCACTCGACTTATCCCCGATGCCAAGGGAACTAAGTGCAACCAGGTGCTAGAACCGATTGCCAACACAAAGCATTCCAACATCCTTCGCGTGCCATTTAGGACGGAAGATGGAGTTCTTCCGCAATGGGTTTCTCGTTTCGACATATATCCCTACCTCGAAAGGTTCACTCAGGCAAGTTCTACATCAATCATCAAATACAAAACTTACCAAATTTCGTTTTCAATTTCGCCTTTTGTTTCTCATTTTCAGTTCTGTTTTCAATCAACAGGACGCTACGGACAAAATCTTGGAAGTCATGGAAGGGAAACCGGATCTCATCATCGGAAACTACACAGATGGGAATTTGGTGGCATCACTCATGGCTAGCAAACTTGGGATTACTCTGGTGCCTCCATCAGTTTCATTTATTCATTTCTTTTTTCTGCAGTTTTGTACTAATAATTGAGAATTACATTTTACTAATTTGGAATGTTCTGAAATAGGGAACTATTGCACATGCTTTGGAGAAGACAAAGTATGAGGATTCAGACCTAAAATGGAAACAATTAGACCCCAAGTATCATTTCTCATGCCAATTCACCGCAGACACGATCGCAATGAATTCTGCAGATTTCATCATCACCAGCACATACCAAGAAATTGCTGGAAGGTTTCGTGTTTAATTTCGTACAAAATGAAAACAATCGTGTTTATTTTTGTACAAAACGTAAACAAAAGTTACCATACAAATGTACTTATTGAAATATGAATCTTTTGTAGTTGCAGCAAAGATAGGCCGGGGCAGTACGAAAGCCACGCTGCATTTACACTTCCAGGGCTTTGCAGAGTTGTTTCAGGCATAAATGTGTTTGATCCCAAATTCAATATAGCTGCTCCCGGGGCAGACCAATCCGTCTATTTCCCTTACACAGATAGACAGAAGCGATTCACTTCATTTCGTCCTGCCATAGAAGAACTACTCTTTAGCAAAGTTAATAACAATGAGCACATGTAAGTGTTGAGAGTTGCTAATTGTTCCTTTTTTTGAAGCATCTAAGGCCATCTCCAATCTTAATTTACTCTATTTTTCATCTCAAATTTCATCGGATTTTAGTAAAAAATTGAAATTTGATGGAGGATTAGGTTAGCCTTTGATTTGATACCACTTCGGTGAAAGGATTGAAGCTGATTTTGGTCCTTATTTAAAGAGTTTCTAATTATGAAACTGTCAATTGGATTACTTGTTTGAAGCATCTAAGGCCATCTCAAATCTTCACTCTATTTTTCATCAGATATGGAAACAAAAAAAATGTTAAAAGCTTGCTCTGATGTGTTAACACTCCAAACCATAACTTTTTTTCATCAACTCTCTCTATAAAAATTGCCCAGCTTATCCCATCAACCATTTTTTTTATGAAAAGAAAACTATCTTCAATCCTTATTTGAAATTTTTATACCATTTTGATGAAAAGATTCGAGCTCATTTTGTTTTTTTATTCATCGAATATGAATTTGATGAAGGATTAGAGATGCCCTAACACCTTATTTGAAATGTTTCCAAAATCAGTGGATATCTAGAAGACAGGAAGAAGCCTATTCTGTTCTCAATGGCAAGGCTTGATATAGTGAAGAACATCAGTGGATTAACCGAGTGGTACGGGAAGAACAAGAGGCTGAGAAGTTTGGCTAATCTCGTTGTTGTCGCAGGGTTCTTTGATCCTACTAAATCCAAAGACAGAGAAGAGGCAGCCGAAATAACAAAAATGCACATGTTGATTGAGAATTACAAACTTAAGGGTCAGATTCGATGGATAGCAGCACAGACTGACAGGCAACGAAACGGGGAGCTGTACCGCTGCATTGCTGACACAAAAGGAGCATTTGTGCAGCCTGCACTTTACGAGGCGTTTGGCCTCACGGTTATAGAGGCAATGAACTGTGGATTACCTACTTTTGCAACGAATCAGGGAGGCCCTGCAGAGATCATTGTTGATGGGGTTTCGGGGTTCCATATTGATCCGAATAATGGGGATGAGTCGGGGAACAAGATTGCTGATTTTTTCAAAAAGTGCAAGGACGATCCTGAGCACTGGAACAGGATTTCCAAGTCGGGTTTGAACCGTATCTATGAATGGTAAACAAGATTTGTCTCTTTTCCCACTCTTTCTGTTCGATCAACTGAAAATCAGTCTATAAATCTTGAGTTTCTTTTTTAAATAAATACCATATTAATATATATCCTCATTTTTTTTTGTACTTGTAATTCGACAAGTTTGAGTCACATTTGTGATATTAGTGCAAATATATGATAATACAACATACGATTGAACAACATTCCTGATTATGCCACTGTTTTCTATGTACCTTTTTTTTTGGCAGCTATACATGGAAGATTTATGCAAACAAGGTCTTGAACATGGGGTGTGTGTATAGTTTTTGGAGGCATTTGAAAAAGGACCAGAAGCAAGCAAAGCAAAGATACATCCAAATGTTTTATAATCTCCAATTCAGGAACTTGGTATTAACCTGCATTAAACATAACAGATTTTGCTCAAATTTTGCTCTTATGTCACTTGAATTGACAGCTTTTTAACCCTCACAGGTGAAGAATGTCCCCACTTCAAGGGTTGAACCTCAACAACAGCCTAAGGAAAAACAACCCAAAGCACAGCCCTCTCAAAAGTATGGTACCCAACTTCTCCCCACATTAAGTTTCCAGAACCACGTTCAGTTACTCTGTTATATTTTTTGTTATGCGATCACAGAATCGGTAGATCTACGAAAACAGTAAAGAAATAGAAACAATCACGCATGTAAGAGAATACAAAGATTTACGTGGTTCAGCAGTGTACCTATATCCACGACGGAGTGAGAGAATAGAAATCCACTTTACACAATAAGAATTACAAGAATCAACCTTTCCGTGTTCTCTCTCAAATTATAAAACACTCTCTTACTCCAAACCCTAATCTAAAATTATAATAAATACCCCAAAATTCGGGACGGGGGCAACGTCCCCAAAAGTTCCGGCTTTGGGGCCTCAACGGCCTTAACAGTAACTCAAAGAGAAATACAAGATTGCATAACTTATTTTAGTTTAGAATTTTTCTCCCCAACTAATTGATGATTTTTTTTTTTGGTAGCGTCAAGCGCACACAAAGTCGGTTTCAGAGGTAAAAATTACAGTTCAAGTTGACTGATTGCTGTGTTTCACTTTCTACTGTTGATTATAGGTAACAAAACCACTCAAACAAGTTCAATAATCTCCAGGTTGTTCGGATCTTGAAGACTCAAAACACAAGGCAGATTGATTCAATGCCACCCTGCTGTTTTTTTTTGGTTTTTTTTTTTAAATAAAATTTCATTGTTTCATAAGCATGAGAACAGTGATTTGTATACTGAAATAATCAAAGAGAAATCAATAAATTCTTTGATGTGTGGAAACATTCAAATGTGATAATAAAAGTGTTGCTAGCTTTGGTATATCTTTTGTTGTGTATTTGAGCAAGTCAAACTCTATTAATTATGAGCAAAGAAAAGGAAGAAATGAATTTGGCAATGAGTACTATGGTTGATTTATAAATGGCAATTTGAGAAAAATGTAGTTAGCATATAATCATCCAGTTCTTTTAGAGAAAATTGATACTTCTCATAAATCCCACAAGGAGGCTTACTAGATTAGGGTTCCATTGATTTTGAAGTCTGTATGAAACGTTTGGGATAATTTTCATCCAAGTTGGATTAATTTTTGTCTCTAGCAATTTTTATCATCTTTTGGTTAAAAAATTTTAATGAAAAAGTTTACTTTCTGATTCATCTTAAAGTAAATTTTTTTTTATCAAAAGATGAAAAAGTTTGTGCTAAACAGACAACTTGCATAGGATTAGATAGAGAGACGAGCAACGCTAGGATCACGATGAGAGAAGGGGAGAGAGAGATAAAAGTTGTTGCGAAGGGGAGGGGATGGCATGGTGGCATTGGGGTGTGGGGTGGCTAAGGATGGCAATTTCCCCAGGAAAGTCGGGTTCTCGCCCCTAATAGGTCGGGTTTGGAGTAGGGTAAACGGGTTTGAGGCAGGTTATGGGAGAAAAGTCCTAACCCATCACGGGTTCGGNNNNNNNNNNNNNNNNNNNNNNNNNNNNNNNNNNNNNNNNNNNNNNNNNNNNNNNNNNNNNNNNNNNNNNNNNNNNNNNNNNNNNNNNNNNNNNNNNNNNNNNNNNNNNNNNNNNNNNNNNNNNNNNNNNNNNNNNNNNNNNNNNNNNNNNNNNNNNNNNNNNNNNNNNNNNNNNNNNNNNNNNNNNNNNNNNNNNNNNNNNNNNNNNNNNNNNNNNNNNNNNNNNNNNNNNNNNNNNNNNNNNNNNNNNNNNNNNNNNNNNNNNNNNNNNNNNNNNNNNNNNNNNNNNNNNNNNNNNNNNNNNNNNNNNNNNNNNNNNNNNNNNNNNNTTTATTTATTTTGCCTCTATCCACAACGATATCAAAGATTAAGAAAAAAAAGCTTTAAGAACCTTTTTTTATAATGAAGAAAACTTAGAAGTAATCATTTGGAATTAGTAAAGGACGATGTAAAATCCGATTAAATATATTAGGAATAAAATTAAAAATATTAGTACATAAGAGTCCAAAAGTGACACAATGTGAATACTTTAGGGACTATTGACATACATTCATGAAGAAAAAGAATAATGTTTAGGAGTTCTTGGGCCACACTCTTGAACCCTAAACATTATGAAAGAGTCTTATCCATTTTTGAAACTTAATTCAAGGTCCTATAATTTAAAATATGCCAAAAATATTACCATGTTAATAAATATCCAACATTCTATAATTCAATCACATAAATATTTTTTTTCAAAAGGACAATAATTAATAGCCATTATGGTTATTCCCATATTAGAAAACTCTTTTTTCTCTCTAATTAACACCTCTCTATATATAATTAATATATATCTATCTCTAATTAACCCCCCTCTCTCTCTAGGATCCACAGTGGAAATGATACCGATGGGAGAACATGATGATATGTTGAAGAACACGACAATGAAAACGATAACTACGATGAATTTTATGAAGAAAGCAAGAAAAAAATAACATTGAGATGTTTTTCTTGACTTAACACAAATTATACGATTCAACATTTAACCCAACCTAGTTTGTTAAATTAAACATTAAATTTGTTATGAAAAATTTGTCATAACTTATTTTATAAACCAACCACTAGTTAAAAAAAAAATACCGATTCTTATGATAAATTTATCATAACAAAATTACTATGAGCAAACTAGTTAATGTTTTTACCTAGGGGTGCACAAATTACCCGACCGACCCGAGAACCGACCTGACGTCATTGGGTCGGTTTGGGTTGGTTCCACAATGCTTTGGGTTGGTTCATGAGTCTAAAAAATGAAGACCCGTACGGATAGGGTCGGGTAGGGTCTCGAGTCAATGAGATTTTTTACCGAGATCCAAACCGAAACGAGATCTGAGAACCGAGACCTAACTCGAGACCCGAAGTCTAGAACCGAACGTATAGACATACAATGCACTCGCGCACACTTGATTATTACCTATGCTATATTAATTTTAGGAAGAACCGAAGACCCGACTCGACCCGAGAACCGAGAAAATGGACAGAATTCGAGCGTTTCTGGTCGGTTCTCCTCGATTCTTGGTTCGATTCTGGGTAAAAAAATAGGGAACCATGAATGGTCGGGTCGGTTCCAGGTCTATCACATACCCGAACCGAACCGACAACCACTATTTTTACCTATGATAAATATATCATAGTAAAACAATTTCATAAATTAGGCACTGATTTATTTATGATAAAGTTATCTTAACAAAAATATTTTATAAACCATATAATTAAATTTTGAAATGGTTCCAGATTATGTAAAAATTATCATAACAAAAATTTTAATTATTGTAAAATGGTTTTAGGTTATGGTAACATTATCATAACATTATTTATAACCATATGCATTAATAATTTATAATATATATACAAGATCTATATTTTTGGCTTACATACAAAACCATCACAAAAAATCAAATACACTTTTCGTGTGTTTAAAAAAAATATATTATCTTTTTTTTAAGGCAATCCAATAATAGTAAGGAGAAAACTCATTAACCATTTTCTATCTCTATGTTATATATTATCGAAGAATAAATTTTTATTCTTTGAGAGCCAAATCATCATACAAATTTATTAAATTTTTGAGTTTTAAATAAATAAAAAAATATTTTGACTACTCAATCTATACGTAAAAGGAAAACAATAACAAATCTATAATTATTTCTGAATTTATAATTTTTAACAAGAAATTAATTTTTGATAAATAAGAATTTTATTACCATATAAAGAAAAGACTGATGTATATTTTTATCTTCAATTCCATAAATCTATCTTTCATTTGTTTCATATAAATAAAATAAAAAAGCTCTTGACTAGCTCAATCAATCAAACAAATTAAAAAAAAAATCAAAATTATTATATAATCATCCCAAGATTTATGATTTTTTAAAAGAAATTATTTTTTAGTAATTAAGCATTTTAGTTGTCATAAAAAGAAAAGATTATATTTAAAGAATGTAATAGTAAAGACAATCCAACAATAGTAATGAGCTAACTTATTAGCCATTTTTTTTGTTAAAAGCTGAAGTAATTTTAAAAGATTGAAATAACAATATTAAATTTTTTTTATTATTTTCTAAAAAAACGGCAAATCTTTCTTTGCATTAGGAAGTGTAAATATATGGAGATCAAAATCTTGACAATATCAAACATTTAAGAATTAGATTTTCTAATAACTTTAAGACAATGGTTCTACTTATTTTATTTGTTTAAGTTTGACCATGCATAGTACCGGTTAGCACGAGTTTTTGCTAGTATTATTAATAGAAAATTGAATTTGAAAAAAAAGTAAAGTATCACGTAATATTTTTAAACATATGAACTAATTGCCTGATATTTATAGAAAATTAAAAAATATTATTTTTATTTATTACACTATAAATATAACTTAATTATAATAAATGATTTTAAGATAGGTCCAGGTAGTTCCAAATCTAGGATTCTTAATTAACCCTAATTTTAACGTAATTCTACTCTGATTCAACGTTTTGAGAGAAAGAGAGAGAGAGGCCTAGTTCGAACCAACAAAACCAGGGTTCAAAACACGTGATTCGCAGCCCTCCGGCTCTAATTGTTGTTGGTTTCGCCAACAACGATTCACTGTTTTTATTTTTCCAAGCATAAATCCATCTCTCTCTCTCTCTCTCTCTCTCTCTCTCTGTAGAGAGAGATTTCGAAGATGGACCTGGGAGCACCGATGGAACCTCTGAATCACCCTCTGCCGCTGCAAAGTAGCATTCCAAGAAACCCAAATGTGCGTTTTCTTTTTAACCCAAATCTTCTTTTCTTCTCTCTCTTCAACTTAAACATTCTGCACAAAATTGTTCTTGAATCAATTTATCATTGTTTTCTAACCCCAAATTTTGTGATTTTTCTTTTGGGTATTTTTTCATTTTATGTGATCAATGTATTGTTCTTTGATTCAATTCTTACAATGAGGTGTCTAGGATCGTACTGGGTAATTGCTGAATTTTTTGTTTGCAAGAATCGTATGCTATGAATCAAGAAGATTTCAATTTTGCTATGACGATTTCAATGAGGTGTCAATTGGTTTCAATTTTGTTTGCAAGAATCTTTACACTATGAATCAAGAAGATTTCAATCTTTATGTCTAGGATCATGTTTGATGTTTGTCTAATGAGGGTTATTTTGTTTGCACTAATCTTTATGCTACGAATCAAGAAGATTTCAATTTTGTAATATTAACATCCACTTTGTGGTTTATACAAGTCAATATTATAAATGTGTTGAAGACCCATTATAAATGCCAAAAACTGCAGTCAGTTAACAAAAAAACCTAGAGACAGTGCATTATTTTGTACAGTGCATAATCATATGTCATAGAATCCACAAAATTGCGAGTCCGTCAGTATGCACTGTCCATGCATTGTACAAAATGCACTATCCTTAGCAAAACTTGTTAGCATGAATTGGGTTTATGCAGATCTTTTTGGTTGCCCTTGATATGTTTTTTTTGGTTGCAAATGTAGGAGACGTTGTGACTATATTTCCAAACAAAGATTTGTATTTGTAACACAATTATATTTTTCCACCATATCGAGCTTGATCAAGAGTATGCGTCGATTCTTATGATCTAGTTTTTATGAATCTCAATGTTCCAATATTAGGGTTTGATTACTTTGGTTGAAGAATTTATGACTGTTCCCTTTAATTATATTTTTTATGTAGTTTGTTCATGAATAAACCAATTGGCTGATGTTTGTTATGTGTTCTCGATTCGAAGTTTACGGAACAAGAGCTTCCTGCTTGCAAACCAATTCTAACTCATGGATGGGTAAGCTGATAGTAAATATGGCAGAATCTAATTTTGAAAAGTCATTTTCTCACTAGATTTTTGTAATTAATTCTCAATCTTCTCTCATGTTTCAGGTCATTACAGCAGTTGTATCTGTTGGCGTCATCTTCATCCCCATTGGCCTTCCTTGTTTGCATCAGAACATGTAA

>AcSUS2

ATGGCAGCCTTGAAGAGGTCTGAGTCGATGGCTGATAGCATGCCGGACGCCCTGAGAGAGAGCCGGTACCACATGAAGAAGTGCTTTGCTAAGTACATTGAGCAAGGAAAGAGATTGATGAAACTTCGACACTTAATGAGCGAAATGGAGAAAGTGATCGATGATAAGACTGAGAGAGAGCAGTTCTTGAACAGCCTACTTGGCTACATTTTGTGCACCACTCAGGTACATTTTCATCTAGAGTAATGCTATACAAACACGAACATCATTCACAAGATGTATACAGAATTCTGCAACTGACTGCTGTCTAGCCACATCATTTTGGTCGATGATGATTCTGTAGACACAAGATATATACAAAATTCTATAGCCGACTGCTGTCTAGTCACATCAATCTGTTCGATGATTCTGTATAAATATCATGTAACAAAAGCGTTTCCCTATAGACTATTCACTAGCTAGTCTCATAGGCATGTGTAATATCGATCTTTTTTCCCCCTGACAGGAGGCGGTTGTTATTCCTCCATATGTTGCCTTCGCCATTAGACCAAATCCTGGGTTCTGGGAATTCATTAAGGTGAGCTCTACCGATCTATCGGTGGAGGGCATCACTGCCACGGACTACTTGAAATACAAAGAAATGTTGGTTGATGAGGACTGGTATATCAATTACTTGCAGCTTATTACTAAAATTTATTTCATTTCAAAGCTGAAGATTTACTAAAATTTAGTCTGAAGATTTACTAAAATTTAGTTATTTATGTTTTCTGATAATATTGCAGGGCAAAGGATGAAAATGCATTGGAAGTTGATTTTGGAGCGATGGACTTTTCCACGCCTAGCCTGACCGTGTCTTCTTCGATTGGAAACGGAATCAATTTCGTTTCCAAATTCCTTTCTTCTAAACTACATGGTGGCTTACAGAAGGCTCAGCCTCTTGTTGATTACTTACTCTCACTAAATTACCATGAAGAAGTATGGAAACAATTTTTTTTATTGCAACAAAACTAGTTAGTACAAGTAATTTTAAAATTTCTACTCTTGTTTTGATCATGGTTTGGTCTTAAAATTTGCAGAAACTAATGATTAACGAGACCATCAACACTGCTGCAAAGCTTCAGAGCGCGCTAATAGTAGCTGAAGCGGCCCTTTTGACACTGCCCAAGGACACACCATACCAGGACTTCGAGCAAAGGTGATTGATTCTAATTTTTATCTCGGAAAAAATCTATGGTTTGTGTTGAATTCATAGCTAATATGGACGCTTTTTTTACAGGTTTAGGCAGTGGGGATTTGAGAAGGGATGGGGCGATACTGCGGAAAGAGTGAGGGAGACAATGAGATCTCTTTCAGAGATATTCCAGGCACCGGACCCGTTAAATATGGAGAAGTTCTTTGGCAGGGTTCCAACGGTTTTCAAAGTCGTTTTGTTCTCGGTCCATGGGTATTTTGGTCAATCCGATGTCCTCGGTTTGCCAGACACCGGTGGGCAGGTACACATTTTTGCCAGCACATCAATCTTATGTTTCCTAATTTTACCCCGAAAAGTATTCAGAGTTTAAAATGTGTGGATCAGATTCGTTCTGGTCACCAAGTTTAAACCTACACCTTAGAATTTGAAACTTCACTTTGTTACAAACAACAGGTGGTCTATGTTTTAGATCAAGTAGTTGCTTTTGAAGAAGAACTGCTTGTTCGGATTAAGCAGCAAGGGCTTAATGTGAAGCCTCAAATTCTTGTGGTGAGTACCAAATTTTGCTCAAAATTCTTTTACTATCTATGGCATTAATCATATATATATATATACTCTTCTTCATTATATAATAGGTCACGCGACTCATCCCCGATGCCAAGGGGACTAAGTGCAACCAGGCGTTGGAACCGGTCGCCAACACCAAGCACTCTAACATCCTTCGGGTTCCATTTAGGACAGAAAATGGAGATCTTCCGCAATGGGTTTCCCGTTTCGACATCTACCCCTACCTCGAAAGGTTCACTCAGGCATGTTCTGCATCAATCATCAAATACACATCTTTCCAAATTTCATTTTCAATTTCGTCTTCTGTTTCTCAGTATCACTTTATTTTTCAATCAACAGGACGCTACGGACAAAATCTTGGAAATCATGGAAGGGAAACCGGATCTCATCATTGGAAACTACACAGATGGGAATTTGGTGGCATCGCTCATGGCTAGCAAACTTGGCATAACTCTGGTGCCTCCATAAGTTCTATTTTTCTCTTTTAGCTTTGTACTTGTTATTGAGAATAACATTTACTAATTTGGAATGTTTTGAAATAGGGAACTATTGCACATGCTTTGGAGAAGACAAAGTATGAGGATTCAGACCTAAAATGGAAAGAATTGGACCCCAAGTATCACTTCTCCTGCCAATTCACCGCTGACACGATCGCAATGAATTCTTCAGATTTCATCATCACTAGCACATACCAAGAAATTGCTGGAAGGTTTAGTGTTTATTTTTCGTACAAAATGAAAGCAGTCGTTTTTATTTTCGTACAAAACGAAAGCAAAAGTTCCATACAAATGTACTTATTGAAATATGAATCTTTTGATGTTGCAGCAAGGATAGGCCGGGGCAGTACGAAAGCCATGCTGCATTTACGCTTCCAGGGCTTTGCAGAGTTGTTTCAGGCATAAATGTGTTTGATCCCAAATTCAATATAGCTGCTCCCGGGGCTGACCAATCCGTCTATTTCCCTTACACTGATAGACAGAAGCGATTCACAAAGTTTCATCCTTCCATAGAAGAACTACTCTTTAGCAAAGTTGATAACATTGAGCACATGTAAGCGTTGAGAGTTTCTCTATTTGTTCCTTTTTTTCAAGCATCGAAGGCCATCCCCAATATTAATTTATTCTATTTCTTACCTCAAATTTTATCGGATTTGAGTAAAAATTTCAAATTTGATGGAGGATTAGGTTAGCCCTTGATTGATACAACTTCGGTGAAAGGATTGAAGATGATTTTGGTCCTCTTTTAAAGAGTTTCTAAAAATCAAACTGTCAATAGGATTATTTGTTTGAAGCATCTAAGGCCATCTCCAATCTTCGCTCTATTTTCATCAGATTTGAAAACAATAAATTGTTAAAAGCTTGCTCTGATGTGTTAACACTACAAACCTTAATCTTTTTTTTTCCCATCAACTCTTTCTAAAAACAGCCCATCTTATCCCCGAAACCCCATTTTGATGAAAAATTTCGAGCTCATTTTGTTTTTTTATTCTTTGAAAATATGAATTTGATGAAGGATTAGAGATGCTGTAACACCTTATTTGAAATGTTTCCAAAATTAGTGGATATCTAGAAGACAGGAAGAAGCCTATTCTCTTCTCAATGGCAAGGCTTGATATAGTGAAGAACATCAGCGGATTAACCGAGTGGTACGGGAAGAACAAGAGGCTTAGAATTTTGGTTAATCTCGTTGTTGTCGCGGGGTTCTTCGACCCTACTAAATCCAAAGACCGAGAAGAGGCAGCCGAAATAAAGAAGATGCACATGTTGATCGAGAAATACCAACTTAAGGGTCAGATTCGATGGATAGCAGCACAGACCGACAGGCAACGAAACGGGGAACTGTACCGTTGCATTGCTGACACGAAAGGAGCGTTTGCGCAGCCTGCACTTTATGAGGCGTTTGGCCTCACGGTTATTGAGGCGATGAACTGTGGATTGCCTACTTTTGCAACCAACCAAGGAGGCCCTGCGGAGATCATTGTTGATGGGGTTTCGGGGTTCCATATTGATCCGAATAATGGGGATGAGTCAGGGAACAAGATTGCTGATTTTTTCCAGAAGTGCAAGGACGATCCCGAGCACTGGAACAAGATTTCCAAGTTGGGTTTGAACCGTATCTATGAATGGTAAACAACCTTTGTCTCTTCTCCCTCTTCGATTCGCAGTCTCTATATCTTGGGATCTTTTTTAAATAAAATTTTACAAGTTTGAGTTACATTGTGATTACGCCATTGTTTGCTATGTAACTCTTTTGTTTTTGTTTTTGCAGCTATACATGGAAGATTTATGCAAACAAGGTGTTGAACATGGGGTGTGTGTATAGTTTTTGGAGGCAGTTGAACAAGGATCAGAAGCACGCAAAGCAAAGATACATCCAAATGTTTTATAATCTCCAATTCAGGAACTTGGTATGTTGCTGCATTAAACATGACAGATTTTTCTCAAATTTTGCCCTTATGTGACTTGGAATTGACTACTTTTTAACCCTCACAGGTGAAGAATGTCCCCACTTCAAGGGTTGAACCTCAACAACAGCCCAATGAAAAACAACCTAAAGCACAGCCCTCTCAAAGGTACGGTCGATCCACAAAAACAGTAAAGAAACAGAAACAATCATGCACGTAATTGAACACAATTTGTGAAGTTCCACCACAAATCCTTTCATTTCAAAGTCACTCGTCCAATCATGCAATAAACCTATTTCTTGTTACCAGTATACGTAACCATACAACGCCTTTCATTTAGTTAGGGAATTTTCTAGCCAACTAATTGATGATTTTTATTTTTTTGTAGCGTCAAGCGCACACAAAGTCGGTTTCAGAGGTAAAGATTACAATTCAAGTTGACTGATTTTGTTTCATTCCTATTGTTGGTTAGGTAATTACCAAATTCACTCCAACAAGTTGAATGATTTCTCCAGGTTGTTCGGATCTTGAAGACTCAAACACAAGACAGCTTGATTCAGTGCCTCCCTGTTGTTATTCTTTCTTTTTTTTTTAAAAAATAAAATTTCATTTTTTACAAGCATAAGAAAAGGGATTTGTGTACTGAACTAATAAAAGAGAATAATGGTGTATTTGATCTGATGCAGATCATTAAATTCTTTGATGTGTACATGGAAATATTCAATGTGATAATAAAAAGTGTTGTGAGCTTTGATATTTCTTTTGCTGTTTTAAAGTGGTTTATATAAAATTCTTTTTTCTTTATATATATTGTGTCACTGTGTTCGTTTACATACTTATTTTTTTAACTTCATCGTATGAAATTGGATTTTACGTGGTTATTTACTAATTACAAATACTTTTTTTATAATTACAGATATTTTTTTATTACAAATGGCTCTTATTTTTTTTTAATTTTTAATTTTTAATTTTTAATTTTTAATATCGATCGTGAATCTGATTTGATAAATTCTTTAAATTTTTTTATTTATTTTGCCTCTATATTCTATCAATTAGGATCCACAATCTATATCAAAGTTTAAAAAAATTCAAAATTCTATCTATAAGAAAGAAAATTTAGAAGATATATTAAGAATAAAATAAAAAAAAAACAGTTTATAAGGGACCAAAATCATCTCTCTTCAACGTTTGGAGGGAGAAAGCGAGCTAGAGAGCGAGATAGACCGGTAGTTCGAACCAACCAAAGCAGAGATTAAACACGTGACGCGCGGCCTCAGGATCTGAACTGTTGTTGGGTTCGCCAACAACAATTCAGAGTTTTCACTTTTCCACGAATAAATCCATTTTTCACCGTCTTCCGATTCCAGATTCCTTTTCTGCTCTTTCTCTCTGTCTCCATGTAGAGAGAGATTTCGAAGATGGACCTGGGGAGCACCGATGGAGCCTCTGAATCACCCTCTGCCGCTGCAAAGAAGCATTCCAAGAAACCCAAATGTGCGTTTTCTTTTTAACCCGAAATCTTCTTTTTTTTTTCTTCTCCCTCTTCAACTTCGTAACCATTCTGCTCAAAATTCTTCTTGAATCAATTTATCATTGTTTTCTAGCCCCCAAACTTTGTGATTCTTCTTTTGGGTACTTTTGGATTTTATGTGATCAAGGTTTTATTCCTTGATTCAATTCTTACTGGGTAATTGCTGAATGTTTTGTTTGCAAGAATCTTTATGCAATGAAATCAAGTAGCTTGTGTCTAGGATCATACTGTGTAATTTCTTTCAATTTCGTTTGCAAGAATCTTTATGCTACACTGATCTAAAAAGCGGCTCCCTAGGCGCTAGATGAGAGTGTCCCGTCTCGATTAGTTTAGGTGGGCGGCAGTAGGCAGCATGTCTTAAGATTTAAAGTTATATTTCAAAAAATATTTCCATTAACTTAACGAATTGGATTAAATTAGTAAATAATAAGTTTTTCTCTAAAATAATATCATTTTATTTTTAAAAAGAAAAAGAAATAACTAAAAATAATTCAATATTGAACATTTGGCGAAAAAGAGTTAGAGACCTAATTTTTAATTTTTATATATTTTAGAGATTTAAATTATTATATTTATCTTTCTCATAGTATTATTTAACATTGAATATTTGAACTCTACTATTTTGATGTCATTTTTTTGTTGGTATTTGATATTTGGTCATTTATTGTTGTTTTTAGTAAATATTAATTCTTTAAATAAATAAAAAATAAAAAATAACAAAACTGCCTAGGCCCCGCCTAGGGCTATGCCTCCCTTGGCCGCCCGCTTACCGCCTAGCAATTTTTATAACATTGTTATGCTATGAATCAACAAGATTTCAATCTTTATGTCTAGGATCGTATTCGGTGTTTGTCTAATGAGGGTTATTTTGTTTGCAAGAATCTTTATGTCATGAATCAAGTAGATTTCAATTTTGTGACACTAACGTCGACTCTGTGGTTTGTACAAGGCATTACTATAAACGTGTTGAAGACCCATCTTATAAATTTCAAAACATAAAAGCTAGAGAGAATGCATTTTGTACTGTGCATATTTTCATGATGGAGTCCAAAAAAATACGGGTCCCATCAATGTGATTATGCACTATCCCGAGCAAAACTCATTTAAAAAACTGCATTCAGTTCAACTGCATGAAATATGGGTTTCTGCAGAATATATTTGTTTGAGTGTGTTATTGGAAGTTCAACCTGTCCTTGAAATGGGATTTTTTGTTTTTGTTGCAAATGTAGGAGACTTGCCACTATATGTCCAAACCACATTGTACTTGTAACACAATTATGTTTTTCCACCATATAGATTATAGAGTTTGATCAAGAATATGCATCGATTCTTATGATCTAATGTTTATGAATCTCATGTTCCAATATTAGCGGTCTGATAACTTTGGTTGAAGAATTTATGACTGTCCCCTTTTAATTATATTTTTTCTGTAGTTTTTTCATTAATAAACCAATTGGCTGGTGTTTGTTACATGTTCTAGATTCGAAGTTTACGCAACAAGATCTTCCTGCTTGCAAACCAATTCTAACTCATGGATGGGTAAGCTGATAGTAAATTTAATTTTGAAAATCCATTTTTCACTGGATTTTTGTATTTTATGCTCTCTCTTCTCTCATGTTTTCAGGTCATTACAGCATTTGTATCTGTTGGCGTCATCTTCATCCCCATTGGCCTCGCTTCATTGTTTGCATCAGAACGTGTAATTTGATCTTATTATATGCCACTGGTGAAGTTTGGTAGCCAATATCCACTTGATCTTGTATACGAACTTCTCTTAGTTTGACTTTCAGGTGGTGGAAATTGTAGACCGTTATGATAAGGATTGCGTTCCTTCAACTTATTGCAATGATACGCTTGCATATATCCAAAGCACCCAAACCAACAAGACCTGTATCAGGAGTTTAACTGTTAGTTGAAAGCCCTATTACTGATGAGTTCCAATAATTTTATGCTTCTTCTTCTTCTGTGTCTTTGTGTTTTTTATCTTTGTTTTCCAACTCTCCTATTCAGATTAACAACTACCCATCTACCAATTGCATTCCTTGAATGTTTTTCAGGTCCCAAAGCAAATGAAAAGTCCCATCTATATATATTATCAGCTAGACAACTTCTACCAGAATCATCGCCGGTAACTTACATAATTTGATCGATTGTATTAAATGGATTTAGGTGGTCCTTCTCTTAATTTAAGTCCTAGCATGAATTATCTCATCACGACTGTGGACTTATATTATAGACACCCAAATCGCTTATTTTCCAAGGGCTGGTCTAAGATTCGAATCTCAAGTGGTGGACCAATGGTGAAATTCCATTCCTCACTCCCCCTTGAGGTTACCGCAGGGCTTAGGCCCGGTAATTATGCGGTATATGTGGGACCTGGGCACTCCCTTGGGGATTAGTCTGGGTGAAGACCTGGACACCCTCGCCAAAAAAAAAAACAAAAAAAAATCACCTATTTTCAAGTATTGGGTATAATAATAAGCCTTATACCTACAATTTTAATTTCTGTTGGTTTTCTCTTACTTGAGGCTTACACTGTTCTTTTTATTTTTTATTTTTTACTTTGCTCAAATAGAGCTATTAAACTCTCTTTTTATCCATATCCAAGTGCAATTTTTGTACTCAAAGTTGGGTTATAATTCTCTTCTGTGTATAGAACTCAGTACTGCAATAGATTATTTACAATACTTTTATAAGCTTCAAGTTCTGTGACTGTACTGATTATGCTCTCAAGTTCAGAGGGAATGTTGAACTTTCCTACCTCTTACTTGTGACTGTAAATTTCCAGGTATGTTAACAGTAGAAGTGACGAGCAATTGCGCAGCAAGGCATATGAGTTTGACACAGATGACTGTGACCCGGAATCAAATACAGACAAAGGTCCCATAGTTCCTTGTGGCCTTATTGCTTGGAGTTTGTTCAACGACACATATGGGTTTTCCTTGCAGAACAAAGTTCTCCAAGTCAGTAAAAAGGGCATTGCATGGAAAAGCGACCAAAAGCATAAATTTGGGTCCGACGTCTATCCTAAAAATTTTCAGAGCAGTGGTTTGATTGGGGGTGCAAAACTCAATAAAAGCATACCCGTACGTAGATGTTTTATTTTTTCTATCTAGTTACTTCTGCTTACGTTTCAATTTATATTTTTGTTCGTTTTGCTGCATGTAACAGTTGAGCAACCAAGTGGATCTTATCGTTTGGATGCGAACTGCGGCATTGCCAACTTTCAGAAAGCTCTATGGGAAGATAGAGGTGGATCTTGAAGCGAATCAAAAGATAACAGTGGTAATACAGAACAATTATAACACGTATAGCTTTGGGGGTAAAAAGAAGCTGGTCCTTTCAACCACAAGTTGGTTTGGTGGAAAAAATGATTTCCTGGGTATAGCATACCTTACAGTTGGTGGACTTTCCTTATTTATGGCAATAAGCTTCATACTTTTGTATGTTGTTAAGCCAAGGTGAGTGAAAGAAACACAAAATGTCTTTGCTAATTTTGATCTACATATGAATTTTAGTTTGCTTGTTATTGCCTTTGAATATATATATATATGTTGGCGTATTTTTTTGGGGTGAATAATTTGTTCGTTGTATTAGTTTACGAAGAAATATTCTTTAAATATTCTAAGGTTATCAGTGTGATGGAAGGATTGTTAACTTTGTGTCCATTTTGTCGAATCTCAACATTTTCTATTGCAGCTCCATTTTCAGTCAAACTCCCTATAAAGTACCCAACATGGAGAAACTCTTATCCGATTCCTGTCAAGATCAGCAATATCTTGCATTTGGAAGTTAGCTCTCACAATTTTATTGAGTCATGATTATTGGTTTCTGGATTATTAGAGCTTTAGTTTTTAATATTGCCTTAAACCCTCCCCTAATAAATTCCATTTTGCAGCTCCTGGTATTACTTGACCTTGTGGGAACTTGTGAGACCTAAGAGTATCAGCGGTTGATTTGGAACAAATCTTTAGTCAAGATAAGAATTAGATGAATTGGAATAGATTTGAGGGTTCAAAGGCAAGAACAGCATTACTGTTCTTTGCAATTACCTTTCAAACATAGTGATATTTCATTAGCTACAGTGGACAACAGTTTGACCCTTCTCAATTTTTATTTTATTTATTGGTTGTTTTCTACTCTTGAATGCAATGCAGTTATGGTAAAGTTTTGTAAGTTTCCTATGTTAGTGCTACAACTGGCATAGGTTGGAAATTTCATTATTATTAACTTTTTATGTGTATCCTTTGATAGAAACATAACCAGTGCTGTGAGGAAGCACATTTGCAAGACTAGCATAGGCTGGAAATTCGTGGGAGAAGTGCAAAGCAGTAACTTTGACATGGCGGGGACAAAGAAGACCAAGAGGCTTAAAGCCCAAGTCAAGGTAGCTAAGTTGGCCAACATATGGAAGTTCATCGACCAAAGCTGCTATTGA

>AcSUS3

ATGATGGAAAAACAAAACGAAACAATCATACCATGGGCAGTGCAGTAAGTGATTTGTCCTCCTACGAGACTTCAAACCGCTTGCCGGTATGGTTTAAGTGATCCTTCCCAAAAGAGGAAGTCGCTTTCGAGATGGACGATAAACATTGGACACCATAATACTAGGGGCACGATATTTCAATGGACCGTCTGTCTTCCCGTGGGGATTCGGTTCGGACCTGCAAAACTTCATCAAACCGCTCACTACTAATTTTCCGCGTGTCTCTATAAATATCTTATATCTCTCTCTCTCTTTCTCTAGACAACAACTAGCTTTCTTCCCGTTCCTCAAATCAACTACTAGAATCTTCTTCTCTCTCCCACTTCTTTCTTTCGATCAACGAACCTAGTCAACTCCAATCAAAATAGCATCAATGCAGACTGCGAAATTGGCGCGAATCCCCAGCATGAGAGAGAGGGTCGAAGATACCCTCTCCGCTCACCGCAACGAACTCGTCTCTCTCCTCTCCAGGTTTTCTTTTCTTTTCTTTTCTTTTCTTTTGTGTTTACTGTTTTGATTTCTCGGTAACCAAACAGGAGTTTGTGGTTTTCTGTGTTTTCGAAGGTATGTGGAGCAGGGGAAGGGGATATTGCAACCGCATCACTTGATCGACGAGCTTGATAAGATCGTCGGCGATGACGAAGCGAATCTTACTCTCATCAATGGTCCGTTCGGCGAAGTCCTCAAGTCTGCACAGGTGAGTCTTTCTGCTTTCTTTCGAAACTTTCTTTGAAGCGTTATAAGGAGGACGAAAAAGTAAAAATAAAAAAAAATCATCTGAATTATACATAATTTAAACCTAAGTAGCTTGTGGTTTTGATCTTTCGAAGAAGATTTAATTTCTATTCGTTTCGTTAGCTTTTATCTTCCAAACAGGACATTCGTCTTCGTTTCTTTCATCTGAATCAATTTCATGGAATGGAAATTCAAGTATGAAGTTGAAGATTCATGTATTGGCGTTAAAGAAACGCGTTTAAGATCGTATAGGATGAGTTAAAAAGCTTGTTCTCTATGGAATTTTGACATGAATTTCTCCGGTGTCATTATTACACTTCATTAAATGGATTGATTCTCTAGGCACAGTCATCCATTGTTATATGCGGGACTTTCAATTCCTTCTAGAATATGTGGCCAATGAGAAAGATTTACAGTGGCCTATGGAATAAGTTAGTAGTTTCTGCTGCCATGGTATAGTTCCGAAGAGATGCTCTGTGTTGTTGTTGTTTTTCCCTTCTCAAAACATAGTTTCCAAAAAAATAAGTGGGTTTTGGTTTCTGCATTTTGTGTGCTTATTTGTTTGCTGGTTTATTCAGTAGAATCTTGTTTTATTTTTGGTTGTTAGGAAGCCATAGTTCTGCCTCCATTTGTGGCTATGGCTATTCGTCCGAGGCCTGGTGTTTGGGAATATGTACGTGTAAATGTATATGAACTCAGTGTGGAGCAATTGAGTGTTGCAGAATATCTTCGTTTCAAAGAAGAACTTGTTGATGGACAGTAAGTGGTTTGTTTCCTTTTGTTAGATTACATTTGCAGAAAAATCCATGAGAATCGTGTGCATGCTAGCTTCAATTGGACGAGTACTTCTTCGAATATTTCTTATTTTTTGATTTTAAAATGGGCCAGGTCCAATGACCAATATGTGCTTGAGCTTGATTTCGAACCTTTCAATGCAACATTTCCTCGGCCAACTCGAACATCATCTATTGGCAATGGCGTTCAATTCCTTAACCGACACCTCTCCTCAATCATGTTCCGCAACAGAGATTGTTTCGAACCATTACTTGATTTCCTCCGAGCACACAAACATAAAGGACATGTAAGTTTCGTTACAGGTCTTGTTGCGAATTTCTTCTGGACATAATTTAATATCTGAACTGTAAAATATCTTTGAATCCAAATCCTGCCTATAGGTCATCTTGAACTAACCATTTATTTCCTTATTTTTGTGCATTACTCACATTATTAGTAAATATAAATCTTTCTGATAAAAATATATGTGGAATTAACTTTGATATTCCTTTGGTTGCTAAGATGCACAGGGATTGATGGCTTTATCTCCCAATTGTTTTATGTAGGTGTTAATGTTAAACGATCGAATATATAGCATGCCCAGACTTCAGTCTGCATTGACTAAGGCAGAGGGTTATCTTGCTAAGCTATCAGCTGATACACCCTATTCTGAGTTTGAACATGAGTAAGCTTCTAGATATATGTGCATCTCCCCCAATTTCTTAATTCCTATTAGGAATGCAAAGTGTTTGACATTTTAAATTATCACTGTTATGCAGCTTTCAAGTAATGGGTTTTGAGAGAGGTTGGGGTGATACTGCAGGACGGGTTTTGGAGATGATGCATCTTCTTTTGGATATCCTCCAAGCTCCAGATCCCACATCCTTAGAGACGTTTCTTGGTAGAATACCTATGGTGTTTAATGTTGTCATTTTGTCCGTCCATGGCTACTTTGGCCAAGCAAAGGTTTTAGGATTGCCTGACACAGGTGGCCAGGTATGATTATCATTAATTTTTCTCTTTCTGCAGAAGTGCGAAGGCAGATTCTTTATTATCATGTTTTCCTTTTTACCTTCTTTTTCCGTGCTTTTCTGGTTACTTTCTTTGTTGATTTTTAGTAATTGATGATAATTTCCTCTTAGTCAGTATAAAGAGAGGAAGTGCTATGATAACTGATGCAAACGGTAAACTTACTTTAGTAGTTGTTATGTGTTGGTTGATGTGCAGATTGTGTACATACTGGATCAAGTGCGTGCCCTGGAGAATGAAATTCTTATGAGACATAAGCAGCAAGGACTGGATGTCACTCCTAGAATTCTTGTTGTAAGTTTATAGGTTTCAACTTCTGCATAACTTAATGTTGCCTACATGTGTTGTATCCACTTAGTCTATCAAGAGACTAGAGCGTATTTGTTATCGGAAATGTAATACAGTTTTCACTTGTTGTCTAAGGTGACACGATTGATACCTGATGCAAAAGGTACTTCATGCAACCAGCGGCTCGAAAGAATAAGTGGGACTCAGCATGCCCATATTCTGCGAGTTCCTTTCAGAACAGATAAAGGAATTCTTCGTAAATGGATCTCAAGATTTGATGTATGGCCTTATCTGGAGAAATTTACAGAGGTATTATTAGCAATAAAGTTGACTGCATTGAAGTGTTAATTCTATTCATTTGGTATCTTCTAAATGAGTGCTTCAAGGCTGATCCTTGTTATAAATTTAAAGAGAGATGATTATTATTAACTTTTTTGTCAGCATTAATGGTGTAAAACAAGAAGATTAGTGATAATAAGAAGGATGTCCTCAAAGCTATAATATGTCATTATTTAGGTATTCGCAACTCGTTCAAAAGGATCTAATTGAATTTGTTTGTTCCTCTAGTGAATGGACTGAATAAGCTTTTTCTTGTCAGTGAATGGGTGGGATTTTTTCATCATTATTGTTATTATTTGGTGTGTGTTTTTTGTGGGTGGGGTGGGGGGTGGGGGTAGTGCTTGCACGAATGTGGTAAGTGTTAGATGAATGCTACTGTATGGCCACCATCATTGTGAATTCAACCAACCATATTTTTACTAATGTGGCCACTGCCACATCTCCCATATTACATTTATTTTTACTACGATCAAAGCTACAAACTTGTAATTGTTTCCACCTAATTATCAACATTGAATTATTGCCGCCGCCACCATTTTTATCTCTGCCATTGCATCGTGCCTAGCACTATTGCTTAAAAAACAAGACAACCATCATGTTGTTAATCAGACACTATTTAGTTTTTCTTTTGGTACGAAGAAGATCCTTAAGTAAATCTTGGAGAACTAATATTTCTTATATCCACTATCTTTGACTTAGCATGCCTATATTTGGAGGATAGTTGATATTCCTCAATTAGAATCAGATAATAAATTGTGATCTTCCCCATTTTATACTGGAAAATAACACTCCCTTGGGCAAAGCAGCAGTGCTAAACTTAATTATATCTGTTTTCTTTATGATTTAGTTGCTAAGCTTATTATATCTACATGCTTCTGTTATTACATTTTGTAATGTTCCTTTTGTATCGTATTCTGTTATCAGGATGCTGCTAGTGAAATTGCTGCCGAGTTACAGGGTGTTCCAGATCTGATTATTGGCAACTACAGCGATGGAAATCTCGTTGCATCTTTGTTAGCTCATAAGATGGGAGTAACACAGGTTAGAAATGATTTTGCATTGAATCAAAGTCAGAAAATTATTTGTGGGGAGTTTAATTTCACACTTAAAAATTCTTGATTTTGCAGTGCACCATTGCTCATGCCTTGGAGAAAACAAAATATCCTGATTCTGACATATATTGGAAAAAATTTGAAGATAAATACCACTTTTCATGTCAATTTACTGCTGACCTAATAGCCATGAATAGTTCAGATTTTATCATCACCAGTACATTCCAAGAGATTGCAGGAACGTAAGCATCTTTGCCTTATTAAAATGATCGTGTTTTTTGTATGTTAATTTTGAAGTCTATGATTGGAATTGGGATCTCGTCTCTCTGTGTGACTGTAAATGCTAGTTGCACCCTTCGACATAAAATAAAGCATGTTCCATGCCTTAGATTATCAGCAAGAGCACCCCAAGATCAGGCTAACTAGAATCATCACACACAACCACCCCATAATCCTAGCAGAAATATTCCAATCCTCACAAATAACTCTATTCCCCTCAGTGCACTTTATCTTCCTCCAAGAGCTGATACAGGACAGTTGATATCCACACCCTTGTGCTGAAAAATTGAGATGATATATCACATCTGCAAAAGAAAGCCTAAACACAGCACCCTGAAAGGATTTTCCTCGCCTATGCTAAGCATCCCATTCCCTTTCAGAAATTGGCACCGTAATATCGCAATACTTGTTCAGCATGTTAACAAAACATTTTATGCTACATTTTACTGTCACTAGTTTGGTTCTTACTAAACTACAGATAAGTTTTTCTAATAGATCTTGCTTATGCTCTCTGATTATATGATTTTTGTGTTACTGTACCTATATGATAGCTTTTTAGTGATGCCTGATGCCTTTACTGGTAATTTGCATTATCTATCTTATCAGGAAAAATACTGTTGGTCAGTATGAGAGCCATTCAGCTTTCACCCTTCCAAGCCTGTACCGAGTTGTTCACGGCATTGACGTTTTTGATCCAAAATTCAATATCGTCTCACCTGGGGCAGATATGTGCATTTACTTTCCATACTTTGAAAAGGAAAAAAGGCTTACAGCCCTACATGGTTCGATTGAAAAGTTGTTATATGATCCTGAGCAAAATGAAGAGCACATGTGAGTTACTTCTTGTTTCTAATTTTCAGTAATCGAAATGTTAAATTCTTTGTGGATGTAAATTGTTATTATTGTTACTACTATGGTGGTGACGACGATGATGATGATGATGTCAATGGTGATGTAAGTTTCCCAGAAATTATTCTTCTAATCATCAGTATCATCTCAAGTCTGCTCATTGCATAAGTCATGTTGTTGGGAAACTCTAGATGTTCATAGGCAGAGACTGGACTAATTTTATCCTCTGCAAGTGTATGCAGAGTACATGGATAATAGATTTTTCATGTAGTTCCTAGATACCTAGTCCTATCTGTCATCTTTAGTCGTTTTTCTCTTCCCATTACAATTTCATTCTCATTGGTCATTAAGGTAATGTACTTCATCTTTTTGGTATGGATTAAAAGCATAAAACTGCATTTACAAGAAATTCTTCACGGGAAATTGGCTTTACAATTACTTATCATTCATCCTCTATCTGACTTGATGCAGTGGAACACTGAGTGATTCATCAAAGCCCATAATCTTCTCCATGGCAAGGCTTGACCACGTGAAAAACATCACAGGGCTGGTAGAGTTCTATGCTAAAAATACCAAGCTGAGGGAACTGGTTAACCTTGTTGTGGTTGCGGGTTACAATGATGTGAAGAAGTCAAATGACAGAGAAGAAATTGATGAAATTGAAAAGATGCATAGCCTTATCAAAGAATACAACTTGGATGGCCAGTTTCGTTGGATATCATCCCAAACAAATCGAGCACGCAATGGTGAGCTCTATCGCTACATGGCTGACAAGAGAGGTGCTTTCGTGCAGGTATAGTGTCTGCATCTATTCTCTGTCATTACTTATACGACTTGTTTTATTAACCTTGAGGTTGTGGGTTTGAAATTGAGGGTAGCCACTTTGTGCACGTAATGCCAGATGTTAGCATAATACCCAATCTACCCCCACGAGGTCACATTAATGGGGATTAAGCTGTGAGCCTAGAATACATATATTGATATGTACGTATGCGGGAGCGACTCATCCCATGCTAACTCTTCCATTTTTTAATCACAGCCCGCATTTTATGAAGCCTTTGGGCTTACAGTTGTGGAGGCCATGACCTGTGGGCTTCCAACATTTGCCACTTGCCACGGTGGTCCAGCGGAGATTATTGAGGATGGAATATCAGGGTTTCATATCGATCCATATCACCCTGATAAGGTTTCTGCAATTTTAGCAGATTTTTTCCAACGGTGCAAGGATGATCCCAGCTACTGGGAAAAAATCTCTAAAGCCGGCCTTCAAAGGATCCTAGAAAGGTTTGTCTTTCTGTACGGAATCGACTGCTCAACAATTATTAATAGGGTTTATCAAGTTGTAACAAATATGAGAAAGCGAAACTAAGTAATGGGCTTATTGAGACCTTCATTACATTACAGGTACACATGGAAGATCTACTCGGAAAGGTTGATGACGTTGTCTGGAGTTTATGGTTTCTGGAAATATGTTTCAAAACTCGAGAGACGTGAAACTTTGCGTTATCTAGAGATGTTCTACATTCTCAAGTACCGTGATTTGGTGAGCTCTCAAATTCTGGTATTAATAATTAATTGTGCTTCAATACAGCATAAAGTTGGAGGATCTTCTGAAATTTTGTATTTATTTCTTTATTTCAGGTAAAGTCTGTCCCTCTAGCAATTGATGGGGAAGACTAA

>AcSUS4

ATGGTGGCCCGCCCAAGGAAGAAAACAAGACGAGAGAAAAGTGCAAAACCATGGCTATGGATACCTTACGACTACTACGACTACTACGACTATTCTATTCGTCTAGTATTTGCATTTTTCAACATCTTTGACAAACAACACACTTCTAGTCCTACCTGACCCCCACCAACATTGAACCGTTCCAAAATGAATGTAATAGATAGACGAATAATCAGTTTACACAAGGGTAGAGAGAGTCCTTGTTTAATAACATATTTTAAAGATAAACATGTTTTTTACGTATATTTTGTGTCTATTATTTATTTATTTATTTTTACTATGTTTATTGTCGTTTGATTAGTAGATTTATTGTAAATAAATTAATTAATTGGCAAATTCACAACTTAAAGGTTAATTTTTCAATCTGTTATCAACCCAAGGAAGGATAAAGTGGATCAACAGAATAATTGTGTAATAGATTAGAGTATAATATACAGTGGGTGGAAAGAGAATACAGGTGATGAACAAGGTGAGAGAGAGAGAGAGAGAGAGAGAGAGAGAAATTGGTGTTCCACCAAAACATAAGTACCAGACGGGCCTGCTAGGGGCATATAAAATGGTAACCAAGGCTCACCCTTTCCACCATCCGAAAGCATAACAATTTCGTTCACTGCTTTCTCTCTCTCTCTCTCCCCCTCTGTTTCCGTCCATTCAATTTCTTTCTCTCTTTTTGATTTCCTTCACTCATTCATTGATCTCTGTTATTTCATTGGTCTGCTTTTTGAAAAGGAAACAAAAGGTAATTAATTTGGCTGTTGCATTGGTAGTTGCATTTTTTTAGAGCTAGTCCAAATTATCTAAAAATCGGTGGCTACGATTTCTGGATATGAAACTAAAAACTTAACCCCGATTTCCAACGACCAAGTCACATGTTTATTACCCACGATTTTAGATTTTGTAAGATTCGATGTTTTTAGTATCTTTCGGGGGAAAGAAATTTCCTTTTTCGGAAGCTTTTTGCACAAACTGATTTGTCGGTATTCTAACCTCTGTTCCGAAGAATTTTCTCCTCAAAAACAATATTGGGAAGATTCTGGTAATCGCTTCCCAGATCCTCCATTTCTTGGAGAGTAAATTACACGAACATGAAGTTTCTTGCTCAAAAAGTTTGAGATCTTTATTATTGTGTAATTTTTCCCTCAAAACATTCCTATTTGAGACGGAAGCTGTTAAAGGTTGCCAAATTTAGGGCAGAGCATAAATCGGACTTTTTCAGTATAAATTCCGTCGCATGCACGCACACTGAATCACTGCACCTTCTTCTTCTTCTTCTTCTTCTTCTTCTTCTGAGTTATCCAGTATCTATATTCTCTCGTAAGTCGTGTAAGTCTAGTCTAGTTGTTTTGGCATGTTTTTCTTTTGACGGATCCTATTTTATTTTTATTTTTCTCTTGCTCTGTTTTTCAATTTTTTTTCTTAGAAAAAGAGGGTTTCTTTGACATGATGCTCTGTTTCACGCAGATCATGTTTGTATTTTTCTAATAGATTGCAGTATGGTAGTGATTAGAGTATTCGGGATGGGTTCTTCACATAAAAAAACACTTGGGGTTTGAAGTTGTTGGATGTTGCAAAAAAATCTAAGATTTTTGAGGACATAAGAGAGAGAGTATTGATGAACCCAGTTGTCAAAAGCGTTGGTTGTCGAGCATTCATCGTCCTTGCCTAATAAAAGTCAATAAGTGGGTGCCGTTGAGTGTATTGGTTGGTTTTTAAATTTTTTAGACGAATCGCATTCTAGATCATCCCAAATGAGGTAGATCATTGACCTTTTGTGGTCAGTCCCGTCTTTTTTAAGCAATAGATTTTGGATAATGCAGTATATCCATATTTATTCTTCAAATTTATTATATAAACTAGTATTTGTTTGTTTTGTTAGTAATAGCAGGTGTTGTTTGATGGGTGTGAATACCTGAAGACATGGCAGGACAAGTCATGACTCGTGTTCACAGCCTTCGCGAGCGTCTTGATGGAACTCTGTCTGCTCAGCGCAATGAAATACTGCTGCTTCTCTCCAAGTATGAAGTATTAATTGCATAATCAGATCACTTCAGTGAAAGAACACTCCCATTTGAGTGGTCTGACATATGGGTTTGGTTTTTTTTATTTATGATATTATTGTAGGATTGAAAGCCATGGCAAAGGAATTCTGAAACCACACCAGATCGAGGCTGAGATTGAAGCACTCTCCAAAGAGGTCCAACGGAAACTGTATGACGGAGCATTTGGAGAGCTTCTCAAATCTGCACAGGTAAATAATCAAAAAACATTGTGCAATTTAATTTCTCTTCTGGATGATTAAATCTTGTGATCTGTTAATTTTGGGTATGACAGGAAGCAATCGTTTTGCCTCCATGGGTTGCTTTTGCGGTTCGGCTGAGGCCTGGTGTGTGGGAATACATGAGGGTTAATCTCAATGCCCATGTTGTTGAGGAATTGAGTGTTCCCGAGTATCTACAGTTCAAAGAAGAACTTGTCGATGGACCGTAAGTAACTCTGCAACTGCTAGTTGTTACTTGGAAGCTGTTAGCATATTTATGGATCATGTTGGTTGCTTGTTGTGGGTGCAGGTGCAATGGAAATTTTGTTCTTGAGTTGGATTTTGAGCCCTTCACCGCATCATTTCCTCGGCCAACTCTTTCAAAGTCAATTGGGAATGGAGTTGAGTTCCTTAACAGACACCTCTCTGCTAAAATGTTCCACGACAAGGAAAGCATGTACCCTCTGCTTGATTTTCTTAAAGTCCACAACTACAAGGGAAAGGTAATTAATCTTTGTGGATTACTCCATTTGATTTAGATTTGGGTTAGGATTGTGGTATAATTTTTTATTTTCTTTGTGCAGACAATGATGCTGAATGACAGAATACAAAACCTCGATGCCCTCCAATTTGTGCTGAGGAAGGCAGAGGAATATCTCCTTACACTCCCTCTAGAGACACCGTATTCCGAGTTTGAGCACAAGTTCCAAGAGATCGGTTTGGAGAGAGGGTGGGGTGACACTGCTGAGAGAGTGCTTGAAATGCTTCATATGCTATTGGAGCTTCTTGAGGCTCCAGATCCATGCACTCTTGAGAAATTCCTTGGCAGAATCCCAATGGTTTTCAATGTTGTGATCCTTTCTCCCCATGGCTACTTTGCCCAAGAAAATGTTTTGGGCTATCCTGACACTGGTGGCCAGGTGCGTGTTGCTTAAACACATACACGCACACAAAAAGAAATCTTTCCAAGTCTTTTGATTTCCCAAATAGTTCAATTGCCTGAAAATGATAACTTGTGTCCTTAATTTTGTTTGTGGCTTACTTAGGTGGTTTACATTTTGGATCAAGTTCCTGCCATGGAGAAGGAAATGCTTAAGCGCATCAAGCAGCAAGGGCTTGATATCATTCCACGCATTCTCATTGTTAGTGTTACTATATGTTCAATTCAAAGCCTTTTCTGCTTAATTTTTGTTTTTTTGTTTGTTTAATTTCCCTGTTTAATGCTTGGTCTGTCTTGCTGTTGTAATCAGGTAACTAGGCTTCTCCCAGATGCAGTAGGCACTACCTGCAATCAGCGCATTGAGAAAGTTTATGGAGCAGAACATTCGCATATACTTCGAGTTCCCTTTAGGACTGAGAAGGGAATTGTCCGCCAATGGATCTCGCGTTTTGAAGTCTGGCCTTACATGGAGAGATTCACCGAGGTCATATTACCTTCTTTATTTAGTTATTTGAATTATTATTAAAGTTTTTTCTTACCTTATCTTTTTTTTAATTTGCATAGGATGTTGCACATGACATCGTCACAGAGTTGCAGGCGAAGCCTGATTTGGTCATTGGCAACTACAGTGAGGGCAACCTTGTTGCCTCATTGTTGGCTCACAAATTAGGGGTAACTCAGGTAATCATGCTTATTGCACTTTGGAGGTGATCTAGATTTCTTAGCTTCATCCATCAATCGACCTACTGACTAATCATGTTTGAAATTGCTACTTCCATAGTGTACCATTGCTCACGCCTTGGAGAAAACAAAATATCCGGATTCCGACATCTATTTGAAGAAATTTGACGAGAAGTACCACTTTTCGTGCCAGTTCACAGCAGATCTCATTGCTATGAATCACACTGATTTTATAATCACAAGCACTTTCCAGGAAATTGCTGGAAGGTATTACTTGGTTATGGTTTTCATTTTTAACTTACTCTTGTTGTTTATAATGCTAATATTGCTACACACATGTTGTTTCTCCAGCAAGAACACCGTTGGACAGTATGAGAGTCATATGGCCTTCACTATGCCCGGACTCTACCGAGTTGTCCATGGTATTGATGTGTTTGACCCCAAATTCAACATTGTCTCACCAGGGGCTGATATGAACATCTACTTCCCTCACACTGAGAAGGACAAGAGACTGACCAAGTTCCACCCTGAAATCGAAGATCTTCTCTTTAGTGATGTGGAGAATAAAGAGCATATGTAAGTTTCAATTTCTTCAATTAGTTTAGTACCTAGTTGTCTTTCTACTCTTATATGCTTTGCCAAAATGCTCATTGATATTGTATTACTTTTGGTGTTCTAACAGTGGGGTGTTGAAAGACCGTACCAAGCCAATCATATTCTCCATGGCAAGGTTGGACCGTGTGAAGAACTTGACCGGACTTGTCGAGTTGTACGGTAAGAATGCCAGACTTAGAGAGCTGGCCAACCTTGTGGTGGTGGGTGGAGATCGTAGGAAGGAGTCCAAGGATTTGGAAGAGCAAGCCGAGATGAAGAAGATGTACGATCTCATTGAAACCTACAAGTTGAATGGTCAGTTTAGATGGATATCTTCGCAGATGAACCGGGTGAGGAATGGGGAACTTTACCGCTGCATTGCTGACACAAAGGGTGTATTTGTTCAACCTGCCTTCTATGAGGCTTTTGGGTTGACGGTGGTGGAGTCAATGACCTGTGGTTTGCCAACATTTGCAACTTGCCACGGTGGTCCGGCTGAGATAATTATTCATGGCAAGTCTGGCTTCCACATTGATCCTTATCATGGTGATCAAGTGGCTGAACTCCTTGTCAATTTCTACGAGAAGTGCAAAGTTGATCCTTCTCATTGGGACGCTATTTCTGAAGGAGGTCTGAAGCGCATCCTGGAGAAGTAAACACCCAAACCTTTCATTTCTGTCGATGTCCATTGCTATTTAAGTTGTAAAGTTAATACAGAAAACTAAAATTTCTTGTGCCTTGGTTTGCTTGGTTGCAGATACACATGGCAGATTTACTCTGAGAGGCTAATGACTCTTGCTGGGGTTTACGGCTTTTGGAAGTACGTGTCTAAGCTTGATCGCCGAGAGACTCGCCGTTACCTGGAGATGTTTTATGCACTCAAGTACCGCAAGTTGGTAAGTGTTGCATTCACTTGTTGAGTGAACCCTAACCGTATAGTAATTGGTATCTTAGGTGATGATTTTGTGTGATGGTGTGTTTCAGGCTGAGGCAGTTCCTTTGGCTGTTGATCAGTAG

>AcSUS5

ATGATTCATATGCTGTTGGAGCTTCTCGAGGCCCCAGATCCATGCACTCTTGAGAAATTTCTCGGCAGAATCCCAATGGTTTTCAATGTTGTGATCCTTTCTCCCCATGGCTACTTTGCCCAAGAAAATGTTTTGGGCTATCCTGACACTGGTGGTCAGGTGTGTGTTTCTTAAACACACACACATAAAAAGAAATCGTTCCAAGTCTTTTGATTTCCCAAATATAGTTCAACTGCCTGAAAATGCTAACTTGTGTCCTTAATTTTGTTTGTGGTTTAATTAGGTGGTTTACATTTTGGATCAAGTTCCTGCCATGGAGAAGGAAATGCTTAAGCGCATCAAGCAGCAAGGGCTCGATATCATTCCTCGCATTCTCATTGTTAGTGTTACTACATGTTCAATTCAAAGCCTTTTCCTTTTCTTTTTTCTTTTTGTTTCCTTCTTTAATGCTTGGTCTGTCTTGCTGTTGTAATCAGGTAACTAGGCTTCTCCCAGATGCGGTAGGCACTACCTGCCATCAGCGCATTGAGAAGGTTTACGGAGCAGAACATTCGCATATACTTCGAGTTCCCTTTAGGACTGAGGAGGGAATTGTCCGCAAATGGATCTCGCGTTTCGAAGTCTGGCCTTACATGGAGAGATTCACCGAGGTCAAATTACTTCATTTAGTCATTTGAATTCTTATTAAAATTGTTTCTGACCTTATCTTTTAATTTGCATAGGATGTTGCACATGATATCGTCACAGAGTTGCAGGCAAAGCCTGACTTGATCATTGGTAACTATAGCGAGGGTAACCTTGTTGCCTCATTGTTAGCTCACAAATTGGGGGTAACACAGGTAATCATGCTTATTGCACGCTTTGGAGCTGATCTACATTTCTTAGCTTCATTCGTGTCAATCGACCTGACTAATCATTTTTTTCAATTGTTACTTCCCTAGTGTACCATTGCTCATGCCTTGGAGAAAACAAAATATCCAGATTCCGACATCTATTTGAAGCAATTTGACGAGAAGTACCACTTCTCATGCCAGTTCACAGCAGATCTCATTGCTATGAATCATACCGATTTTATAATCACCAGCACTTTCCAGGAAATTGCTGGAAGGTATTACTTTGTTACATGGTTTTCATTTTTAACTTGCTGTGTTGTTGTTTATAATGCTAATATTGCTACACACTTTTTTGTTTCTCCAGCAAGAACACCGTTGGACAGTATGAGAGTCATATGGCCTTCACTATGCCCGGACTTTACCGAGTTGTCCATGGTATTGATGTGTTTGACCCCAAATTCAACATTGTCTCACCGGGGGCCGATATGAACATCTACTTCCCTCACACTGAGAAGGACAAGAGACTGACCAAGTTCCACCCTGAAATCGAAGATCTTCTCTTTAGTGATGTGGAGAATAAAGAGCATATGTAAGTTTCTATTTCTTCAATTAGTTTAGTACCTAGTTGATATGCTTGGCAAAATGCAAATTGATATTTTATTACTTTTGGTGTTTCTAACAGAGGTGTGTTGAAAGACCCTACCAAGCCAATCATATTCTCCATGGCAAGGTTGGACCGTGTGAAGAACTTGACCGGGCTTGTCGAGTTGTACGGAAAGAATGCCAGACTTAGAGAGCTGGCCAACCTTGTGGTGGTGGGTGGAGATCGTAGGAAGGAGTCCAAGGATTTGGAAGAGCAAGCCGAGATGAAGAAGATGTACGATCTCATTGAAACGTACAAGTTGAATGGTCAGTTTAGATGGATTTCTTCCCAGATGAACCGGGTGAGGAATGGGGAACTTTACCGCTTCATTGCTGACACCAAGGGTGTGTTTGTTCAACCTGCCTTCTATGAGGCTTTTGGGTTGACAGTGGTGGAGGCGATGACCTGTGGTTTGCCAACATTTGCAACTTGCCATGGTGGTCCAGCTGAGATAATTATTCACGGCAAGTCTGGCTTCCACATTGATCCTTATCATGGTGATCAGGTTGCTGAACTCCTTGTCAATTTCTACGAGAAGTGTAAAGTTGATCCTTCTCATTGGGACGCTATTTCCGAAGGAGGTCTGAAGCGCATCCTGGAGAAGTAAACACCCAAACCTTTCTCTTGTAGATGATGCCCATTGCTATTTTAAGTTGTAAAGTTGTACAGAGAACTAAAATTACTTGTGCCTTTGCTTGCTGGTTTGCAGGTACACATGGCAGATTTACTCTGAGAGGCTAATGACTCTTGCTGGGGTTTACGGATTTTGGAAGTACGTCTCCAAGCTTGATCGCCGTGAGACTCGCCGCTACCTGGAGATGTTTTATGCTCTCAAGTACCGCAAGTTGGTAAGTGTTGCATTCACTTGTTGGTAATCGTATAATTGGTATCTTAGGTGATGATTTTGTGTGATGGTGTGTTTCAGGCTGAGGCAGTTCCTTTGGCTGTTGATCAGTAG

>AcSUS6

ATGTCGACTGCTAAATTGGCGCGAATCCCCAGCATGAGAGAGAGGGTCGAAGACACCCTATCAGCTCACCGCAACGAACTCGTCTCACTCCTCTCCAGGTTTTCTTTTCTTTTCTTTGGGATCTTCTGTGTTTTCCGTTTTGATTTCTCGGTAACCAAACAGAAGTTTGTGGTTCTGTTGATAAAATTTGGGGTTATCTGTGTTTTCGAAGGTATGTGGAGCAGGGGAAGGGGATATTGCAACCGCATCACTTGATCGACGAGCTTGATAAGATCGTAGGCGATGACGAAGCGAATCTTACTCTCAGCGATGGTCCGTTCGGCGAAGTCCTCAAGTCTACACAGGTAAATCTTTCCGCTTTCGGTCGAAACTTTCTTTGAAGCGCTTAGATGCTAAGAAAGTGGACGAAAAAGTAAAAAAAAAAAATCTGAATTATACGTAATTTAAACCCAAGTGGCGTGTGGTTTTGATCTTTCGAAGAAGATTTAATTTCTATTCGTTTCTTTAGCTGTAATCATCCAAACAGAACATGAGTCTTCGTTTCTTTCGTCTGAATCTATTTCATGAGTGGAATTTCAAGTATCAAATTGAAGATTCATGTATTGGGGATAAAGAAACGCGTTTAAGATCGCATAGGATGAGTTAAAATGCTTGTTCTATATGGAATTTTGTCATGAATTTTTCCGGAGTAATTATTACACTTCATTAAATGGATTGATTCTCTAATCACCGTCATCAAATTGTTATATGCGAGACTCTGAATTCCATCTTGAATATGTGGCCAATGACAAAGAAACAATGAAATAAATTACAGTGGCCTATGTAATAAGTTCCCGATCACACCATATGGATCACCGCATACGTCACACAAGATGGGCCCATGTGGGTCCCACGTGTGATCGTAGAAGGACTGCCTCTAATACTATTTCAACCTTTTCTTTCTTCTAAAGAAATACTGGAATTCAGTAATAATTCCAAGCCTTTTGGACAGGAATGAAAGTAGTTTCTGCTGCCATTGTTTAGTTCTGAAGAGAGGAGCTGTGTTGTCTTTTTTTCTCAAAAGATAGTTTCCAAAAAAGTGGGTTTTAGTTTCTGCATTTTGCAGCGTTTTTTTGTTTACTTGTTTTCTTCATTAGAAACTTATTTTGTTTTTGGTTGGTAGGAAGCCATAGTTCTGCCTCCATTTGTGGCTATTGCTATCCGTCCAAGGCCTGGTGTTTGGGAATATGTACGTGTAAATGTATATGAACTCAGTGTGGAGCAATTGAGTGTTTCCAAATATCTTCATTTCAAAGAAGAACTTGTTAATGGGCAGTAAGTGGTTTGTTTCCTTTTGTTAGATTACATTTGCAGAAAAATTCATGACAATTGCGTGCATGTTAGCTTCAATTGGATGAGTACTTCTTTGAATATTTCTTATTTTTTGATTTTAAAATGTGCCAGGGCCTATGATCCTTATGTGCTTGAGCTTGATTTCGAGCCTTTCAATGCAACATTTCCTCGGCCAACCCGAACGTCATCTATTGGCAATGGTGTTCAATTCCTTAATCGACACCTCTCCTCAATCATGTTCCGCAACAGAGATTGTTTCGAACCATTACTTGATTTCCTCCGAGCACACAAACATAAAGGACATGTAAGTTTTGTTACAAAAGTGGTCTTGTTACGAATTTCTTCTGGACATAATTTAATATCTGAACTGTAAAATATATTACCTTTGAATCCAAATCCTGCCTACAGGTCATCTTGAACTAACCATTTATTTTCTTATTTTTGTGCATTACTTAACAGTTATTAGTAAATACCTATCTTTCTGATAAACCTATCTCTGGACTTAACTTTGATATTCCTTTGGCTGCTAAGATGCACAGGGATTGATGATTTTATCTCCCAATTGTTTTACGTAGGTGTTAATGTTAAACGATCGAATATATAGCATGCCCAGACTTCAGTCTGCATTGACTAAGGCAGAGGGTTATCTTGCTAAGCTATCAGCAGATACACCCTATTCTGAGTTTGAACATGAGTAAGCTTCTAGATATATGTGCATCTCCCCCAATTTTTAATAATTCCTATTAGGAGTGCAAAGTGTATGACATTTTAAATTATCGCTGTCATGCAGCTTTCAAGTAATGGGTTTTGAGAGAGGTTGGGGTGATACTGCAGGACGGGTTTTGGAGATGATGCATCTTCTCTTGGATATCCTCCAAGCTCCAGATCCTACAGCCTTAGAGACTTTTCTTGGTAGAATACCAATGGTGTTTAATGTTGTCATTTTGTCTGTCCACGGGTACTTTGGCCAAGCAAATGTTTTAGGCTTGCCTGACACAGGTGGCCAGGTACGATTATCATTAATTTTTCTCTTTCTTCAAAAGTACAAAGATTCTTTATTATCATGTTTTCCTTTTTGCCTTCATTTGCCATACTTTTCTGGTTACTTTCTTTGTTGATGTTTAGTAATTGATGATCATTTCCTCTTAGTATAAAGAGAGTGGGTGCTATCGTAACTTATTCAAATGGTAAACTTACTCTAGTAGTTATGTTTTGGTTGATGTGCAGATTGTGTACATACTGGATCAAGTGCGTGCCCTGGAGAATGAAATTCTTGTGAGACATAAGCAGCAAGGACTGGATGTCATTCCTAGAATTCTTGTTGTAAGTTTATAGGTTTCAACGTCTACATAACTTAGTGTTTCCTACATACGTTGTATCCACTTAATCTATCAAGAGACTAGAGCATATTTGTTTTCAGAAATGTAATACTGTTTTCACTTGTTTTCTAAGGTGACACGATTAATACCTGACGCAAAAGGTACTTCATGCAACCAGCGGCTCGAAAGAATAAGTGGGACTCAGCACGCCCATATTCTGCGAGTTCCTTTCAGAACTGATAAAGGAATTCTTCGTAAATGGATCTCAAGATTTGATGTATGGCCTTATCTGGAGAAATTTACAGAGGTATAATTAGCAATAAAGTTGTCTGCCTTGAAGTCTTAATTCTATTCATTTGGTACCTTCTAATTAAATGATTCAATGCCGATCATTATTATAAATTTACAGAAGTATGATTATTATTTACTTGTGTCAGCATTAATGGTGTAAAACAAGATTAGTGATATTTAGAAAGATGTCCTCAAAGCTATAATATGTCATAATTTAGGTATTCACAGCTCGTTCAAGGGGATCTAATTGGATTTGTTTCTTTCTCTGGTGAATGGACTGAACAAGGTTTTTCTTGTCAGTGATTGGGTGGGATTTTTTCGTTATTATTATTATTATTATTATTATTATTATTATTATTATTATTTGGGGGTGGGGGTGGGCGTAGGGGGTTGTTGCTTGCATGTGTGTGAGAGAGGACGCAAGCCCCTTAAATCAATATCACCGCTGCCACCATTCACCACTTCTACTACCTCACTCCACTACAAACTATCGCACCATCAGCCCCTTCCGTCTAACCATTAGTTTTAACTCCTTAGTGTTAGATGATTGCTACTGTATGGTCACCATCGTTGTGAATTCAACCAACCATATCATTTACTATTGTGGCCACTGCCACATCTCCCATATTACACTTGTTTTTAATACCATCAAAGCTACAAACTTGTAATTGTTTCCACCTAATTATCAACATTGAACTATTGCTACCGCCACCATTTTTATCTCTGCCATTGCGTCGTGCCTAGCATTATTGCTTAAAAAACAAGACAACGATTCTGTTGTTAATCAGACACTATTTAGATTTTCTTGTGGGAAGAAGATCCTTTAGATCAGATACCAGAAGTAAATCTTGAAGAACTAATATTTCTTATGTCCAGTGTCTTTGACTTAGCATGACTATATCTGGAGGATAGTTGATATTCCTCACTTAGAATCAGCTAATAAATTGTGGTCTTCCCTATTTTATCCTGGAAATATCACTACCTTGGGCAAAGCAGCAGTGCTAAACTTAATTATCTCGTTTTCTTTATGATTTAGTTACTAAGTTTATTATATCTACATGCTTCCGTTAATGTTCCTTTTGCATTGTTTTCTGTTATCAGGATGCTGCTAGTGAAATTGCTGCCGAATTACAGGGTGTTCCAGATCTGATTATTGGCAACTACAGTGACGGGAATCTCGTTGCATCTTTGTTAGCTCATAAGATGGGAGTAACACAGGTTAGAGAAGTGATTTTGCATCGAATCAAAGTCATCAAATTATTTGTGTGGAGTTGAATTTCACACTTAAAATTCTTGATTTTGCAGTGCACCATTGCTCATGCCTTGGAGAAAACAAAATATCCTGATTCTGACATATATTGGAAAAAATTTGAAGAGAAATACCACTTTTCGTGTCAATTTACTGCTGACCTAATAGCCATGAATAGTTCAGATTTTATCATCACCAGTACATTCCAAGAGATTGCAGGAACGTAAGCATTTTGTGCCTTATTAAAATGATCATGTTTTTTGTATGTTAACTTGGAAGTCTATGATTGAAATTGGGACTCGTCGATCTGTGTGATTGTAAATGCTAGTTGCACCCTTCGACATAAAATAAAGCATGTTCCATGCCGTAAATTATCAGCAAGAGCGCCCCAAGACCAGACTAACAAGAATCACACACCTACAACCCCATAATCCTAGCAGAAATATTCCAATCCTCACAAATAACTCTATTCCCCTCAGTGCACTTTAACTTCCTCCAAGAGATGATACAGGACTGCTGATATCCACACCCTTATGCTGAAAAATTGAGATGATATATCACAGCTGCAAAAGAAAGCCTAAACACAGCACCCTGAAAGGATTTTCCTTCCTCTATGCTAAGCAGCTCAATCCCTTTCGGAAATTGGCACCATAGTATCCCAGATTCGCAATACTCGTTCAGAATGTTAACACAACTTTTTATGCTACATTTTACTGTCACTAGTTTGGTTCTTACTAAACTACAGATAAGTTTTTCTAAGAGATCTTGCTTATGCTCTCTAATTATATGATTTTTGCATTACTGTACCTATGTGATAGCTTTTGAGAGATGCCTGATGCCTTTACTGGTATTTTGCATTATTTATCTTTTCAGGAAAAATACTGTTGGCCAGTATGAGAGCCACACGGCATTCACCCTTCCAAGCCTGTACCGAGTTGTTCACGGCATTGACGTTTTTGATCCAAAATTCAATATCGTCTCACCTGGTGCAGATATGTGCATTTACTTTCCATACTTTGAAAAGGAAAAAAGGCTTACAGCCCTACATGGTTCAATTGAAAAGTTGTTATATGATCCTGAGCAAAATGAAGTGCACATGTGAGTTACTTCTTGTTTCTAATTTTCAGTAATAGAAATGTTAAATTCTTTGTTGATGTAAGTTGTTATTATTATGATGATAATGATGATGATGATGTAACTTTCACAGTTCCAATCATCAGTATCATCTCAAGTCTGCTCATACTTGTTCTACCATGCTTAAGTTATGTTTTTGAGAAACTCTGGATGTTCCTAGGCAGAAGACAGGACTAATTTTGTGCAAATACATGCAGAGGACACATATAATAGATTTTTCATGTAGTTCCTAGATGCTAGACCTATCTGTCTTCTTTGGACATTTTTCTTTTCCCATCACAATATAATTCTCACGGGACTTGAAGGTAATGTACTTCATCTTTCTGGTATAGATTAAAAGCATAAGATTGCATTTAAAAGAAAACTTATGTGCTGGAAATTGGCTTTGCAAGTAATATATGCAGTTTACAATTACTTATCATTCATCCTCTATCTGCCTTGATGCAGTGGAACACTGAGTGATCCATCAAAGCCCATTATCTTCTCCATGGCAAGGCTTGACCGTGTGAAAAACATCACAGGGCTGGTAGAGTGCTATGCTAAAAATACCAAGCTGAGGGAACTGGTAAACCTTGTTGTGGTTGCGGGTTACAATGATGTGAAGAAGTCAAATGACAGAGAAGAAATTGATGAAATTGAAAAGATGCATTGCCTTATCAAAGAATACAACTTGGATGGCCAGTTTCGTTGGATATCAGCCCAAACAAATCGAGCACGCAATGGTGAGCTCTATCGCTACATAGCTGACAAGAGAGGTGCTTTCGTGCAGGTATAGTGTCTGCATCTATTCTCTGTCATTTCTTATATGACTTGTTTTATTAACCTTGAGGTTGTGGGTTTGGACTCGAGGGTAACTACTTTGTGTAGGTAATGCCAAAGTTTAGGTTAATACCCAATCTACCCCTACGAGACCACATTAATAGGGACTAAGCTGAGCCTAGAATACATGTATGGATATGTATGTAAGTTGGTGCGTGACTCATCCCATGTACTAACTATTCCATTTTTAATCATAGCCTGCATTTTATGAAGCCTTTGGGCTTACAGTTGTGGAGGCCATGACCTGTGGGCTTCCAACATTCGCCACTTGCCACGGTGGTCCGGCGGAGATTATTGAAGACGGAATATCAGGGTTTCATATCGATCCATATCACCCTGATAAGGTTTCTGCAAGTTTAGCAGATTTTTTCCAACGGTGCAAGGATGATCCCAGCTACTGGGAAAAAATCTCTAAAGCCGGCCTTCAAAGGATTCTAGAAAGGTTTCTTTCTTTATGGAATCGACTGCTTATCAAGTTGTAACAAATATGAGGGAACCTGAGTTATGGTCTTACAGAGACCTACATTACATTACAGGTACACATGGAAGATCTACTCGGAAAGGTTAATGACGTTGTCTGGAGTTTATGGTTTCTGGAAGTATGTTTCAAAACTCGAGAGGCGTGAAACCCGGCGATATTTAGAGATGTTCTACATTATCAAGTACCGTGATTTGGTGAGATCTCAAACCCCCTTAGGACTCGTCTCAAATTATGGTATTAATATTTAACAGTACTTCAATACAGCACAAAATTAGAGGATCTTCTGAAATTTGTATTTATTTCTTTATTTCAGGTAAAGTCCGTGCCTCTGGCAATTGATGAGGAACACTAA

>AeSPS1

CGCAATCTCTCTCTCTCTCTCTCTGGCAAACTTACAGTCACTACCGTCCTCATCGCATCACCGACCCCCTCCACCAGAATCATCTCTCTCTCGATACAGTCACTGTCCTCGTCGTATCACCGAATCCCCAACCTGCGAACCAGAAATCTCTCTCTATAGGGACTGGTGATGGCGGGAAACGACTGGATAAACAGTTACCTGGAGGCGATACTGGATGTGGGGCCAGGGATCGACGACGCGAAATCGTCGTTGCTACTTAGAGAGAGAGGCAGGTTCAGTCCCACTCGCTACTTCGTCGAGCAGGTCATCGGCTTCGATGAGACCGATCTCTACCGCTCCTGGGTTAAGGTATATCACAACATTTCTCTCTTCTCTCTCTCTCTGTGTATCAGTTTGTATTGATGTATCGTTGTGTGAATCGATGCGTGTGTGTAATTGTTGATTGTCGGTGGTAGGCGGCGGCGACGAGAAGTCCCAGGGAGGCGGAATACGAGACTCGAGAAACATGTGCTGGCGGATTTGGAATTTGGCTCGCCAGAAAAAGCAGGTTTCTCCCCCCCCCCCCTCTATATATATCTATATATATGCCACAACTTGAAATGATTGTTGCCGATTAAGTGAAGGACTTAAGATTCTTGATGTGGAATCCTGATTTGAATACGTATATACGTGTGTATAGTTGTCTAGGTTTGGAGTGTTAATTGAAATGGTTGTTGCCAATTAAGTGGAGGATGTAAGATTTTTGATACGGAGTTTTGTTGTATACTTGCACATGCACAAGTGAATTGCTGTGGTTTCAATTCTAGGTTTGGCGTGTTATTTGAAATGGTTGTCACCCGAATAAGTGGGGGACTTAAGATCCTTGATGTGGAATTCTGCTTTGGATATGTCTATACATGTGGATACTTGTATGAGCACCAGCGGATTACTGTGGTTTGAATTCTAGGTTTGGAGTATTAATTGAAATGATTGTTGCCAATTAAGTGGAGGATCGTGAGATTCTTGATGCGGAGTTTTGTTGTATACTCGCACATGCACAAGTGAATTCCGTGGTTTCAATTATAGGTTTGGGGTTGTTATATGTTATGGCTGGTGCCGACTAAATGGTGAACTTAAGATTCTTCATGTGGAATTGTGTTATTAATTTTCTGGTCCTTATACTTAATTACGTATATATGCACGAATGAATGACTGTAGTTTGAATACTAGGTTTGGAGTGCTATTTGAATGACTTAAGCTTCTTGATGAGTGGATTTTGGTTTTTTAATTCTCCGGTCCCCGCCGATGATGGGGTGGGTGTGTTTTACTGATCGGCTATTGGTTCATTCTGTGGTTTGATTTTGCTTTGATGATTTGAATGTAATTAACCATCGTTTTACCATTGAACTTCTTTTGGCTTCAAATTTTACAATTTAATTATTGTGTTAAGGAACAAGATGCCACATGTGATTGCAAAAGAAACTTTTGTAGGCCAAAGGGAATGCATTCGATTGTTAGGCTACTTTTTGTTTTTATTTCTGTCATTCTGTTATTAAAAATCAACTTACTTCTTTTGGAGAAGCCTATCTTAGACTCTTAGTGGTGTTTAAAAATTGGAAAAGCCTCGTTTCGTAGCGGGATGCTTTATTGGCAGAAAACCAATAGTGTGTATTTGTCATAGCCATTTTAAGAAGGTCTTTAAACTTTTTCAATGTTTTTGGGTTGTCATTACTAATTTACTAAAAATGACGTGGCTGTAGTACCATGTTTTGTATCTTGCTGGCATTTTAATTTGATTTGCACATAAACGTATTGATGAAGATTTTACCGAGTTCATTCATGTTAAGAGTGATGCTCTAGCCATCTATTTTGCTTTGCTTTGTTTCAACCATTGTTGTTCCAGCATAAAATGTATTTCTTTTTCTACATTTGGAAGTCTATATAACTATATTGAATGTCTTTGTTTTTTTGGTAACTATGCTTCAAAAGGAGGCACCACATTCTGGTATTTGGATTCCTTGAATCTTGTCCTTGTTGGTGAAATTGTGAATAATTAATTCAGTTTTATTTGTTATCTACGTAGCTTGAGGGAGAGGAAGCTCAAAGGATGGCTAAACGTCGTCTTGAACGTGAAAGAGGCCGCAGAGAAGCAACTGCTGATATGTCTGAAGACTTATCTGAGGGGGAAAAAGGAGATACAGTCAGCGATCTGTCGGCTCATGGTGAAAGCAACAGGGCGACGAGGTATACCTATAGGGTTAAAGTCTCACTAAGGTATGTGGAGATGGACACGATGATGATGCATGGTGTGTTCACGTATCAAGCTACATATTATGCAGGAAGACCTGTAATGATAATGGTCTATAAACGTGATGATAAGGCATGAATTTGAATTCCATTCATTTGGCTTGCAAATCTTGTAATGAATTACTATCTCAATTAATCAGTTGTTATTGTGATATTGCATATCGAGTACAAAAATTGGTTATTTAATTGTGTTTTTAACCTGTAATTGTTGTATTAACTTTATGAACTGTAGAGGGGAAACTTTTTGAGGTCTAAACGTTTCCTGTAGTAGGTGTGCACTTTCTCCTGAATGTTATGGTGTAGAAGGATATGGTATCTCTTGCATGAGCAATGTCTTAATTATTCTTAACGGTTACTATCTTGCAGCCTTCATGGTCTAATACGGGGTGAAAATATGGAGCTTGGTCGTGATTCTGATACTGGTGGCCAGGTAAATTGTAGTTGCAAGAGCTTATAAAGTCATTATCCTAATCAATTAAAACTGCAAGTCAATGAATGCGAGGTTGCTTGTGGAGATTGCTAAAGTTGTTTTTTCCCCTTTTCTTTTTTCTCTTAGGTTAAGTATGTTGTGGAACTTGCAAGGGCTTTGGGTTCAATGCCAGGAGTTTGTATCGGGTTGATTTACTCACTAGACAAGTATCATCACCAGAAGTAGACTGGAGTTATGGTGAACCCACTGAAATGTTGCCTCCAAGAAATTCTGATGGTTTGATGGATGAGATGGGGGAGAGTAGCGGTGCTTATATTATTCGTATTCCATTTGGCCCAAGAGATAAATATGTACCGAAAGAACTTCTGTGGCCACACATCCCTGAATTTGTTGATGGTGCTCTTAACCACATCATACAGATGTCCAAAGTACTTGGTGAGCAAATTGGCAGCGGGCATCCTGTGTGGCCTGTTGCTATCCATGGGCATTATGCAGATGCAGGTGATGCCGCTGCTCTTCTATCAGGTGCTCTAAATGTACCCATGCTTTTCACTGGTCACTCACTTGGTAGGGATAAGCTGGAACAGCTTTTGAGACAAAGTCGATTATCAAAGGATGAAATAAATAAGACATACAAAATAATGCGTCGTATAGAAGCTGAGGAGTTATCGCTTGATGCCTCTGAAATAGTGATAACTAGCACTAGACAGGAGATAGAACAGCAATGGCGTTTGTATGATGGTTTTGATCCGGTGCTAGAACGGAAACTACGAGCCAGGATCAGGCGTAATGTGAGCTGTTATGGAAGGTTCATGCCTCGCATGGTTGTAAGTATTCTGATATCAGTTTTGAATCTGTTATGACTTGGTTCTTTTTGTTCTATAGTTAAAGTTATTAGTAACTATACACTGGAATCAACATGGATTGCTCTTATTTCTTGCTTATTACTATTGAGGCAGGTAATGAGGGTAACATGCTGGTCTTGAATTCTTCTTCTTCTGCTAGGGGCTAGACAGCGATAATTTTCTTGTTTGTCAGATCACAATACTTGAGCATGAGTTACAGTAGTTTCAGTCTTGCTTAGCTTGTCAAGATTCCATAAATGAACCTTTTTGTATCTTTGAGAGAACTCAACATGAAGCAACAAACACAGGGAACTCAACTGCGGCAAATTACATTTAATTGCACTGTTTGTACGTAGAGATAGAATTGTAAAAACGAATTTCATGGTGCTTGATGACTTTGATCTCAATTGTAGAATTAAAGCCTTGACTTGTATATAGTAATTGTTAGCTTCATGAATAGTGACAGTATTCATGATAGAGATGCTGACTTGTTTAATTATGTAAATGATAAGTTAACATATGTGCTAAAATTGAACTGAGCTTGAACTTGCGAAGAATCTGGCTGTCTAGATTGGTTCGTTTTAAATTGTGGTCTCAAATGATAACCAAATGTAACTGGCTTGCTAGTTGGAAGTACGAAGACCCGTCGTAAATATACAAGCTTCCCCGCAGGTAATGCCCCCTGGGATGGAATTTCATCACATTGTTCCACATGAAGGTGATATGGACGGTGAAACTGAAGGAAATGAAGATCAGCCTACTTCTCCAGACCCACCCATTTGGCCTGAGGTTCCAATCATTTCTCTGCATTTTCTTTGATGCGTTCATAGAGTGATAACATGACTGCAGTGCTAGTCCTTCGGGATATAACTTTTTCTCAATCCTTGAATGCATTGAGAAGTTCATGCCAATATCTAAGTTGACGTTATGATATGCAGATAGTGCGCTTCTTTACTAATCCACGCAAGCCGATGATACTTGCCCTAGCTAGGCCAGATCCCAAAAAGAATCTCGCAACTTTGGTTGAAGCATTTGGGGAATGTCGTCCATTAAGAGAGCTGGCTAATCTGGTACTTTTGGATCACCCCTTGTTCTGTAGCACCTTTTATTTTGGTTGAATGAATTGTTTTGACTGTACTAAGCTGCTGAATCGGCTTTGACATTAATGTCCTTTGTCCTGTATTTTGCAGACTTTAATAATGGGTAACCGAGGTGATGTTGATGAAATGTCAAGCACTAATTCTTCTGTTCTTCTCTCAATACTTAAGCTTATTGATAAGTATGATCTCTATGGTCAAGTGGCATACCCCAAACACCACAAGCAGTCTGACGTTCCTGATATCTACCGTCTGGCAGCTAAGACAAAGGTACTCTTTTATAGGTGATATGACTTGGCAGGTTGGATATGGATCACTAGTTTAAAGCTGAAATAAAAATAGGACGGGTGTTAGTTAATCAACTTAATCTATTTATAGTACTCTTCTAGTTCCCTATGTGGTACATCAAGACAATTACTTCCCATGATACCTAAATAGCCAGCTCTCTCGGAACCTACATATTGCTTTCTTTATTTTTTTTCTTATATGAGTCTGCTCTCCAGGAATCCTGCTTGTTACTTGGGCAATTCTAGAATCCTTTTTGCTACAGTAATCTATATTTTTTCTACAATGATGAAACTTATCCTGCCAAGTTTTGGTAGCTCCAGGAATCAATTTTTTTTCTTCTTCCATGACGCTTTCTCTTTGGCCATACCTTCTTCCCTTTTGTGTTGTTTTAGAAATTACGAAGGTTTATGTTCATGCACGTGTGCGTCTCTCCTAGTTGCAAGTTGTGTCCATTTGTACTTTTTCTTGCAGTTAAATTGTATTATTTTGTATTTATTGCATAATATGAAGAAATTTATTTTATTTTTTGTATTAGACAATTTTGTAGAAGTTTATGTGGCTACAAAAGATGAGACTTTTGTATGGTATGCTTCTATTAGAGATACATTGGTAGACAAATAGGTTGTTTTGTTCGGACTTTCTGCAGAAATAATAATGCACTACTAGGTAGTAAGCAGGAGCTGTGAATCTGAGCGGGAAAGTACATTCATCTATGAATGGTTAAGGACTTTAAGTTGTACTTGTAATTGAAATGCATGATAATAGAAAAAAAATGACATTTCCAAGTTTTCGCTTGATCTGAAAGCATTACAAATGTGGTTTTCAGTCAATGGACTGGCAATACTAGAAGATGCTGAACTACGAGTTTTATGGTTGTTTGAAAACATGAAATATTTTTCATTGTGGAAAAAGTAACTTTGTCTTTTATTTGACCGACAAAAATAGGTTGAATCTGGGTAAATAATCTGTTTTAAGTTCTTTTCGTCTCTTGTTTTTGTGTAATTGGCTACCATTGCACTGTCCTAGAAATCAACTCAGTGCTGGGAGAATATGAATTATAACACTTGAAGGGAAAGCTAAATAGCCTTTTCTGGGACAAATGAAGCCCAGATAACATATTAGTAGCGTCTTTGATCTCTTTAGTATTTATACCAATAGATGAATATGTCTGATCCCTTTACCGTAGTTTCATCTTTTCTCTCACTACTAAACATGGTTTTAAAAATGGGCAGCATTTGTTACGTAACGTTATGGGTCACTCCCTCTACCCGTTGCGGCCGCTATAGCAGTGACACTGAAATTCATATGCATAACGGCTGAAACCGTTATGTAACTCCCGTTACACCGCATTTATGACAAGTATGACGGTTAAGTAACCATCAGAGTATGACTGAAATTCATATGCATAACGGCTGCAACCGTTATGTAACTCCCGTTACACCGCATTTATGACCGGTATGACGGTTAAGTAACCATCAGAGTATGTCCATTTTTCACTTTTCACTTTTAGCTTCTAGCTTATATTTATTTAATCGACTTTAAAGCTTATAAACATAACTTCTTTTTGTGTGTGTGTGTGTGTGTGTGTGTGTGTGTGTTTCTGTGTGTGTGCAGTTGGAACTTACAAGTTGTATGTTAATTTGTCAAATAGAGTATAATAATGGTATATTTTGCTTTTTATATTTTATTTTCATTAGTTACATTCACCTATTTTGTTTATTTAAAAACACCACATGAATCGCAATTGTATAATTATTAAATAAATAAAGTTCAATAGGACTAATGCATTATAAATTTATGGAATTTGAAACTATTCACTATTTATAGAAAATATCTAAATATCTCTTCTTAGTGATTTAAAAATAATTTGGAATGCAAATAAGATTCTAATAAACCCATGAAAAAATATTTTAATCCCAAAAGAAAATTCTGAAATAAAATCATGCATATCAGGAAGTCTATATGATGATGCGAAAAGCAGAAAAATCTCTGGTTGCGCTACTGTTACGCTACAGTCGTTACACTCTGAAACCGCGTCACCACCACCATTACTGTTACGGTAACTACAACTGATTTTTAAAACCTTGCTACTAGATAGTTGTCTATCGTTCTCATGCAGTCGTTACAATTAGTTGCCGTGTGATTTCTTTTGTCCTGTCCAGTGAAGGCTCTGAAATTAGCTGATCTTATCATACTAGTTTGAATCTTGATGGCATTCTCAGATGATAATAACCTGTAAGTAATGAAATATAAAGGGTTCCTGCCCCTAAAGTTTTCATCCAGGCTTACTAATTAAATACCTTTGTGAATTACCAGAAATGAAATGATAGGAACAAGGAACTCGATCTATATCCCGCAAACATAACTCATAATTCGCTGAAAATTTGTCTATTGTACAGTGATCGTTTCGTTTCGTTTCTCCACGTGTGTCTGAATCCTTTGTATTTTAGATCATTTGATAAGGCATCTTAACATTGCATGAAGTCCTCTGAATAATATAAAAGGTGTTTTTGTGGGCTTTAATTAAAATTGGATTGCTTTCATGGTACTACCTTATCTTATCCAGAGATATATAACTGCATGAATTATGGATTAATTATTTCTTTAATTTGGTCTACTTTTTTTTTTATCGGATTTTGTCATGTTTAAGGTATGATGGTTTTATAGCTGAACACAGAATAAGAGAAAATAAATTATGTCTTTTGCTCAGTGGCATGAAAGCCATTTCATGTGGCTGTTTTTATACAGGATAGCCCGAGTTAACGGTTGTCATTGTAATATAAAGTCATACATGCTAAGGTTGTTTTAGTTCGTTTTCTGCTGTATTTATGTTCTCTCTCTCTCTCTCTCTCTCTCTCTCTCTCTCTCTGACTTATCATATTATTAGATATCTTATTCTGTTGCCAAATTTTGCTAGGGCGTTTTCATTAATCCAGCTGTCATTGAGCCTTTTGGGCTTACTCTGATCGAGGTACACTTATTATACCTGTGAAGCCCTTAGATTTCATTAGTTACTTGACTATAATCAAGTATGACTTCTTTTCCATTGAACAGGCAGCAGCTTATGGGTTACCAATTGTCGCCACAAAAAATGGAGGTCCTGTTGACATACATCGGGTATGTCTACAGATCGGTTCCATTTTTTTTGATCTTCGGGCTTCTATCGATATAATAGTGACGTGTGAAGGTTATGCTGACAACAAGAACTCTACTTCAGATCATTCTGATTACAATTACTGTATATCTGTTGCAGGCTCTTGACAATGGTCTCCTTGTGGACCCCCATGATCAGAAGTCTATTGCTGATGCTCTTTTAAAGCTGGTTGCGGATAAGCAACTTTGGTCCAAGTGCCGCCAGAATGGGTTGAAAAATATTTACCTTTTCTCATGGCCAGAACATTGTAAAACTTACCTATCTCGAATAGCAGCTTGCAAACTGAGGCAACCATGGTGGCAAAGAAGTGACGATGGGGATGAAAATTCTGAGTCGGATTCACCAAGTGACTCCTTGAGAGATATATCCTTGAACTTAAAGTTTTCACTGGATGGAGAAAAGAATGAAGGCAGCGGAAATGCCGATAGTTCTTTAGAATTTGAAGATCGCAAGAGTAAGTTGGAGAATGCTGTTTTGACATGGTCAAAGGGTTTCCAGAAGGGCACACAAAAGGTTGGGCTTACAGAGAAAGCAGATTCGAACATCACTGCTGGAAAGTTCCCAGTGTTGAGGAGGAGGAAGAATATTATTGTTATTGCCGTGGATTTTGGTGCTATATCAGATTTTTCCGAAAGTATTAGAAAGATATTTGACGCTGTGGAGAAGGAAAGGACTGAAGGCTCTATAGGATTCATATTAGCAACATCCTTTACTTTGTCCGAAGTCCATTCTTTTCTCATCTCTGGGGGACTGAGCCCTTCTGATTTTGATGCGTTTATCTGCAATAGTGGTAGTGATCTCTACTATTCATCTCTTAATTCAGAGGATAATCCCTTCGTTGTTGACTTATATTACCACTCACATATTGAATACCGCTGGGGTGGAGAAGGGTTGAGGAAGACTTTGATTCGTTGGACGGGTTCTATCAATGACAAGAAGGGCGAAAATGAAGAGCAGATTGTTACCGAAGATGAAAAGATTTCAACCAATTATTGTTATGCTTTCAAAGTGCGAAATGCAGGGAAGGTAATTTTTTTCTACTTGTGAATGATTTTCTTATGAAGTGGCTTGTTGAATTTACTTTACCTACCCTTAAGGGTGTTAGTCAAACCGAGTAGCGTGAGATTGGCTTGCTCAGTCACTCTTGACTCTGCTTGAGCTCATTTGAGCTCGGTCTTGAGATAAAGTGAGTCAACCTCAATTTTGGCTTGCAGGAAATGTGTTGGAAAGGGTAGAGCTTATATGCTATTACAGAATACCAAAACCTAATGGACGCGTTTGCATTAATGAGTTAACGAGAGAGCATGAACAGTACAAGGCAGCTCGAGAGTGTCAATAACATATATATATATATATATATATATATATCTTACCTTTATAAGACATAAAGTTGGGGGTCAGTGGCGGCATAAGCATAAGACCAACAAAGTCATCGCTTTTGGCCCCCGATTTTAAAAGGCCCCAAAATTTTTATTGGAGTTATGTAGGTTTATAAAAGTTTATAAAATTTCTTATAATTTGCTATAGTTAAAGAGCCCCGCTGCAGTTAAAAAAATGCCCACCAATGAAGACAATGCTAAAAAATAATAACCACTATAAAAAAATCCAAAAGACACAAAACCCCAAAATTTGTACAAACCGTATAAATATCACTTCCAAACGAAAAAATCAATCTATTTGTCCACAACGATGATGCATGAATTTCTAGACAATCACTGGAAAAATCTTTAAAACTAATTATTTTATATTAATTGTCGATCAATTTGTATCTTCACTTGAAAGTAGATTTGAATAATTGCTATTATATAATATTTTTTTGATTTTTTTTAAATTTTAAAAAAATTAAAATCATCAGATGATGACAGTATTCATTAATTACTCAAACTAAAAGCAAGATTCATTTGAAAATACATGCATTGCATATTGCATCTTATTGACAATATCTATTACAATTGCTTTTGTAGAAAGATGGTTTTTGAAATTAAAACTGATAAAATTTAAGATGAACATTGTGACAAGAAAAATTAAGTGGATTAGTTGTTCTATCTATTAGAAATAACTTATGAGCAATACTAAAATACAAAGTCTTAAATCAATAATTTTACATCTAAAAGGCAAAATAAATTTTTAAATAAAATAGTATATAGGCCTCATTTAAAGTTACGCTTGAGGCCTCGCATGTGTCTGAGCCGGCCCTGCTTGGGGTGGGTTGATATTGCTAAATATCATAGGCAATATTCTAGTTCTGGTAAGATAGTTAAATAAATTTAGAATTGTAAAGTTCTCTGCAACTATTTCTTTGTGTGTGTGTATTTATGATCTTTGTGCATCATTGCGTATCATTCTTCTTCATCTTATTATTGTTACATCTTCACAGGTTCCTCCGGTCAAGGAAATCAGAAAATTAATGAGGATTCAGGCTCACCGTTGCCATGTTATTTATTGCCAGAATGGGAATAAGATAAATGTAATTCCAGTATTGGCGTCTCGTTGCCAAGCCCTGAGGTATTGCGCTTATAGTTTCAATTAATTAGTTGTTAGCTTATTATTATTATTATTATTATTTTTGGTGTGTGTGTGTTGAATATTCCATGATATGCTGACTTCAGTTGATCTTTTAATCATTGCACAAAAACCTCAGTATTTTGTTCTTGAAATATGACGGGAAATAGTAATTGTTAATTAGGTCCTACTAATCTTATTTAGGCACAAATAATGAAATTCTACCGTTCGTCCGTGTTCGGTATTGTGTACGCAATTAGCTAGTCATTAAAACCAAAACATGTGATAGTAACAAGGTGCATACAGTTTGAGCACTGCTACAGTCCCATAATTTGGACAAAGAAAAGTACTCAGAATTCAAAATAAATTATTGAAATCCGCTTGGTTACATGGAATCCAAACGCTCAAGTGAATATAAACCAATCATTTTGCATTATGGGTACTTTTCTTGGTTCAAATTCTGAGAACCTAAGGTGTCTCGTACAGTTTTAGAGCAAGTTGGGTAATATAAAACTTAAATGTCACACTATGATTGCACCTCCAACATAAATGCCAAAGATTACATTTTTTATGGATATCTTACTGAGTTTACCCCTCATCTTTTTGGTATGAGGTGTTGTAGTTGTTCCACTTAATAAGTTTTACATATTTTTTAGAAAAGGAGAACTTGACCCTTCCCTAAATTTGTTGTGGATAAACAGCCGTAGAAATTCGGTGTCAATGTTATATATGTCTACAACTAACACACTTAATGATGAGGCGTCCACATTTTTTTTTTTGCAAAGATTTTATGTATGTGAATTTTTATTATAAGATTATCATTAGCCATTTGAACCAATTCATGTTTATATAGCTTGCCAGTTGCCACTATGTGGGAACTTTTGCATTGGATTTAGTTTTAGCAGGATATGGTATCCAAACATTGAAACGGACTTAAAGTTGCCCAATCAGTCATATCGCTGTTAGCAGCAAGAAGATCCACAGCACTCTTTTTAACTGCAGAAACCTTTTTCTCTTTCCTTTGTGGTTGGAGTGCTACTGTGCTGGTAATTGGTACCACTTAGACTGTCAATGTGAGCAAATTTGGCTGTCTAGAAGTGACACATTGTGTGACCTAGAGGCACCATTTTGACCTGATTTGCCATGACATTTTTGCCAGAGTCCAATTAACTTCAGATTTGGAGTGAGTGAGAGAGGTTGCCTCGGTGATTATTAAACACTTCCACATGTCACTAGTTGTGTTGAGCCCGTCAATGGATCCATCCTTTGGCTAGATCACTATATAACCTAATACGATGAGCTCATAGTGTTAAATTTTAAATAGAGAAAGATGAGAAGGAGAAATAGAAGACATAAATTAACGTGATTTGGTCATATTAACCTACGTCTACGGACATAAATGCCCATAAAAGATTTCTTCTTTATTGATAGATCATTGTGTGTCTAATACATAAAATACACACCTATTTATTTAGGTGGAGATTGAATAAAATCACATATTCAATGGTATTGAATGATATCAATAATCCCACTAATTGTGTCTTTGGCCTACCACATGCCAATAATCAAAAGTCAATTAACCCACTAATTATGGGATCTTCAAACTCTTAACACATAGGTTCAGTAGGCTCCTGTGGATGGTGGAGGATATAAATTTCATCACCCCTTTAGTTAGAAGCTCATGGATCTCAATACTATAGCATCAGACATTACGCTGCCTCGTGGAAGATATAATTGTTTAGTTCATATCCTTTTTAAAAAATTAAGTGCGTGTTTTTCTATTGTTATTGTACTTTTTGATCTTCTTAATTTTGAAATAAAAATGGCGAGAATTAAGAAAAAAACAATCCTCTCCATCATTTTCGATTTTATAATGAATCCAGTTTTTAGAAAAGAGAAAGTTAAAACTACTTTCTTTAAATGCACTGTTTCAAAGGATTATCTTGAAATTAGTTTTGCTACAATGAATTTTCTGCATCATTTTTGTCTGTTTCTTTAAAATGAAACCAAATCTGCTTTAAGTTTTTGGGGCAATTGTTCTCCTGATTTGCTGTATCCTTTTTTGTTTGTCGGAAACCTTCTAAAATTGTAGCGAAGTGGGTACAACGGGGATATGTCAATAATAAAGTATAGTTTATATTTTGTTACTATAGATATGACCATAATAGATGCAATTTGTGCTATTCTTTAAATGTTGAAGTTAAAACATTTATCAAGAGGAGTTATTCATGTTTTCTTTCTTATGAAGTGTAGATATGAATATAAGACGTGTAAGAGGCCTTAAGAATATGTAAGTACGATAATCTGAAAACATAAGGTACTTGAAATTCGCTATTTTTATCGTTCTTATAAATAAACCAAATATGGTGCAACTATTCTTGTATTTATCCTCTCAACTGGAGTATAGTCCAATACTGTGAAAGAAATGCTATAATCTCAATATAGTAATAGTTAGGAAATGTGATAGAGTATCTGAAAAGTATCCGATACTTCTCAAACTAATGAAAATAAAGTTTAAATTACGTATACTTTTAGGAAGTATCAGATATATATCTATCCAAAGAGTATCCATATCCGATACGGCTATGGTACGGGTACAATACCCTCCCGGAAGTATCCGTGCTTCATAGCTTAAAACTTGTTATTTTGACTGAGCTGTTTCTTTGAACTTGGTATGAAACGCTGTTACTGATAACCCATTATATTCAGGTATCTCTATCTCCGGTGGGGCATGGACTTGTCAAAAATGGTGGTTTTTGTCGGAGAAAGCGGGGACACTGACTACGAGGGCTTGCTCGGTGGCATACACAAGTCTGTAATATTGAAGGGAGTTTGTAGCGGTCCGACCAATCAACTCCATGCCAACAGAACCTACCCTCTTTCTGATGTCCTGCCGATTGACAGCCCTAACATTGTCCAGGCAGCTGAGGAATGCAGCAGTGCCGATCTCCGGACCTCGTTGTTGAAGCTAGGGTTCATCAAGGGCTAGAAATTTGCACTGTGGCGCTTTGGGTAAGTCTCGTTGTCATTTGGTCCTTAATGCTTCTAGATCTTTAAACTTTTTTTGGAAAGTATTTTCATCATCATTCATGGAAGTTCCCGAAGCTGGTGTTCGGTTTCTTCATGAAAACTTGGTAACAATCCATACTTTTTTGTTTTGTTTTATTTCTTTGGGTCAAAAATCAATCCTTTGTTGGTTCTATTGGCATATAGAAGCCAACCTGTCTTGTACAGCTGTTGTAATTTTTGTTTTGGAACGTATCGTGGACCCATAAAGGATGTGAATTAAACTTTATTATATCCAATTATGAATTTGATAAAGATGCAGAGTCTCTCTCTCTCTCTCTCTCTCACTTAATTTTTATATAAAAAATGATACAATAATTAAATCCTAAAAAAAATCCTGCACCAATTTAAATCTTGAAAATAACAAAATATGGTGTCCAAAGTTATTGGGGGCTCATCCCAAAATGGTTATCTTCAATAGAAATATTTTCTTAGATTCATTGTGTGTGAATCTTTTTATTTATTTTTCTTAATTTTCAAGAATGTATTTTTGCTCATAATCTTTTTTTTATAAATATTTTTTTTCTCGGTCCCTTTTTAACTTTGCTTGATTTCTTTTGGTTATCTACTTTGTTTCAGGTTAAATATGAAATTTTTGATGTAATTAAGGTAAGCTCCGCAAATTCAAATCATATTCAC

>AeSPS2

CCCCCGCCCTCTCTCTCCTCCCCTGCTCTCTCTCACAACACAAACACTCCCTCTCCTACCAAAACTCTCTCACAACACCCCAATTGAGCATTACCACTTCACATTCAGCACGAACCCATAATTTTCCCTCCCATTTTCTTGCGTATTTTCTCGGGAAAATCCGGGAATGGCCGGGAACGAGTGGATAAATGGGTACTTGGAGGCGATTCTTGACAGCGGCGCGGCGGCGATTGAGGATCAGAGCAAGCCAATCCCCGTGAATCTTAGAGAGAGAGGGAATTTCAATCCCACCAAGTATTTTGTCGAAGAGGTTGTTACTGGGGTCGATGAGACCGATCTTCACAGAACATGGATCAAGGTGGTTGCGACTCGCAATACTCGCGAGAGGAGCTCCAGATTGGAGAACATGTGTTGGAGGATTTGGCATCTCACTCGCAAGAAGAAGCAGGTTTCAAGATTTCACTCTCTATCTATCTTTTAATTCCCCTAACAAAAATCCATCTATCTATCTATATGTATATTATATATGTATATATGAAATGTCTTTTTGTTTGTGTATGTGGGTATGTGTGCTTGTAGGTAGAAATGGTGTTTCTTATTTGGATTTTTGGAGAATTGGGTTTTTCTTGAATTCTGAATTATCTCTCCCCCCACCCACACCCCAACCCACACCCACCTCTCTCTCTCTCTCTGAAATGTCTATATGTTTGTGTATGTGGGGTATGTGTGAAAATATGCAGAACTGGCGTTTGATATGGAGATTCTTGGTTTTTTCATGAACTGGGTGTTTCTTATAATCCATGTTTGTTTAAGTTCGTGCGTAGAATTGGCTGATTGTCAATTGGTGAATATGTACTTGTTTGTGGGTATGTGAGCTTGTATGTAGACCTGGTGTTTGTCATTGGGTTTCTTGGATTTCTTGAGAATTGGGTTGTTTTTACTTTGTTATTAGTATAAATTGTGTGGAATTTGGTTGATTAACAATGGATTTCTTGAATATTAGAGTCTATATACATGTATATTTTTTTCTCTGTGTGGTTGTTATTGAGCTTCTTTAAGATTTCAAGAATTGGCTTCTCTGTACATCAGTATTAGTATAAGTTTTTGTGTGGAATTGGGTGATTATCAGTGGGTTTCTTGAATTTTAGACCATTCTCTTCCTCTTTTTTTGCATATAATATTATATATGTCTCTCTCTCTCTCTCTTTCGGAATAGAGGCGCCTATGTATGTTTAAGCCTCTTTGTGTAAGGAAAAAAAAAAGACTGGAGAAACTTCTAGGCCATTTTTAGATAAAAATTTATAGAGGGATTTGTGGAGAGAAAATAAAAGGTAAGAGAAACACGTCTCAGACTTGGTGAGAGAAATCTGTTGCATAATCTTTTCCACCCTAATGCTGCTACCACACACATCTATATTATAAATCAAGACTTAATTTGAATCCAAAATCTATCTCTCGCTATCTACGGCTCAAATCATTAAGTTTGAATTCAAACGTCTGAGATTTTCACCTCAAATATTTTTTTTCTGCCCAAAAACATCACTTTCACTCTCTCTCTCCTCTCCTCCCATCACTCTCACTTTCTCTCTTTTTGGTGGGACCTACACACCTTTATTTTTCTCTCATTTTAATTTTTTATGTAATATTTATTTACTATTTAATCAAAATTGAATAATCTTTTCATTATTTTGACAATAAGAGCCTAAAAATATAAAAAACTTATTTTATTTGAATATAAAAATAAATTATTATTTTTATGAATTTTCCTCTTAATTCGATAATTTTTTTATTTTTAGATCTTTTTATTAGAGAAGCCGAAAAGGTATAAATATTGATTAATTGGTGGAAAATCACTAAAAAGCATACAACGAAATTACTTCAATAAACTGAAAGTTAGTCCAATTTTCTTTAATTGTCCTTTTTTTATATTTATTAAATTAAATTTGTCCAATGATTATAGTAAAAATATTTGATAACAATAATCTTTAAATAATTTATCTATAAAGACAAAAAATCTATAATATAAAATAACATATTTTTATATCTTTTAAACTAACTATCTTATATAAATCAGGACTTAGTTTGAATCCAAAATCTATCTCTCGCTATCTACGGCTCAAATTCATTTAAGTTAATCCAACTACTGAGATTTTCACCTCAAATAATTTTTTTTCCCAAAAACATCACTTTCACTCTCTCTCTTTCCTCCCATCACTCTCACTTTCTCTCTCGATCATAACTCTCTCTCTTTCTGGTGGGACCTACTCTCTCTCTTTCTGGTGGGACCTACGCACCTTTATTTTTCTCTTATTTTTAAATTTATTTACTATTATTTTTTACTTTCACTTCTAAATTTTTATATAAAAAAATTATAAAAAATACATATAATTTATTTTAGAGGAGAGATATTGGATTAGAAGTTATGTTCTTATGTAATATTGAATTTATTACTGTATTGATATGTTAAAAGTTATTATTTTTTTAAAGTATTTTTATGATAAATTAAATTACTGAAAAATACATCATTAATTTTTAAGAATAATCTCAAATAACATATAACTATCAACCCATCAAATTTTTAAAAAATATTAAAAATTTTGATCTATATTTTTGTTTATGTAATAAAAAATCTAGGGGGATTAAAAAAGCTATAAATTTTATTATAATTTTTTTTATATAAAAAATTTAGAAGTGAAAGAGTAAAAGAAAAAAATAAGAATCAAGGGGTATTAAAAGAAAAAAAAAATAAAGGGGTTAAAAATTTTATCATAATTTTGGTTATATAAAATTCAGAAGTTCCAACGTTAGAAAAAAAGAATCAAGGGTATTAAAAAAAAAGAAATAAAGGGATATTAAAAAAAGAATATAAAAGGAGTTGAATGACAAAATGTAGTGTGGGAGAATTGAGGGGTTAAAAGTTGAAAGTCAAAAAATAAAAGTGCAACATTAAGGGGGTAGGTTGTCAGTGGGGGGTGAAAAAAAACGGCTCTCTTTGTTAGTTTTAGATAGATTGATTTAGTTAGTTTTCTGAGAAAGTTACTGAGAGCTACCTTTAGTTGTGGATTATTTTTATTTTATTTTTATTTATTTTTCTATGATGAAGAATCATCAAAATTGATGAAAATAAATTTTTTTTTTTTTTTACTTAGTGCCTATGGACACGGGTGGGGTTAATTACTAGTATTTGTATAAACCTTCTTTCATGGGTGTGTAGGACCCACATAGTCATAATGTGCAGGCTCCACACCTATGTATATGTGGGTGTGCAAATGTTTTTGTTTGTAACGTTTCTCTTCTTTTCCCTCCACGCTTGATCCTGACCTTTAAGGGATTTTTTTATTTTTTGACTCTCTCTCTCTCTCTCTGAAATTGTTGACTACTGATGGTGTTTAGTCAAATTACAGAGAATTGGAAAGCCTTAATGGGATTCAGTGCACTTGTGTGACTTTGTGAGTGTGTGTTTACACATTTAATTAGGGTGGTGACTTATAGTGGGGTTTTCATGACTTTTCCTAAGAATTTGACAGTTGAAGGGTCTCTATCACCATATCTGGAGAATTAGGTTCATAGTGAATACATGACTGCCTTGATATCCCTATTAAAGATGAATGAATACGCTTGCTCTATTAGGTATATTTTTTATGGGTTAATATGAATAAGCTTTTTTTCTAACAGTTTTTCACAATAAACTCTTTTAACTTTATCCACCAACCCTCAAGGAAAGATCTTTCTTTGATGAAAACTTTCTTTATAGTTAAACTTCATTGAAACTATTTCTATAAAGACGTCCTAAAGGAAAAGGCACAATTCAATTCAGCAGTTGCCGCCAATAGTCTAGAAGGATTCACACTATCATACAAGCTACCAGGAACTTTCAAAATTAAAAAGGTTTGACTTTGAATTAGAAAAACAAAAACCTTTTAGTTTTAGAAGTTCCTCCACGTCGCACTTTCGTTCATTTTATTTTAAGAAATTGATATTTCTTTTTTCCGAATTGCCGTGCACCCCGCAGATGATGTGGCAGATGCTCAGGTTGAATCCATAAACATGTAAATTATATAGAATTTTTCATCTCATATGATGATTGAGGCAATTGCCTAGTATAATATAATATGGAGGTTGTTAGGTGTTAAGAAGTTTACATGTATATAAATATGATTTGGTACTTCCCTCACGTGCCACATGAATAACAAAAAATCAAAACCAGGAATGATATTTGATTTGATCTGAAACTTGTGTATGCTTTTAAGAGAAAATGCTATAAAAAAAAATTGTTTCTAACATGTATTCCAAATGATTCCATGACTAAATAGTGGAGCTTTTTTTGGTTTTATGTCAGTTGGAATGGGAGGATTTCCAAAGGATGGCACACCGAAGATGGGAACGTGAACAAGGACGCAGGGATGCGACAGAAGACATGTCCGAAGACTTGTCTGAAGGAGAGAAGGGAGATGTACTTGGAGAAACGATGGTGAATGAAAGTCCGAGGAAAAAGTTCCAGCGTAACTTTTCCAATTTGGAAGTATGGTCGGATAATAACAAGGAGAAGAAGCTTTACATTGTTCTTATCAGGTACAGTTCTTTATGTATGTTCATCACCGTATTGGATTTGAACCACTCTTTGGATATCAGTCACAGTCACACCACTGATCTATTAATATTGAAAGAATATCAAGTTGACAAGATTTTAGGCAGCTTCCTTTTAATCATTATTAGAAAGCTGTCTCTGAGTTTAACAATTTTATTCAGCATCCCAAGTCTTATTTACTTAATCTGTAGAGAAATTGACTTCACAAATAATGGTTTAAGCATTCACTGAAACGATATCTCTTATGCTTGCTTTTGTTGCACAGCGCAGACTTTCCAAATATTGTAATAGTTCTAGGTCATCTAAAAATGACAAGGGTTCTCACTTCTCATCTTTATGAGGTGGGTCTTTTTAGATAGCACAAAAGACTAACTTATTTACGCAAAGTTTCTTGTTAGTCAATTCGACGGTGGCACAGGTCAATTGATAACGTAAAGCATAGAAATATACAAGTTTAGGAAGAAATTCATTGATGCAAAGAATACATTTTTTAAGTCACTTGTTTTAAGCTAGTTACGGAATTAAGTAGAATGAAGTTTAGAACTCAAAATCACAATTGATCTTCTTGACCAGTTTGCACGGTTTGGTCCGTGGGGAAAATATGGAGCTTGGGCGTGATTCCGATACTGGTGGTCAGGTAAATACTTAATTTTTCTTTAAACGAACAATACTTTAACTATATTTTGTTCTTCTTGTTTGCTTTTTTATCTAGTATCTCCAGTTTTCATGACAATTCCAACTCCAGATCAAATATGTGGTAGAACTTGCTCGGGCCCTGGCTAGGATGCCGGGGGTATATAGGGTCGATCTGTTCACCCGCCAAATATCTTCTCCCGAAGTCGATTGGAGCTATGGAGAGCCCACAGAGATGCTCACCGCAGGTGCTGATGACGATGCTGATGTTGGAGAAAGCAGCGGGGCTTACATTGTAAGGATACCCTTTGGTCCACGTGATAAGTACATGAGAAAAGAATTGTTGTGGCCCTATGTTCAAGAATTTGTAGATGGAGCTTTGGCTCACATTCTCAATATGTCAAAGGCTTTGGGTGAACAAATTGGTGGGGGGCAGCCTGTTTGGCCATACGTAATTCATGGCCACTATGCAGACGCGGGGGATAGTGCTGCTCTTCTTTCAGGTGCTTTGAATGTTCCTATGGTTTTAACAGGACATTCACTAGGTAGAAACAAGTTAGAACAGCTTCTCAAGCAAGGAAGGCAATCAAAAGAGGATATCAATTCTACGTATAAGATTATGAGGAGGATAGAGGCGGAAGAGCTTTCCCTTGATGTTGCAGAGCTTGTTATCACGAGCACCAAACAGGAGATTGACGAGCAATGGGGGCTTTATGACGGGTTTGATGTCAAGCTTGAGAAAGTTTTGCGGGCACGTGTTAGACGAGGGGTCAATTGCCATGGACGATACATGCCAAGGATGGCGGTAAGGAGCTGTTTTGAACATCTTATTGATCTACTTGTAGAGAAAAATTGGAATGTTGGAAATTTGAGAAAAAATCATTTTGTTTTGGAACGTGAAGACCCCATCAGATCCTTAATGAACTTCACTTTCGAATTGCCTTTCCCAACAGACGTAATATAATAACATATTCTACAACTCCCCTTTCACCTTTACATTCTTGAGAAGCAAACTATCTTGTAAGTATGATCTTGGTTGCTTATTTTGTCTGCTATCAGTGTGTAAAGGTCTATTGCAGCTAATTTTCATTGGTTATATCTTTGTGGGGAGTGAGTTCTATTCTGTTTATCCGTGAGAGGGCCCCATTTCATATGCACATATATTAGAAGTAATATTAAAACTAGAAAGCTCTTTTGATTTGAACAATTATGTCAGCATGAAGGCCTGAGAGAAACTGAGTTTCCTTTCAATTCAAAAACCGCCTTACATGTTGTTTTCCTAAACTAAATTCTAATTTTTCCCAGATCATCAAGTCATAATGGTAATTTGAAACTATGAATATCCTACCAGATATTTAATAAGAGATTCGTATAGTATTGATTTGAATAACAACATGCAAGTGAACAATGTGTCATCTGCCAACAATGCTTATTGTGGGTAATCCTGATTGAATTACATTGATCTTTCAATGTCCAGAATAGCCCTTTGTAACGAAATACATTTACTTTTCAGGTTATTCCTCCCGGAATGGACTTCAGCAATGTTGTGGTTCAAGAGGATACCGCTGAAGCTGACGGGGAGCTGACAGCACTAACTACTGCAGACGGGTCTTCTCCAAAAGCTTTCCCACCAATATGGTCAGAAGTGAGTATCTAATTTCTATGCTTCCTCGTCAATACCCATCTTTGCTACTTTTTGTAACTAACCAACTGAACTTTCTTTGCAGATGATGCGTTTTCTAACAAATCCCCATAAACCAATGATCTTGGCCTTATCAAGACCTGACCCGAAAAAGAATATCACCACTCTTTTGAAAGCCTTTGGAGAATGCCGCCCGTTGAGAGAACTTGCTAATCTGGTAAGTAAGGTCTCACGTTTTCTCATTGGACCACCAGAGAGTAATCAAATGCCTCATGTCTCCTAACATTTTGCACAGACGCTTATAATGGGAAATAGGGACGATATTGATGAGATGTCCGCTGGGAACGCTAGTGTCCTCATCACAGTTCTGAAACTGGTTGACAAATATGATCTGTACGGGCAAGTGGCCTTCCCAAAGCATCACAAGCAAAGTGATGTTCCGGAAATCTACCGCCTTGCTGGGAAAACTAAGGTTCGTCGAAGTTCGCAGTTTTCATACTTGCTTTACATGGTTTCATTTGTGTTGGTTTTACTAATTCCTGCAACTATTGCAGGGGGTCTTCATAAATCCAGCTTTGGTTGAGCCTTTTGGGCTTACCCTAATTGAGGTTGGTTTGAACTATTTAGGTTTTTTTCATCTTTTATAGTCTTATCTTAACTTGGTCTTGAGACGGTGGTTCAAAATCCATTATTTTTATGATGTAGGCTGCAGCACATGGACTTCCAATGGTGGCAACTAAAAATGGTGGTCCAGTTGACATTCATCGGGTAAGAATATCTATGCTCCACACCCACTAATTTCTATTGGTTAAAAATGTGGAATTGTGTTTCACTCTGTTTGATTGCCAAGAAGACAAAAGGGGAGGTAAACTCTTAATAGTTGATGGCAGTTAACGGGGGTCAATGGATTTTCTACATTTTTCGTTGTATCCTTCGCAAGTCCCAACTCATTTGAGTAACATACAAACAATGATTTGATTTCCTAAAAGTGCTCTTAGTATTTCTTTTTGTTGTTTCAGGCTTTGAATAATGGTCTGCTTGTCGACCCACATGATCAGGAAGCAATTGCTAATGCGCTGCTTAAACTAGTGTCAGAGAAGAATTTATGGCATGATTGCAGAAAGAATGGTTGGAAGAACATACACCTATTTTCATGGCCCGAACACTGCCGCACATACTTGACAAGGGTGGCAGCATGTAGAATGCGACACCCTCAGTGGCAAACTGACACCCCGGCAGATGAATTTGCCGCTGAAGAGTCCCTAAATGACTCACTCAAGGACGTGCAAGACATGTCCCTGAGGCTGTCAGTTGATGGGGAAAGGACATCACTTAATGAATCGCTTGACCATGTAACTGCAACTGGTGGTGGCCCTGAGCTCCAAGACCAAGTGAAACAGGTACAAGGATGGAGCCAATATTATAGTAGGTTTGTTTTTTTTAAAATTTAGTACCTGTCCCCTTAACGACATTTTTTTCCCCAGTTTTATCGATTGTATGTTCATTGTTTTCCGAAATAAAATCTGTGTCCCCTTGAACAGGTAGTAAGCAGGATGAGGAAACCAGAAACAAAAGCTCAAGATTCCGAAGGCAGTGGGAAACTTGTTGATAATATTGCAAGCAAATATCCGATGTTGAGAAGGAGGCGCCGGTTGATTGTCATAGCACTTGATTGCTACGACAGTGATGGAGCCCCAGAGAAAAAGATGATTCGGATAATTCAAGAGATATTTAGGGCTGTTAATGTAGTCTCTCAAACTGCTAGGTTCTCGGGATTTGCTCTATCGACAGCTATGTCAATGTCGGAGCTGAAAGCCTTCTTGAAAACTGGGAATATTCAAGTAAATGAGTTTGATGCTTTGATTTGTAGCAGTGGGAGTGAAGTCTACTACCCGGGTACTTATACACAAGAAGACGGGAAGCTTTATCCCGACCCAGACTATGCCACACATATTGACTACCGTTGGGGTTGTGATGGCTTGAAAAAGACTATTTGGAAGTTGATGAATTCACATGAAGGCGGGTCTTCTCATTCTAAAAGCCCAATTGAAGAAGATGTGAAATCTAGTAATTCGCATTGCGTCTCCTACTTGATAAAGGATCTCAGTAAGGTGCGCAAAAGTATTGGATGCAAGCTTTGAAGCATAAGAAGTAACTGTTAGTATCGGTTACATTATATTCATGATGGTTGGATTTTGCAGGCAAAGAAAGTGGATGATATGAGGCAGAAGCTGAGGATGCGGGGTCTCCGTTGCCATCTGATGTACTGCAGAAACTCAACAAGAATGCAAGTTGTCCCTCTTCTTGCATCTCGAGCACAAGCACTGAGGTAATATCTCATAAACTAATCGGATTTTTGGACGCCCCTATGAACTTGGGGTTTAGAGTGTGCCAATTTGAGGTTCTCCAGCTTGCAATGTTTCTCAGATCTTTCTTAGGAGATCACTTTTGGAAATCATGCAACTGCTAGTTCTAAGCAAATTGGGTTCACTTGATTGAATTCAAGTGTTTTCTTCTTTAAACACTCTGTTATGGTGTCTAAAGAGACATAATGTACTGGTTCCATTCCTTTCAGTCTTGTTCACGCTTAAAAGGTGCTGGACCCTCTCTCCCCAAAGCCTTTTGTCCCAATCCACTTCCAACAACACAATCTTACCTTGAGATCTGTGTGTTTCGAGTTTTCCACCCATCTTGCGACCGTGAGTGCATTGGGACATGGGACAGAGTTTTAAGAGAGAAGAGGATCGCTAGCCCACTTGAAATGCTTTCAATCCATTTTCCATTGCATTTGAAACCATCTCTTTGATTGCGATGTGGAATCCTTTTGCCTACTCATTTTCTTTTCGTAGTCTGTCTTTTTCTCTCATTAGTAGTGTGATGTAACGAAGGAATTTATTATTGATTGGATTGTCTAATGAGTTCTCTCTTCATGGTTGTAGCTCTTATCTAGCATGATTTGTCTTTCTGATTGGCTCGTGTCTTCTCATCTTCTGGTATCATTAGGTACCTTTTTGTTCGTTGGAGACTAAACGTATCTAACATGTATGTGATTCTCGGTGAAACTGGAGACACTGATTATGAGGAACTGATATCTGGCACCCATAAGACGCTAATCATGAAAAACATGGTGGAGAAAGGTTCCGAAGAGCTGCTCAGAGCAGCAGGAAGCTATCTAAAAGATGACATTGTCCCCGAGGAGAGCCCACTGGTGACCTACACAAGTGGAGAAGCGAAAGCCGATGACATTGCAAATGCTTTGAAGCAAATCTCTAAATCTAGCCCGGGAATATGAGATAGTAAGCTCTCTTTTTTGTGTGTTACGCGCGTTACAAGTCACGTGCTCGTTCTTTTTGGACGGTTGTAAGGGGATCCGAAAATATGCATTTACAAGCATTTTCTGACATGGGTTTTAGCCAAATTGTAGGTAGAAATGGTTCCCATTGTTGGAAATGCTTTTGTTTCTAGGTATCATCTACTAAAACTGCTATATAGCTTCCACTATGCAATGTTCTTTCTCTTGAATATACATGAATTACATGAAATTGCAAGGGAAGTCTTATATATTTCTGCATATTATTGTGG

>AeSPS3

CAAATCCCTCCTAACCATAACCACCTTCTCCTCTCTCCAAACTCTCACTCTACCTTCTCTCTCTTCTAGTTGCACCTTCTACAACAATATTGTTTTATTCATTTTATGTATACATCTTATTGTTTTAATTTGTGAAGTTATTTTTGGAAACGAAAACGGGGAGAGAGATGGCGAATAACGAGTGGATAAACGGGTACTTGGAGGCGATTTTGGACGCGGGGAGTCGGAGAAGCGGATTGAGAGAGAATGGCTATGCTGATGAAGGAATTAGAAGCAACAATAGCATGAACAACATGAGTATAAGGAAAAGGTTTGAAGACAAGTTGAGAATTGAAAAGTTCGATGATGACAAAGGAAAGGAAGAGAAGCTGTTTAGTCCCACCAAGTATTTTGTTGAGGAAGTTGTTAATTGCTTTGATGAGTCTGACCTCCACAGGACATGGATCAAGGTATGTTTCTAAGCAATCCTCCCATATATACGAACATATATGTGTCTCTATATACACACACACATATGTGTATATAATATATATATATATATTAACGGAGAATTTATACGTGACTCGTGTGCAATGTCTGTAACAGTTATTGTGAACGTGCTTGTGAATCAGTTACCAGAATAGAATAATTTTTTAGTTTTATTAAATAAGGTATTTTCTTTTAATAATAGAATATATAAAAGAGATATCGAGATGAAACGCAAACCAAATGTGAAAAGTGAAAAAGGAAAAAAAAAATTTTAATTCAAAATGGAACTTGAACTTAATAAAAATAGTTCATAATTTTTTCACCTCTCAACTTTATTAGCAACTTCTTTTTTAGGTATATATACTTCCTAACAACAAACAAAAAAAGCTTTTCTTTGCTTTCTCTAGAAATTGCCCAATTAATTTCCTTCAATTCTTCTCCACAGAAGAAAATAAAACAAATAATTAAAATGAATTAGTCCAATAAAATAAAGAAGAGTGCATTGTGTGGAAGGGGCTCCCTCCAAAGTGACAAACTTTTTGAAATTGATGCTCAAAACCATATGCCATGCACAAAAAAACAATATGAAACTAAGGATTAGAGATTGAATTTTTTGGAGAGAAGAAGAAATTAAAAGCAAAAATTTAAAATATAGACGTTAATCCATTACCCAACTTCTCTCTATTCTCGTTAGCTAACACACGTTTTTTCTGGTAAACCTACCAAGTAACCCCGACTAAACTTCTTCACACCCACAACCTTTAGCAATAACCTCAATTTGTTCTATGCACACCGCAAACTTCTTCTCCACAAAACGACCCGATATCAACATACACTTTGTCGCCTCTTATCACCCACTTGACTGTCTCCGACACCTAAACCGAATCCACTTCCACCATGCACACACCCCCAACCACGCAAACCAACCAGCCATCCATCTACTTCACTGGTTTATTTTTTTCGACTCACCACTATAAATCGGTTCATAATCTAGGATTATTGTTTCTTCCAACATTAGCGTGAGACAGACGAGGAGAGAGAGAGAGAGTGGGTGCATAGGATGGGGAGTTGAGCGTGGCTACAGCGGAATCTATCTCATAACGCTTTTCGTGCGGCAGGGGTGGCAGTTGGGGAAGGGACGTGGCTGGGGATGCGATGAGGAGGGGGGCAGGGGCTGGGAGGGTTCACACAAGAGGGGACTGGGGTGGGTGTGGGCCAAGGCTGGTGATGGTGAGGAAGAGTGGGGTGGAGGTGGGAGATTGGAGGTGCGGCAGCGGCAGTCGTGCAGGTTTCTGGCGACTACGAGACGGTCTGTGATGAGAGATTGATTTTTGAGCCTAGAGAGAACAAATTCGAGTTCAAATAACATCTCCTGAGCATTTTAACTCCTTAAAAAGTTAAATTCTCCTAAAATTGGCAAATAAATTGGAATAGAGGATTCTTATTTGGAAAAATTATGGTACATCGCATTTCTTGCAAAAATTCTATAGCCGTTGGCACTGGTGGAAGGCTGTTTTGATAACCCCACGTACTGCGGGGTCCAGACAATTTGGAGGCACTATAACGGGTTCATTGGAACCCCAAAGGCCACCATCACTCGCCATCCCTCCACCATATCATTTTTTTGTCCTCTTTTTTCTTTGGAAGTATATATGCATTTGAGGCTTAGTTGAGTCTATTACTTGAATTGAGATTACAACTACAAAATAACTTTAGTAACCCAAATCATTAAGGTTGTGATTGGACCAATTTAAGTAAGACAGTCATATATAAATAGCGTTGAGAAACAAGCTAAAGAGTTGAACAATATATTATTCAAGTTTGTTTTAAAACTTAACAAATTTTAAAAATATGAGAACCGAACTCGCGTAACTTGAGTTTTTTAAGCATTATAAACTTATCGAGTCAATTAGTAGATTATTCAAACTTGGTTTGAAAGTTTAAAAACTTTTAAAATATTTTCGAACTTGATTTATTTATAAAGCAAACTGATCTTAAACGAGTTCTTAATGAGTCGATTACGAGCCAAACTCAAAATAGCTGAATACATTTATCATCCTACTTACAGAAGAATATAAAATAATAGAGAATGATGCAAAGGTGAATAGCAACTTAAAAACAGCAAATGAAAGTGCTAGTTTCTGATATAAAGTTGCAACACCCACCATCCCCACAAAAAAAAAAAAAGGAAAAATCTTAAAGTTTCATAGTAACTATTTGGGAAACTTAGACCTAAAACAGTTGCGCTATTCCTGGATGGGGTTTAGAGACGTACTAAATGAATAGCTTCTGCTAAGCTAATAGCAACTCCAACAATATTATTTTCTTTATCTTTACTAATTATTTTTCATTGCATCTTCTACACAATTATCAACACCCCTGCATATACACAGCCGCCGCCATCGGTGCTCCACCAAACCCGCCATTCCTCAATCCAACCAAACCCAGCACACACATACAGAATCAAACTCTTTGAATGTTGAATCGAACCACTCTAAAAGTAAAATATATGTGAATCTAGAAACTTCATCGGCGATGGCGATGGCGATGGCGATGATTCAAAGGGCTTCAAGTGCCCATCAATGGCCAAACATCACCGTTGCACAACCACATCAATTTTTAGATAATATTTCAAAAAACCTAATGAAAGCATAAAGAAATAGTTTCCAAAAGTCCAATTTTTACGCAAGTTAATTGTCAAGTGAAGATTGATATTTAACTGAAATTCTAACAGGCCTTAGGATGGGCTCATGAAAATTATAAAGAGAAATATTTTTTTTGGTATATTATGAAGGGAAGCCCTATGATACACATTAAACCACTTTCATAAATATTTTTATAACTAGCTGTAATAATTCATAACATTTTCATGCATATCTCGTAACTAACCAAAATAACACGTAATGAACATTACAAAAAGTCTAAAAAAAAAAAAAAAAAAAACCACAAAAAATAACGCAGCAAACCATACAACCGAAATGCTTACCAAATGAACCATAAATTTTGTAACTAATCACAAAAATGCACAACTCTACTTTTCTATAGTCGACACATACGCCTAACTCGTTACAATAAATCATAACCAGCACTCACAAAGCATGTGTACCGTGTAGCTTTTTGTCCTTCAAAGTATGTGTGATTCTTATTCTGATAGTGTTAAATGGAAAAGAGAAATGTAAAAGTGGGAAATACATGTGAGTTGATGGTGTTATTACGCTATGTATTGTGAATTGACAGGTAATAGCAACAAGGAATACTCGTGAACGCAGTAACAGGCTCGAGAATATGTGCTGGCGAATTTGGCATCTCGCACGTAAAAAGAAACAGGTCTCGCTCTCGCTCGCTCCAAATACATATAAAGTTGGCTCTCTCTTTTTTAAGCCTAAAATATTCGAGAATCAATTTCACAACAATAACGGCTAAATAAAAACAATAATCAATCGATTTCAAAAATTAGTTTTCATTAAATATTCTGCTCCTGTACTTGCATCGCGTGAGCACCGATCGCTAGTATCCATACGTGCATGTGTGTCCATATGTTATGGAAGTGGATTGTCGATACAATGGCATGAAAGCATAGAAACACATGTATGGAATCAATTTCTGATGATAACAAAAGCAAAAAAAACAAAGCACGAAAAATGGAGGAATTTGGGGTCTACCTCTGTGTTCCAAATCTTTTATTTTACAAGACTAGTAAATCAGATCATCCAAACAAAAAATAAGATTGATGAGACAATAATAGCCACGTTGTTGGATTACGTTTGTATAGCAAAAAATAAACAATCTATTTCTAAAATAGTTACTAAACAATAAACTTGATTCATTTATGTACTTGATGACGTCCAATCCAAATTAGTGGATGCTACCAAAAAGAGTGGCAAGGCCATAGCCGAAAAAGAAGACACCCACAATGTGGGAACTCAGGATTAACCTCCTAATTACGACAATATGTTTGTGGGTAAATGTATAACGAACAGAAAATATATACAAGAATATGGTATACATAATCATGCGTTTCTCTAAAACAAATAAATCATCAAGATGCCTTCTAATTACAAGAATACCGCTCCACGTTATATTGGTGGAAGATTCCGTATCCAAATTCTGGAAGAATATCATTTGCCTGTGTTTAATTTGACAGCGCAGAGCGGGCATATATGGGCATTGTAGCTGTTCTTTTTCCACATATATGTTTAATTTATCCTCCAATAGCCCAAAGGCCTTTATGCTCTTTAATTTTTCTGAGATTCCTAAAACTTTCACATGGACTGAACCAAAACACTACCAGAATAGCTATTACAATGAGAGAAACAAAACTGTAAAAATGCTAGAAATACCAGCAATAGGTTTTTTCCACCCCCTTTTGTGGTAACCAACTTTGAGACATTCATATGATCCTTTAGATGAACGCACCGACGAACACAAGATTTTAACTTTAGGGATAAAATATAACCTTTGGAACCTATACAATGAAATACATGGTGATTCAATCTTAGGTGGAACCTAAACGTAGTTTTTGTATATTATGAAGTTATAAAAATACTATAAACAGTCCCTAGCCACCAGGGGTGCCCCCTCAAGAACTCATTCGTCGATATGTAGTTAACTCGGCACATAAAACTAAACTAAAACTGCAAAAAGTAAGAGTAAAGCCTACCAACTTGATGTGATTCTGTTTATTATGTAAGTTTTTCTTCATAATTTAATGCGTCGAGACCTGAAAATCTGGGAGAAAAGATTTATCTGCAGATACCTTGGGATGAGCACAAAAGGCTGCAAAAAGACGAATCGAGCGTGAGAAAGGTCGGAATGATGCTGCAGAAGACCTGTCTGAGCTTTCCGAAGGGGAGAAGGAAAAGGGTGATGCCAATCAGACAGAGCCTATATCAGAAAAGATTTCCAGAATTAACTCTGACATGAAAATATGGTCGGATGATGATAAATCTAGGCGCCTCTACATTGTCCTAATCAGGTAATTCATAAAAACTACCATGTATTATTTGTGTTCCAAACAAGGTAATCGTGTCATGCACTGTACAACTAGTTATACGATACCAAGACAATAAACTAAGGTCTCCATTAATTCCTATACTATACCAAACCAAACCTTGGTACATAAATTCCAATAGGAAAATGAGGCATTCATATTAACAAATGTTAGTATTGTCGGGGCATTCCTCACAGTGATAGATGCATACAAGTCATTATATACAAAAGGTTAGTGTCTATAATTTTCATCAGTTGTCAAGTACTTTCTGACGCTATTTTTGAACCTTGATGAGTTAAGGCAAAGGAATATGAAAGGGAAATGAAATTGTGCCCGGGAAATCAGACAGAGAAAAGGATTCTTTTGACCTCTACCTATTGATTTTCTTCTCATGTTCTTAATCTGACGAAATAAAAGAAAAGAATCTCGCATCTTTCTATCCATTTTGTTTTCCGATCTTTTCCTACCCCTTTCCCTTTCCTCCACAATCCAAGATCCAAACATGCCATCAATGTCAACAAGATGTTTTAGGAAAGATACTGAAAAGTGTACTGTTTATGGAGGGCAAAGAATAGGAAAGGGAAATGAAATTATGCAGGGTACCAGTTTGATAACATGTTTATTGTTTATGTAGTCTACATGGATTGGTGCGTGGGGAAAATATGGAACTCGGAAGAGATTCAGACACTGGTGGTCAGGTAGTAACATAATCCTGGGAAGGAAACCTGTATCTAATGATTAATGAGCTCTTAACTGATGCCATCACTAGCCGAAACTGAATGGATAATTGGTTTATATGTTCAGGTGAAGTATGTAGTAGAGCTTGCCCGAGCCCTGGCCAACATGAAAGGAATCTATCGTGTTGACCTTCTGACTCGACAAATCACCTCGTCGGAGATTGACTTTAGCTACGGTGAGCCCAATGAGATGCTCTCGTGCCCATCCGATGGCAGTGGTAGCTGTGGTGCATACATTATTCGGATCCCATGTGGACCTCGCGACAAGTAATTACATGCATTCATTTCTTCTCTAGCTATGTTTACTTATATCACCATCAAGAATTTTATATTTGTAATTCATAACCTTCACTGTAAGTTTTTCACACTCCTAGAGGTATAATATACCAAAACATGTGTCTAACTTCGAATGCCATGGCTACAGGTACATTCCAAAAGAGTCACTCTGGCCTCACATACCAGAATTTGTAGATGGGGCCCTAAGCCACATCGTGAACATGGCAAGAGCTATAGGAGAACAAGTGGATGGGGGAAAGCCGACGTGGCCTTACGTAATTCACGGTCACTATGCTGACGCAGGGGAGGTAGCAGCACGCCTATCCGGGGCATTGAATGTGCCAATGGTGCTAACGGGGCACTCCTTGGGAAGAAACAAATTTGAGCAATTACTTAAACAAGGGAGGTTGTCTAGGGAGGATATAAATTCAGCGTACAAGATAATGAGGAGGATTGAGGCTGAAGAGTTGGGGTTGGATGCTGCTGAAATGGTGGTAACTAGCACGAGGCAAGAGATCGAAGAGCAATGGGGTTTATACGATGGGTTTGATATCAAGTTGGAGAGGAAGCTTAGGGTTAGAAAACGGCGTGGAGTGAGTTGCCTTGGTCGATACATGCCAAGGATGGTGGTATGTACAATCTCTTTTTGCTAGTCATTCTAATAAACCTAGGCTTAAGTTCCTTTTCTGATATTTACATGAAACTAGGTTACACCACCGGGGATGGACTTCAGCTATGTCACAACACAAGATTCATTGGAAGGTGACGGAGATCTAAAGTCGTTGATTGGCTCTGATAGAACTCAAAACAAAAGGCACATACCTCCAATATGGTCCGAGGTCACGTATCATTTTCTTCCATACTTGTATAATGTCAGCCCTTTGGAATAATACGACCAATCAGATCACAAGCCTAATGACATTTGTCATTTCTATGGACATCATGTCTACATTAAGTTAACCATTAACCCTTTCACTATATTTAATGCAGGTAATGCGATTTTTCACAAACCCTCACAAGCCTATGATCCTAGCATTGTCACGTCCAGACCCCAAGAAAAATGTGACCACTTTGCTCAAGGCTTTTGGAGAATGCCGACCACTCAAAGAATTAGCCAACTTGGTAAAACTATATTCACCGCAATAATCTCGTACTTTCTACAAGTCATAGTTCCACACACAATTAGTGAATCACGCCAACTTATTATTGCAGACCCTAATACTTGGTAACAGAGACGACATAGAAGATATGTCTAACAGCAGTTCAGTAGTTCTCACAACAGTGCTTAAACTCATTGACAAGTACGACTTGTACGGTCAGGTAGCATATCCCAAACATCACAAACAATCCGAAGTTCCTGAGATCTATCGACTGGCTGCAAAAACAAAGGTAAAATGCATGTCCTAGTTTACCTCCAAGCAAATTGTGAAGTCTTAATCATGAGATGGTACTACGACTCTGAAGTAATCACCAAGCTAGGCTCTGATGAATAGTGGACCATGATATAGTAATCGCCTGTAGAGCCTATATAGATTCCAGATGCAATATCTATGTGAATGTCGGGCCATAGGTCATCAGCCTATATCATTATTTAAGAAGTTACTAATTACTTAACGTTCCGTTCGTTGCAACATTTACACTGCTAACAGTAACACTTCTATGGCGGATTTGTCTCTTGAAGGGAGTTTTCATCAACCCAGCCCTGGTTGAACCATTTGGTCTCACACTCATTGAGGTTAGTTACCACTATCTGACTCGGCTATTGAACGGATTTAATGCGGTTGAAATTCCTGGAAAAAGAACTATTACAAAATCTTAAAGTTCATATATCTCTATATTTTGCAGGCGGCTGCTTATGGTTTACCAATTGTTGCCACGAAAAATGGTGGCCCTGTGGATATTCTCAAGGTGAAACACAGAGATAATTTTAAGTTTCTTTAGTCAGTCATTTGAATCATATAAAAACAAAGTCTAACTTATCAGGAACCTAATCAATTTGAGATTTTAATGGGTCAATTAACCCCATCTTGGAACACATAGATAATTTTAAGTTTCTTCAGTCAGTCGTTTGAGTCGTACAAAAACAAAGTCTAACTTATCAGGTGACTCATTGCCCACAACCTAATCAATTTGAGATTTTAATGGGTCAATTAACCCCATCTGTTGACGTTCAACACCATGATTACCCAAAACTATACGCTAATTTGCAGAAAGTGACACGCAAGAAACTACTCAAACGAATCTAATTGGGAGTTCCTTGTCAATCAGGCACTCAACAATGGCCTTCTCATTGATCCACATGACCAGAAAGCCATAGCAGATGCCCTCCTAAAGCTTGTAGCTGAAAAGCATCTTTGGCTTGAGTGCCGCAAAAATGGCCTAAAGTATATTCATCGTTTCTCGTGGCCAGAACATTGTCGTAACTATCTTTCCCATGTCGAGCATTGCAGGAACCGCCATCCTACAACCCGTCTTGAGATCATGCCAACTCCTGAAGAACCCATGAGCGAATCTTTAAGGGATGTGGAAGACATTTCTTTAAAATTCTCCATTGATGCAGACTTCAAGCTCAATGGAGACCTTGATGTACCAAATAGACAATGCAAACTCATTGAGGCCTTAACACAGATGGGTTCCTCCAATAGCCCTTCCAGCACTAGTTACTGTCCAGGAAGAAGGCAGGCACTATTTATAATTGCTACTGATTGTTATAACAGGGGTGGAATGTGCACCGAGACCTTTCCATTGGTCATCAAGAATGTGATGCAAGCTGCAGTCTCAAACTCAGGCAAGATAGGCTTTATATTGTCAACAGGTTTAACTTTACTTGAGACAAAGGAAATGTTAAGACATTGCCATGTAAATTTGGAAGATTTCGACGCATTTGTATGCAATAGTGGAAGTGAAATGTACTATCCATGGAGAGACTCCACAGCTGATATGGATTATGAAGCCCATATTGAATACAGGTGGCCCGGCGAGAATGTGAGATCAATGATAATGAGGCTTGGTCGGGTAGGAGATGGAGACGGAGATGGAGATGATATTATGGAAAGTACAGATGCATTTAGCTCCCGATGCTATTCTTATAGCATTAAACAAGGAAGCAAGGTGAGAATTTTTATAAACCAGTGTGTTGTATAATTAAGAAAAAGAAGTTAGGGAGAGATCATAAGGCATACTATCTAAGGCATTCATTATGTTGGTATAATCTGTCTACTTATCACCCAAATGTGTGGTTGGCAGACTCGAAGGATCGATGAACTGCGCCAGAGGCTACGCATGAGAGGTTTACGGTGCAATGTTATCTACACTCGTACTTCATCACGGTTGAAGGTAGTACCTTTATTTGCATCAAGAGCTCAAGCACTAAGGTTAGAACTTCTGTTCTTAATTACTGAATTCTATGGATGGTGTGTATGCGCACTCGTGGTTTATGTGCAGTCAACATGAGAGAGAGAGAGAGAGAGATCAGCCAACCACTCATGTTCCTGCATGTCTTTCAGGTATCTATCTGTAAGGTGGGCTATTGATCTTTCCAAAATGGTAGTGTTTGTCGGAGAAAGAGGGGATACAGACTATGAAGACCTGTTGGTTGGCCTACACAAGACTGTTATTCTAAGAAATTCTGTAGAATATGGCAGTGAGATGCTTCTGCGCAGTGAAGAAAGTTTTAAACGCGAAGATGTGGTCCCCCAAGATAGCCCTAGGATTGCCTTTGGTGCATGTTATGAAACCCATGATATCTCTGCAGCTTTAGATGCTCTACAGGTCATATGATTTTTACTTACCCTATTATTACCTCTTTTGGGAAGAGGGATTCATGTTATACCTTTATATGTAATGCAGAAGCAAGTATATAATTATATATATGCGAGAAATACAAATCTACTTTGG

>AeSUS1

AAAGAGAATACAGGGGATGAACAAGAAAAGCGGTTTTGTGAATACAAGGTGTGTGTGAGAGAGAGAGAGAGAGAAATTGGTGTTCCACCAAAACATAAGTAGCAGACGGGCATATAAAATGGGATCCAAGGCTCCCCTTTCCATCATCCCAAAGCATAACAATATCGTTCACTGCTTTCTGTTCCCCCTCTGTTTCCGTCCATTTATTTTCTTTTGATTTCATTCACTCATTCATTCCTCTCTGTTATTTCATTGGTCTGCTTTTTGGAAAGGAAAAAAAAAGGTAATTAATTTCGCAGTTGCATGATCTCTTTTGGTAGTTGCTGTTTTTTTTTAGAGCGAGTCCATATGATCTAAGTCGGTGGCTAAGATTTCTGGATATCAAACTAGAATCTTAGCCCAGATCTCCAATGACCACGTCACATGTTAATTACCCAAGATTTTAGATTTTGTAAGATTCGATGTTTTGAGTATCTTTCGGGGAAAGAAATTTCTTTTTCGGAAGCTTTTTGCACATACTGATTTGCCGATGTTCTAACATCTCTTCCGAATAATTTTCTTCTAAAAAAGGGTAAATTTCGCCTCCCAAATTCTCCAATTTTTGGCGAGTAAAAAACACGAACAGGAAGTTTCTTGCTCAAAAAGTTTGAGATCTTTACAATGTGTAAAAACATTCCATTTTGAGACGGAAGTTGTTAAGGTTGCCAAATTTAAGGCAGAGAATAAATCGGATCTTTTCACTATAAATTCCATCGCATGCACGCACATGGAATCACTGCACTCTCTGCTTATTCTTCTTCACTATCAACATTCTTTCGTGAGTAGTATACGCCTAGCCTAGTTTTTTGGACATATTTTTCGTTTGGATGATCTTTTTTTTTTTTCTCTTCTCTTCTTACTCTGTTTTTCTATTATATTCCATTCTCAGTGTTGTTTTCTTAGAAAAAAGGATTCTTTAACATGATCTGTTTCACGAAGTATAATTAGAGTTTTTTTTAATGGGTCGCAGTAGTAGTTATTTGAGCATACCGTATACGGGATGTGTTCTTCATATAAAAAAAAAAAGACAAAAAAACAAACAAACTTGGGTTTGAAGTTGTTGGATGTTGCAAAACAATCTGGGATTTTTTAGGACATAAGAGAGAGAGTATTGATGAACCCAGTTGTCAAAAGCGTTGGTTGTCAAGCATTCATCGTCCTTGCCTACCATTGGGGACCTAATAAATGTCAATAAGTGGGTGCTGTTGCGTGTGTTGGTCGGTTTTGAATTTTTTTAGACGAATCGCATTCTAGATCATCCCAAATTAGGTGGATCATTGACCTTTTGTGGTCAGTTCCGTCTTTTTTAAGCTGTAGATTTTGGATAATGCAGTATATCCATATTTATTCTTCAAACTTGTATAAACTAGTATTTGTTTTGTTTGTAATAGCAGGTGTTGTTTGTTGTGTGTGAACACCGGAAGACATGGCAGGACAAGTCCTGACTCGTGTTCACAGCCTTCGCGAGCGTCTTGATGGAACTCTGTCTGCTCATCGCAATGAAATATTGCTGTTTCTCTCCAAGTATGTAGTATTAATTGCATAATCGGATCACTTCAGTGAAAGAACACTCTCATTTGAGTTGTCTGACACATGGGTTTGGATTTTTTATGATATTATTGTAGGATTGAAAGCCATGGCAAAGGAATTCTGAAACCCCATCAGATTGAGGCTGAGATCGAAGCACTCTCCAAAGAGGTCCAACAGAAACTGTATGATGGAGCATTTGGAGAGCTTCTCAAATCAGCACAGGTAAATCATCAAAAAAGATTGTGCAATTTAATTTCTCTTTCTGGATGATCAAATCTTGTGATCTGTTAATTTTGGGTATGACAGGAAGCAATTGTTTTGCCTCCATGGATTGCTTTTGCGGTTCGGCTAAGGCCCGGTGTGTGGGAATACATGAGGGTGAATCTCAATGCCCTTGTTGTTGAAGAATTGAGTGTTCCCGAGTATCTGCAGTTCAAGGAAGAACTTGTGGATGGACCGTAAGTACTAACTCTGCAACTGTTCGTGTTACTTGGAAGCTGTTAGCATATGTATTGATGATGTTGGTTGCTTCTTGTTGTGGGTGCAGGTGCAATGGAAACTTTATTCTTGAGTTGGATTTTGAGCCCTTCACTGCATCATTTCCTCGGCCAACTCTTTCAAAATCAATTGGGAATGGAGTTGAGTTCCTTAACAGACACCTCTCTGCTAAAATGTTCCATGACAAGGAAAGCATGCACCCTCTCCTTGATTTTCTTAAAGTCCACAACTACAATGGCAAGGTAATTAACCTTTATTAATTACTACATTTGATTTAGATTTTGGTTTAGGATTGTGAAATAATTTTTTATGTTCTTGGTGCAGACAATGATGCTGAATGACAGAATACAAAACCTCAATGCTCTCCAATTTGTGCTGAGGAAGGCTGAGGAATATCTCCTTACACTCCCTCTAGAGACACCCTATTCCGAGTTTGAGCACAAGTTCCAAGAGATCGGTTTGGAGAGAGGGTGGGGTGACACTGCTGAGAGAGTGCTTGAAATGATTCATATGCTGTTGGAGCTTCTTGAGGCCCCAGATCCATGCACTCTTGAGAAATTTCTTGGCAGAATCCCAATGGTTTTCAATGTTGTGATCCTTTCTCCCCATGGCTACTTTGCCCAAGAAAATGTTTTGGGCTATCCTGACACTGGTGGTCAGGTGTGTGTTTCTTAAACACACACACGCACATAAAAAGAAATCGTTCCAAGTCTTTTGATTTCCCAAATATAGTTCAACTGCCTGAAAATGCTAACTTGTGTCCTTAATTTTGTTTGTGGTTTAATTAGGTGGTTTACATTTTGGATCAAGTTCCTGCCATGGAGAAGGAAATGCTTAAGCGCATCAAGCAGCAAGGGCTTGATATATCCTCGCATTCCATTGTCGTGTACAACATGTGTCAATTCAAAGCCTTTTTCTTCTTTTTTCTTTTTGTTTCCTTCTTTAATGCTTGGTCTGTCTTGCTGTTGTAATCAGGTAACTAGGCTTCTCCCAGATGCGGTAGGCACTACCTGCAATCAGCGCATTGAGAAGGTTTACGGAGCAGAACATTCGCATATACTTCGAGTTCCCTTTAGGACTGAGGAGGGAATTGTCCGCAAATGGATCTCACGTTTTGAAGTCTGGCCTTACATGGAGAGATTCACCGAGGTCAAATTACTTCATTTAGTCATTGGAATTCTTATTAAAATTGTGTCTGACCTTATCTTTTAATTTGCATAGGATGTTGCACATGATATCGTCACAGAGTTGCAGGCAAAGCCTGACTTGATCATTGGTAACTATAGCGAGGGTAACCTTGTTGCCTCATTGTTAGCTCACAAATTGGGGGTAACACAGGTAATCATGCTTATTGCACGCTTTGGAGCTGATCTGCATTTCTTAGCTTCATTCGTGTCAATCGACCTGACTAATCATTTTTTTCAATTGTTACTTCCCTAGTGTACCATTGCTCATGCCTTGGAGAAAACAAAATATCCAGATTCCGACATCTATTTGAAGAAATTTGACGAGAAGTACCACTTCTCGTGCCAGTTCACAGCAGATCTCATTGCTATGAATCATACCGATTTTATAATCACCAGCACTTTCCAGGAAATTGCTGGAAGGTATTACTTTGTTACATGGTTTTCATTTTTAGCTTGCTGTGGTGTTGTTTATAATGCTAATATGCTACACACATTGTGTTCTCCGCAAGAACACTGTTGGACAGTATGAGAGTCATATGGCCTTCACTATGCCCGGACTTTACCGAGTTGTCCATGGTATTGATGTGTTTGACCCCAAATTCAACATTGTCTCACCGGGGGCCGATATGAACATCTACTTCCCTCACACTGAGAAGGACAAGAGACTGACCAAGTTCCACCCTGAAATCGAAGATCTTCTCTTTAGTGATGTGGAGAATAAAGAGCATATGTAAGTTTCTATTTCTTCTATTAGTTTAGTACCTAGGTTGATATGCTTGGCAAAATGCAAATTGATATTTTATTACTTTTGGTGTTTCTAACAGAGGTGTGTTGAAAGACCGTACCAAGCCAATCATATTCTCCATGGCAAGGTTGGACCGTGTGAAGAACTTGACCGGGCTTGTCGAGTTGTACGGCAAGAATGCCAGACTTAGAGAGCTGGCCAACCTTGTGGTGGTGGGTGGAGATCGTAGGAAGGAGTCCAAGGATTTGGAAGAGCAAGCCGAGATGAAGAAGATGTACGATCTCATTGAAACGTACAAGTTGAACGGTCAGTTTAGATGGATTTCTTCCCAGATGAACCGGGTGAGGAATGGGGAACTTTACCGCTTCATTGCTGACACCAAGGGTGTGTTTGTTCAACCCGCCTTCTATGAGGCTTTTGGGTTGACAGTGGTGGAGGCGATGACCTGTGGTTTGCCAACATTTGCAACTTGCCATGGTGGTCCAGCTGAGATAATTATTCACGGCAAGTCTGGCTTCCACATTGATCCTTATCATGGTGATCAGGTCGCTGAACTCCTTGTCAATTTCTACGAGAAGTGTAAAGTTGATCCTTCTCATTGGGACGCTATTTCCGAAGGAGGTCTGAAGCGCATCCTGGAGAAGTAAACACCCAAACCTTTCTCTTGTAGATGATGTCCATTGCTATTTTAAATTGTAAAGTTGTACAGAGAACTAAAATTACTTGTGCCTTTGCTTGCTGGTTTGCAGGTACACATGGCAGATTTACTCTGAGAGGCTAATGACTCTTGCTGGGGTTTACGGATTTTGGAAGTACGTCTCCAAGCTTGATCGCCGTGAGACTCGCCGCTACCTTGAGATGTTTTATGCTCTCAAGTACCGCAAGTTGGTAAGTGTTGCATTCACTTATTGGTAATCGTATAATTGGTATCTTAGGTGATGATTTTGTGTGATGGTGTGTTTCAGGCTGAGGCAGTTCCTTTGGCTGTTGATCAGTAGAGGCATGATTGGAAAGTAATGCATCAAAGGCTTGTCAAGTGGAAGTTTTCCGGAGCTGTTGAATAAACTTTCAAGTATTTGATGTGTTGATGACAAGTCTTCTGTTTTTGTTTTTTCACGTCTGTTTACGTTTTTCCCTCTCCTTTTCCATTTCATTCTGTTTTGTCGTTCCCATGATGATGGGGCATTGCTTGGAATTGGTAGTTGTTTTTTGCATTGCTGTTTGCCCATCCCTTTCTAAGATTAAGATTGGGTATTTGAGGTTCTGAATTGTCAGTATTAATAAAGATCAAATTACCAATCTGTTCTTATATTTTGTTCTCTTTATGTTTGTGTTGGAAGTTGATGGATGTGCCCACCAAACTCAATTTGATCCACTCCACTAACAATATATATCGTACGATACCGA

>AeSUS2

ATACAGTGGGTGAAAAGAGAATACAGGTGATGATTCTGATGAACAAGAAAGGTGAGAGAGAGAGAGAGAGAGAGAGAGAGATTGGTGTTCCACCAAAACATAAGTACCAGAGGGGCCTGCTAGGGGCATATAAAATGGTAACCAAGGCTCACCCTTTCCACCATCCGAAAGCATAACAATCTCGTTCACTGCTTTCTCTCTCTCTCTCTCTCTCCCCCTCTGTTTCCGTCCATTCAATTTCTCTCTACCTTTTTGATTTCCTTCACTCATTCATTGATCTCTGTTATTTCATTGGTCTGCTTTTTGAAAAGGAAACAAAAGGTAATTAATTTGGCAGTTGCATTGGTAGTTGCCTTTTTTTAGAGCTAGTCCATATTATCTAAAAGTCGGTGGCTACGATTTCTGGATATGAAACTAAAAACTTAACCCCGATTTCCAACGACCACGTCACATGTTTATTACCCACGATTTTAGATTTTGTAAGATTCGATGTTTTTAGTATCTTTCGGGGGAAAGAAATTTCCTTTTTCGGAAGCTTTTTGCACATACTGATTTGTCAGTATTCTAACCTCTGTTCCGAAGAATTTTCTCCTCAGAAAAAATATTGGGAAGATTCTGGTAATCGCTTCCCAGATGCTCCATTTCTTGGAGAGTAAATTACACGAACATGAAGTTTCTTGCTCAAAAAGTTTGAAATAATAATTATTGTGTAATTTTTCCCTCAAAACATTCCTATTTGAGACGGAAGCTGTTAAAGGTTGCCAAATTTAGGGCAGAGAATAAATCGGACTTTTTCAGTATAAATTCCGTCGCATGCACGCACACTGAATCACTGCACGTTCTTCTTCTGAGTTATCCAGTATCTATATTCTCTCGTAAGTCGTGTAAGTCTAGTCTAGTTGTTTTGGCATGTTTTTCTTTTGACGAATCCTATTTTATTTTTATTTTTCTCTTACTCTGTTTTCCAATTCTTTTTCTTAGAAAAAAAAAGGGGTTTCTTTGACATGATGCTCTGTTTCACGCAGATCATTGTTTGTGGTGGGTTTCTATAGATTGCAGATGTAGTGATTAGAGTATTCGGAATGGGATCTTCACATAATAAAAACACTTGGGGTTTGAAGTTGTTGGATGTTGCAAAAAAATCTAAGATTTTTGAGGACATAAGAGAGAGAGAGAGAGTATTGATGAACCCAGTTGTCAAAAGCGTTGGTTGTCGAGCGTTCATCGTCCTTGCCTACCATTGGGGACCTAATAAAAGTCAATAAGTGGGTGCCGTTGAGTGTATTGGTTGGTTTTTAAATTTTTAGACGAATCGCATTCTAGATCATCCCAAATTAGGTGGATCATTGACCTTTTGTGGTCAGTCCCGTCTCTTTTAAGTAATAGATTTTGGATAATGCAGTATATCCATATTTATTCTCAAATTTATTATATGTATAAATAGTATTGTTGTTTTGTTTGTTAATGCAGGTGTGTTGTTGCGTGTGAATCCTTGAAGACATGGCAGGACAAGTCATGACTCGTGTTCACAGCCTTCGCGAGCGTCTTGATGGAACTCTGTCTGCTCAGCGCAATGAAATATTGTTGATTCTCTCCAAGTATGAAGTATTAATTGCATATCAGATCACTTAGTGCAAGACCACGCCCCATTTTGAGTGGTCTGAAATATTTTGTTTTTTTTTTTTTTTATGATATTATTGTAGGATGAAAGGCCATGGCAAGGAATTTCTGAAAAACCCCACCAGATCGAGCTGAGGGAGTGAAGCACTCTCCAAAGAGAGGTCCAACAGACACTGTAGGACGGAGCATTTGGAGAGCTTCTCAACTCTGCACAGGTAAATAATCAAAAAATCATTGTGCAATTTAATTTCTCTTCTGGATGATTAAATCTCGTGATCTGTTAATTTTGGGTATGACAGGAAGCAATGGTTTTGCCTCCATGGGTTGCTTTTGCGATTCGGCTGAGGCCTGGTGTGTGGGAATACATGAGGGTTAATCTCAATGCCCATGTTGTTGAGGAATTGAGTGTTCCCGAGTATCTACAGTTCAAAGAAGAACTTGTCGATGGACCGTAAGTGACTCTGCAACTGTTAGTTGTTACTGGGCAGCTGTTTAGCATCTTATGGATTCATGGTGGTTGCTTGTTGTGGGGCAGGTTGCAATGGAAAGCTTGTTGCTTGAGTTGGATTTGAGCCCTTCACCGCATCATTTCCTCGCCCAAACTCTTTCAAAAGTCAATTGGGAATGGAGTTGCAGTCCTTAACAGACACCTCTCTGCCAAAGGTTCACGACAAGAAAGATGTACCCTATACTTGATTTCTTAAAGTCCACAACTACAATGGAAAGGTAATTAACCTTTGTGGATTACTCCATTTGATTTAGATTTTGTTTAGGATTGTGGTATAATCTTTTATTTTCTTTGTGCAGACAATGATGCTGAATGACAGAATACAAAACCTCGATGCCCTACCAATTTGTGCTGAGGGAAGCGGAAGGCAGAGGAATTATCTCCTACACTCCCTCTAGAGACACCGTATTCCGAGTTTGAGCACAAGTTCCAAGAGATCGGTTTGGAGAGAGGGTGGGGTGACACTGCTGAGAGAGTGCTTGAAATTGCTTCATATGCTATTGGAGCTTCTTGAGGCTCCAGATCCATGCACTCTTGAGAAATTCCTTGGCAGAATCCCAATGGTTTTCAATGTTGTGATCCTTTCTCCCCATGGCTACTTTGCCCAAGAAAATGTTTTGGGCTATCCTGACACTGGTGGCCAGGTGTGTGTTGCTTAAACACATACACGCACACAAAAAGAAATCTTTCCAAGTCTTTTGATTTCCCAAATAGTTCAATTGCCTGAAAATGATAACTTGTGTCCTTAATTTTGTTTGTGGCTACTTAGGTGGTTTAACATTTGGATCAAGTTCTGCCCTGGAGAGGAAATGCTTAAGCGCATCAAGCAGCAAGGCCTTGATATCATTCCACGCATTCTCATTGTTAGTGTTACTATATGTTCAATTCAAAGCCTTTTCTGCTTAATTTTTGTTTTTTTGTCTGTTTAATTTCCCTCTTTAATGCTTGGTCTGTCTTGCTGTTGTAATCAGGTACTAGGGCGTTCTTCTCCCCAGATGCAGTAGGCACTACCTGCATCAGCGCATTGATAAGTTTATGGAGCAGAACATTCGCATATACTTTTCGAGTTACTTTAGACTGGAAGGGAATTGTCCGCCAATGGATCTCGCGTTTTGAAGTCTGGCCTTACATGGAGAGATTCACCGAGGTCATATTACCTTCTTTATTTAGTTATTTGAATTATTATTAAAGTTTTTTCTGACCTTATCTTTTTTTTTTTTTTTTTTTTAATTTGCATAGGATGTTGCACATGACATCGTCACAGAGTTGCAGGCAAAGCCTGATTTGGTCATTGGCAACTACAGTGAGGGCAACCTTGTTGCATCATTGTTAGCTCACAAATTAGGGGTAACTCAGGTAATCATGCTTATTTCACTTTGGAGGTGATCTAGATTTCTTAGCTTCATCCATCAATCGACCTACTGACTAATCATGTTTGAAATTGCTACTTCCATAGTGTACCATTGCTCACGCCTTGGAGAAAACAAAATATCCTGATTCCGACATCTATTTGAAGAAATTTGACGACAAGTACCACTTTTCGTGCCAGTTCACAGCAGATCTCATTGCTATGAATCACACCGATTTTATAATCACAAGCACTTTCCAGGAAATTGCTGGAAGGTATTACTTGGTTACATGGTTTTCGATTTTTAACTTACTCTGTTATTGTTTATAATGCTAATATTGCTACACACATGTTGTTTCTCCAGCAAGAATACCGTTGGACAGTATGAGAGTCATATGGCCTTCACTATGCCTGGACTCTATCGAGTTGTCCATGGTATTGATGTGTTTGACCCCAAATTCAACATTGTCTCACCAGGGGCCGATATGAACATCTACTTCCCTCACACTGAGAAGGACAAGAGACTGACCAAGTTCCACCCTGAAATCGAAGATCTTCTATTTAGTGATGTGGAGAATAAAGAGCATATGTAAGTTTCAATTTCTTCAATTAGTTTGGTACCTAGTTGTCTTTCCACTCTTATATGCTTTGCCAAAATGCTCATTGATATTGTATTACTTTTGGTGTTCTAACAGTGGGGTGTTGAAAGACCGTACCAAGCCAATCATATTCTCCATGGCAAGGTTGGACCGTGTGAAGAACTTGACCGGACTTGTCGAGTTGTACGGTAAGAATGCCAGACTTAGAGAGCTGGCCAACCTTGTGGTGGTGGGTGGAGATCGTAGGAAGGAGTCCAAGGATTTGGAAGAGCAAGCCGAGATGAAGAAGATGTACGATCTCATTGAAACCTACAAGTTGAATGGTCAGTTTAGATGGATATCTTCTCAGATGAACCGGGTGAGGAATGGGGAACTTTACCGCTGCATTGCTGACACAAAGGGTGTATTTGTTCAACCTGCCTTCTATGAGGCTTTTGGGTTGACGGTGGTGGAGTCGATGACCTGTGGTTTGCCAACATTTGCGACTTGCCACGGTGGTCCGGCTGAGATAATTATTCATGGCAAGTCTGGCTTCCACATTGATCCTTATCATGGTGATCAAGTGGCTGAACTCCTTGTCAATTTTTACGAGAAGTGCAAAATTGATCCTTCTCATTGGGACGCTATTTCTGAAGGAGGTCTGAAGCGCATCCTGGAGAAGTAAACACCCAAACCTTTCATTTCTGTCGATGTCCATTGCTATTTAAGTTGTAAAGTTAATACAGAAAACTAAAATTTCTTGTGCCTTGGTTTGCTTGGTTGCAGATACACATGGCAGATTTACTCTGAGAGGCTAATGACTCTTGCTGGGGTTTACGGCTTTTGGAAGTACGTGTCTAAGCTTGATCGCCGAGAGACACGCCGTTACCTGGAGATGTTATGCACTCAAGTACCGCAAGTTGGTAAGTGTTGCATTCACTTGTTGAGTGAACCCTAACCGTATAGTAATTGGTATCTTAGGCGATGATTTTGTGTGATGGTGTGTTTCAGGCTGAGGCAGTTCCTTTGGCTGTTGATCAGTAGAGGGATGATTGGCAAGTAATGCATCCAGGCTTGTCAAGTGGGGAGTTTACCAGAGCTGTTGAATAAACTTTCAAGCATTTGATGTGTTGATGACAAGAGTCTTCTGTTTTTGATTTTTTATGTTTGTTTGCGTTTTCCCTCGCCTTTTCCATTTTCTACGGTTTTGTCGTTCCCATTATGATGGGGCATTGCTTGGAATTGGCAGTTGGTTTTTGCATTTTCTGTTTCCAATGAAGATTGTGTTGCGGAAGCACCCAAACATTGAGATTCAAACACAACCACAATCACAAGATTTACGTGGTTCGGCTATAACATGCTTAACTCCACAGAAAGGATCCCGTTCTTTGTCCTATGAGAATAATTTGTACAAGTACACTTGAGTATGAACGACCCACACGACCCACACCCAACCCTTTATACGCTCGCTTATACAAGAACTCACTCTCACCCAAGCCTAAGCTTTACACTTGATCACACATTGTCACCCACAATTGCTGTATATGACTCCTGTGTTCTTCTTAATATACATGATCATGGATAAA

>AeSUS3

AACAACTTTCTTTTTTTAATCTCTCTGTATCTTGAAACTATCCCTGATCGAAATGGCAGCCTTGAAGAGGTCTGAGTCGATGGCTGATAGCATGCCGGACGCCCTGAGAGAGAGCCGGTACCACATGAAGAAGTGCTTTGCTAAGTACATTGAGCAAGGAAAGAGATTGATGAAACTTCGACACTTAATGAGCGAAATGGAGAAAGTGATCGATGATAAGACTGAGAGAGAGCAGTTCTTGAACAGCCTACTCGGCTACATTTTGTGCACCACTCAGGTACATTTTCATCTAGAGTAATGCTATACAAATGCGAACATCATTCACAAGATGTATACAGAATTCTGCAACTGACTGCTGTCTAGCCACATCATTTTGGTCGAAGATGATTCTGCATACGCCAGATATATACAAAATTCTATAGCCGACTGCTGTCTAGTCACATCAATCTGGTCGATGATTCTGTATAAATATCATGTAACAAAAGCGTTTCCTATAGACTATTCACTAGCTAGTCTCATAGGCATGTGTAATATCGATCTTTTTTTCCCCGACAGGAGGCGGTTGTTATTCCTCCATATGTTGCCTTTGCCATTAGACCAAATCCTGGGTTCTGGGAATTCATTAAGGTGAGCTCTACCGATCTATCGGTAGAGGGCATCACTGCCACGGACTACTTGAAATACAAAGAAATGTTGGTTGATGAGGACTGGTATATCAATTACTTGCAGCTTATTACTAAAATTTATTTCATTTCAAAGCTGAAGATTTACTAAAATTTAGTCTGAAGATTTACTAAAATTTAGTTATTTATGTTTTCTTATATTATTGCAGGGCAAAGGATGAAAATGCATTGGAAGTTGATTTTGGAGCGATGGACTTTTCCGCGCCTAGCCTGACCGTGTCTTCTTCGATTGGAAATGGAATCAATTTCGTTTCCAAATTCCTTTCTTCTAAACTACATGGTGGCTCACAGAAGGCTCAGCCTCTTGTTGATTACTTACTCTCACTAAATTACCATGAAGAAGTATGGAAACAATTTTTTTTATTGCAAACAAAATTAGTTAGTACAAGTAATTTTAAAATTTCTACACTAAAATACTATGAATATTTGTTTTGATCATGGTTTGGTCTTAAAATTTGCAGAAACTAATGATTAACGAGACCATCAACACCGCTGCAAAGCTTCAGAGCGCGCTAATAGTAGCTGAAGCGGCCCTTTTGACACTGCCCAAGGACACACCATACCAGGACTTTGAGCAAAGGTGAATGATTCTAATTTTTATCTCGAAAAAAATCTATGGTTTGTGTTGAATTCATAGCTAACATGGACGCTTTTTTTACAGGTTTAGGCAGTGGGGATTTGAGAAGGGATGGGGCGATACTGCGGAAAGAGTGAGGGAGACAATGAGATCGCTTTCAGAGATATTCCAGGCACCGGACCCGTTAAATATGGAGAAGTTCTTTGGCAGGGTTCCAACGATTTTCAAAGTCGTTTTATTCTCGGTCCATGGGTATTTTGGACAATCTGATGTCCTCGGTTTGCCAGACACCGGTGGGCAGGTACACATTTTTGCCAGCACATCAATCTTATGTTTCCTAATTTTACCCCGAAAAGTATCCAGAATTTAAAATGTGCGGATCAGATTCGTTCTGGTCACCAAGTTTAAATCTATACCTTAGAATTTGAAACTTCACTTTGTTACAAACAACAGGTGGTCTATGTTTTAGATCAAGTAGTTGCTTTTGAAGAAGAACTGCTTGTTCGGATTAAGCAGCAAGGGCTTAATGTGAAGCCTCAAATTCTTGTGGTGAGTACCAAATTTTGCTCAAAATTCTTTTACTATCTATGGCATTAATCATATATATATATATATATATATATATATATATACTCTTCTTCATTATATAATAGGTTACGCGACTCATCCCCGATGCCAAGGGGACTAAGTGCAACCAGGCGTTGGAACCGGTCGCCAACACCAAGCACTCTAACATCCTTCGGGTTCCATTTAGGACAGAAAATGGAGATCTTCCGCAATGGGTTTCCCGTTTCGACATCTACCCCTACCTCGAAAGGTTCACTCAGGCATGTTCTGCATCAATCATCAAATACACAACTTTCCAATTTTCAATTTCAATTTCGTCTTCTGTTTCTCAGTATCACTTTATTTTTCAATCAACAGGACGCTACGGACAAAATCTTGGAAATCATGGAAGGGAAACCGGATCTAGTCATTGGAAACTACACAGATGGGAATCTGGTGGCATCACTCATGGCTAGCAAACTTGGCATAACTCTGGTGCCTCCATAAGTTCTATTTTTCTCTTTTAGCTTTGTACTTATTGAGAATAACGTTTACTAACTTGGAATGTTCTGAAATAGGGAACTATTGCACATGCTTTGGAGAAGACAAAGTATGAGGATTCAGACCTAAAATGGAAAGAATTGGACCCCAAGTATCACTTCTCCTGCCAATTCACCGCTGACACGATCGCAATGAATTCTTCAGATTTCATCATCACTAGCACATACCAAGAAATTGCTGGAAGGTTTTTATTTTTCGTACAAAATGAAAGCAGTAGTTTTTATTTTCGTACAAAACGAAAGCAAAAGTTCCATACAAATGTACTTATTGAAATATGAATCTTTTGTTGTTGCAGCAAGGATAGGCCGGGACAGTACGAAAGTCATGCTGCATTTACGCTTCCAGGGCTTTGCAGAGTTGTTTCAGGCATAAATGTGTTTGATCCCAAATTCAATATAGCTGCTCCCGGGGCTGACCAATCCGTCTATTTCCCTTACACCGATAGACCGAAGCGATTCACAAAGTTTCATCCTTCCATAGAAGAACTACTCTTTAGCAAAGTTGATAACATTGAGCACATGTAAGCGTTGAGAGTTTCTAATTGTTCATTTTTTTCAAGCATCGAAGGCCAACCCCAATATTAATTTACTCTATTTTTTATCTCAAATTTCATCGGATTTGAGTAAAAATTTCAAATTTGATGGAGGATTAGGTTAGCCCTTGATTTGATACAACTTCGGTGAAAGGATTGAAGATGATTTTGGTCCTTTTTTAAAGAGTTTCTAAAAATAATACTGTCAATAGGATTATTTGTTTGAAGCATCTAAGGCCATCTCCAATCTTCGCTCTATTTTTCATCAGATTTGAAAACAATAGATTGTTAAAAGCTTGCTCTGATGTGTTAACACTCCAAACCTTACTCTTTTTTTCCCCCATCAACTCTTTCTAAAAACAGCCCATCTTATCCCCGAAACCCCATTTTGATGAAAAAATTCGAGCTCATTTTGTTTTTTTATTCATTGAAAATATGAATCTGATGAAGGATTAGAGATGCCGTAACACCTTATTTGAAATGTTTCCAAAATCAGTGGATATCTAGAAGACAGGAAGAAGCCTATTCTCTTCTCAATGGCAAGGCTTGATATAGTGAAGAACATCAGCGGATTAACCGAGTGGTACGGGAAGAACAAGAGGCTTAGAAGTTTGGTTAATCTCGTTGTTGTCGCGGGGTTCTTCGACCCTACTAAATCCAAAGACCGAGAAGAGGCAGCCGAAATAAAGAAGATGCACATGTTGATCGAGAAATACCAACTTAAGGGTCAGATTCGATGGATAGCAGCACAGACCGACAGGCAACGAAACGGAGAGCTGTACCGTTGCATTGCTGACACGAAAGGAGCGTTCGCGCAGCCTGCACTTTATGAGGCGTTTGGCCTCACGGTTATTGAGGCGATGAACTGTGGATTGCCTACTTTTGCAACCAACCAAGGAGGCCCCGCGGAGATCATTGTTGATGGGCTTTCAGGGTTCCATATTGATCCTAATAATGGGGATGAGTCAGGGAACAAGATTGCTGATTTTTTCCAGAAGTGCAAGGACGATCCCGAGCACTGGAACAAGATTTCCAAGTTGGGTTTGAACCGTATCTATGAATGGTAAACAACCTTTGTCTCTTCGATTCGCAGTCTCTATATCTTGAGATCTTTTTTAAATAAAATTCTACAAGTTTGAGTTACATTTGTGATTACGCCATTGTTTGCTATGTAACTTTTTTGTTTTTGCAGCTATACATGGAAGATTTATGCAAACAAGGTGTTGAACATGGGGTGTGTGTATAGTTTTTGGAGGCAGTTGAACAAGGACCAGAAGCACGCAAAGCAAAGATACATCCAAATGTTTTATAATCTCCAATTCAGGAACTTGGTATGTTGCTGCATTAAACATGACAGATTTTGCTCAAATTTTGCCCTTATGTGACTTGGAATTGACTACTTTTTAACCCTCACAGGTGAAGAATGTCCCCACTTCAAGGGTTGAACCTCAACAACAGCCCAAGGAAAAACAACCCAAAGCACAGCCCTCTCAGAGGTACAGTCGATCCACGAAAACAGTAAAGAAACAGAAACAATCATGCACGTAAACGAACACAAATTGTGAAGTTCCACCACAAATCCTTTCATTTCAAAGTCACTCGTCCAATCATACGATAAACCTATTTCTTGTTACCAATATACGTAACCATACAACACCTTTCATTTAGTTAGGGAATTTTCTAGCAAACTAATTGATGATTTTTTTTTTTGTAGCATCAAGCGCACACAAAGTCGGTTTCAGAGGTAAAGATTACAATTCAAGTTGACTGATTTTGTTTCATTCCTATCGTTGGTTAGGTAATTACCAAATTCACTCCAACAAGTTGAATGATTTCTCCAGGTTGTTCGGATCGTGAAGACTCAAACACAAGACAGCTTGATTCAGTGCCTCCCTGTTGTTATTCTTTCTTTTTTTCTTAAAAAAAAATAAATTTTCATTTTTTACAAGCATAAGAAAAGGGATTTGTGTACTGAACTAATAAAAGAGAATAATGGTGTATTTGATCTGATGCAGATCATTAAATTCATTGATGTGTACATGGAAATATTCAATG

>AeSUS4

ATGGCAGCCTTGAAGAGGTCTGAGTCGATAGCTGATAGCATGCCGGATGCCTTGAGAGAGAGCCGGTACCACATGAAGAAGTGCTTTGCTAAGTACATTGAGAAGGGGAAGAGGTTGATGAAACTTCACCACTTAATGAGCGAAATGGAGAAAGTGATCGACGAAAGGACGAGAGAGAGCAGGTCTTGAATGGCCTTCTTGGCTACATTTTATGCACCACACAGGTATATTTTCATCTCGAGTAATGATATACAAACACCAACATCATTCCCCAGATATATACAAACTCTTTTAGCAGATTACTGTCTAGTCACATCATTCTGGTCGATAATTATGTATCCCTATAGACTATTCCTGTAATATCACTTTTTTTTTCTCTCGACAGGAGGCAGTTGTTATTCCTCCATATGTTGCCTTCGCAATTAGACCAAATCCCGGGTTCTGGGAATTCGTTAAAGTGAGCTCTACAGATCTATCAGTAGAGGGCATCACCGCCACGGACTACTTGAAATCCAAAGAAATGCTGGTTGATGAGGACTGGTATACCGATTACTTGCAGCCTGTTACCAAAATTTATTTCATTTTAAAGCTGAAGATTTACTAAAATTTAGGTATTTGTGTTTGCTGATATTGCAGGGCAAAGGATGAAAATGCTTTAGAAGTTGATTTTGGCGCGATGGACTTTTCCGCGCCTAACCTGACCATGTCTTCTTCGATTGGGAACGGAATCAATTTCATTTCCAAATTCCTTTCTTCTATACTATATGGTGGCTCACAGAAGGCTCAGCCTCTTGTTGATTACCTACTCTCACTAAATCACCATGAAGAAGTATGGAAACAACTTTTTTTAATTGCAAACAAAATTAGTTAGTACAAGTAATTTTAAAATTTCTACAGAAAAATACTAAGAATATTTGTTTTTATTAAGGTTTGGTCTTAAAATTTACAGAAACTAATGATTAACGAGACCCTCAACACCGCTGCCAAGCTTCAGAGCGCGCTAATAGTAGCTGAAGCGGCCCTTTTGACACTGCCCAAGGACACACCATACCAGGACTTTGAGCAAAGGTGATTGATTCTAATTTTTATTTCAAAAAAATCTATGGTTTGTGTCGAATTGATAGCTAATACCGATGCTTTTTTTACAGGTTTAGGCAGTGGGGTTTTGAGAAGGGATGGGGCGATACTGCAGAAAGAGTGAGGGAGACAATGAGATCGCTTTCAGAGATATTCCAGGCACCGGACCCGTTAAATATGGACAAGTTCTTTGGCAGGGTTCCAACTGTTTTCAATGTCGTTTTGTTCTCGGTCCATGGGTATTTTGGTCAATCTGATGTCCTTGGTTTGCCAGATACCGGTGGGCAGGTACACATTTTTTCCAGCACATCAATCTGATGGTTCCTAATTTTATCAGAAAAGTACCCAGAAATTGAAATGTACGGATCAGATTCATTGTGGCTGCATTAAATCTACACCTTAGACTTTGAAACTTTGCTTTGTTACAAACAACAGGTGGTCTATGTTTTGGATCAAGTAGTTGCTTTTGAAGAAGAACTGCTCATTCGGATTAAGCAGCAAGGGCTTAATGTGAAGCCTCAAATTCTTGTGGTGAGTACCAAATTTTGCTCAAAATTCTTTCGCTAACTATAGCATTAATCATATATACACTCTTCTTGGTTACAATAGGTCACTCGACTTATCCCTGATGCCAAGGGGACTAAGTGCAACCAGGTGCTAGAACCGATCGCCAACACAAAGCATTCCAACATTCTTCGCGTGCCATTTAGGACGGAAGATGGAGTTCTTCCGCAATGGGTTTCTCGTTTCGACATCTATCCCTACCTCGAAAGGTTCACTCAGGCATGTTCTACATCAATCATCAAATACAAAACTTTCCAAATTTCGTTTTCAATTTCGCCTTTTGTTTCTCATTTTCAGTTCTGTTGTCAATCAACAGGACGCTACAGATAAAATCTTGGAAGTCATGGAAGGGAAACCGGATCTCATCATCGGAAACTACACAGATGGGAATTTGGTGGCATCACTCATGGCTAGCAAACTTGGGATTACTCTGGTGCCTCCATCAGTTTCATTTATTCATTTCTTTTTTCTGCAGTTTTGTACTAATAATTGAGAATTACATTTTACTAATTTGGAATGTTCTGAAATAGGGAACTATTGCACATGCTTTGGAGAAGACAAAGTATGAGGATTCAGACCTAAAATGGAAACAACTAGACCCCAAGTATCATTTCTCATGCCAATTCACCGCCGACACGATCGCAATGAATTCTGCAGATTTCATCATCACCAGCACATACCAAGAAATTGCTGGAAGGTTTAGTATTTAATTTCGTACAAAATGAAAACAATCGTGTTTATTTTTGTACAAAACGTAAACAAAAGTTACCATACAAATGTACTTATTGAAATATGAATCTTTTGTTGTTGCAGCAAAGATAGGCCGGGGCAGTATGAAAGCCACGCTGCATTTACACTTCCAGGGCTTTGCAGAGTTGTTTCAGGCATAAATGTGTTTGATCCCAAATTCAATATAGCTGCTCCCGGGGCAGACCAATCCGTCTATTTCCCTTACACAGATAGACAGAAGCGATTCACTTCGTTTCGTCCTGCCATAGAAGAACTACTCTTTAGCAAAGTTGATAACAATGAGCACATGTAAGTGTTGAAAGTTGCTAATAGTTCCTTTTTTTGAAGCATCTAAGGCCATCTCCAATCTTAATTTACTCTATTTTTCATCTCAAATTTCATCGGATTTTAGTAAAAAATTGAAATTTGGAGGATTAGGTTAGCCCTTGATTTGATACCACTTCGGTGAAAGGATTGAAGCTGATTTTGGTCCTTGTTTAAAGAGTTTCTAATTATGAAACTGTCAATTGGATTACTTGTTTGAAGCATCTAAGGCCATCTCCAATCTTCACTCTATTTTTCATCAGATTTCGAAACAAAAAAATTGTTAGAAGCTTGCTCTGATGTGTTAACACTCCAAACCATAACTTTTTTTCATCAACTCTCTCTAAAAATTGCCCAGCTTATCCCATCAACCATTTTTTTTATGAAAAGAAAACTATCTTCAATCCTTATTTGAAATTTTGATACCATTTTGATGAAAAGATTCGAGCTCAGTTTGTTTTTTTATTCATCGAATATGAATTTGATGAAGAATTAGAGATGCCCTAACCCTTTTTGAAATGTTTCCAAAATCAGTGGATATCTAGAAGACAGGAGAAGCCTATTCTGTTCTCAATGGCAAGGCTTGATATAGTGAAGAACATCAGTGGATTAACGGAGTGGTACGGGAAGAACAAGAGGCTGAGAAGTTTGGCTAATCTCGTTGTTGTCGCAGGGTTCTTCGATCCTACTAAATCCAAAGACAGAGAGGCAGCCCGAAAAATAAACAAAAATGCACATGGTGTGATTGAGAATTACAAATGCAAGGGTCAGATTCGATGGATAGACAGCACAGACTGACAGGCAACGAAACGGGGAGCTGTACCGTTGCATTGCTGACACAAAAGGAGCATTTGTGCAGCCTGCACTTTACGAGGCGTTTGGCCTCACGGTTATTGAGGCAATGAACTGTGGATTACCTACTTTTGCAACGAATCAGGGAGGCCCCGCAGAGATCATTGTTGATGGGGTTTCGGGGTTCCATATTGATCCGAATAATGGGGATGAGTCGGGGAACAAGATTGCTGATTTTTTCCAAAAGTGCAAGGACGATCCCGATCACTGGGACAGGATCCAATGTGGCGTTTGAACCGTAGTCTGATGAATGGTAGTAACAGATTTGATGTGCTCTTCCCCCAATCTTGCTGTTTTTCGATTCAACTGAAAATCCAGAGTATAGTAAATCTGAGTTTCTTTTTTAAATAAATACCATATAATATATATCCTCAATTTTTTTGTACTAGTAATTCAACAAGTTTGAGTCACATTTGTGATATTAGTGCAAATATATGATAATACAACATACGATTTTGAAACAAACATTCCTGATTACGCACTGTTTCTATGATACCTTTTGTGTTGGACCAGCTATACAGGAAGATTATGCAACAGGTGTTGAACATGGGGTGTGTATATAGTTTTTGGAGGCATTTGAAAAAGGACCAGAAGCAAGCAAAGCAAAGATACATCCAAATGTTTTATAATCTCCAATTCAGGAACTTGGTATGTTACTGCATTAAACATAACAGATTTTGCTCTTACGTGACTTGAATTGACAACTTTTTAACTCTCACAGGTGAAGAATGTCCCCACTTCAAGGGTTGAACCTCAACAACAGCCTAAGGAAAAACAACCCAAAGCACAGCCCTCTCAAAAGTATGGTACCCAACTTCTCCCCACATTAAGTTTCCAGAACCACGTTCAGTTACTCTGTTATATTTTTTGTTATGCGATAACAGGATCGGTAGATCCACGAAAACAGTAAAGAAATAGAAACAATCATGCATGTAAAAGAACACAAAGATTTACTAAAAGAACACACAGATTTACGTGGTTCGGCAGTGTGCCTATATCCACGGCAGAGTGAGAGAATAGAAATCCATTTTACACAATAAGAATTACAAGAATCAACCTTTCTGTGTTCTCTCCCAAATTATAAACACTCTCTTACTCCAAAACCCTAATCTAAGATTATAATAAATACCCAAAATTCGGGGCGGGGGGCAATGTCCCCAAAAGTTCTGGCTCTGGGGCCTCAACCTATCAATTTCAACTACTCCACCTCCATAACTGGCCTTAACAGTAACTTAGAGAGAAATACAAGATTGCATAACTTATTTTAGTTTAGGATTTTTCTTGCCAACTAATTGATGATTTTTTTTTTGGTAGCGTCAAGCGCACACAAAGTCGGTTTCAGAGGTAAAAATTACAGTTCAAGTTGACTGATTGTTGTGTTTCACTTTCTACTGTTGATTATAGGTAACAAAACCACTCAAACAAGTTCAATAATCTCCAGGTTGTTCGGATCTTGAAGACTCAAAACACAAGGC

>AeSUS5

ATGGCTTCCGCAAAAGTTCTTAGGAAGTCGGACAGTGCAATAGCCGAGAGCTTGTCGGATGCTCTAAAGCAGAGCCGGTACCACACGAAGAGATGCTTTGCTAGGTATGCAATTCTTTTTTGTTCATAGAATTTCTCATTTTCCCCCCCTAATTGGCTAACAAAATGGGACTTTGTGTGTGTGGCAAATTAAGGTTTGTTGAAACGGGGAAGAGGTTGATGAAACCCCGTCATTTAATTGAGGAAATGGAGAAGGTGATTGGGGACAAGAGCGAAAGAGCCAAAGTTTTGGAGGGTTTACTCGGTCTCATCATCAGTTCCACTCAGGTATTGAAGTCACTCGCTCTCTATATCTATTTATCTATCTATCTATAGATATATATAGATGAATGTAGAATATTGGTGTTTGTGTGGAAAAATTCGGTAATTTCATGTGAATAATTGCAGGAGGCAGCTGTGGTTCCACCAAATGTTGCTTTGGCAGTGAGGAGGAGCCCCGGTTTCTGGGAGTTTTTCAAGGTGAATGTTGATGATCTAACCGTGGATGCTATTTCGGCTAAAGACTACTTGAAGCTCAAAGAAACAATCTGCGACGAGAATTGGTGTGTGCGGAACTAATGTTTCATATTAATACATACTCCTAGTTTTCATGAGAATTGGTTCTTATGCTAAGTTTGAGATGATTTGATATGCAGGGCAAAGGATGAAAATGCATTGGAATTGGATTTTGGTGCATTTGATTTCTCCAGTCGTCGCCTAACCCTTTCTTCTTCGATTGGAAATGGGGTCGATTTCATCTCAAAGTTCATGGCTTCAAAGACTAGTGGGGATCTTGAGCATTCGAAGCCTTTGCTCGAGTACTTGCTGGCGCTTAATCATCACGGGGAGGTATGCGAATTAGTAATTTGAAAGAGACCCAATAAAGATCAGATAAATTTGAAGCAGAAACTAACGGATTTGCTTTTTGTTTCCAATTTTTTGCAGAATCTAATGATCAATGAGACTCTCAACACATTTCCCAAGCTTCAAGAAGCGTTGATTGTAGCTGATGTTTACCTTTCTGCTCTCCCAAAAGACACACCCTACCAGAACTTTGAGAAAAAGTAAACATTTCTTGAAATTTCTTTATCGAAAGAAGTTTACAATCTACGATTATTATCCAAACCTATTTTCATGTTCAGGCTTAAAGATTGGGGCTTTGAGAAAGGGTGGGGAGATAATGCAGAAAGAGTTAGAGATACAATGACAATCCTTTCGGAGATATTCCAAGCACCGGACCCGACTAAAATGGAGTCTTTCTTTAGAAGACTTCCAAATATATTCAATATCGTGATCTTCTCGGTCCACGGTTACTTTGGCCAAGCTGACGTCCTCGGTTTGCCTGATACTGGAGGGCAGGTACATAACACAACCGGTTGCCTTTAGGTCTTGAAATGAAAATAAGTGCATGGGAAAAGTTATTCAAACATATCCGAAGTTTTTTCCTATATTTTCCCTCTACAAATCCCTCCAGTAATACACACGAGAACTCCATGTAAATCGAGGGCTAATGTGTTGAATTTAAACAGGTGGTTTACATTTTAGATCAAGTGAAAGCTTTGGAGGAAGAACTACTACTCAGAATTAAGCAGCAAGGATTGAGTGTGAAGCCTCAGATTCTTGTGGTGCGCTATGCCTAAAATTCACCACTAGTTGACCCAGCGGAGATAGTTTTGAGTGTTTAATGGTCTTTCTTGTTTTGCGTATATACGTTTATAGGTAACTCGTCTCATACCAGATGCACAAGGAACAAAGTGCAACCAGGAAATCGAGCCAGTCCTCAACACGGCACACTCCCACATTATTAGAGTCCCATTCATGACCGACAAAGGGGTTCTCCGCCAATGGGTTTCCCGGTTTGATATCTACCCGTATCTGGAGAGATTTACCCAGGCAAGTCCCTTTTGTTTTTCGATCTTCTAGTTGCAATTTCCTTTTACGATATTTTGTTTTCACTCATTACATTTACATCGTGTTATTAGGATGCTACTGCTAAGGTCCTTGGGCACTTGGAATGTAAACCAGACCTTATACTTGGGAACTATACTGATGGAAACTTGGTGGCCTCTCTAATGGCTAACAAACTTGGAGTCACCCTGGTTAGATCCTTTTCGAGAAGTTTCTACTTGTTTCTGACGGTTCAGTTTTTAGAATCAGTTTTGCAGCACAATTTTTCACAAACTATTTTTGAGAACAATTTTTTTTATTGTTTTCATAGATTATTATTTTAGCAGAAATAGTTTTTGTTTTCTCAAAACTGTGTTCTTTTATAAACTTTCCCATTGAAAAAGTTTTGAAAATCATTAACCATGCAAACATTCTAAAACTGTTTTGCAAAACAAATCCTGAAAATTAAACCATACAGAGCACTAAAGTTTGGCGTGCTTCAAATCTAGGGAACCATTGCTCATGCTTTAGAGAAGACTAAGTATGAAGATTCTGACATCAAATGGAAGGAGTTGGATCCAAAGTACCACTTCTCATGCCAATTCACAGCTGACATTATTGCGATGAATTCGGCCGATTTCATAATTACCAGCACATATCAAGAAATCGCAGGAAGGTCAGTTTGTTTGATACAAAACTATTGTCTCCATCGTTACCCATCTAACTAATTTTCTTTTCTTTATTTTTTTTCCCTTTCAGCAAGAATAGGCCTGGACAATATGAAAGCCATATGGCATTTACCATGCCGGGCCTTAGCAGAGTAGTTTCAGGCATCAATGTCTTTGACCCAAAGTTCAATATCGCTGCTCCCGGGGCTGAACAAGAAGTTTACTTTCCCTTCACCGAGAAAAACAAACGATTCACTTCATTTCATCCCAGCATCGAAGAGCTACTCTACAGTAAGGAGGATACCAGCGAACATATGTAAGACCATCTACCCCAAACTCCACTTGAGTCTCCGTTCAATCATCAAGAATTTGACGCAGACAAGTTGTTTGGAGGAAAAGTGAAGTAACGTACAAGGAAACTTAATTTCTTTAGTGACAAAAGAATTTTATAGGAAGTAATAAGGAATTTTTTTTGATAACTTTGTATCTGGAAAATCTTGTGTTTTTTAGGACGAGTTTTGAAGGAAAGCTAATATTACTGGAAACTTAAAATAAGAATCCCACGAGTTTCTTGCAAACTGAACAATCAAAAAATGCAAAAAACTGCATTTTTCCAATATTTTACTTTCCTTTCTTGACAATTGAACAATGCTAAGAGCAAAATGCTAACAAAATTTTGGCATTGACTATGAACCATGCTTGGGACAGAGGATTTCTAGCAGACAGAAAGAAACCAATCATCTTCTCGATGGCAAGGCTCGATACGGTGAAGAACATTTCCGGATTGACCGAGTGGTACGGGAAGAACAAAAGGCTCAGGAACTTGGCAAATCTTGTTGTTGTTGCGGGATTCTTCGATCCATCCAAATCAAAAGATAGGGAAGAAATTGCAGAAATCAACAAGATGCACGCTTTGATACAGAAGTACCAACTCAAGGGTCAGATCAGATGGATAGCAGCGCAAACCGACAGGTACCGCAACGGAGAGTTGTACCGATGCATTGCTGATACGAAGGGGGCTTTTGTGCAGCCGGCACTGTATGAAGCTTTTGGTCTGACAGTTATCGAAGCAATGAACTGTGGATTACCCACATTTGCAACCAACCAAGGTGGACCAGCGGAAATCATAGTCGATGGGGTTTCGGGTTTCCATGTTGATCCGAACAATGGCGATGAATCGAGCAACAAGATAGCCGATTTCTTTGATAAGTGCAAGGGGGATGCTGAGTATTGGAATAGGATGTCTAAAGCAGGTCTCCAGCGCATATACGAATGGTAAAAACTGGACCTAAAAGATCTAAAGAATTTATGTGTCTAAATTTGATTTTTATAGTCTCTAAGGCTGTTTTATTTGCAGCTACACATGGAAGATCTATGCAAACAAAGTCTTGAACATGGGGTCTTTATATGGCTTTTGGAAGCAGTTGAACATTGAACAGAAGAAAGCTAAGCAAAGATACCTTCAAATGTTTTATACTCTCCAATTCAGGAATCTGGTGGGTTACCTAGACTATATATATTCCATGCATTTATTCACAAGCAAATATTCGAAGCTATTTTTTCCATGTATTTAGTCGGTCCAGTGGGCCGGATACCGAGTTATCAAATAAGCTATATACGATGTTACAGGCAAACAAGATAGCCATCCCAACTGCTGAAGCCCAACAAATAGCACCAACTGTGGCTAAACCCCAAGAATCATCACAGACAAAATCACCTAAACCCTCGGAACAAAAGCCCATCGAGACAACACAAACTCAGCCGATGCCCAGGCATGTTTTCTAACTTTTATTGTAACTATGGGTCATAGTTTTTTTGTTTTTACAGTTCATATGTCACCTTGATTTCAAACATGCTTCAACTCGTATGATAATTGTGCTCAGGGAAAACGGGTTAGAGCAGCAGAAGCAGCTTGCCTCACCGAGAGATGCTCACACTTCCTGCCCCTGCTCTTCGTGGTGTTTCCTGTTTCTCTCTGTTTCCATCATTATCTACGCTGCTATGAAGTACTATGGCTTCTTCAGACAACCATGA

>AeSUS6

GGGGAAGAGGTTGATGAAACCCCGTCATTTAATTGAGGAAATGGAGAAGGTGATTGGGGACAAGAGCGAAAGAGCCAAAGTTTTGGAGGGTTTACTCGGTCTCATCATCAGTTCCACTCAGGTATTGAAGTCATCGCTCTCTATATCTATTTATCTATCTATCTATAGATATATCTGATGATGTAGAATATTGGTGTTTGTGTGGAAAAATTCGGTAATTTCATGTGAATAATTGCAGGAGGCAGCTGTGGTTCCACCAAATGTTGCTTTGGCAGTGAGGAGGAGCCCCGGTTTCTGGGAGTTTTTCAAGGTGAATGTTGATGATCTAACCGTGGATGCTATTTCGGCTAAAGACTACTTGAAGCTCAAAGAAACAATCTGCGACGAGAATTGGTGTGTGCGGAACTAATGTTTCATATTAATACATACTCCTAGTTTTCATGAGAATTGGTTCTTATGCTAAGTTTGAGATGATTTGATATGCAGGGCAAAGGATGAAAATGCATTGGAATTGGATTTTGGTGCATTTGATTTCTCCAGTCGTCGCCTAACCCTTTCTTCTTCGATTGGAAATGGGGTCGATTTCATCTCAAAGTTCATGGCTTCAAAGACTAGTGGGGATCTTGAGCATTCGAAGCCTTTGCTCGAGTACTTGCTGGCGCTTAATCATCACGGGGAGGTATGCGAATTAGTAATTTGAAAGAGACCCAATAAAGATCAGATAAATTTGAAGCAGAAACTAACGGATTTGCTTTTTGTTTCCAATTTTTTGCAGAATCTAATGATCAATGAGACTCTCAACACATTTCCCAAGCTTCAAGAAGCGTTGATTGTAGCTGATGTTTACCTTTCTGCTCTCCCAAAAGACACACCCTACCAGAACTTTGAGAAAAAGTAAACATTTCTTGAAATTTCTTTATCGAAAGAAGTTTACAATCTACGATTATTATCCAAACCTATTTTCATGTTCAGGCTTAAAGATTGGGGCTTTGAGAAAGGGTGGGGAGATAATGCAGAAAGAGTTAGAGATACAATGACAATCCTTTCGGAGATATTCCAAGCACCGGACCCGACTAAAATGGAGTCTTTCTTTAGAAGACTTCCAAATATATTCAATATCGTGATCTTCTCGGTCCACGGTTACTTTGGCCAAGCTGACGTCCTCGGTTTGCCTGATACTGGAGGGCAGGTACATAACACAACCGGTTGCCTTTAGGTCTTGAAATGAAAATAAGTGCATGGGAAAAGTTATTCAAACATATCCGAAGTTTTTTCCTATATTTTCCCTCTACAAATCCCTCCAGTAATACACACGAGAACTCCATGTAAATCGAGGGCTAATGTGTTGAATTTAAACAGGTGGTTTACATTTTAGATCAAGTGAAAGCTTTGGAGGAAGAACTACTACTCAGAATTAAGCAGCAAGGATTGAGTGTGAAGCCTCAGATTCTTGTGGTGCGCTATGCCTAAAATTCACCACTAGTTGACCCAGCGGAGATAGTTTTGAGTGTTTAATGGTCTTTCTTGTTTTGCGTATATACGTTTATAGGTAACTCGTCTCATACCAGATGCACAAGGAACAAAGTGCAACCAGGAAATCGAGCCAGTCCTCAACACGGCACACTCCCACATTATTAGAGTCCCATTCATGACCGACAAAGGGGTTCTCCGCCAATGGGTTTCCCGGTTTGATATCTACCCGTATCTGGAGAGATTTACCCAGGCAAGTCCCTTTTGTTTTTCGATCTTCTAGTTGCAATTTCCTTTTACGATATTTTGTTTTCACTCATTACATTTACATCGTGTTATTAGGATGCTACTGCTAAGGTCCTTGGGCACTTGGAATGTAAACCAGACCTTATACTTGGGAACTATACTGATGGAAACTTGGTGGCCTCTCTAATGGCTAACAAACTTGGAGTCACCCTGGTTAGATCCTTTTCGAGAAGTTTCTACTTGTTTCTGACGGTTCAGTTTTTAGAATCAGTTTTGCAGCACAATTTTTCACAAACTATTTTTGAGAACAATTTTTTTTATTGTTTTCATAGATTATTATTTTAGCAGAAATAGTTTTTGTTTTCTCAAAACTGTGTTCTTTTATAAACTTTCCCATTGAAAAAGTTTTGAAAATCATTAACCATGCAAACATTCTAAAACTGTTTTGCAAAACAAATCCTGAAAATTAACCATACAGAGCACTAAAGTTTGGCGTGCTTCAAATCTAGGGAACCATTGCTCATGCTTTAGAGAAGACTAAGTATGAAGATTCTGACATCAAATGGAAGGAGTTGGATCCAAAGTACCACTTCTCATGCCAATTCACAGCTGACATTATTGCGATGAATTCGGCCGATTTCATAATTACCAGCACATATCAAGAAATCGCAGGAAGGTCAGTTTGTTTGATACAAAACTATTGTCTCCATCGTTACCCATCTAACTAATTTTCTTTTCTTTATTTTTTTCCCCTTTCAGCAAGAATAGGCCTGGACAATATGAAAGCCATATGGCATTTACCATGCCAGGCCTTAGCAGAGTAGTTTCAGGCATCAATGTCTTTGACCCAAAGTTCAATATCGCTGCTCCCGGGGCTGAACAAGAAGTTTACTTTCCCTTCACCGAGAAAAACAAACGATTCACTTCATTTCATCCCAGCATCGAAGAGCTACTCTACAGTAAGGAGGATACCAGCGAACATATGTAAGACCATCTACCCCAAACTCCACTTGAGTCTCCGTTCAATCATCAAGAATTTGACGCAGACAAGTTGTTTGGAGGAAAAGTGAAGCAACGTACGAGGAAACTCAATTTCTTTAGTGACAAAAGAATTTTATAGGAAGTAATAAGGAATTTTTTTTTATAACTTTGTATCTAGAAAATCTTGTGTTTTTTAGGTCGAGTTTTGAAGGAAAACTAATATTACTGGAAACTTAAAATAAGAATCCCACAAGTTTCTTGCAAACTGAACAATCAAAAAATGCAAGAAACTGCGTTTTTCCAATATTTTGCTTTCCTTTCTTGACAATTGAACAATGCTAAGAGAAAAATGCTATCAAAAATTTTGGCATTGACTATGAACCATGCTTGGGACAGAGGATTTCTAGCAGATAGAAAGAAACCAATCATCTTCTCGATGGCAAGGCTCGATACGGTGAAGAACATTTCCGGATTGACCGAGTGGTATGGCAAGAACAAAAGGCTCAGGAACCTGGCAAATCTTGTTGTCGTTGCGGGATTCTTCGATCCGTCCAAATCAAAAGATAGGGAAGAAATTGCGGAAATCAACAAGATGCATGCTTTGATACAGAAGTACCAACTCAAGGGTCAGATCAGATGGATAGCAGCACAAACGGACAGGTACCGCAACGGAGAGTTGTACAGATGCATTGCTGACACGAATGGGGCTTTCGTGCAGCCGGCGCTGTATGAAGCTTTTGGTCTGACAGTTATCGAAGCAATGAACTGTGGATTACCCACATTTGCAACCAACCAAGGTGGACCAGCGGAAATCATAGTCGATGGGGTTTCGGGTTTCCACGTTGATCCAAACAATGGCGACGAATCGAGTAACAAGATAGCCGATTTCTTTGAGAAGTGCAAGACGGATGCCGATTATTGGAATAGGATGTCTCAAGCAGGTCTCAAGCGCATCTACGAATGGTAAAAACTGGCCCTAAAAGATCTAAATAATTTATATATCTAAATCTGATTTTTTATAGTCTCTAAGGCTGTTCTATAATCTATTTGCAGCTACACATGGAAGATCTATGCAAACAAAGTCTTGAACATGGGGTCTTTATATGGCTTTTGGAAGCAGTTGAACAATGAACAGAAGAAAGCTAAGCAAAGATACCTTCAAATGTTTTATACTCTCCAATTCAGGAATCTGGTGGGTTACCTAGATATATATATTCCATGCATTTATTCACAAGCAAATATTCTAAGCTATTTTTTCCACGTATTTAGTCGGTCCAGTGGGCCGGATACCGAGTTATCAAATAAGTTATATACAATGTTACAGGCAAACAAAATAGCCATACCAAGTGCTGAAGCCCAACAAATAGCACCAACTGTGGCTAAACCCCAAGAATCAACACAGACAAAATCACCTAAACCCTCGGAACAAAAGCCCATCGAGACAACACAAACTCAGCCGATGCCCAGGCATGTTTTCTAACTTTTATTGTAACTATGGGCCATAGTTTTTTTTGTTTTTTACAGTACATATTTCACCGTGATTTCAAACATGCTTCAACTCGTATGATAATTGTTCTCAGGGAAAACGGGTTAGAGCAGCAGAAGCAGCTTGCCTCACCGAGAGATTCTCACACTTCCTGCCCCTGCTCTTCGTGGTGTTTCCTGTTTCTCTCTGTTTCCATCATTATCTACGCTGCTATGAAGTACTATGGCTTCTTCAGACAACCATATCCAATGTGTGGATGCTACCAAAATGTGCGATTCTTCTGA
