# Supplementary material for: Genome-wide identification and expression profiling analysis of sucrose synthase (SUS) and sucrose phosphate synthase (SPS) genes family in Actinidia chinensis and A. eriantha
Source: BMC Plant Biol. 2022 Apr 26;22:215. doi: 10.1186/s12870-022-03603-y (PMC9040251; doi:10.1186/s12870-022-03603-y)

Supplementary file 4 Phosphorylation site of the SUS and SPS gene families in *Actinidia*.

AcSPS1


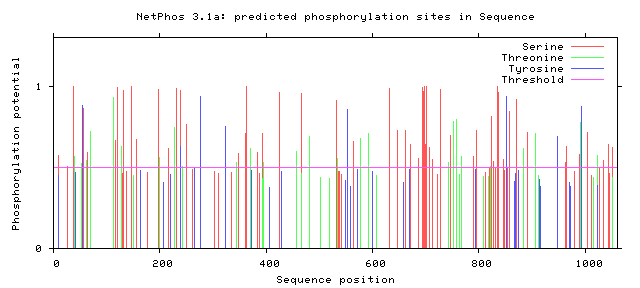


AcSPS2


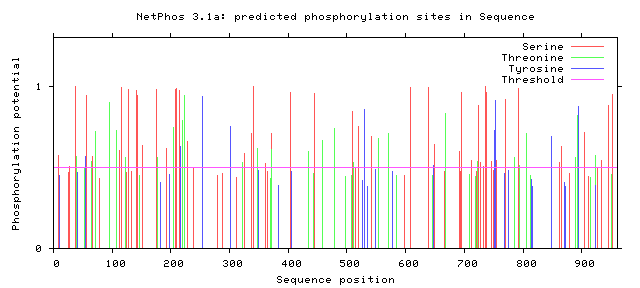


AcSPS3


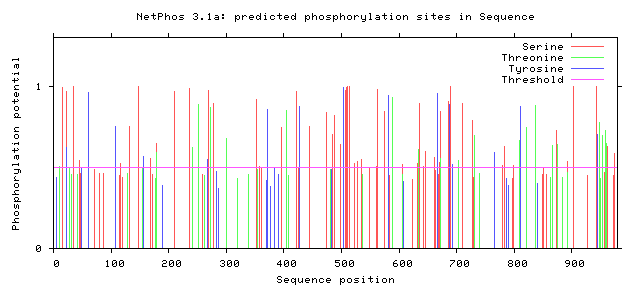


AcSPS4


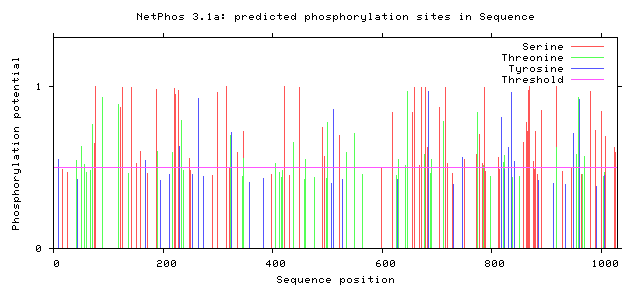
AcSPS5


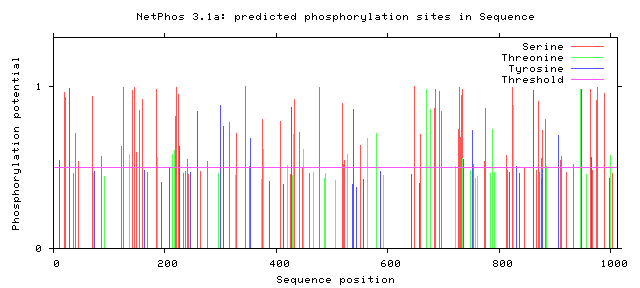


AcSPS6


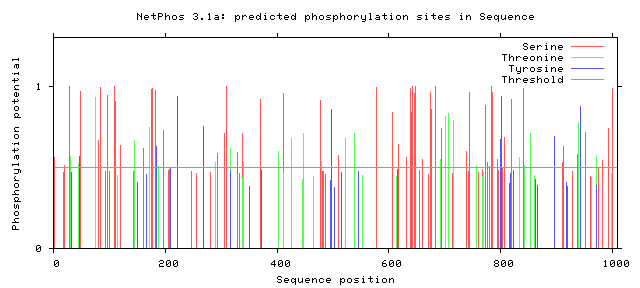


AcSUS1


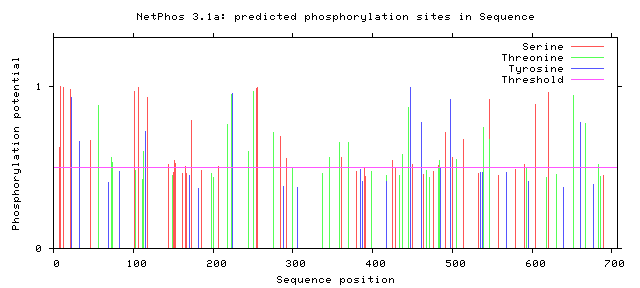


AcSUS2


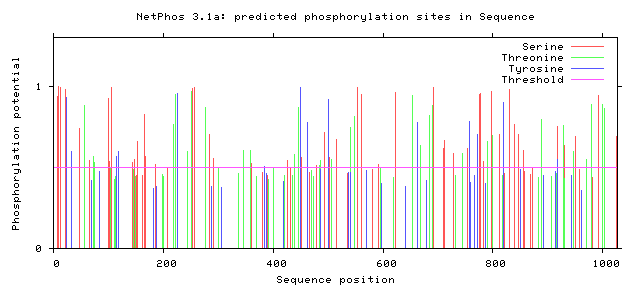


AcSUS3


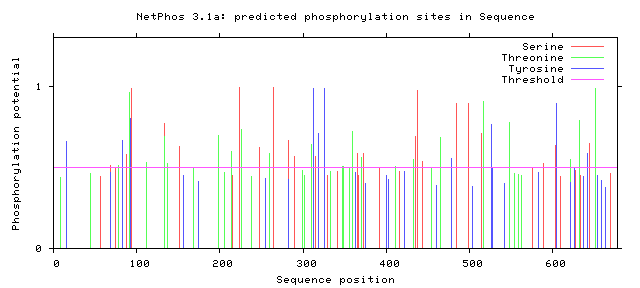


AcSUS4


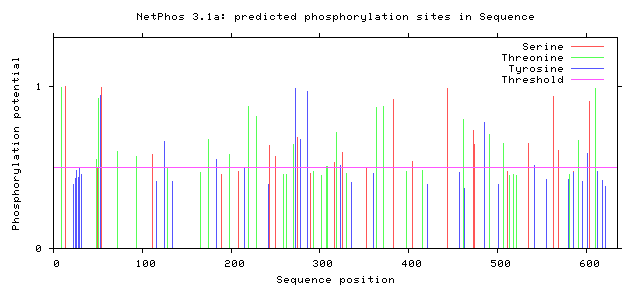


AcSUS5


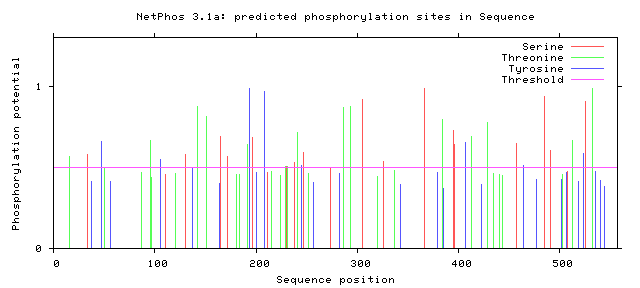


AcSUS6


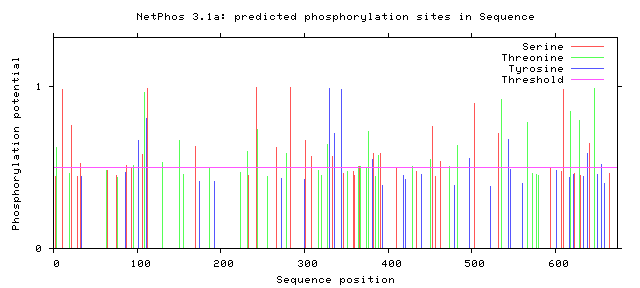


AeSPS1


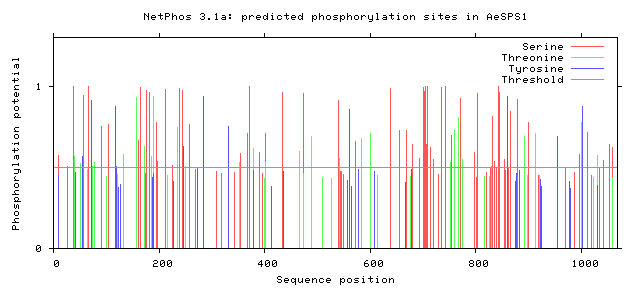


AeSPS2


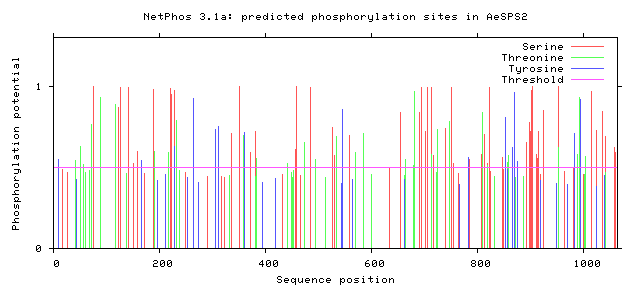


AeSPS3


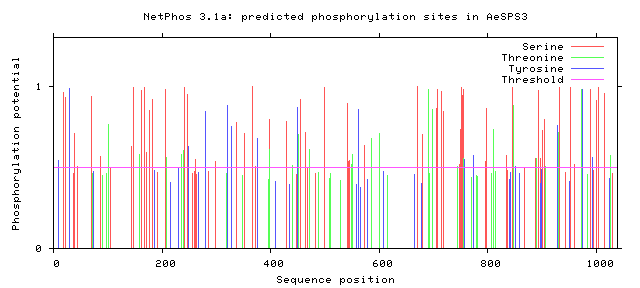


AeSUS1


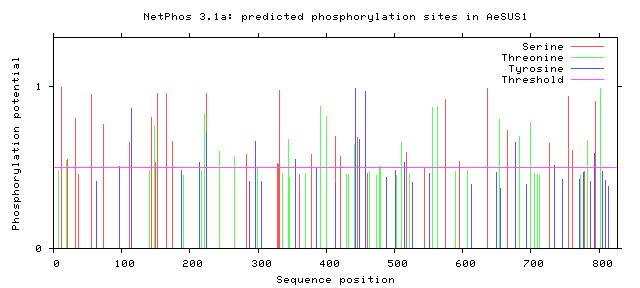


AeSUS2


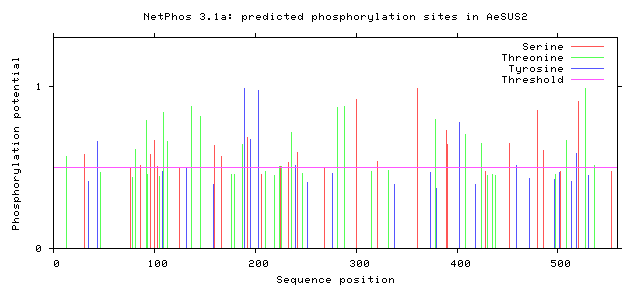


AeSUS3


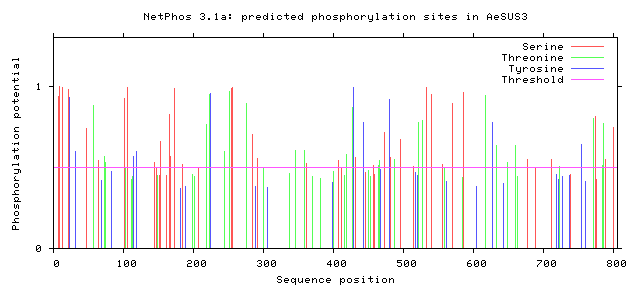


AeSUS4


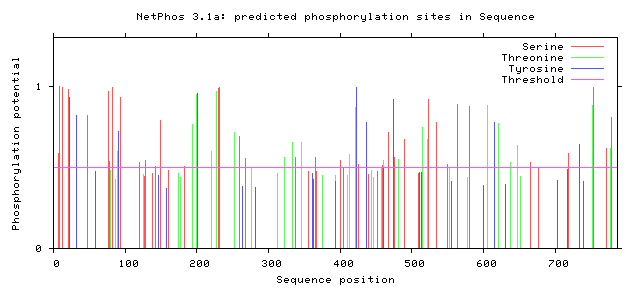


AeSUS5


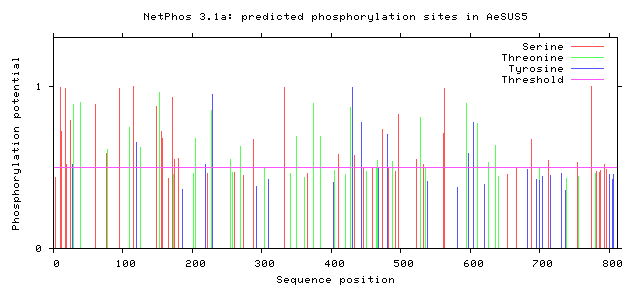


AeSUS6


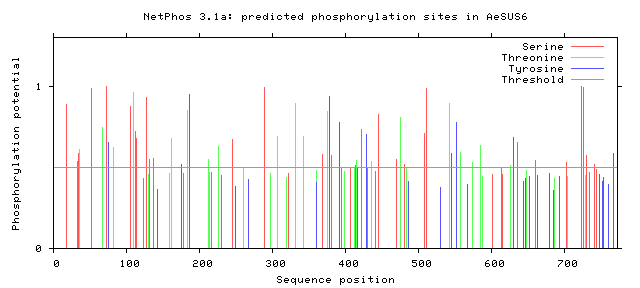

Supplement: Supplementary file 4 — Additional file 4. [file 12870_2022_3603_MOESM4_ESM.docx]
