# Supplementary material for: Genome-wide identification and expression profiling analysis of sucrose synthase (SUS) and sucrose phosphate synthase (SPS) genes family in Actinidia chinensis and A. eriantha
Source: BMC Plant Biol. 2022 Apr 26;22:215. doi: 10.1186/s12870-022-03603-y (PMC9040251; doi:10.1186/s12870-022-03603-y)
Supplement: Supplementary file 5 — Additional file 5. [file 12870_2022_3603_MOESM5_ESM.docx]

Supplementary file 5 Promoter sequences of members of the *SUS* and *SPS* gene families in *Actinidia*.

>AcSPS1 Chr13 Chr13:11490397..11492092

TTCTATAATCCCAGCTTTTGACCTAACATCTCTAAAGTTTATAAATTAATTAAACTCCACTGTGTATAATTGGGAAATTGTAGGCCTAATTTGAACAGTTCATCGTCTACCTGTTAAAAATGTCATTATAAAAATATTAAGTTGATTAAATATTAATAGTCACATTATCGAATAATATATATTTTGATGACAAATAAGTTTTTAGATATAAAATTAGTAAAAAATCACAAATGGGTGCAGGATTTACTAATCAAATGAGCCTTAAAAAATCATCAGTCCAACATAAAAAAATGGCCAAAAAAAATAAAATAAAATATTTTTTTTAATCAAAACACAGCTAAATAATCTAAGTGAAACGGAACCTAGGAAACAAACAAAATCCAAGCTCAACATTACAAATAAAAAAGAACTATGGATCAACCATGTTAGTATAAATTATAATTTACTAACAAATACGTGTACCCTACTTAATGATAAATAAAAAAAGTATCATACGTGTACGTATGACATTCTTGATAGGAACCCCGAATCATTCTTTCTATAAAGAATCTCATAGTTGTCCACGTAAAGTGTGGCGAGCTTATTAAAAAAATAAAAATTGCCAATTCATTGTGTAAGCACTTTTTAATCAAACAACTATTAAAATGCTAAGACAGATATTTATACACGAGTTGTGTGGGCTCGTATATTTATATATATTCTAAATGTGAGAGAATATATATTTGTGTGTATAAATAAAATATATTAGTATTATTAGTAATAATTATTGTTAAAAAAAAAAGCATTTGGGATATTAAATTCCACAATACCAATAATGCCCCTAAGAGATGGCAAAATGTAAGGGCAATTTTGACATAGTAAGTGCGTGGGAAACCGTAAACTTTAGGAGCTTCTAAATGCAAACCAAACCTATCTCAGGACACCCCAAATATCAAAAATAGTTTTATTACATTTTCATCGGTGTGTCAAATTACCATTGAAAACATCGTGGGCTGTACTGAGATAAAATAGGAAGCCCTAGTTTGGGTTGAGGTGAACTTAGAACATGGTTTGCATTTAGAAGCTCCCCTAAACTTTTGACGCATATTATTCGAATAATCTCAAATAATATTCCCTCAATCATTACAAGTTCACCGAATTAAAAAACTCACACCCACTGACTCTCATTTTTAAATACAATTTTGAGAAATAATTTGAACATTTAATTTAAAAATAAATTTTAATTGCATTAATTTTAGCTTGATAATGTGTCCTAATTGTATCACTTTTAAATGATAATAATTTTAAAACATTTTTTTTAATTTTTTTATAAAAATTAATTGACTATGCCAATTCTAATAAATGAATAATTTTAATTATTATCGAATCAAAATTTTAAAATTTAATTTATTTTAATTAAAATTATATATTATTATTGAAGTTAAACCATGAAATTATTTTTCTAATTTCCCCAAAATATCTCTTCCGCTTAAAACATCACAAAAACTCGCCGCCTCCATATATCAGCACTTTGTCGCTCTGTACATCCCGCAATCTCTTCTCTCTCTGCAAATCTTACAGTCACTACTGTCCTCATCGCATCACCGCCCCCCTCCACCAGAATCATCTCTCTCTCTAGATACAGTCACAGTCCTCGTCGCATCATCGAATCCCCAAACTGCGAACCAGAAATATCTCTCGCTATAGGGGCTGGTGA

>AcSPS2 Chr06 Chr06:12984877..12986572

ACCACCCCCTATATATATAGAGAGAGAGAGTTCTGGTTCGTCAGTTGGTGATTCGGTGATGCGACGAGGACACTGACGGTAAGGATCTAGAGAGAGAGGATTCAGGTTGAGGGGGTCGGTGAAGCGATGAGGACAGTAGCGACTGTAAGATTTGCAGAGAGAGGAGAGAGCACGAAGCACTTATATACGGGTAAATTTCTTCTCCCGCCCCGACTTAAGGGCCGGGCCGAGCTGGGCCGGCTTGGGCCGGGTCTGTTGAATTTTGATTGGTCAACTTGAGTTGGAGCCGGAGCCTACGAGATGAGACCAAATCCCAACCAATAGGCCGGGTGGAGCCGGAGTCTACCGGGCTTTAAATAATTTTTTTTTTATAAATATATTTTATTCTAAATTAAAATACTCAAAATATTAAATATGAAAAAAATAATGTGTAATTACATGAAATCATTTATAAATATTAATTCTTTACATAAAAAATATAAAATATTATAAACAATTAAAATTAAGTAGAAGGATTACATCTCTATAATATAAATATATCCCAACTAGGTTGACACATATATATCTGTCAATCATTTGCACAACTCTCCAAAAAATTAATTTCACGAGATAACAAAAAATAATATATAAAATAAAAAAAAATTATTTGTCATTCTTCTTCATCAAATGAACTATCCTCAATCCATGATGATCTTGATATTGATAAGTTTTTTATTTCAGATTCAAACTCTATCACAAGTTTTTCTTCTTATCATAAACGACCAATTTTTCAAACAAATTTGTGCTTCAACAGTTTCTGGATTTAGACTACTTCACATCTCAGATAGACACGACCACCTAAACTAAAAGCAGCTTCTGATGCAACAGTTAAAACAAAAACAATTAGAATATCACGGGCCATGGCGGAAAGCACATGACATCTAAGATCTTGTGTTTTTCACCGGCTAAAAAATTAAAATCGTTATCTTCAATATCAAGTATGAGCTGATCAAAATAGTGTTGCAATTTTGAATTAGAAGATATAAAACTTATTTGCATTCTTTTATTCTTAAACATTTGTAAAGCATCTCCTAATAAGGAGGATGAAGAAAATGAAAAGCCCCTTGCGGAAGTCGATGTAGACGGTCGATTTTGACCAATGATATTTTTATAATATTCGTATAAGTCAAATAGAGTTTTTCTTAGCGATGATATCCATTCTCTAATTTTGTCCATCTTATTTACATAAATAACTTCTAAACAATTATCTAAAAGTTCAAATTTATACATATGATCCATTACAATAGCCATACCGAAAATAAGTGGTATATTATCCAAATATTTATCATATTTTAGCATCATTGCATTTAATAAATCATTGAATATTTCTAGAGTTTTATAATTATTAAATACCAAATAAATATTAGTCAATTAAACAAGTACTTTACAAACAATTGGATAATAAATACCACAAAAAAATTTAGTTGAAATGTCAAAAACTTATAAAAAAAAATTTACAACATCAGTAATAGTCCAATCATATTCAGAAGGGGGTTGTACAGAACAATGGCGAATGTTTTGCAATAATTTCACATAATATAAATTCAAAACTCGTCTTTGTTTTAATGTTAATTTCAAAAGTGTATAAGTAGCATTCTAAGGTAAATCAACATTTATATTTTTTCTTTTCAAATTTATAGTTGCACAACATTTTCTA

>AcSPS3 Chr00 Chr00:26460537..26462232

GCCGATTCTTTTCACTGATGTCTGAAGAAGTAGAAAGATGATCAGAGTGAGATAACAATGATTTACATATTGCCTGTGCGCGATTATGCATACATGTATGAGAGAGAGAGAGAGAGAGAGAGATCAAATCTTGCTCATATAGAGATGAAGAGAGAGGGAGAGAATGAACAGAGAGAGATTGATAAACAGAGATCAGAGATGGTGACACGTGCAGAGAGTACTGATCCGTAAAGGCCCTGTATACGAGAGAGAGAGAGAGAGAGAGAGAGAGAGAGAGAGAGAGAGAGAGATGGATGTGGCTTTCTTCTGGAAAGAATTTTCAGGGACGTTGCAGTTGATCTGGGGTCGGTGCTATTTTTATTTAGTTTGAATTGTTTAGTCCTCGAATTTGTTTCAATTTTCAATATCATCCCCAAATTTTAATTCGTTTACATCTTAATCTTTTTAAGACCATGTCAATGGAATCCAAGTATCGAACAAAAACTTTTATCGTCATGTACTTAAAAGTAATTTTATGATCTCAGGAAAAAAATCCATTATGATAAAATAAACCAGATGAAGAAAAAGGTTTCAATTTTTGTTTAAAGTGCAAAACCACTTACAAATATTAAGAGGACGGCTCCTCTCCAGTTCGTTATCTGAACTAGATCGTTAATTCAATTTATTGAGCCATGTTTGAATCCACTTCGATATTCATAAAAAAAAAAATCAAATTGAATGGATAACGGTATTCTCATTTTCGGACTTAAAAGTTAAAATTATACGTATAATTGTATTTTCAGCTTTTTAGGGTATTTCATTTTTTAAAATTTTCAAGAAATAGTCAAAAAGCCATTGGGCATGTGCCACGTCTAACTGAATCGTTAAAATAGTAATATAGTATAGAGAGTTCGGATCCTCTAAAGTTTTTGTTGGGTGAAGTGATTTTAAAATATGCAAAACGATGTCGTTTTGTATATTTTATGACTAAAACGATATCGTTTTATGTTAACAACCAAAACAACGTCGTGTTGGTCCTTACATATACAAAACGACGTCATTTATACACGTTATGACCATTTCACTAAACAAATACTTCAAAGGATCCTAATCCTATTGTATAGTGTTGTTGATAAGATTAGGTCATTGCCTTGTAGCATTTCTAGCAGCTTTCTTGATTTTGTCTTCCTATTTTAGTTAAATCATATTTTGTCTAGTTATCTTTTTTTCACTTTCTCTAATAGTCTAAATAATAAAATTTTTAAATAGTACATTATATCTCTCTCTCGTCTGTCAATTAGCTCTGTTTGGGTAAATTTTTTGGGAGTTTTTTCAGATACTTTGGTAGTTTTTTTTGTCTTTTTGACAAATTTTTTTTACCATTCAATCAAAATTTTAAACGTTTTCGATTTCGTTTAATGAGGGAAGTTCAAAAAAATATTTTTTTTTGTTGAATTTAAAAAAAAAGTTTGTAAATAAAAAAATTTATTTTTATCCAAAAAAAATATTACCTAAAAGTTATTATTTTCGTCTTTAATAAACTTTATCAAATTAGATGAAAATTATATTTCTAACTCCATTCTTCAAAAGGAATCCGAAAAATATAAAATTTTGATCAAACTGATAAAAAAATCATTAAAGAAAAAAAAACGAAAACCATGAAAAACACTCAAAAAAATTAAAACCACAAGCATAACCCATCATCTCTCTTTTCAAT

>AcSPS4 Chr06 Chr06:8829243..8830938

AAATAAATGTGTATTTTAGTTTTTTTGTGGGTCCTACTAAAAATGATCAAATCGATAAAAAATATACCCAAATTTAAAGTTTTTTTGTCCATTTGATCAAAATTTATGTGGGTTTCATACCTTTTAGAAGAAAAAAAGTGATAGTTTTAGGGAGAAAAAAATAATCTCAGGTAAAATCTCTGTCGTTAAATTAATTCAAATGAATTTAAACCGTAGATAACAGGAAATATATTTGAAATTCAAAATTAATTATGTTTTATAATATAGGTTTACAACAATAATATTTTTTATTTAATAAATTAGGCAAAGAGTGAACAGAAAATTGAATCCACTTGATCTCTATAGGAGAAATTTGAATGGAAATCAGTAGAGTAGGGTCTAGTACGTGGGTTGGGTGAAACAAAATTTTATTAAATTTGAATCAATTATGTGAGAGGGATGATTCAAGTAGATCCACTGTCGTATGTGGGTACAAGCCACCAATACATGACTAGACAGGGGTGCCGTTACTTGTCATCCAGATAAAAAGAGATGGAGATGACTTTCTGAATTCAAATTTTTCTGGAGGGTCTGATGGTCGTTCAACAACCCCCAGCCCAATGACTTGCTGGGCTTACCACATGGCTGATCCTGTCTCTAGATAGAGTGATTAAGAGTAAGCATCGTCTCGTGTCCGTGTTATATTTCCTTCAATACTAATCTGATCTATTTATTTATTCGTGTTACAGTATCATGTCATTTCATATTCATGTTGTATTAATAAGCCATACCTAAAAATTTCAATCTTAATTAAAAATTTTAAGTATTATCAAATAAATCTCCATTGGATGATAATACTCATAAAAATGATTTTTATTACTCAATTATTTTTATTTTCATTTGAAAAGATTGATCCGGATGTGAAATATTATGTTTCTATATTATATGTACTCAAAGTTTGTGAACAAGAGGAATAAAATTACCTTAAATGAACAATAAAAATATCTTTATTGAATATTAACACATAATTTCTTTTTACATTCTTTAATTAAATTGGACCGTTGGATCATGTCTTATCATGAATTGAACTATACCATGTCACCTCTAAGTAAAAATATAGCTAAAAATGTATAATTAATTATTCATTAATTGTGTGTCATATCTAGTTATAAGGATTTGACTAATATGACTCTATTTATTTTTTATATTATCGAATTGTATTATAATAGTTTTATGTGAAAAATGGCTAATCATAACCTACATTTTATGCACGATTCGAGTCGTGTTAACAGGTTTGCTCTGGTAGTGAGCACTTCTCTAATTATTAACATATTTTGAATTATTTTAGGTAAATTTAAGCTTCTTTTGGTGAGGAACACCCTTGGTCTGATGTTCCTGCACCACACTCAATAGCCTCTCCTTCAGAACTGTTCCCGCTATAACAGGTGATCCCCCATAGACCCATTAAAACTCTCAATCTGTTTATCACACGTGTCACCTCTCTGGCCCCAGTTTTAACTGCCATGTGGTTCTCCCTCCCTCATCCCCCGCCCCCTCTCTCTCTCTCTCTCCTCCCCTGCTCTCTCTCACAACACAAACACTCCTCCTCCCAAAACTCTCTCATAACACCCCAGTTGAGCATTACCACTTCACATTCAGCACGAACCCATAATTTTCTCTCCCATTTTCTTGCGTATTTTCTCGGGAAAATCCGGGA

>AcSPS5 Chr10 Chr10:5211091..5212786

CAACAATCATACTACATGTTGAACCTCAGGCTTAAAACTATTATACCAACTCAAAGAAAGATTTGTGAATAGAAATTTAATAGATGCATTAAACAATTCTCCAGCTTCCCCATCACCGACAATCAGCCACCAGCACCACCAGGTACCACCACAAACACTCACCTCCATATCCCATGAAAGAGGGATTGGTTTTTAATGAGTTGGGGAAGGTGAGGGTGAGGGGGGATATGTAGCGTCAGGGCCGCCGGTCATGATAATTATGGGGCCCTAAGAGGGGATATTAGCGTAAGGAACCATAATTATGGAGCCCCAAGCAAGACTTTAATACGGGGCATTTATATATTTTAAGAAAAGATAAAGTTAACTTAAAACTTATTTTTCCAAATGCGGCTCTATTACTATATTCAACAATATTCTTTAAAAAAATTCTGTATTATAAAAATAATTATCTATCAATTTAGGTTCTCCATTAATAAAATTAGCTTTATGTTTGAACGTGATTTTCTGAGCTAGATCTACGTGAAAGTTTGTAAGATTACAAGCAATTACTTCAAGCTCTCCTCTAATAAAATGATTTCCTTATAGACTTGATATTATGGTAGAATTAGGCAGAATATTTTTTAAAAAGCATGATCTAGTTGTAATTTTTTCCAAAGTTAAAAAATCAAACTATTTTGGGCCCTTCATTTGATTGAGGCCTAATATTTTTTGGGTAAAGCATAAGGGTAGTACCTGAACGGGGGCCCTAATTTTGAACAAAAAATCGGGGGCAAAAGTGGCTGCTTCCTAGGCTTATGCACAGGGCCGGCTTAGGGTAGCGTTGAGGAGAGGATGGTAGGGTTTGCATGTCGTTGGGAAGGAGAGGGTTTAGAGGTCAGGTGCTAGGGGGGCTAGAGAAGGTGACAGGTGGAGGTGGTGGTGGTGGTGGTGGTGGTGGTGGTGGTAGAGGGTGGATTAGTATTAGGGTTTGGGATTTAGAGCGGTGGATTAAGTAAAAACCAAAATTGAAAATTTTGAATTAGCTACACCGAAAAGGTTTGTGTTGAGAAAACTAAAATTGTTTGCACATAGAAATTTGGCTAGATAGAACATTTAAGTGACGATTTTTCCCTTGCAAAATGCAATGCCTCTACTCGTGCATTATTTCCATCAAAATTCTTAACAACCGTTATCAATTGGGTTAAAATTGTTGCAATTGCTACTAGAGGGGACGAAATTGAGATTTTTGTGAAACTAGAGGAACAGTGTCTATATTTTGCCCTAGATTAATTTTACCATATAATTCGCTAGGCATGTTTTGACGAGAAAGAGAGAGAGGGAGAGAGAAAAGGGTAGCATTGTATGTGGAGAATAATAAAAGAATAAGGATGTCTGGCTAATATACCCTCGTAAAGGAAAGCAAGGGAACCGAAGGCACCACGCACCCCCTCCATATTTTGAGTAATTAATTAATTTGTTGTAAACTCAAAAACTAGCCTTTAAAAGCTGGCTATCTTCGCCTCTTCCAACTTGGTTTCCCACACCAAATCCTCCTACCATACCCCCTTCTCTCTCTCCAAAACTCTCACTCTACCTTCTCTCTCTTCCTATCTCTTCTAGTTGCGCCTTCTACAACAATATTGTTTTATTCATTTGATGTATACATCTTATTGTTTTTTGTGAAGTTATTTTTGGAAACGAAAACGGGGAGATAGAT

>AcSPS6 Chr00 Chr00:103040531..103042226

CCAAAAGCATCATCTCGACACTCTCTCGTGTGGGAGTTTGGGGTTTGAAAAGTGCGGTGTTGAAAATGACAGTTCGAATGGTAAAAATAAGTGCATCACCCTCTGCTCTATGGGTGCACCCACTCACAACACTCAGTTCCTTTTAGTTAATTTGGTATCAAAATTTTATATTTTTTATAAAAAAATTAAAAAATACGTGTGGAATACTTTTGACGTTTGAAAAATTACAATGACATTTGAAAAATTACTTCTTTTTGACAAAATGATGTTGATTTGAAGCCAAGAGTACTTTGTGTATTAACCCGGAATCTCAGAGGTGGTCATTGCAAAATGGAAAAATCTGTTATAAATTTCAAACCTCAGAGGAGGTCATTGTAATTTCTCAAACGTTAAAGGTACTTTGTGTATTAACCTGAAATTTTAGGGAAAGTCAGTGTAATTTATCCAAATTTTTTTTTTGTACACACCGGTTTAAAACATCTCTTATACGAGTCACATGGATCCCACTTATCACGTGTGGGGCCCATATATCCAATATGCGTGCGTTGTAAAAAAAATATTGATGTTGGCAGTATTATTGTTAGAATAAAAAAACATCTGGACTTTCTAATTCCAATAATGCCCCTAACAAGGGCAATTTTGACATATTAAGTGCATGGGAAACCTGTAATTTTTTTAAGCATATTACTAGAACAATCTCCATAATATTCCCTCCTTAATCATTACAAGCTCAATTAATTAAAAAAAAACACGCTCTCTGACTCTCGGCAGTATTGAAGCTGACTTCACTCTCAGCTTTCTTCGAACATTTGTCCTTATGCGCCGTCCCCACTTGAGAAAATACAAGTGGGCTTGTCACAGAGCGAGTATAGGCTTTTATCTACACCAATGTCTAAAAGAAACAATCAGCAAGATATTTGTGGTAATATGAAATGAGTAGAGTTTGAGAAAAATATAGCAAGTGAGTAGTCTTTTTCAGTAATTTGAGAGGAAGTAAATGCTTTGAAACTAATTTCATTTACAAGAAATATTAAATGAAGGGAAATTGGAACGATTTTGCCCCTCTAGTGTGATACACCATCCCTCTTTCTCTTTTCATTTTTACATTTTCTTTTCGTTATCGTATCTTTCTTTTTATCCAATTTTTTAAACTTTAAATGAGATAATCATTCATAGCTATTTATGTCCATTCCACTATTACTATTGTCATCATTTATGCGCACCACGGCAAAACATCCATGAAAAAATATCGATAATGCCATTTTTTCTTCACTGACACACCATTGCACCCATCCCCATTGGTTCCCCTTAATGCTTTCTCATCCTTCCCGCCACCTCCGTCCCTTACACTCCCTACCTTGCCAATGCCTGGCACCCACCCACACAAAAAACCATAATTGCAAACTCGATGTGATGCACTTCAAGTTGTGGAAGATGGGGTCAATGCAAGGGGAACTTATGCTCATGCATGTTTTACTTGCACCATTCACATTTTTCATTTATTACATTGACGACTGTTTCAGATATGTTCACTAAAATATTTTATTTTGCAAAAAAAATAAGAAAGATAATAGAAAAGTGAAGAGATAAGAGATAATAAAGACGATGATAACAAACATATAGATGGTGATCTATGGCGATTTTAAATTTATTCATACTGATAACAAGAGTAGAGAGCATAGAGTTGCGTGACAAC

>AcSUS1 Chr00 Chr00:87006579..87008274

ACACCCAACATTAATTTTTAGTTTTTAGTTTTTCCTTAGTACACCTATGTAATCCCCAAACTGCCTTCTTCTTCAACTTTCCACAACCCTAACCCTAACCCTAACCCTAGGCCACCTCAGCCGCCGCTACCTCCAAACCCCCTTCGAATAGGATGTCGTAGAGTTCTGGTTCGTCCCAGAGTGAGCAATGAAAACTCTGAATCTATCTGAATCGTCGATTACTATGAAAACCATGAAACACGAATCGATCTGAATCGATCAATGTTTGTGTAAATTAGGTAACGTTTCTTCGATCTTGTCTTTCTGGAAGGTTTACAGAATTTCGATTTTATACGCGTAAATTATGTATATCAAATGAAGGCCGTCTCGTGTTTTTGATCTTTGCAAGTGTTTATTTATTTATATTCTTGCTATTAGCAGCTGTTGAACGTGATAATTTATGCCTCTTTAAGAGGGCTTGTGTTGTTGAATTGAGGATTAGGGTATTCTGGTGGTAACAATGGTGAGTGATGGGTGGTCGGCGAAGGAGGGGAGGGGCTTAGGTGATTGGATAATGTGGAGGAGGGTGCTGTGGGAGGCGGGGAGATTGGGGGAAAGGAAGACGCTGGCTGCCGGGGGTTGAGAAGGGCTGGTGATGGCGGGAGATGGTGGAGGTGCTGGTAGTGTTGGAGGTTCGCTGGCGGTGTGGAAATTGGTGTCCGGAACGGTCATTTCATACTTGAAAAAAAATGTAATAAAAAAATTGTACTTTGGGGGTATTCCGAGACCAATTGGTTTGCATTTAGGAGCTTCCAGCATACTATGTCAATTTCCGTTTTCCCGTTCTTGGGGGGTTGATAAACCATTCCTAGAATTGGTAAACGAACAAAATTATTCGAACTTAGTTCGTGTTCAACTCATTAAAAACTTGTTTGAAATTAGTGTGTTATTTAAATAAATTAAATTTGAACATATTTTAAAAAATTTATTACAAATCAAGTTTGAACAATTTAATACTCGGTTAGATAAGATTATAATGTGTAAAAAATTCAAACTACTTGAGTTCGGTTCATGATCGGCTTAATTAAAACTCGTTTGAACTCGTTTTTTACAAACAAGCTAAGCTCAAACTTATTTTTAAAATTCGTTAAATTTTCAAGCCAAACTCGAACACTCCATGTTCACTTGTTTGACCCGTTTACCACTTCCCTGCACATTCATTTAGCATTTCCATAATCAATAATCTTATCAGTTTTCTTTTATCATCGGCCCTACATAGTCTATATTATATGTGATCGAACTATCCTAATCTATTTTCTCTCAATTTATATCCCATTGTTCCTACCCCTGGCAAATTCCAATTGGTGGATTTGAATATATATATATATATATATATATATATAGAGAGAGAGAGTGAGAGAGAGATTACCTTTGTTACGCACCACGAATCTCCTTTCTGTGACAAAATTCTTTTCTTTCGCTCTTTCCTGGATTTAGAGAATGTTCTTTCTCCTCTTGTTACATCCTACTTTCCTTCTCTTGTGCCATTACCAATTTCAAAAAAACAAGAACTCTCATAACCAAACACTTCAAGTAACCAAAAAAGCATGTCTTAATCCAGCAATATATAAGTACCCAAAATCTCCCATTCCAATACACACCAAAACAAGTCTCTTTTTTAATCTCTCTCTATCTCGAAACAAGCCCCGATCGAAAT

>AcSUS2 Chr21 Chr21:1613985..1615680

GATTGGGATTGTCTCCAGATTACATTTGGACTTAACTTTGCAGAAGGCCCAAACAAAACGATCCATTCAAAAAGTTGACTAAGACAATCCATTGCCCATGTGTAGTTTTAATATCCCGCCCAAACCCGATGGGTATGAGTTTTATTCGATTTTTCGTAAGATGGGAGAAAAACTCGATCGAGGATAGATCAGGTATGGTTTATTACCTCGATATCCCGAATACCCAAACCCGAACCCTACCTGAATAAACTTTGTTAAATTGATATTTTTTATAGTAATATATGATATATAAACAAGATTGAAAATTATTCAATGTGATGTATTTTAAAAAAAAAATTAAGTTAAATTTTGATATATGAATATATTAAATTTGTATTTTAAAGATTAAAATTTAATTTATATATTTTTTAAAAGATTCAGGTATCGAGTATACTCGATACCCGATTGAGTTTGAGTTTGAGTTTGAATTTTGTAGCCATATTCAATACCAATAAAATTGATACTATACCATCTAAATTTAAATTAATTTTACGAGTAAAATCTAAAAAAAAATGATAAATGAATACTTCAAATAAAAAATATGGCAACCAAATTTAAAAAATGCCACAATATAAGTTACCTTTATATTCTTTTTTGTACTTTTTGATGTCAATAATATTAATTATGCTAATGCTATAGTATTTCTAGAGAATTTTGTCATTTAAAAATATTTTTTTAATGTAAAATATTTTTTTAAATCAAATATATTTCAGCCATTGGATTAACTCAAATATATTTCATCCGTATAGATAGTAAGAGATAATTTTAGATTCAAAAAGAGTTTGTGTTTTATATTATAGATGTGTATCAAAGCTTTCCCTAACAACAAGGAAGAATGAAAATTAGTTCAAGAACAGAGGAGAAACTTAGGCTTTATGAAGTCAACCCAGTGGAAACAAATAAAATCCCACTCCAACATAGACGCAAGCTAGGACTGAATCATCTCACTCATCATAGTCTTTTTTAATGTTATACAGTGGAAAAGGCAATTTCCCAATGAGATATTCTTTTCTTTCTCCAATCACCCTTCTATATGAATATACCAAAATCAAAACCTAACCAAGCCAAGGCTTGTTTCACAAATTAATTGAGCTCAGCTAGTCAGCTATATGATTTTGTTATTGATATTTACTTTGTCGAAGGCAGGGACGGGCATAAGATTTTTATATTGGGAAAACGAATATACATACAAACTATAAAATTTGTAATATACGCATAAAATCTAATACTAGTTTTAATATTGGAGTTCCAAACTTATATAATTTAAAAATATTAAATTATCCAGCTTCGAAAAGAATGATAAGATAGAGAGATAGAGAGATATATGTTATCTTTGCTTCCAACCGGGAATCTCCTTGCTGTGACAAAATTCTTTTCTTTTGCTCTTTAATGGCTTTAGAGAATGTTTTTTCTCCTCTGTTACATCCTACTATACTTTCCTTGTGCCGTTTCCAATGCCAACAAAACAAGAACTCTCATAACCAAACACTTCAAGTAACCAAAAAGGCATATCTTAATCCGGCAATCTATTAACGAATCCATGAAGAAAACAACACACCATTGATCTATAAGTACCCAAACCCTCCCATTTCATTATACACCAAAACAACTTTCTTTTTTTAATCTCTCTGTATCTTGAAACTATTCCTGATCGAAA

>AcSUS3 Chr23 Chr23:20286097..20287792

GATGCTATTTTGATTGGAGTTGACTAGGTTCGTTGATCGAAAGAAAGAAGTGGGAGAGAGAAGAAGATTCTAGTAGTTGATTTGAGGAACGGGAAGAAAGCTAGTTGTTGTCTAGAGAAAGAGAGAGAGAGATATAAGATATTTATAGAGACACGCGGAAAATTAGTAGTGAGCGGTTTGATGAAGTTTTGCAGGTCCGAACCGAATCCCCACGGGAAGACAGACGGTCCATTGAAATATCGTGCCCCTAGTATTATGGTGTCCAATGTTTATCGTCCATCTCGAAAGCGACTTCCTCTTTTGGGAAGGATCACTTAAACCATACCGGCAAGCGGTTTGAAGTCTCGTAGGAGGACAAATCACTTACTGCACTGCCCATGGTATGATTGTTTCGTTTTGTTTTTCCATCATTTCGGTTTGCGCAATGGTTATCCATATTTATAATATAATATAAAACTAAGATATTTTTAAATTTTATAAATTATCTTTTATTTTTTACTATTGAAATTAATTTGAATTAATTTATTGGTTGAGATTTTACTTGAGACTATTTTTTTTAAAAAAAATATCACTTTCTTCATCTCAAATGTGTGGGGTCCACATATAGGCCAAAAAATTTCTAAAACTGAGCATATTTTTATTTATTAGATTATTTTTTGGTGAGACATAACCAACAAAACTAAAATACTTATTTTTTTTTATTTCTCCCAAGCTAGCAACACTTTTTAAAAATTATTATATATTTTTTAATTTATTCTCTATTTTGTAACAATGTATCTCTTCATATATGCATGCATTGTATCTCTTATCTGACGTTTATATTTTACCTGTTTTTTGTATTGAAAAACTCTAACGATTACACCCAAGATATATATCTTTTTTTACGTAGTGTCTATAAGCATAAACAACTACTAGTTTATATATATGTTTCACCGTAGGGCTACGTTATGTTTGATTTTTAAATGGATTAAAAATAAAATTATATATAATTATTATCGGAAGGCGTGTTGTAAGCAAGGAAAGTGGTTTGAAAGTGGTCAAATTAAAAAAGGGCATGAGTAGTGCATATTTACATAAATTCACAAAAATACGAATTTTATTATATGTGAGTAATGCACTGAACAAAATGCATTGTCTATAATAAAAACTCTTGTTCAAAATTAAAAAAAAAAATAGTGTTTTAGAAGCTCAACGGATAACTACTAATAAGCCTCACATCACACGAGCAAATGAATGGAAGTTGTCCACCTGCTTCAAGATGAATTGAATTATTATAGTTTGGGTAAATTACACTGACCTCTCCTGAGGTTCGGATTTAATACACAAAGTATTCCTGACGTTTAAAAAATTATAATGAAGGTTTAAAACTTGTAATAGATTCCCTTATTTTACACTGACCACCCCTGAAGTTATGAGTTAATACACAAAATACTCCTCACAAAGTTACTTCTTTTTGTTTTTTTTACAAAGGATGTTGATTTGACATCAGGAGTGTGTTGTGTATTAATCCCGAACCTCAGGGGTGGTCATTATAATTTTTTAAACGTCAGGGGTATTTTGTGTATTAACTCCGAATCTCAGGAGAAGTCAGTGTATTTCACCCTTATAGTTTTTAGTAATTTTTTTTTATCTCCAAGTTAATAAAAAATTATTATTTTACCATTCATCTTAATTAAAGAGACCGAAAAAGTTTAAA

>AcSUS4 Chr12 Chr12:12788687..12790382

TTCAAGCACTCTCTCAGCAGTGTCACCCCACCCTCTCTCCAAACCGATCTCTTGGAACTTGTGCTCAAACTCGGAATACGGTGTCTCTAGAGGGAGTGTAAGGAGATATTCCTCTGCCTTCCTCAGCACAAATTGGAGGGCATCGAGGTTTTGTATTCTGTCATTCAGCATCATTGTCTGCACAAAGAAAATAAAAAATTATACCACAATCCTAACCCAAATCTAAATCAAATGGAGTAATCCACAAAGATTAATTACCTTTCCCTTGTAGTTGTGGACTTTAAGAAAATCAAGCAGAGGGTACATGCTTTCCTTGTCGTGGAACATTTTAGCAGAGAGGTGTCTGTTAAGGAACTCAACTCCATTCCCAATTGACTTTGAAAGAGTTGGCCGAGGAAATGATGCGGTGAAGGGCTCAAAATCCAACTCAAGAACAAAATTTCCATTGCACCTGCACCCACAACAAGCAACCAACATGATCCATAAATATGCTAACAGCTTCCAAGTAACAACTAGCAGTTGCAGAGTTACTTACGGTCCATCGACAAGTTCTTCTTTGAACTGTAGATACTCGGGAACACTCAATTCCTCAACAACATGGGCATTGAGATTAACCCTCATGTATTCCCACACACCAGGCCTCAGCCGAACCGCAAAAGCAACCCATGGAGGCAAAACGATTGCTTCCTGTCATACCCAAAATTAACAGATCACAAGATTTAATCATCCAGAAGAGAAATTAAATTGCACAATGTTTTTTGATTATTTACCTGTGCAGATTTGAGAAGCTCTCCAAATGCTCCGTCATACAGTTTCCGTTGGACCTCTTTGGAGAGTGCTTCAATCTCAGCCTCGATCTGGTGTGGTTTCAGAATTCCTTTGCCATGGCTTTCAATCCTACAATAATATCATAAATAAAAAAAACCAAACCCATATGTCAGACCACTCAAATGGGAGTGTTCTTTCACTGAAGTGATCTGATTATGCAATTAATACTTCATACTTGGAGAGAAGCAGCAGTATTTCATTGCGCTGAGCAGACAGAGTTCCATCAAGACGCTCGCGAAGGCTGTGAACACGAGTCATGACTTGTCCTGCCATGTCTTCAGGTATTCACACCCATCAAACAACACCTGCTATTACTAACAAAACAAACAAATACTAGTTTATATAATAAATTTGAAGAATAAATATGGATATACTGCATTATCCAAAATCTATTGCTTAAAAAAGACGGGACTGACCACAAAAGGTCAATGATCTACCTCATTTGGGATGATCTAGAATGCGATTCGTCTAAAAAATTTAAAAACCAACCAATACACTCAACGGCACCCACTTATTGACTTTTATTAGGCAAGGACGATGAATGCTCGACAACCAACGCTTTTGACAACTGGGTTCATCAATACTCTCTCTCTTATGTCCTCAAAAATCTTAGATTTTTTTGCAACATCCAACAACTTCAAACCCCAAGTGTTTTTTTATGTGAAGAACCCATCCCGAATACTCTAATCACTACCATACTGCAATCTATTAGAAAAATACAAACATGATCTGCGTGAAACAGAGCATCATGTCAAAGAAACCCTCTTTTTCTAAGAAAAAAAATTGAAAAACAGAGCAAGAGAAAAATAAAAATAAAATAGGATCCGTCAAAAGAAAAACATGCCAAAACAACTAGACTAGACTTACA

>AcSUS5 Chr00 Chr00:61505985..61507680

TTCAAGCACTCTCTCAGCAGTGTCACCCCACCCTCTCTCCAAACCGATCTCTTGGAACTTGTGCTCAAACTCGGAATAGGGTGTCTCTAGAGGGAGTGTAAGGAGATATTCCTCAGCCTTCCTCAGCACAAATTGGAGAGCATTGAGGTTTTGTATTCTGTCATTCAGCATCATTGTCTGCACAAAGAACATAAAAGATTATTCCACAATCCTAAACCAAAATCTAGATCAAATGGAGTAGTAATTAATAAAGGTTAATTACCTTGCCATTGTAGTTGTGGACTTTAAGAAAATCAAGGAGAGGGTGCATGCTTTCCTTGTCATGGAACATTTTAGCAGAGAGGTGTCTGTTAAGGAACTCAACTCCATTCCCAATTGATTTTGAAAGAGTTGGCCGAGGAAATGATGCAGTGAAGGGCTCAAAATCCAACTCAAGAATAAAGTTTCCATTGCACCTGCACCCACAAGAAGAAGAAACCAACATCATCAATACATATGCTAACAGCTTCCAAGTAACAACGAACAGTTGCAGAGTTAGTACTTACGGTCCATCCACGAGTTCTTCCTTGAACTGCAGATACTCGGGAACACTCAATTCTTCAACAACAAGGGCATTGAGATTAACCCTCATGTATTCCCACACACCGGGCCTCAGCCGAACCGCAAAAGCAATCCATGGAGGCAAAACGATTGCTTCCTGTCATACCCAAAATTAATAGATCACATGATCATCCAGAAGAGAAATTAAATTGCACAATCTTTTTTGATGATTTACCTGTGCTGATTTGAGAAGCTCTCCAAATGCTCCATCATACAGTTTCTGTTGGACCTCTTTGGAGAGTGCTTCGATCTCAGCCTCAATCTGATGGGGTTTCAGAATTCCTTTGCCATGGCTTTCAATCCTACAATAATATCATAAAAAAACCAAACCCATGTGTCAGACAACTCAAATGGGAGTGTTCTTTCACTGAAGTGATCCGATTATGCAATTAATACTACATACTTGGAGAGAAACAGCAATATTTCATTGCGATGAGCAGACAGAGTTCCATCAAGACGCTCGCGAAGGCTGTGAACACGAGTCAGGACTTGTCCTGCCATGTCTTCCGGGGTTCACACACAACAAACAACACCTGTTATTACAAACAAAACAAATACTAGTTTATACAAGTTTGAAGAATAAATATGGATATACTGCATTATCCAAAATCTACAGCTTAAAAAAAGACGGAACTGACCACAAAAGGTCAATGATCCACCTAATTTGGGATGATCTAGAATGGGATGCGTCTAAAAAAATTCAAAACCGACCAACACACGCAACAGCACCCACTTATTGACATTTATTAGGTCCCCAATGGTACGCTAGGACGATGAATGCTTGACAACCAACGCTTTTGACAACTGGGTTCATCAATACTCTCTCTCTTATGTCCTAAAAAATCCCAGATTGTTTTGCAACATCCAACAACTTCAAACCCAAGTGTGTGTTTTTTGTCTTTTTTTTTTATATGAAGAACACATCCCGTATACTCAAATAACTACTACTGCGACCTATTAAAAAAATCTCTAATTATACTTCGTGAAACAGAGCGTGTCAAAGAATCCTTTTTTCTAAGAAAACAACACTGAGAACGGAATATAATAGAAAAACAAAGTAAGAAGAGAAGAAAAAAAAAAGATCATCCAAACGAAA

>AcSUS6 Chr00 Chr00:84916729..84918424

CCGGCTAATCCGGTAGCCAAACAGCCCCTAAAGCAAAAGCTAGGATCAAATGGAGATGTTTTTCTTTCCCATGAATATATTCTAGGGAATTTGAAAGGACAAAAAAGTTGAGAATTATGGATCACGGCATAAATACGAACCTTCAAAAGAGTAAAACCTTAAAACTAATAACATACAAGTCCTCATGGATACAATCAAAACTTCAACACTATGGGAATTATGTTTGTTTTGTTTTATGTTTTGTAAAATAAAAATTAAAAATATATTTGTGTTTACCTATTTTTAAATTATTTAATAATAAAAAATATATTTGTGTTTATCTTTTTTATACTATTTTAAAAAAAAAGTGGCGGTAGAGGAGGAGGTGGTGGTGGTTGAAAAAATTTGGATGAAAAAAATTTATAAACAAATTTTGTAAACAAAACAAAAAACAAATTTTTCTTATTTTAACTATTTTCAAAAATAGTAAAAATAGTTTTGCCTTATTTTTATTTATTTTTTGCAATACAAACAAATTTTTCGTTTTTATTAATAAATTAAATAAAATAAAAAAAAATATAAATTAAACAGTTAACAAACATCACATATATATGGAGTGTTGGAATATTATTCTTTTTATACTACATATTTCCTTCGGGCTTATAAAAGTACCCACCAATAGACATACATAAAAAAAAAATAACAACAGAAAATTTATGTAATTCTCCTCAAAATGCCTACATATCCACGAGAGAGATTTTATTATGTATATAAAATAAATATTACAATTGTGAGATGAACAAAATACAAAAAAATACACTCCTACTCGAGCTCCATACTCAAAGGTATTTAAGTCATTACTTATCTTAGTGTATCATAGTATTTTTTCTCTTACTCTTTACATCTTTATTTTACCTTCGAGTAGGAACTTCTTGGCCCGAAGACCCTAAACTCACCCGCACCCGCACCCGCTACCTCTTTTATCTCTTCTATTCTCCCCTTCTCTTTCTAAACTCTATCCACTCTTAGGCCAGTTATAGAGAATGTCCACCACCTTAAAAGCATTTATTCAGAAGTCAAAGCATCTTGTTTGTCATTCAAGTTTAGGGTTAAGACCGACTATTTATTAGTATGAATCATTCAACACTCGCTACATTTATTTTATGAGAAATCTTAAGGTCTCCAAAAAAAGTCTTATAATTCAACTTGATTAAAATTTATTTGAGTATGTAAAATTTAATGTAATTAAATGAGTTTAAATTAAATATTTTAAATTATAAAAAAATTTCTAATCAGTACTAGTAAATCGTCCATATAAAAATGTTAACATCAAAAGGAGTGCGTGTGCAGCTGCGCTCCTTCCCAAAAGGAGAAGTCGCTTTCGAGATGGCAGAGATAAAACAAGAAGCGAGATTGGACACCATACTACTTGGTTCACTGCATTGCAGTGAGCCATTTTTTTTTTTTATTAACCACGTAGTGTCTTAGATAGGTCATCTTTATTCCCGTTGGACCAAAATATTCCGATCTTCAAAACTTGATCAAACCGCTCACGGCTAATTTTCCGCGTCGAATAACTCTCTCTCTCTCTCTCGACAGCAAACATCTTGCTTCCTCTTCCTCAGATCGACTACTAGAAAATTCTTCTCTCTCCCTCTTATTTCTTTCGATCAACGAGAGATCTCGAGCCTAGTCACCTCCAATCAAAATCGCATCA

>AeSPS1 Chr13 Chr13:6061575..6063270

CATCACCAGTCCCTATAGAGAGAGATTTCTGGTTCGCAGGTTGGGGATTCGGTGATACGACGAGGACAGTGACTGTATCGAGAGAGAGATGATTCTGGTGGAGGGGGTCGGTGATGCGATGAGGACGGTAGTGACTGTAAGTTTGCCAGAGAGAGAGAGAGAGAGATTGCGGGATGTACAGAACGACAAAGTGCTGATATATAGAGGCGGCGAGTTTTTGTGATGTTTTAAACGGTAAAGATATTTTGGGGAAATTAGAAAAATAATTTCATGGTTTAACTTCCATAACAGTATATAATTTTAATTAAAATAAAGTAAACTTTAAAATTTTGATTCGATTATAATTAAAATTCTTCGTTTATTAGAATTGGAATAGTACAATAATTTTTATAAAAAAATTAAAAAATATTTTTTTTTAAATATCATCATTTAAAGACCTTAAAATCGATCAATTATGATATATTATCAAGTTAAAATCAATGCATAAATTCATTTGAATTAAATATCAAATTTTTCTCAAAATTTTATATTTAAAAATGAGAGCCGGGGGTGTGAGTTTTTTAATTCGGTGAACTTGTAATGATTGAAGGATATTATGAGATTTTCGAATAATACGTGAATCAAAAGTTTTAGGGGACGCTCGAAATGCAACCATGTCCTAAGTTCACCTCAACCCAAACTAGGGCTTCCTATTTTATCTCAGTACAGCCCACAGTGTTTTTCAATGGTAATTTGAGACGGATGAAATTGTAATAAAACTATTTTTGATATTTGGGGTGTCCTCAGATAGGTTTGGTTTTCATTTAGAAGCTCCCAAAGTTTACGGTTTCCCACGCATTTACTATGTCAAAATTGCCCCTTACATTTTGCCCTCTCTTAGGGGCATTATTGGTATTGTGGAATTTAATATCCCAATGCCTTTTTTTTTTTTTAAGCATAATTATACATATACTATATCTTTTTTAGTTGTACTAGACTGTGCAAAATATCCGACAACCCGTCTAACCCGATCGATCCGACCTGACCTGACCCTAACCCGATCAAACTCGATCACTCTACGGATGGAATTTAACTAACCCGAATATATTCGGTTCGGGTGTCGAGCTATTAAATCGTCCAATCCGATAAAATATTACACTAAAATTCATAATTTTTATCCAATCCGACCCCATCTCTACCATTTATTAGATCGGATTCGGGCCCAAATTTTTTCAACCCGACCAGTTCGATTCAGGTGTCAGGTGACCACAAACCCGACTCAACCCGTGAACAGAATTTGTACACACAACTGTACATTTTCTTTCATTTACAATATACGAGCCCCACACATAGGCTCCGTTAGGTAATGCTTAAAATCCAAGCTTTTTTTAAACTTGTTATCAAAAAGGGCAAGATTTTTGGACTTTTTAGATTTTGTGGCTTTATTTTAGTTGAAATTAAGTAAAAAAATAAACTCTAATTAATTTTTTTATTTTTTAACTTTAAAAAAAAAATTAAAATAAAGAAAAAAACTGGTGCCAAACCGAGCCATAATATGCGAGTCCACACAACTCATGTATAAATAATATCTGCCTTAATAGCATTTTCCTAATAGTATTTTAGTAGTTGTTTGGTTAGAAAGTGCTTACCCAATGAATTGACAATCTTTATTTTTTTAATAAGCTCGCCACACTCTACGTGGACAACTGTGAGATTC

>AeSPS2 Chr06 Chr06:7915205..7916900

TAATTTTTATTTAATAAATTAGGCAAAGAGTGAACAGAAAATTGACTCTACTTGATCTCTATGGGAAAAATTTGAAAGGAAATCTCAGTAGAGTAGGGTCTAATACGTGGGTTGGGTAAAACAAGATTTTATTAGATTTGAATCAATTATGTGAGAGGGATAATTCAAGTAGATCCACTGTAGTATGTGGGTAGCAAGCCACCAATACATGACATAGGGCCCGTTTGGTAGTGCTTAAAAGCCAAGTTTTTTTTAAGCTTGCTACCAAAAAGGCTAAGAATTTTTGATTTTTTTGCTTTTGGGTTTATAGTTTACCTAACTAAGCAAAAAGCCAAACAGACAAAGCAAAAAAGCCAACTCTGATTGGCTTTTTTGCTTATTTTAAGCCAAAAAGCCAGGAAAAAAGCTGGTGCCAAACGGGTCCATAATGTCATCTCTAGATAGGGTCGGTCCTGATATTTAGGTTGCCCTAATTCGATCTCAATATGTGAGAGAGTTGCTTGCCACCCAGATAAAAAAGAGACGGAGATGACTTTCTGAATTCAAATTTTTTTGGAGGGCTTGATAGTTGTCCTGCAACCCCTAACCCAATGACTTGATGGGCTTACCACATGGCCGACCCTGTCTCTAGATAGAGTGATTAAGAGCGAGCATCGTGTTGTGTTCGTGTTATATTTCCTTCAATGCTAATCTGATCTATTTATTTGTTCGTGTTATCGTATCGTGTCATTTCATATTCGTGTTGTATTAATAGGCCATATCTGAAAATTTCAATCTTAATTAAAAAATTTAAGTATTATCAAATAAATCTCCATGGATGATAATACTCAAAAAGTATTTTTATCGACTCAATTATTTTTATTTTCATTTGAAAAGAGTGAGCGGGCTGTCAAATATTATGTTTCTATATTATATGTACTCAAAGTTTGTGAACAAAACGAATAAAATTACCTTAAATGAACAATAAAAATGTCTTTATTGAATATTAACACATAATTTCTTTTTACATTCTTTTATTAAATTGGACCATTGGATCATGTATTATCATGGATTGAACTATATTATGTCACCTCTAAGTAAAAATATAGCTAAAAATGTATAATTAATTATTCACTAACTGTGTGTCATATCTAGCTGTAAGGATTTGATACTAATGACTCTATTTATTTTTTATATTATCGAACTGTATCATATTCGTTTTATGTGAAAAATGACTAATCATAACCTACATTTTATGCACGATTCGAGTCGTGTTAACAGATTGGCTCTGGTAGTGAGCACTTCTCTAATTATTAACATATTTTGAATTATTTTAAGCAAATTTAAGCTTCTTTTGGTGAGGAACACCCTTGGTATGGTGTCCCTGCACCACACTCAATAGCCTCTCCTTCAGAACCGTCCCCGCTATAACAGATGATCCCACATAGACCCGTTAAAACTCTCAATCTGTTTATCACACGTGTCACCTCTCTGGCCCCAGTTTCAACTGCCATGTGGTCCTCCCTCCCTCATCCCCACCCCCACCCCCCGCCCTCTCTCTCCTCCCCTGCTCTCTCTCACAACACAAACACTCCCTCTCCTACCAAAACTCTCTCACAACACCCCAATTGAGCATTACCACTTCACATTCAGCACGAACCCATAATTTTCCCTCCCATTTTCTTGCGTATTTTCTCGGGAAAATCCGGGAATGGCC

>AeSPS3 Chr10 Chr10:13629821..13631516

CATCTCTCTCCCCGTTTTCGTTTCCAAAAATAACTTCACAAATTAAAACAATAAGATGTATACATAAAATGAATAAAACAATATTGTTGTAGAAGGTGCAACTAGAAGAGAGAGAAGGTAGAGTGAGAGTTTGGAGAGAGGAGAAGGTGGTTATGGTTAGGAGGGATTTGGGTGGGAACCAATTGGAAGAGGCGAAGATAGCCAGCTTTAAAGGCTAGTTTTTGAGTTTACAACAAATTAATTAATTACTCAAAATATGGAGGGGGTGCGTGGTGCCTTTGGTTCCCTTGCTTTCCTTTACGAGGGTATATTAGCCAGACATCCTTATTCTTTTATTATTCTCCACATACAATGCTACCCTTCTCTCTTCTCTCTCTCTCTCTCGTCAAAACATGCCTAGCGAATTATATGGTGAAATTAATCTAGGGCAAAATATAGACAATGTTCCTCTAGTTTCACAAAAATCTCAATTTCGTCCCCTCTAGTAGCAATTGCAACAATTTTAACCCAATTGATAATGGTTGTTAAGAATTTTGATGGAAATAATGCACGAGTAGAGGCATTGCATTTTGCAAGGGAAAAATTGTCACTTAAATGTTCTATCTAGCCAAATTTCTATGTGCAAACAATTTTAGTTTTCTCATCACAAACCTTTTCGGTGTAGCTCATTCAAAATTTTTAATTTTGGTTTTTACTTAATCCACCGCTCTAAATCTCAAACCCTAATACTAATCCACCTTCTTCTACTACTACCACCACCACCACCACCACCACCACCTCCTGTCACCTTCTCTAGCCCCCCTAGCACCTGACCTCTAAACCCTCTCCTTCCCAATGACAAGCAAACCCTACCATCCTCTCCTCAACGCTACCCTGAGCCGGCCCTGTGCATAAGCCTAGGAAGCAACCGCTTTTGCCCCTGATTTTTTGTTCAAAATTAGGGCCCCCGTTCAGGTACTACCCTTATGCTTTACCCAAAAAAATATTAGGCCTCAATCAAATGAAGGGCCCAAAATAGTTTGAATTTTCAACTTTGAAAAAAATTACAACTAGATCTAGCTTTTTTTAAAAATATTCTGCCTAAATCTACCATAATATCAAGTCTATAAGGAAATCATTTTAATTAGTGGAGAGCTTGAAGTAATTGATTGTAATCTTCCAAACTTTCACATAGATCTAGCTCAGAAAATCACGTTCAAACATAAAGCTAATTTTATTAATGGAGAAACTAAATTGATAGATAATTATTTTTATAATACAGAATTTTTTTAAAGAATATTATTGAATATAGTAATAGTAATAGAGCCGCATTTGGAAAAAATAAGTTTTAAGTTAACTTTATCTTTTCTTAAATATATAAATGCCCCATATTAAAGTCTTGCTTGGGGCTCCATAATTATGGTTCCTTACGCTAATATCCCCTCTTAGGACCCCATAATTATCATGACCGGCGGCCCTGACGCTACATATCCCCCCTCACCCTCCAACTCATTAAAAACCAACCGCTCTTTCATGGGATAGGTGAGTGTTTGTGGTGGTACCTGGTGGTGCTGGTGGCTGATTGTCGGTGATGGGGAAGCTGGAAAATTGTTTAATGCATCTATTAAATTTCTATTCACAAATCTTTCTTTGAGTTGGTATAATAGTTTTAAGCCTGAGGTACAACATGTAGTATGATTGTGGAAGTGAAAAAAAT

>AeSUS1 Chr20 Chr20:21238231..21239926

ATTACCTTTTTTTTTCCTTTCCAAAAAGCAGACCAATGAAATAACAGAGAGGAATGAATGAGTGAATGAAATCAAAAGAAAATAAATGGACGGAAACAGAGGGGGAACAGAAAGCAGTGAACGATATTGTTATGCTTTGGGATGATGGAAAGGGGAGCCTTGGATCCCATTTTATATGCCCGTCTGCTACTTATGTTTTGGTGGAACACCAATTTCTCTCTCTCTCTCTCTCACACACACCTTGTATTCACAAAACCGCTTTTCTTGTTCATCCCCTGTATTCTCTTTTCACCCACTGTTATACTCTCGTCTTTATGGATCCCCTTTTTCCTTTTGTTGATACTCTCCCCGTGGAATTGTACATTTATCCCATGGAGCCCCATTTCATTATGTCTTTTACAAAAATTATGTTGGATTTACTCCAAATTCATTCCTCATTGTTTTTGACTTCAAACTCTTTGTTGTATTTTGATTCAAACTCTTAATTGAATTTAGTTTTAAATATAAAAATACTTTTAGCGAAAGTCTCAAATACAAAATAATAGAATAAGAAAAAAGATGTAAAGTGAGTATGAGAGATGTAGTTGCTCCTCTGCCACTACCAGAGGAGAAAGTAAGGTACAATATTATGTTTATAAATATTTCTCTACATGAATAATGATTTTACTGCACCACTGCGTAAACAATTTTGCATATATGTGTTTTTGATAACGTGTGGTTGTTGATACGATTTTGAGTACAACAAATTAAACTTTGTCCTTGTAGTTTATTGAGATTGTTCATACCAATCATAGAAAATTCTGAATTTTATTATAAGGTGGTATTCTTAATTTATTTCTATGCAATTATTAATCCTATTAAAATTGAGAATTAAATATATGTTATTAACTATGAGTCTACGCGTGCGCGTGTGTGTATATCTTACATGGATTTAGGAATATGGTTCATTGTGGGCAAGGCAGGACGAGGAGTGGGTGGAGTGGTGGGTCAAAGATGTTGAAAAAAAGGCAATACGAGACGAATACAGTCGTAGTAGTAATGGATTTTTTCTATCTCAGCAGTGGTTTCTGTTATCGTTGACTTGCCCCACACATGGTTTTGCTTTTTTTAAAAAATAATAATAATAATAATAATCGAGGGCTCTTATTGTTTTCTTCCTTGGGTTGGGCAGTTGGGGTCTCATCCAGTGGGAGAAGCGAAAGGAACTCCCAAAAAACAAAACAATGGCACCGACGATCACGGAATGAATGGAGAATTATTGATGACCCGACACTATAGCGATTAGCTAGCTAGCTAATCTAATCTTTCAGATCTGTTATTTGTTTTGATTTTTAATTTTTTTTAAAATCTTTACATTTTTAGCCAATTTGAGCCTTTTTTTTTCATTGGATCTCCATTTTATAAGTTATTTGGGATCATGGTGTACTTTTTTTGCTAATGCATTTATTTGTTTTGATAAATGTTGTGACAAATTCGACCTACCATTTTGACTTTGCTAAAATTTTCATATACGAAGAACATCCACGTAGGCTAGTTATTTTAGCATTTTAAGTAAATTGGCTAAAAAAACTTCAAAAAAAAATAATAATTTACTACATCAACTTAGTTAAAACTCTACATGAGTTTAACATGGCTACGATTCACAATCGGCTCACCATTTTATTATTTTTTTCAAAGAAAATGGTGTTGAAGTTTG

>AeSUS2 Chr12:407491..409186

TTTGTTTCCTTTTCAAAAAGCAGACCAATGAAATAACAGAGATCAATGAATGAGTGAAGGAAATCAAAAAGGTAGAGAGAAATTGAATGGACGGAAACAGAGGGGGAGAGAGAGAGAGAGAGAGAAAGCAGTGAACGAGATTGTTATGCTTTCGGATGGTGGAAAGGGTGAGCCTTGGTTACCATTTTATATGCCCCTAGCAGGCCCCTCTGGTACTTATGTTTTGGTGGAACACCAATCTCTCTCTCTCTCTCTCTCTCTCTCTCACCTTTCTTGTTCATCAGAATCATCACCTGTATTCTCTTTTCACCCACTGTATATTATACTCTAATCTATTACACAAACAATTATTCTGTTGATCCACTTTATCGGTTGATAACAGATTGAAAAATTAACCTTAAGTTGTGAATTTGCCAATTAATTAATTTATTTACAATAAATCTACGAATCTAACCACAATAAACATAGTATAAATAAATAAATAATAGACACAAAATATACGTAAAAAACATGTTTATCTTTAAAATATGTTATTAAACAAGGACTCTCTCTACCCTTGTGTATACTGATTATTCGTCTATCTTTTACGTTCATTTTGGAACGGTTCAATGTTGGTGGGGGTCAGGCAGGACTAGAAGTGTGTTGTTTGTCAAAGATGTTGAAAAATGCAAATACTAGACGAATAGAGTAGTCGTAGTAGTAGTAGTAGTAGTAAGGTATCCATAGCCATGGTTTTGCACTTTTCTCTCGTCTTATTTTCTTCTTTGGGCGGGCCACCGTTGTTGAGATCGCATCCAGGAGTCGTTTACGGCTTCATCCTGTTTAACAGGGATGAAGCCGTAATTAATTTACGTAAAAATGCGTCCCAAAAACCTAAAGAATTGGAAAAAAATTGTTATTATTCCCCTTGCACGACAATTACGGGAAATGAATGGAGAATGATTCATGACAAGAAGAGCGAGTCACCATACTTGTTCATTGGATTTCTTTTTAAAAATTATTTGTAAATTGTCATGTACTGTTTGCTAATGCACCGCACCCTATTTCTTTGTTTTGCCAAATTTGACATAGGATTCAACTTTACGCAAACTTTTAGAGCATCCTAATTGCTTGCTAAATGTTGTGCTAAATTTGACATACGATTTGACTTTGCCAAATTCTTGGAAAACTTTTACAACATGAGTTGCTAAACATCCTCCTCAGATAGTTTAGGGAGGAAAAATTCACTCGAACAACAGGCTTTCTATTTTGGGCTCCTTTCTTGGCTCGAGGCTAGTTATTCTAACATTTTAGGTAAAATTAGCTAGTAAAACTTTTAAAAAAGGATATACTACAGCAATTTAATTAAAACTCTCTACGATTCACTAATATATTATTTTTTTAAAAAAAAAAGAACGTGTGTAAGTGTGCTGGAGGTTGTGAAAAAAAAAATATGAAAATAATAAAGAAAATGTTATTGATCTGTAATAGGATAAAATACACAACTACCAATTCTGGCCGAGCTAGGCTAGGCACCACTATCAAGCTATCGCTATGAAGTAAACTCAGTGAATACTATGTATCGGAGCTCAGTCGAGACATTATCGGACATTAACTCTGTCTGAACTAAGGCAAGCACCGCTGTCGAGCTGTCGCTCTATAGCAAGCTCGGCAGGGTTGCTGCACCGAAGCTTGATCGAGAGGCAATGGTCGACGG

>AeSUS3 Chr21 Chr21:15291335..15293030

TAAATAGTTAAAACAGTTTAAGACAAAAAAAACTCAAAACTAAAGTAGTTAAAATTTTTTTATAAAAAAACTAGAAAAACGATCCAGTCAGAAAGTTGACTAAAGACAATCCTTTGCCCGCGTGTAGTTGTAATATCCCGCCCAAACCCGGTGGGTATGAGTTTTATTCGATTTTTTGTCCGATAGAAATGAATATGGTAAGGTGGAAAAAAACCCAATCGGGATAGATCGGGTATGGTTTATTACCTTGATATCTCAAATACCCAAACCCAAACCCTACCTGAATAAGCTTTGTTAAATTGATATTTTTATAGTAATATATGATATATAATCGAGATTGAGAATTATTCAATATGTTATGTGTTTAAAAGATTTTTTAAGTTAGATTTTGATATATGAATATGTTAAATTTGTATTTTAAAAAATTAAAATTTAATTATATAGGTTATAAAGATTCAGGTATTGAGTATACTTGATACCCGATGAGTTTAAGTGAATTTGTAGACCATTCAATTACCAATAAAATTGATATTATACCATCTAAATTTAAATTAATTTTACGAGTAAAATCTAAAAAAAAAAAGGTAAATGAATACTTCAAATAAAAAATATGCAACCAAATTTAAAAAATGCCATAATATAAGTTGCCTTTATATTCTTTTTTGTACTTTTGAATGTCAATAATATTAATTATGCTAATGCTATAGTATTGCTAGAGCAATTTTTTCATTTAAAAATATTTTTTTAACGTAAAATATTTTTTTAAATCAAATATATTTTAGCCATTGGATTAACTCAAATATATAATTTTAGATTCAAAAAGAGTTTGGGTTTTATGAAAAAGTGATTTCTGGTTGTTGCTGGTAAGCCAGTTTCTGAAGCATTGAAGTATGAAAATTAGTTCAAGAACAGAGGAGAAACTTAGGCTTTATGAAGTCAACCCAGTGGAAACAAATAAATCCCACTCCAACATAGACGCAAGCTAAGACTGAATCATCTCACTCATCAGTCTTTGTAATGTTATACAGTGGAAAAGGCAATTTCCCAATGAGATATTCTTTTCTTTCTCCAATCACCCTTCTATATGAATATACCAAAATCAAAACCTAACCAAGCTAAGGCTTGTTTCACAAATTAATTGAGCTCAGCTATATGATTTTGTTATTGATATTTACTTTGTCGAAGGCAGGGACGGGCATAAGATTTTTATATTGGGAAGACGAATGTACATACAAACTATAAAATTTGTAATATACGCATGAAATCTAATACTAGTTTTAATATTGGGGTTTCAAACTTATATAATTTAAAAATATTAAATTATCCAGCTTCGAAAAGAATGATAAGATAGAGAGACATATGTTATCTTTGCTACCAACCGGGAATCTCCTTGCTGTGACAAAATTCTTTTCTTTTGCTCTTTAATGGCTTTAGAGAATGTTTTTTCTCTTCTTGTTACATCCTACTATACTTTCCTTGTGCCGTTTCCAATTCCAACAAAACAAGAACTCTCATAACCAAACACTTCAAGTAACCAAAAAGGCATATCTTAATCCAGCAATCTATTAACGAATCCATGAAGAAAACAACACACCATTGATCTATAAGTACCCAAACCCTCCCATTTCAATACACACCAAAACAACTTTCTTTTTTTAATCTCTCTGTATCTTGAAACTATCCCTGATCGAAATGG

>AeSUS4 Chr05 Chr05:2858833..2860528

CAAACTGCCTTCTTCTTCAACTTTCCACAAACCTAACCCTAACCCTAACCCTAACCTTAGGCCACCTCAGCCGCCGCTACCTCCAAATCCCCTTCGATATGATGTCGTGAGAGTTGTTCCGTCCAAGCGAAGCATGAATCTTCTGAATCTATCTGATCGTCGATTACTATAAAAACCATGAAATTCGAATCGATCAATGTTTGTGTAAATTAGGTAACGTTTCTTCGAACTTGTCTTTCTAGAAGGTTTACAGAATTTCGATTTTATACGCGTAAATTATGTATATCAAATGAAGGCCGTCTCGTGTTTTTGATCTTTGCAAGTGTTTATTTATTTATATTCTTGCTATTAGTAGCTGTTGAACGTGATAATTTATGCCTCTTTAAGAGGGCTTGTGTTGTTGAATTGAGGATTAGGGTATTCTGGTGGTAACAATGGTGAGTGATGGGTGGTCGGCGAAGGAGGGGAGGGGCTTAGGTGATTGGATAATGTGGATGAGGTGCTGTGGGAGGCGGGGAGATTGGGGGAGGGAAGACGCTGGCTGCGGGGGTTGGAGAAGGGCTGGTGATGGCGGGAGAAACATGGTGGAGGTGCTGGTAGTGTTGGAGGTTCGCTGGAGGTGTGGAAATTGGGGTCTGGAAAGGACTTAAATTTGACCGTCCAGAACGGTCATTTCATACTGGAAAAAAAATGTAATAAAAAAATTATACTTTTGGAGTATTCCGAGACCATTGGTTTGCATTTAGAAGCTCCCAGTCAATTTTTTTTTTTTTATTAATCACATAGTGTTTCAGGCGATATGCAGACGGTTTCACGCCCCCAGACGGTTGTAGTGAATTTTACACCCAGACTAATCTCTGTATTCCTGAGCAGCTAGACGAACAAAATTGTTCGAGCTTAATTCTTGTTGGACTCATTAAAACTTTGTTTGAGATTGGTATGTTACTTAAACAAATCAAATTTGAACATATTTTTAAAAACTTGTTACAAATCAAGTTTGAACAATTTAATATTAGGTTTGGTAAGATTATAATGTGTAAAAAATTCAAACTACTCGAGTTCGATTTTTGATCGGCTTGATTAAAACTCTTTTGAGCTCATATTTTTACAAACAAGCCAAACTCAAACCTATTTTTAAAGCTAATTAAATTTTCAAACCAAACTCGAACACACCACCAATTGCTTGTTTGACCGGTTATCAACCCTACACATTCGTTTAGCATTTCCATAATCAATAATCTTATCAGTTGTCTTTTATCAACGGCCCTACATAGTCTAGATTATATGTGAGCAAACTATCCTAATCTATTTTCTCTCAATGTATATCCCATTGTTCCTACCCCTAGCAGTTCCAATTGGTGAATTTGAATAGAGAGAGAGAGAGAGAGAGAGAGAGAGAGATTACCTTTGTTACGCACCACGAATCTCCTTTCTGTGACAAAATTCTTTTCTTTCGCTCTTTCCTGGATTTAGAGAATGTTCTTTCTCCTCTTGCTACATCCTACTTTCCTTTTCTTGTGCCATTACCAATTTCAAAATAACAAGAACTCTCATAACCAAACACTTCAAGTAACCAAAAAAGCATGTCTTAATCCAGCAATATATAAGTACCCAAAATCTCCCATTCCAATACACACCAAAACAAGTCTCTTTTTTAATCTCTCTCTATCTCGAAACAAGCCCCGATCCGAATGGC

>AeSUS5 Chr26 Chr26:17940479..17942174

GCCATTCTGATGTTAAAATGTCGTTGCTTGTGAATTCAAAAGTGAGGTGATTTTAAATTTGTGAGAATACGTATATATAGGGTGGGGATTTAATGCGGGGGACTAGAAGAAGAAGAGAGAGAACATTCCTTGGTGCGGAGTGTGGGAAAGGACGACATGAAGAAAGAAAGGAATTTACCATCGCGAAAAGGAAATCTCGCATGGATTGAATGGAAGTGTGGAGGGAAAGTATTCCTTGTTTATATGTCCTTCGAGATGGGTGAACTTTTTGTTCTTTTCTTCCAAACCAAACTTCCATAACAGGCAGCTGCATGCATGTAACCCAAAAACCAACAGTACAGGACTTGTAGAGGAATTTATAGAAGGGAAAAAAGGGGGAAAAGAAAAGGATAATAAATTTGGAAAGTGAATGTATCTTTTGAGGGTTTTTTTGTCATTTTTTAGTTACTTGGTAAACCAATCTATAAATTCATATTCCAAACACAAACGTACCTAAATGATTACATGTTACATAATAATAACCAGTTACATGTCAAGTAACGTTCATTTTCTACTCTTTTTTGTTTAATTTTTTGGCAAATTTGTAAATTCCTATGGATATAAAAACTTTAGGGGTTCGTTGTATCCTTGGAGTGTACAAGGTTTCTGCACCCTTTTTTTCGGCTCTAGATTTACATAAATTTTCATGTAAATTTTCGTATAACTTTCATATTATGTATAAAATGCTCTTTTTCAACTGATAAACACCACTTCAGTGACCTCACCCTCGACAACAGAAAAAAATTTTGGTAAAAAACAACAGGAAAAAACTTTATTAAAAAAATGAGTATATATTGTGCCCTAAGACATGAGAATAGAAATCATTGGCCCTCTCTCTCTCTCTCTTTGTAGTTGTGATCCAGCCAAAGCGTAAGCCATTAGATGAAGAGATCCCAACATTATACAAAGTCATAACATCTCTCATATATCAGTATAAAAGCAAACATTAGAAGAATAAAAACAGATTCCTGCGTATTACAATATCAATCTTCTTTCCAGCTGATAATCTTGCTGCCCCGACTAGCGACGATTAGGTTTTCCCCTCTTTTACCGCTTTTGCCATCACATAACATAACCACCGATGGTGCTAAGAGATGGTAAACATAAGCGCATAATTTGGTTCATGCTAGGCCAATTAGTTTAACAAAAAAGAATTATACAAAATTCATAATACCTAGCGGTTATTCAACCCCAACAAAACACAACTGTTGGGGGAAAATGTACAACTTCATTTAGCATCGATACGGCCACAAAAAATGCTGAAAAGAGACGGACGACACAGATATGTTTAACCGGAGAGTGCACTTGAGATAACATACATGACGAATTAATCCAGATCCGCGATGTTCCTTTCACCATGGTAATTAAGTTCTCTGCTTCCACTGTGTTAACATCCCTGCTGCACAAAATTACAGTTCCGTTGCCAAGCATTTATTTCCTGTGGGTTAGGATAAATACCTTTAACCAGCAAACTGGGATTCGGGCAACATAAGCAGTGCTGAATTATTTATAAAAAAGTGTCAACCAAAAACATCAACGTCCCTTAGGCCTCAGTTTCGATAATCCTATGGAGCTTAAAGATGCTGAGTATCCGATATGGGCAATAAATCTATATACCTAGTCCGGTGGACAAACTATGTGAGGCTTATCAAGACAG

>AeSUS6 Chr28 Chr28:19819599..19821294

TAACCCACAGGAAATAAATGCTTGGCAACGGAACTGTAATTTTGTGCAGCAGGGATGTTAACACAGTGGAAGCAGAGAACTTAATTACCGTGGTGAAAGGAACATCGCGGATCTGGATTAATTCGTCATGTATGTTATCTCAAGTGCACTCTCCGGTTAAACATATCTGTGTCGTCCGTCTCTTTTCAGCATTTTTTGTGGCCGTATCGATGCTAAATGAAGTTGTACATTTTCCCCCAACAGTTGTGTTTTGTTGGGGTTGAATAACCGCTAGGTATTATGAGTTTTGTATAATTTTTTTTTGTTAAACTAATTGGCCTAGCATGAACCAAATTATGCGCTTATGTTTACCATCGCTTAGCACCATCGGTGGTTATGTTATGTGATGGCAAAAGCGGTAAAAGAGGGGAAAACCTAATCGTCGCTAGTCGGGGCAGCAAGATTATCAGCTGGAAAGAAGATTGATATTGTAATACGCAGGAATCTGTTTTTATTCTTCTAATGTTTGCTTTTATACTGATATATGAGAGTTTGTATAATGTTGGGATCTCTTCATCTAATGGCTTAAGCTTTGGCTGGATCACAACTACAAAGAGAGCGAGAGAGAGGGCCAATGAATTTCTCATTCTTCATGTTTGAGGGCACAATTAATACTCATTTTTTAATAAAGTTGTTTCGTTTTGTTTTTTTACCAAAATTTTTTCCTGTTGTCGAGGGTGAGGTCACTGAAGTGGTGTTTATCAGTTGAAAAAGAGCATTTATACATAATATGAAAGTTATACGAAAATTTACAATGAAAATTTATGTCAACTAGAGCCGAAAAAAGGGTGCAGAAACCTTGTACACTCCAAGGATACAACGAACCCCTAAAGCCTTTATATCCATAGGAATTTACAAATTTCCCAAAAAATTAAACAAGAAAGAGTAGAAAATGAACGTTACTTGACATGTAACTGGTTATTATTATGAACAGTAATCATTTAGGTACGTTTGTGTTTGGAATATGAATTTATAGATTGGTTTCACCAAGTAACAAAAAATGACAAAAAACCCTCAAAAGATACATTCACTTTCCAAATTTATTATCCTTTTCTTTTCCCCCTTTTTTCCCTTCTATAAATTCCTCTACAAGTCCTGTACTGTTGGTTTTTGGGTTACATGCATGCAGCTGCCTGTTATGGAAGTTTGGTTTGGAAGAAAAGAACAAAAAGTTCACCCATCTCGAAGGACATATAAACAAGGAATACTTTCCCTCCACACTTCCATTCAATCCATGCGAGATTTCCTTTTCGCGATGGTAAATTCCTTTCTTTCTTCATGTCGTCCTTTCCCACACTCCGCACCAAGGAATGTTCTCTCTCTTCTTCTTCTAGTCCCCCGCATTAAATCCCCACCCTATATATACGTATTCTCACAAATTTAAAATCACCTCACTTTTGAATTCACAAGCAACGACATTTTAACATCAGAATGGCTTCCGCAAAAGTTCTTAGGAAGTCGGACAGTGCAATAGCCGAGAGCTTGTCGGATGCTCTAAAGCAGAGCCGGTACCACACGAAGAGATGCTTTGCTAGGTATGCAATTCTTTTTTGTTCATAGAATTTCTCATTTTCCTTTTCCCCCCCTAATTGCTAACAAATGGATTTTGTGTGTGGTGGAAATAAGGTTGTTGAAACGGGGAAGAGGTTGATGAAAC
