# Supplementary material for: Genome-wide identification and expression profiling analysis of sucrose synthase (SUS) and sucrose phosphate synthase (SPS) genes family in Actinidia chinensis and A. eriantha
Source: BMC Plant Biol. 2022 Apr 26;22:215. doi: 10.1186/s12870-022-03603-y (PMC9040251; doi:10.1186/s12870-022-03603-y)
Supplement: Supplementary file 6 — Additional file 6. [file 12870_2022_3603_MOESM6_ESM.docx]

Supplementary file 6 Protein sequences of members of the *SPS* and *SUS* gene families in *Arabidopsis thaliana*.

>AT5G20280(AtSPS1)

MAGNDWVNSYLEAILDVGQGLDDARSSPSLLLRERGRFTPSRYFVEEVITGYDETDLHRSWVKAVATRSPQERNTRLENMCWRIWNLARQKKQHEEKEAQRLAKRRLEREKGRREATADMSEEFSEGEKGDIISDISTHGESTKPRLPRINSAESMELWASQQKGNKLYLVLISLHGLIRGENMELGRDSDTGGQVKYVVELARALGSMPGVYRVDLLTRQVSSPDVDYSYGEPTEMLTPRDSEDFSDEMGESSGAYIVRIPFGPKDKYIPKELLWPHIPEFVDGAMSHIMQMSNVLGEQVGVGKPIWPSAIHGHYADAGDATALLSGALNVPMLLTGHSLGRDKLEQLLRQGRLSKEEINSTYKIMRRIEGEELSLDVSEMVITSTRQEIDEQWRLYDGFDPILERKLRARIKRNVSCYGRFMPRMVKIPPGMEFNHIVPHGGDMEDTDGNEEHPTSPDPPIWAEIMRFFSNSRKPMILALARPDPKKNITTLVKAFGECRPLRELANLALIMGNRDGIDEMSSTSSSVLLSVLKLIDKYDLYGQVAYPKHHKQSDVPDIYRLAAKSKGVFINPAIIEPFGLTLIEAAAHGLPMVATKNGGPVDIHRVLDNGLLVDPHDQQSISEALLKLVADKHLWAKCRQNGLKNIHQFSWPEHCKTYLSRITSFKPRHPQWQSDDGGDNSEPESPSDSLRDIQDISLNLKFSFDGSGNDNYMNQEGSSMDRKSKIEAAVQNWSKGKDSRKMGSLERSEVNSGKFPAVRRRKFIVVIALDFDGEEDTLEATKRILDAVEKERAEGSVGFILSTSLTISEVQSFLVSGGLNPNDFDAFICNSGSDLHYTSLNNEDGPFVVDFYYHSHIEYRWGGEGLRKTLIRWASSLNEKKADNDEQIVTLAEHLSTDYCYTFTVKKPAAVPPVRELRKLLRIQALRCHVVYSQNGTRINVIPVLASRIQALRYLFVRWGIDMAKMAVFVGESGDTDYEGLLGGLHKSVVLKGVSCSACLHANRSYPLTDVISFESNNVVHASPDSDVRDALKKLELLKD

>AT5G11110(AtSPS2)

MVGNDWVNSYLEAILAAEPGIANSKPPGTGDSKSSLLLRERGHFSPTRYFVEEVITGFDETDLHRSWVQAAATRSPQERNTRLENLCWRIWNLARQKKQVEGKNAKREAKREREREKARREVTAEMSEDFSEGEKADLPGEIPTPSDNNTKGRMSRISSVDVFENWFAQHKEKKLYIVLISLHGLIRGENMELGRDSDTGGQVKYVVELARALGSMPGVYRVDLLTRQVTAPDVDSSYSEPSEMLNPIDTDIEQENGESSGAYIIRIPFGPKDKYVPKELLWPHIPEFVDRALSHIMQISKVLGEQIGGGQQVWPVSIHGHYADAGDSTALLSGALNVPMVFTGHSLGRDKLEQLLKQGRPKEEINSNYKIWRRIEAEELCLDASEIVITSTRQEVDEQWRLYDGFDPVLERKLRARMKRGVSCLGRFMPRMVVIPPGMEFHHIVPHDVDADGDDENPQTADPPIWSEIMRFFSNPRKPMILALARPDPKKNLVTLVKAFGECRPLRELANLTLIMGNRNDIDELSSTNSSVLLSILKLIDKYDLYGQVAMPKHHQQSDVPEIYRLAAKTKGVFINPAFIEPFGLTLIEAGAHGLPTVATINGGPVDIHRVLDNGLLVDPHDQQAIADALLKLVSDRQLWGRCRQNGLNNIHLFSWPEHCKTYLARIASCKQRHPKWQRVEFENSDSDSPSDSLRDINDISLNLKLSLDGEKSGSNNGVDTNLDAEDRAAERKAEVEKAVSTLAQKSKPTEKFDSKMPTLKRRKNIFVISVDCSATSDLLAVVKTVIDAAGRGSSTGFILSTSMTISETHTALLSGGLKPQDFDAVICSSGSELYFTSSGSEDKTALPYTLDADYHSHIEFRWGGESLRKTLIRWISSVEEKKKTKKGEILVEDESSSTNYCLSFKVKDPALMPPMKELRKLMRNQALRCNAVYCQNGARLNVIPVLASRSQALRYLLVRWGIDLSNMVVFVGDSGDTDYEGLLGGIHKTVILKGLASDLREQPGNRSYPMEDVTPLNSPNITEAKECGRDAIKVALEKLGISLLKP

>AT1G04920(AtSPS3)

MAGNEWINGYLEAILDSQAQGIEETQQKPQASVNLREGDGQYFNPTKYFVEEVVTGVDETDLHRTWLKVVATRNSRERNSRLENMCWRIWHLTRKKKQLEWEDSQRIANRRLEREQGRRDATEDLSEDLSEGEKGDGLGEIVQPETPRRQLQRNLSNLEIWSDDKKENRLYVVLISLHGLVRGENMELGSDSDTGGQVKYVVELARALARMPGVYRVDLFTRQICSSEVDWSYAEPTEMLTTAEDCDGDETGESSGAYIIRIPFGPRDKYLNKEILWPFVQEFVDGALAHILNMSKVLGEQIGKGKPVWPYVIHGHYADAGDSAALLSGALNVPMVLTGHSLGRNKLEQLLKQGRQSKEDINSTYKIKRRIEAEELSLDAAELVITSTRQEIDEQWGLYDGFDVKLEKVLRARARRGVNCHGRFMPRMAVIPPGMDFTNVEVQEDTPEGDGDLASLVGGTEGSSPKAVPTIWSEVMRFFTNPHKPMILALSRPDPKKNITTLLKAFGECRPLRELANLTLIMGNRDDIDELSSGNASVLTTVLKLIDKYDLYGSVAYPKHHKQSDVPDIYRLAANTKGVFINPALVEPFGLTLIEAAAHGLPMVATKNGGPVDIHRALHNGLLVDPHDQEAIANALLKLVSEKNLWHECRINGWKNIHLFSWPEHCRTYLTRIAACRMRHPQWQTDADEVAAQDDEFSLNDSLKDVQDMSLRLSMDGDKPSLNGSLEPNSADPVKQIMSRMRTPEIKSKPELQGKKQSDNLGSKYPVLRRRERLVVLAVDCYDNEGAPDEKAMVPMIQNIIKAVRSDPQMAKNSGFAISTSMPLDELTRFLKSAKIQVSEFDTLICSSGSEVYYPGGEEGKLLPDPDYSSHIDYRWGMEGLKNTVWKLMNTTAVGGEARNKGSPSLIQEDQASSNSHCVAYMIKDRSKVMRVDDLRQKLRLRGLRCHPMYCRNSTRMQIVPLLASRSQALRYLFVRWRLNVANMYVVVGDRGDTDYEELISGTHKTVIVKGLVTLGSDALLRSTDLRDDIVPSESPFIGFLKVDSPVKEITDIFKQLSKATA

>AT4G10120(AtSPS4)

MARNDWINSYLEAILDVGTSKKKRFESNSKIVQKLGDINSKDHQEKVFGDMNGKDHQEKVFSPIKYFVEEVVNSFDESDLYKTWIKVIATRNTRERSNRLENICWRIWHLARKKKQIVWDDGVRLSKRRIEREQGRNDAEEDLLSELSEGEKDKNDGEKEKSEVVTTLEPPRDHMPRIRSEMQIWSEDDKSSRNLYIVLISMHGLVRGENMELGRDSDTGGQVKYVVELARALANTEGVHRVDLLTRQISSPEVDYSYGEPVEMLSCPPEGSDSCGSYIIRIPCGSRDKYIPKESLWPHIPEFVDGALNHIVSIARSLGEQVNGGKPIWPYVIHGHYADAGEVAAHLAGALNVPMVLTGHSLGRNKFEQLLQQGRITREDIDRTYKIMRRIEAEEQSLDAAEMVVTSTRQEIDAQWGLYDGFDIKLERKLRVRRRRGVSCLGRYMPRMVVIPPGMDFSYVLTQDSQEPDGDLKSLIGPDRNQIKKPVPPIWSEIMRFFSNPHKPTILALSRPDHKKNVTTLVKAFGECQPLRELANLVLILGNRDDIEEMPNSSSVVLMNVLKLIDQYDLYGQVAYPKHHKQSEVPDIYRLAAKTKGVFINPALVEPFGLTLIEAAAYGLPIVATRNGGPVDIVKALNNGLLVDPHDQQAISDALLKLVANKHLWAECRKNGLKNIHRFSWPEHCRNYLSHVEHCRNRHPTSSLDIMKVPEELTSDSLRDVDDISLRFSTEGDFTLNGELDAGTRQKKLVDAISQMNSMKGCSAAIYSPGRRQMLFVVAVDSYDDNGNIKANLNEIIKNMIKAADLTSGKGKIGFVLASGSSLQEVVDITQKNLINLEDFDAIVCNSGSEIYYPWRDMMVDADYETHVEYKWPGESIRSVILRLICTEPAAEDDITEYASSCSTRCYAISVKQGVKTRRVDDLRQRLRMRGLRCNIVYTHAATRLNVIPLCASRIQALRYLSIRWGIDMSKTVFFLGEKGDTDYEDLLGGLHKTIILKGVVGSDSEKLLRSEENFKREDAVPQESPNISYVKENGGSQEIMSTLEAYGIK

>AT5G20830(AtSUS1)

MANAERMITRVHSQRERLNETLVSERNEVLALLSRVEAKGKGILQQNQIIAEFEALPEQTRKKLEGGPFFDLLKSTQEAIVLPPWVALAVRPRPGVWEYLRVNLHALVVEELQPAEFLHFKEELVDGVKNGNFTLELDFEPFNASIPRPTLHKYIGNGVDFLNRHLSAKLFHDKESLLPLLKFLRLHSHQGKNLMLSEKIQNLNTLQHTLRKAEEYLAELKSETLYEEFEAKFEEIGLERGWGDNAERVLDMIRLLLDLLEAPDPCTLETFLGRVPMVFNVVILSPHGYFAQDNVLGYPDTGGQVVYILDQVRALEIEMLQRIKQQGLNIKPRILILTRLLPDAVGTTCGERLERVYDSEYCDILRVPFRTEKGIVRKWISRFEVWPYLETYTEDAAVELSKELNGKPDLIIGNYSDGNLVASLLAHKLGVTQCTIAHALEKTKYPDSDIYWKKLDDKYHFSCQFTADIFAMNHTDFIITSTFQEIAGSKETVGQYESHTAFTLPGLYRVVHGIDVFDPKFNIVSPGADMSIYFPYTEEKRRLTKFHSEIEELLYSDVENKEHLCVLKDKKKPILFTMARLDRVKNLSGLVEWYGKNTRLRELANLVVVGGDRRKESKDNEEKAEMKKMYDLIEEYKLNGQFRWISSQMDRVRNGELYRYICDTKGAFVQPALYEAFGLTVVEAMTCGLPTFATCKGGPAEIIVHGKSGFHIDPYHGDQAADTLADFFTKCKEDPSHWDEISKGGLQRIEEKYTWQIYSQRLLTLTGVYGFWKHVSNLDRLEARRYLEMFYALKYRPLAQAVPLAQDD

>AT5G49190(AtSUS2)

MPTGRFETMREWVYDAISAQRNELLSLFSRYVAQGKGILQSHQLIDEFLKTVKVDGTLEDLNKSPFMKVLQSAEEAIVLPPFVALAIRPRPGVREYVRVNVYELSVDHLTVSEYLRFKEELVNGHANGDYLLELDFEPFNATLPRPTRSSSIGNGVQFLNRHLSSIMFRNKESMEPLLEFLRTHKHDGRPMMLNDRIQNIPILQGALARAEEFLSKLPLATPYSEFEFELQGMGFERGWGDTAQKVSEMVHLLLDILQAPDPSVLETFLGRIPMVFNVVILSPHGYFGQANVLGLPDTGGQVVYILDQVRALENEMLLRIQKQGLEVIPKILIVTRLLPEAKGTTCNQRLERVSGTEHAHILRIPFRTEKGILRKWISRFDVWPYLETFAEDASNEISAELQGVPNLIIGNYSDGNLVASLLASKLGVIQCNIAHALEKTKYPESDIYWRNHEDKYHFSSQFTADLIAMNNADFIITSTYQEIAGSKNNVGQYESHTAFTMPGLYRVVHGIDVFDPKFNIVSPGADMTIYFPYSDKERRLTALHESIEELLFSAEQNDEHVGLLSDQSKPIIFSMARLDRVKNLTGLVECYAKNSKLRELANLVIVGGYIDENQSRDREEMAEIQKMHSLIEQYDLHGEFRWIAAQMNRARNGELYRYIADTKGVFVQPAFYEAFGLTVVESMTCALPTFATCHGGPAEIIENGVSGFHIDPYHPDQVAATLVSFFETCNTNPNHWVKISEGGLKRIYERYTWKKYSERLLTLAGVYAFWKHVSKLERRETRRYLEMFYSLKFRDLANSIPLATDEN

>AT4G02280(AtSUS3)

MANPKLTRVLSTRDRVQDTLSAHRNELVALLSRYVDQGKGILQPHNLIDELESVIGDDETKKSLSDGPFGEILKSAMEAIVVPPFVALAVRPRPGVWEYVRVNVFELSVEQLTVSEYLRFKEELVDGPNSDPFCLELDFEPFNANVPRPSRSSSIGNGVQFLNRHLSSVMFRNKDCLEPLLDFLRVHKYKGHPLMLNDRIQSISRLQIQLSKAEDHISKLSQETPFSEFEYALQGMGFEKGWGDTAGRVLEMMHLLSDILQAPDPSSLEKFLGMVPMVFNVVILSPHGYFGQANVLGLPDTGGQVVYILDQVRALETEMLLRIKRQGLDISPSILIVTRLIPDAKGTTCNQRLERVSGTEHTHILRVPFRSEKGILRKWISRFDVWPYLENYAQDAASEIVGELQGVPDFIIGNYSDGNLVASLMAHRMGVTQCTIAHALEKTKYPDSDIYWKDFDNKYHFSCQFTADLIAMNNADFIITSTYQEIAGTKNTVGQYESHGAFTLPGLYRVVHGIDVFDPKFNIVSPGADMTIYFPYSEETRRLTALHGSIEEMLYSPDQTDEHVGTLSDRSKPILFSMARLDKVKNISGLVEMYSKNTKLRELVNLVVIAGNIDVNKSKDREEIVEIEKMHNLMKNYKLDGQFRWITAQTNRARNGELYRYIADTRGAFAQPAFYEAFGLTVVEAMTCGLPTFATCHGGPAEIIEHGLSGFHIDPYHPEQAGNIMADFFERCKEDPNHWKKVSDAGLQRIYERYTWKIYSERLMTLAGVYGFWKYVSKLERRETRRYLEMFYILKFRDLVKTVPSTADD

>AT3G43190(AtSUS4)

MANAERVITRVHSQRERLDATLVAQKNEVFALLSRVEAKGKGILQHHQIIAEFEAMPLETQKKLKGGAFFEFLRSAQEAIVLPPFVALAVRPRPGVWEYVRVNLHDLVVEELQASEYLQFKEELVDGIKNGNFTLELDFEPFNAAFPRPTLNKYIGDGVEFLNRHLSAKLFHDKESLHPLLKFLRLHSHEGKTLMLNNRIQNLNTLQHNLRKAEEYLMELKPETLYSEFEHKFQEIGLERGWGDTAERVLNMIRLLLDLLEAPDPCTLENFLGRIPMVFNVVILSPHGYFAQDNVLGYPDTGGQVVYILDQVRALETEMLQRIKQQGLNITPRILIITRLLPDAAGTTCGQRLEKVYGSQYCDILRVPFRTEKGIVRKWISRFEVWPYLETFTEDVAAEISKELQGKPDLIIGNYSDGNLVASLLAHKLGVTQCTIAHALEKTKYPDSDIYWKKLDEKYHFSCQFTADLIAMNHTDFIITSTFQEIAGSKDTVGQYESHRSFTLPGLYRVVHGIDVFDPKFNIVSPGADMSIYFAYTEEKRRLTAFHLEIEELLYSDVENEEHLCVLKDKKKPIIFTMARLDRVKNLSGLVEWYGKNTRLRELVNLVVVGGDRRKESQDNEEKAEMKKMYELIEEYKLNGQFRWISSQMNRVRNGELYRYICDTKGAFVQPALYEAFGLTVVEAMTCGLPTFATCNGGPAEIIVHGKSGFHIDPYHGDKAAESLADFFTKCKHDPSHWDQISLGGLERIQEKYTWQIYSQRLLTLTGVYGFWKHVSNLDRLESRRYLEMFYALKYRPLAQAVPLAHEE

>AT5G37180(AtSUS5)

MEMTSGSLGNGIPEAMGQNRGNIKRCLEKYIENGRRVMKLNELMDEMEIVINDVTQRRRVMEGDLGKILCFTQEAVVIPPNVAFAVRGTPGNWQYVKVNSSNLSVEALSSTQYLKLKEFLFDENWANDENALEVDFGALDFTLPWLSLSSSIGNGLSFVSSKLGGRLNDNPQSLVDYLLSLEHQGEKLMMNETLNTARKLEMSLILADVFLSELPKDTPFQAFELRFKECGFEKGWGESAGRVKETMRILSEILQAPDPQNIDRFFARVPRIFNVVIFSVHGYFGQTDVLGLPDTGGQVVYILDQVKALEDELLQRINSQGLNFKPQILVVTRLIPDAKKTKCNQELEPIFGTKYSNILRIPFVTENGILRRWVSRFDIYPYLERFTKDATTKILDILEGKPDLIIGNYTDGNLVASLMANKLGITQATIAHALEKTKYEDSDIKWKEFDPKYHFSSQFTADLISMNSADFIIASTYQEIAGSKERAGQYESHMSFTVPGLYRVVSGINVFDPRFNIAAPGADDSIYFPFTAQDRRFTKFYTSIDELLYSQSENDEHIGYLVDKKKPIIFSMARLDVVKNLTGLTEWYAKNKRLRDLVNLVIVGGFFDASKSKDREEISEIKKMHSLIEKYQLKGQFRWITAQTDRTRNGELYRSIADTRGAFVQPAHYEAFGLTVIEAMSCGLVTFATNQGGPAEIIVDGVSGFHIDPSNGEESSDKIADFFEKSGMDPDYWNMFSNEGLQRINECYTWKIYANKVINMGSTYSYWRHLNKDQKLAKQRYIHSFYNLQYRNLVKTIPILSDIPEPPPLPPKPLVKPSASKGSKRTQPRLSFRLFGA

>AT1G73370(AtSUS6)

MSSSSQAMLQKSDSIAEKMPDALKQSRYHMKRCFASFVGGGKKLMKREHLMNEIEKCIEDSRERSKILEGLFGYILTCTQEAAVVPPFVALAARPNPGFWEYVKVNSGDLTVDEITATDYLKLKESVFDESWSKDENALEIDFGAIDFTSPRLSLSSSIGKGADYISKFISSKLGGKSDKLEPLLNYLLRLNHHGENLMINDDLNTVAKLQKSLMLAVIVVSTYSKHTPYETFAQRLKEMGFEKGWGDTAERVKETMIILSEVLEAPDNGKLDLLFSRLPTVFNVVIFSVHGYFGQQDVLGLPDTGGQVVYILDQVRALEEELLIRINQQGLGFKPQILVVTRLIPEARGTKCDQELEAIEGTKHSHILRVPFVTNKGVLRQWVSRFDIYPYLERFTQDATSKILQRLDCKPDLIIGNYTDGNLVASLMATKLGVTQGTIAHALEKTKYEDSDAKWKELDPKYHFSCQFTADLIAMNVTDFIITSTYQEIAGSKDRPGQYESHTAFTMPGLCRVVSGIDVFDPKFNIAAPGADQSVYFPYTEKDKRFTKFHPSIQELLYNEKDNAEHMGYLADREKPIIFSMARLDTVKNITGLVEWYGKDKRLREMANLVVVAGFFDMSKSNDREEKAEIKKMHDLIEKYKLKGKFRWIAAQTDRYRNSELYRCIADTKGVFVQPALYEAFGLTVIEAMNCGLPTFATNQGGPAEIIVDGVSGFHIDPNNGDESVTKIGDFFSKCRSDGLYWDNISKGGLKRIYECYTWKIYAEKLLKMGSLYGFWRQVNEDQKKAKKRYIEMLYNLQFKQLTKKVTIPEDKPLPLRLASLRNLLPKKTTNLGAGSKQKEVTETEKTKQKSKDGQEQHDVKVGEREVREGLLAADASERVKKVLESSEEKQKLEKMKIAYGQQHSQGASPVRNLFWSVVVCLYICYILKQRFFGANSAQEY
