# Supplementary material for: Genome-wide identification and expression profiling analysis of sucrose synthase (SUS) and sucrose phosphate synthase (SPS) genes family in Actinidia chinensis and A. eriantha
Source: BMC Plant Biol. 2022 Apr 26;22:215. doi: 10.1186/s12870-022-03603-y (PMC9040251; doi:10.1186/s12870-022-03603-y)
Supplement: Supplementary file 7 — Additional file 7. [file 12870_2022_3603_MOESM7_ESM.docx]

Supplementary file 7 Protein sequences of members of the *SUS* gene families in *Actinidia*.

>AcSUS1

MAALKRSDSIADSMPDALRESRYHMKKCLAKYIEKGKRLMKLHHLMSEMEKVIDDKTEREQILNGLLGYILCTTQEAVVIPPYVAFAIRPNPGFWEFVKVSSTDLSVEGITATDYLKSKEMLVDEDWAKDENALEVDFGAMDFSEPNLTMSSSIGNGINFISKFLSSILYGGSQKAQPLVDYLLSLNHHEEKLMINETLNTAAKLQSALIVAEAALLTLPKDTPYQDFEQRFRQWGFEKGWGDTAERVRETMRSLSEIFQAPDPLNMDKFFGRVPTVFNVVLFSVHGYFGQSDVLGLPDTGGQVVYVLDQVVAFEEELLVRIKQQGLNVKPQILVVTRLIPDAKGTKCNQVLEPIANTKHSNILRVPFRTEDGVLPQWVSRFDIYPYLESSVFNQQDATDKILEVMEGKPDLIIGNYTDGNLVASLMASKLGITLGTIAHALEKTKYEDSDLKWKQLDPKYHFSCQFTADTIAMNSADFIITSTYQEIAGSKDRPGQYESHAAFTLPGLCRVVSGINVFDPKFNIAAPGADQSVYFPYTDRQKRFTSFRPAIEELLFSKVNNNEHIGYLEDRKKPILFSMARLDIVKNISGLTEWYGKNKRLRSLANLVVVAGFFDPTKSKDREEAAEITKMHMLIENYKLKGQIRWIAAQTDRQRNGELYRCIADTKGAFVQPALYEAFGLTVITAVVSVGVIFIPIGLPCLHQNM

>AcSUS2

MAALKRSESMADSMPDALRESRYHMKKCFAKYIEQGKRLMKLRHLMSEMEKVIDDKTEREQFLNSLLGYILCTTQEAVVIPPYVAFAIRPNPGFWEFIKVSSTDLSVEGITATDYLKYKEMLVDEDWAKDENALEVDFGAMDFSTPSLTVSSSIGNGINFVSKFLSSKLHGGLQKAQPLVDYLLSLNYHEEKLMINETINTAAKLQSALIVAEAALLTLPKDTPYQDFEQRFRQWGFEKGWGDTAERVRETMRSLSEIFQAPDPLNMEKFFGRVPTVFKVVLFSVHGYFGQSDVLGLPDTGGQVVYVLDQVVAFEEELLVRIKQQGLNVKPQILVVTRLIPDAKGTKCNQALEPVANTKHSNILRVPFRTENGDLPQWVSRFDIYPYLESITLFFNQQDATDKILEIMEGKPDLIIGNYTDGNLVASLMASKLGITLGTIAHALEKTKYEDSDLKWKELDPKYHFSCQFTADTIAMNSSDFIITSTYQEIAGSKDRPGQYESHAAFTLPGLCRVVSGINVFDPKFNIAAPGADQSVYFPYTDRQKRFTKFHPSIEELLFSKVDNIEHIGYLEDRKKPILFSMARLDIVKNISGLTEWYGKNKRLRILVNLVVVAGFFDPTKSKDREEAAEIKKMHMLIEKYQLKGQIRWIAAQTDRQRNGELYRCIADTKGAFAQPALYEAFGLTVKNVPTSRVEPQQQPNEKQPKAQPSQSFFINKPIGWCLLHVLDSKFTQQDLPACKPILTHGWVPKQMKSPIYIYYQLDNFYQNHRRYVNSRSDEQLRSKAYEFDTDDCDPESNTDKGPIVPCGLIAWSLFNDTYGFSLQNKVLQVSKKGIAWKSDQKHKFGSDVYPKNFQSSGLIGGAKLNKSIPLSNQVDLIVWMRTAALPTFRKLYGKIEVDLEANQKITVVIQNNYNTYSFGGKKKLVLSTTSWFGGKNDFLGIAYLTVGGLSLFMAISFILLYVVKPRNITSAVRKHICKTSIGWKFVGEVQSSNFDMAGTKKTKRLKAQVKVAKLANIWKFIDQSCY

>AcSUS3

MMEKQNETIIPWAVQYVEQGKGILQPHHLIDELDKIVGDDEANLTLINGPFGEVLKSAQVLMLNDRIYSMPRLQSALTKAEGYLAKLSADTPYSEFEHDFQVMGFERGWGDTAGRVLEMMHLLLDILQAPDPTSLETFLGRIPMVFNVVILSVHGYFGQAKVLGLPDTGGQIVYILDQVRALENEILMRHKQQGLDVTPRILVVTRLIPDAKGTSCNQRLERISGTQHAHILRVPFRTDKGILRKWISRFDVWPYLEKFTEDAASEIAAELQGVPDLIIGNYSDGNLVASLLAHKMGVTQCTIAHALEKTKYPDSDIYWKKFEDKYHFSCQFTADLIAMNSSDFIITSTFQEIAGTKNTVGQYESHSAFTLPSLYRVVHGIDVFDPKFNIVSPGADMCIYFPYFEKEKRLTALHGSIEKLLYDPEQNEEHIGTLSDSSKPIIFSMARLDHVKNITGLVEFYAKNTKLRELVNLVVVAGYNDVKKSNDREEIDEIEKMHSLIKEYNLDGQFRWISSQTNRARNGELYRYMADKRGAFVQPAFYEAFGLTVVEAMTCGLPTFATCHGGPAEIIEDGISGFHIDPYHPDKVSAILADFFQRCKDDPSYWEKISKAGLQRILERYTWKIYSERLMTLSGVYGFWKYVSKLERRETLRYLEMFYILKYRDLVKSVPLAIDGED

>AcSUS4

MVARPRKKTRREKSAKPWLWIPYDYYDYYDYSIRLVFAFFNIFDKQHTSKTPYSEFEHKFQEIGLERGWGDTAERVLEMLHMLLELLEAPDPCTLEKFLGRIPMVFNVVILSPHGYFAQENVLGYPDTGGQVVYILDQVPAMEKEMLKRIKQQGLDIIPRILIVTRLLPDAVGTTCNQRIEKVYGAEHSHILRVPFRTEKGIVRQWISRFEVWPYMERFTEDVAHDIVTELQAKPDLVIGNYSEGNLVASLLAHKLGVTQCTIAHALEKTKYPDSDIYLKKFDEKYHFSCQFTADLIAMNHTDFIITSTFQEIAGSKNTVGQYESHMAFTMPGLYRVVHGIDVFDPKFNIVSPGADMNIYFPHTEKDKRLTKFHPEIEDLLFSDVENKEHIGVLKDRTKPIIFSMARLDRVKNLTGLVELYGKNARLRELANLVVVGGDRRKESKDLEEQAEMKKMYDLIETYKLNGQFRWISSQMNRVRNGELYRCIADTKGVFVQPAFYEAFGLTVVESMTCGLPTFATCHGGPAEIIIHGKSGFHIDPYHGDQVAELLVNFYEKCKVDPSHWDAISEGGLKRILEKYTWQIYSERLMTLAGVYGFWKYVSKLDRRETRRYLEMFYALKYRKLAEAVPLAVDQ

>AcSUS5

MIHMLLELLEAPDPCTLEKFLGRIPMVFNVVILSPHGYFAQENVLGYPDTGGQVVYILDQVPAMEKEMLKRIKQQGLDIIPRILIVTRLLPDAVGTTCHQRIEKVYGAEHSHILRVPFRTEEGIVRKWISRFEVWPYMERFTEDVAHDIVTELQAKPDLIIGNYSEGNLVASLLAHKLGVTQCTIAHALEKTKYPDSDIYLKQFDEKYHFSCQFTADLIAMNHTDFIITSTFQEIAGSKNTVGQYESHMAFTMPGLYRVVHGIDVFDPKFNIVSPGADMNIYFPHTEKDKRLTKFHPEIEDLLFSDVENKEHIGVLKDPTKPIIFSMARLDRVKNLTGLVELYGKNARLRELANLVVVGGDRRKESKDLEEQAEMKKMYDLIETYKLNGQFRWISSQMNRVRNGELYRFIADTKGVFVQPAFYEAFGLTVVEAMTCGLPTFATCHGGPAEIIIHGKSGFHIDPYHGDQVAELLVNFYEKCKVDPSHWDAISEGGLKRILEKYTWQIYSERLMTLAGVYGFWKYVSKLDRRETRRYLEMFYALKYRKLAEAVPLAVDQ

>AcSUS6

MSTAKLARIPSMRERVEDTLSAHRNELVSLLSRYVEQGKGILQPHHLIDELDKIVGDDEANLTLSDGPFGEVLKSTQVLMLNDRIYSMPRLQSALTKAEGYLAKLSADTPYSEFEHDFQVMGFERGWGDTAGRVLEMMHLLLDILQAPDPTALETFLGRIPMVFNVVILSVHGYFGQANVLGLPDTGGQIVYILDQVRALENEILVRHKQQGLDVIPRILVVTRLIPDAKGTSCNQRLERISGTQHAHILRVPFRTDKGILRKWISRFDVWPYLEKFTEDAASEIAAELQGVPDLIIGNYSDGNLVASLLAHKMGVTQCTIAHALEKTKYPDSDIYWKKFEEKYHFSCQFTADLIAMNSSDFIITSTFQEIAGTKNTVGQYESHTAFTLPSLYRVVHGIDVFDPKFNIVSPGADMCIYFPYFEKEKRLTALHGSIEKLLYDPEQNEVHIGTLSDPSKPIIFSMARLDRVKNITGLVECYAKNTKLRELVNLVVVAGYNDVKKSNDREEIDEIEKMHCLIKEYNLDGQFRWISAQTNRARNGELYRYIADKRGAFVQPAFYEAFGLTVVEAMTCGLPTFATCHGGPAEIIEDGISGFHIDPYHPDKVSASLADFFQRYTWKIYSERLMTLSGVYGFWKYVSKLERRETRRYLEMFYIIKYRDLVKSVPLAIDEEH

>AeSUS1

MAGQVLTRVHSLRERLDGTLSAHRNEILLFLSKIESHGKGILKPHQIEAEIEALSKEVQQKLYDGAFGELLKSAQEAIVLPPWIAFAVRLRPGVWEYMRVNLNALVVEELSVPEYLQFKEELVDGPCNGNFILELDFEPFTASFPRPTLSKSIGNGVEFLNRHLSAKMFHDKESMHPLLDFLKVHNYNGKTMMLNDRIQNLNALQFVLRKAEEYLLTLPLETPYSEFEHKFQEIGLERGWGDTAERVLEMIHMLLELLEAPDPCTLEKFLGRIPMVFNVVILSPHGYFAQENVLGYPDTGGQVVYILDQVPAMEKEMLKRIKQQGLDISSHSIVVTRLLPDAVGTTCNQRIEKVYGAEHSHILRVPFRTEEGIVRKWISRFEVWPYMERFTEDVAHDIVTELQAKPDLIIGNYSEGNLVASLLAHKLGVTQCTIAHALEKTKYPDSDIYLKKFDEKYHFSCQFTADLIAMNHTDFIITSTFQEIAGRYYFLAVVLFIMLICYTHCVLRKNTVGQYESHMAFTMPGLYRVVHGIDVFDPKFNIVSPGADMNIYFPHTEKDKRLTKFHPEIEDLLFSDVENKEHIGVLKDRTKPIIFSMARLDRVKNLTGLVELYGKNARLRELANLVVVGGDRRKESKDLEEQAEMKKMYDLIETYKLNGQFRWISSQMNRVRNGELYRFIADTKGVFVQPAFYEAFGLTVVEAMTCGLPTFATCHGGPAEIIIHGKSGFHIDPYHGDQVAELLVNFYEKCKVDPSHWDAISEGGLKRILEKYTWQIYSERLMTLAGVYGFWKYVSKLDRRETRRYLEMFYALKYRKLAEAVPLAVDQ

>AeSUS2

MLLELLEAPDPCTLEKFLGRIPMVFNVVILSPHGYFAQENVLGYPDTGGQFCPGEEMLKRIKQQGLDIIPRILIVSVTICTRAFFSPDAVGTTCISALISLWSRTFAYTFRVTLDWKGIVRQWISRFEVWPYMERFTEDVAHDIVTELQAKPDLVIGNYSEGNLVASLLAHKLGVTQCTIAHALEKTKYPDSDIYLKKFDDKYHFSCQFTADLIAMNHTDFIITSTFQEIAGSKNTVGQYESHMAFTMPGLYRVVHGIDVFDPKFNIVSPGADMNIYFPHTEKDKRLTKFHPEIEDLLFSDVENKEHIGVLKDRTKPIIFSMARLDRVKNLTGLVELYGKNARLRELANLVVVGGDRRKESKDLEEQAEMKKMYDLIETYKLNGQFRWISSQMNRVRNGELYRCIADTKGVFVQPAFYEAFGLTVVESMTCGLPTFATCHGGPAEIIIHGKSGFHIDPYHGDQVAELLVNFYEKCKIDPSHWDAISEGGLKRILEKYTWQIYSERLMTLAGVYGFWKYVSKLDRRETRRYLEMLCTQVPQVGKLRQFLWLLISRGMIGK

>AeSUS3

MAALKRSESMADSMPDALRESRYHMKKCFAKYIEQGKRLMKLRHLMSEMEKVIDDKTEREQFLNSLLGYILCTTQEAVVIPPYVAFAIRPNPGFWEFIKVSSTDLSVEGITATDYLKYKEMLVDEDWAKDENALEVDFGAMDFSAPSLTVSSSIGNGINFVSKFLSSKLHGGSQKAQPLVDYLLSLNYHEEKLMINETINTAAKLQSALIVAEAALLTLPKDTPYQDFEQRFRQWGFEKGWGDTAERVRETMRSLSEIFQAPDPLNMEKFFGRVPTIFKVVLFSVHGYFGQSDVLGLPDTGGQVVYVLDQVVAFEEELLVRIKQQGLNVKPQILVVTRLIPDAKGTKCNQALEPVANTKHSNILRVPFRTENGDLPQWDATDKILEIMEGKPDLVIGNYTDGNLVASLMASKLGITLGTIAHALEKTKYEDSDLKWKELDPKYHFSCQFTADTIAMNSSDFIITSTYQEIAGSKDRPGQYESHAAFTLPGLCRVVSGINVFDPKFNIAAPGADQSVYFPYTDRPKRFTKFHPSIEELLFSKVDNIEHMLDIVKNISGLTEWYGKNKRLRSLVNLVVVAGFFDPTKSKDREEAAEIKKMHMLIEKYQLKGQIRWIAAQTDRQRNGELYRCIADTKGAFAQPALYEAFGLTVIEAMNCGLPTFATNQGGPAEIIVDGLSGFHIDPNNGDESGNKIADFFQKCKDDPEHWNKISKLGLNRIYECYTWKIYANKVLNMGCVYSFWRQLNKDQKHAKQRYIQMFYNLQFRNLHQAHTKSVSEVVRIVKTQTQDSLIQCLPVVILSFFLKKK

>AeSUS4

MAALKRSESIADSMPDALRESRYHMKKCFAKYIEKGKRLMKLHHLMSEMEKEAVVIPPYVAFAIRPNPGFWEFVKVSSTDLSVEGITATDYLKSKEMLVDEDWAKDENALEVDFGAMDFSAPNLTMSSSIGNGINFISKFLSSILYGGSQKAQPLVDYLLSLNHHEEKLMINETLNTAAKLQSALIVAEAALLTLPKDTPYQDFEQRFRQWGFEKGWGDTAERVRETMRSLSEIFQAPDPLNMDKFFGRVPTVFNVVLFSVHGYFGQSDVLGLPDTGGQVVYVLDQVVAFEEELLIRIKQQGLNVKPQILVVTRLIPDAKGTKCNQVLEPIANTKHSNILRVPFRTEDGVLPQWVSRFDIYPYLESSVVNQQDATDKILEVMEGKPDLIIGNYTDGNLVASLMASKLGITLGTIAHALEKTKYEDSDLKWKQLDPKYHFSCQFTADTIAMNSADFIITSTYQEIAGSKDRPGQYESHAAFTLPGLCRVVSGINVFDPKFNIAAPGADQSVYFPYTDRQKRFTSFRPAIEELLFSKVDNNEHMLDIVKNISGLTEWYGKNKRLRSLANLVVVAGFFDPTKSKDREAARKINKNAHGVIENYKCKAQTDRQRNGELYRCIADTKGAFVQPALYEAFGLTVIEAMNCGLPTFATNQGGPAEIIVDGVSGFHIDPNNGDESGNKIADFFQKCKDDPDHWDRIQCGLYRKIMQQVLNMGCVYSFWRHLKKDQKQAKQRYIQMFYNLQFRNLVKNVPTSRVEPQQQPKEKQPKAQPSQNVKRTQSRFQRLFGS

>AeSUS5

MASAKVLRKSDSAIAESLSDALKQSRYHTKRCFARFVETGKRLMKPRHLIEEMEKVIGDKSERAKVLEGLLGLIISSTQEAAVVPPNVALAVRRSPGFWEFFKVNVDDLTVDAISAKDYLKLKETICDENWAKDENALELDFGAFDFSSRRLTLSSSIGNGVDFISKFMASKTSGDLEHSKPLLEYLLALNHHGENLMINETLNTFPKLQEALIVADVYLSALPKDTPYQNFEKKLKDWGFEKGWGDNAERVRDTMTILSEIFQAPDPTKMESFFRRLPNIFNIVIFSVHGYFGQADVLGLPDTGGQVVYILDQVKALEEELLLRIKQQGLSVKPQILVVTRLIPDAQGTKCNQEIEPVLNTAHSHIIRVPFMTDKGVLRQWDATAKVLGHLECKPDLILGNYTDGNLVASLMANKLGGTIAHALEKTKYEDSDIKWKELDPKYHFSCQFTADIIAMNSADFIITSTYQEIAGSKNRPGQYESHMAFTMPGLSRVVSGINVFDPKFNIAAPGAEQEKPIIFSMARLDTVKNISGLTEWYGKNKRLRNLANLVVVAGFFDPSKSKDREEIAEINKMHALIQKYQLKGQIRWIAAQTDRYRNGELYRCIADTKGAFVQPALYEAFGLTVIEAMNCGLPTFATNQGGPAEIIVDGVSGFHVDPNNGDESSNKIADFFDKCKGDAEYWNRMSKAGLQRIYECYTWKIYANKVLNMGSLYGFWKQLNIEQKKAKQRYLQMFYTLQFRNLFICHLDFKHASTRMIIVLRENGLEQQKQLASPRDAHTSCPCSSWCFLFLSVSIIIYAAMKYYGFFRQP

>AeSUS6

MKPRHLIEEMEKVIGDKSERAKVLEGLLGLIISSTQEAAVVPPNVALAVRRSPGFWEFFKVNVDDLTVDAISAKDYLKLKETICDENWAKDENALELDFGAFDFSSRRLTLSSSIGNGVDFISKFMASKTSGDLEHSKPLLEYLLALNHHGENLMINETLNTFPKLQEALIVADVYLSALPKDTPYQNFEKKLKDWGFEKGWGDNAERVRDTMTILSEIFQAPDPTKMESFFRRLPNIFNIVIFSVHGYFGQADVLGLPDTGGQVVYILDQVKALEEELLLRIKQQGLSVKPQILVVTRLIPDAQGTKCNQEIEPVLNTAHSHIIRVPFMTDKGVLRQWDATAKVLGHLECKPDLILGNYTDGNLVASLMANKLGTKYEDSDIKWKELDPKYHFSCQFTADIIAMNSADFIITSTYQEIAGSKNRPGQYESHMAFTMPGLSRVVSGINVFDPKFNIAAPGAEQEKPIIFSMARLDTVKNISGLTEWYGKNKRLRNLANLVVVAGFFDPSKSKDREEIAEINKMHALIQKYQLKGQIRWIAAQTDRYRNGELYRCIADTNGAFVQPALYEAFGLTVIEAMNCGLPTFATNQGGPAEIIVDGVSGFHVDPNNGDESSNKIADFFEKCKTDADYWNRMSQAGLKRIYECYTWKIYANKVLNMGSLYGFWKQLNNEQKKAKQRYLQMFYTLQFRNLYIFHRDFKHASTRMIIVLRENGLEQQKQLASPRDSHTSCPCSSWCFLFLSVSIIIYAAMKYYGFFRQPYPMCGCYQNVRFF
