# Supplementary material for: Genome-wide identification and expression profiling analysis of sucrose synthase (SUS) and sucrose phosphate synthase (SPS) genes family in Actinidia chinensis and A. eriantha
Source: BMC Plant Biol. 2022 Apr 26;22:215. doi: 10.1186/s12870-022-03603-y (PMC9040251; doi:10.1186/s12870-022-03603-y)
Supplement: Supplementary file 8 — Additional file 8. [file 12870_2022_3603_MOESM8_ESM.docx]

Supplementary file 8 Protein sequences of members of the *SPS* gene families in *Actinidia*.

>AcSPS1

MAGNDWINSYLEAILDVGPGIDDAKSSLLLRERGRFSPTRYFVEQVIGFDETDLYRSWAAATRSPQERNTRLENMCWRIWNLARQKKQLEGEEAQRMAKRRLERERGRREATADMSEDLSEGEKGDTVSDLSAHGESNRGRLPRISSVETMEAWVSQHKGKKLYIVLIRHEFEFHSFGLQIFLHGLIRGENMELGRDSDTGGQVKYVVELARALGSMPGVYRVDLLTRQVSSPEVDWSYGEPTEMLPPRNSDGLMDEMGESSGAYIIRIPFGPRDKYVPKELLWPHIPEFVDGALNHIIQMSKVLGEQIGSGHPVWPVAIHGHYADAGDAAALLSGALNVPMLFTGHSLGRDKLEQLLRQSRLSKDEINKTYKIMRRIEAEELSLDASEIVITSTRQEIEQQWRLYDGFDPVIERKLRARIRRNVSCYGRFMPRMVVMPPGMEFHHIVPHEGDMDGETEGNEDQPTSPDPPIWPEIVRFFTNPRKPMILALARPDPKKNLATLVEAFGECRPLRELANLTLIMGNRGDVDEMSSTNSSVLLSILKLIDKYDLYGQVAYPKHHKQSDVPDIYRLAAKTKGVFINPAVIEPFGLTLIEAAAYGLPIVATKNGGPVDIHRALDNGLLVDPHDQKSIADALLKLVADKQLWSKCRQNGLKNIYLFSWPEHCKTYLSRIAACKLRQPWWQRSDDGDENSESDSPSDSLRDISLNLKFSLDGEKNEGSGNADGSLEFEDRKIKLENAVLTWSKGFQKGTQKAGVTEKADTNITAGKFPVLRRRKNIIVIAVDFGAISDYSDSIRKIFDAVEKERTEGSIGFILATSFTLSEVHSFLISGGLSPSDFDAFICNSGSDLYYSSLNSEDNPFVVDLYYHSHIEYRWGGEGLRKTLIRWMGSINDKKGENEEQIVTEDEKISTNYCYAFKVRNAGKVPPVKEIRKLMRIQAHRCHVIYCQNGNKINVIPVLASRSQALRYLYLRWGVDLSKMVVFVGESGDTDYEGLLGGIHKSVILKGVCSGPTNQLHANRTYPLSDVLPIDSPNIVQAAEECSSADLRTSLLKLGFIKG

>AcSPS2

MAGNDWINSYLEAILDVGPGIDDAKSSLLLRERGRFSPTRYFVEQVIGFGETDLYRSWVKAAATRSPQERNTRLENMSWRIWNLLEGEEAQRMATRRLEHERGLREATADMSDDLSDGDKGDTDSDLSAHGESNRGRLPRISSVKTMEAWVSQQKGKKLLHGLIRGENMELGRDSDTGGQVKYVVELARALGSMPGVYRVDLLTRQVSSPEVDWSYGEPTEMLTPRNSDVLMDEIGESSGAHIIRIPFGPRDKYVPKELLWPHIPEFVDGALNHIIQMSKVLDEQIGSGHPVWPVAIHGHYADAGDAAALLSGVLNVPMLFTGHSLGRDKLEQLLRQSRLSKDEINKTYKIMRRIEAEELSLHASEIVITSTRQEIEEQWRLYDGFDPVLERKLRARIRRNVSCYGRFMPRMVVIPPGVEFHHIVPHEGDMDGETEGNEDQPTSPDPPIWPEIMRFFTNPRKQMILALARPDPKNNLTTLVEAFGECRPLRELANLTLIMGNRDDVTEMSSTNSSVLLSILELIDKYDLYGQVAYLKHHKQSDVPDIYRLAAKTKGVFINPAFIEPFGLTLIEAAAYGLPIVATKNGGPVDIHWALDSGFLVDPHDRQSIADALLKLVVDKQLWAKCRQNGLKNIHLFSWREHCKTYLSRIAACKLRQPWWQRNQDSTAGKFPALRRKNIIVIAVDFGAISDLSESIRKIFDAVAKERTEGSIGFVLATSFTLSEVQSFLISGGLSPSDFDAFICNSGSDIYYSSLNPEDNPFVVDLYYHSHIEYRWGGEGLRKTLIRWAGSITDKKGENEEQIVTEDEKISTNYCYAFKVRNAGKVPPVKEIRKLMRIQALRCHVIYCQNGNKINVIPVLASRSQALRYLYLRWGVDLSKMVVFVGETGDTDYEGLLGGIHKSVILKGVCSGPTHQLHANRTYPLSDVLPIDSPNIVQAAEKCSGADLRTSLGKLGFIKC

>AcSPS3

MPGVYRVDLLTRQVSAPDVDWSYGEPTEMLNTRSSENDMKETGESSGAYIIRIPFGPKDKYIPKEFLWPHISEFVDGALSHVIQMSKVLGEQIGGGEPVWPVAIHGHYADAGDSASLLSGALNIPMLITGHSLGRDKLEQILQQGRQSKEEINATYRIMRRIEAEEISLDASEVVITSTRQEIEEQWRLYDGFDPIIERKLRARIKRNVSCHGRFMPRMVVIPPGMEFHHIVPHDSDMDRETEGNEDNPATPDPPIWSEVLTIPCRYLSAQRTPCLSNEGLPYIFYRGNFLFLQIMRFFTNPRKPMILALARPDPKKNITNLVKAFGECKPLRELANLTLIMGNRDDVDEMSGTNASVLLSILKLIDKYDLYGQVAYPKHHKQYEVPDIYRLAAKSKAAAHGLPTVATKNGGPVDINRVCSLLEPSYCKVLDNGLLVDPHNQQSIADALLKLVADKQLWAKCRHNGLKNIHLFSWPEHCKTYLSRIASCKPRQPRWQKSDDGYEKSDSDSPGDSLRDIQDLSLNLKISLDGDKGSGTLDNALDCEENASGGKNRLENAVSVLSKGEEQNPRKAGSMPKLDYNNSKFLTLRRRKFVFVIAVDCDMTSEYLKMVKVIVEAAGENKSGFIGFILSTASSIPEIFSCLKSGGLNPMDFDAFICNSGSELYYPSSTSEVGPFGLPVVADSDYHSHIDYRWGGEGLRKTLVRWAASMNDKKGAGGGQVAEDESRSATHCCAFKVTNPAMLPPVKELRKLMRIQGLRCHIIYCQNGIKMNVIPVVASRSQALRYLYVRWGVDLSSMVVFVGECGDTDYEGLLGGVHKTVILKGVCVDARKLHTNRNYPLEHVVPSDSPNLVESEGCNNTNIRATLAPDVSTSNPKLFKNLTRVLCNANTSEGFQPPRDVSLREIKIPHNHLGPPRLGQPPNNHSILAFFAGGDHNHARKSYSDTGKTKITIFKSTTTSQNPKLLRANGSSQVVLMC

>AcSPS4

MAGNEWINGYLEAILDSGAAAIEDQSKPIPVNLRERGNFNPTKYFVEEVVTGVDETDLHRTWIKVVATRNTRERSSRLENMCWRIWHLTRKKKQLEWEDFQRMAHRRWEREQGRRDATEDMSEDLSEGEKGDVLGETMVNESPRKKFQRNFSNLEVWSDNNKEKKLYIVLISLHGLVRGENMELGRDSDTGGQIKYVVELARALARMPGVYRVDLFTRQISSPEVDWSYGEPTEMLTAGADDDADVEESSGAYIVRIPFGPRDKYMRKELLWPYIQEFVDGALAHILNMSKALGEQIGHSLGRNKLEQLLKQGRQSKEDINSTYKIMRRIEAEELSLDVAELVITSTKQEIDEQWGLYDGFDVKLEKVLRARVRRGVNCHGRYMPRMAVIPPGMDFSNVVVQEDTAEADGELTALTTADGSSPKAFPPIWSEMMRFLTNPHKPMILALSRPDPKKNITTLLKAFGECRPLRELANLTLIMGNRDDIDEMSAGNASVLTTVLKLVDKYDLYGQVAFPKHHKQSDVPEIYRLAGKTKGVFINPALVEPFGLTLIEAAAHGLPMVATKNGGPVDIHRALNNGLLVDPHDQEAIANALLKLVSEKNLWHDCRKNGWKNIHLFSWPEHCRTYLTRVAACRMRHPQWQTDTPADEFAAEESLNDSLKDVQDMSLRLSIDGERTSLNESLDYVTATGGGPELQDQVKQLLSRMRKPETKAQDSEGSGKLVDNIASKYPMLRRRRRLIVIALDCYDSDGAPEKKMIRIIQEIFRAVNVVSQTARFSGFALSTAMSMSELKAFLKTGNIQVNEFDALICSSGSEVYYPGTYTQEDGKLYPDPDYATHIDYRWGCDGLKKTIWKLMNSHEGGSSHSKSPIEADVKSSNSHCVSYLIKDLSKAKKVDDMRQKLRMRGLRCHLMYCRNSTRMQVVPLLASRAQALRYLFVRWRLNVSNMYVILGETGDTDYEELISGTHKTLIMKNMVEKGSEELLRAAGSYLKDDIVPEESPLVTYTSGEAKADDIANALKQISKSSPGI

>AcSPS5

MANNEWINGYLEAILDAGSRRSGLRENGYADEGIRSNNSMNNMSIRKRLEDKLRIEKFDDDKGKEEKLFSPTKYFVEEVVNCFDESDLHRTWIKIAWDDAKRLAKRRIEREKGRNDAAEDLSELSEGEKEKGDANQTEPISEKISRINSDMKIWSDDDKSRRLYIVLISLHGLVRGENMELGRDSDTGGQVKYVVELARALANMKGIYRVDLLTRQITSSEIDFSYGEPNEMLSCPSDGSGSCGAYIIRIPCGPRDKYIPKESLWPHIPEFVDGALSHIVNMARAIGEQVDGGKPTWPYVIHGHYADAGEVAARLSGALNVPMVLTGHSLGRNKFEQLLKQGRLSREDINSAYKIMRRIEAEELGLDAAEMVVTSTRQEIEEQWGLYDGFDIKLERKLRVRKRRGVSCLGRYMPRMVVTPPGMDFSYVTTQDSLEGDGDLKSLIGSDRTQNKRHIPPIWSEVMRFFTNPHKPMILALSRPDPKKNVTTLLKAFGECRPLKELANLTLILGNRDDIEDMSNSSSVVLTTVLKLIDKYDLYGQVAYPKHHKQSEVPEIYRLAAKTKGVFINPALVEPFGLTLIEAAAYGLPIVATKNGGPVDILKALNNGLLIDPHDQKAIADALLKLVAEKNLWLECRKNGLKYIHRFSWPEHCRNYLSHVEHCRNRHPTTRLEIMPTPEEPMSESLRDVEDISLKFSIDADFKLNGDLDVPNRQCKLIEALTQMGSSNSPSSTSYCPGRRQALFIIATDCYNSGGMCTETFPLVIKNVMQAAVSNSGKIGFILSTGLTLLETKELLRHCHVNLEDFDAFVCNSGSEMYYPWRDSTADMDYEAHIEYRWPGENVRSMIMRLGRVGDGDGDEYDIMESLDAFSSRCYSYSIKQGSKTRRIDELRQRLRMRGLRCNVIYTRASSLLKVVPLFASRAQALRWAIDLSKMVVFVGERGDTDYEDLLVGLHKTVILRNSVEYGSEMLLRSEESFKREDVVPQDSPRIAFGACYETHDISAALDALQVI

>AcSPS6

MSRRAEEQKKEGIDDAKSSLLLRERGRFSPTRYFVEQVIGFDETDLYRSWVKLEGEEAQRMAKRRLERERGRREATADMSEDLSEGEKGDKVSDLSAHGESNRGRLPRISSVETMEAWVSQQKGKRLLHGLIRGENMELGHDSDTGGQVKYVVELARALGSMPGVYRVDLLTRQVSSPEVDWSYGEPTEMLPPRNSDVLMDEMGESSGAYIIRIPFGPRDKYVPKELLWPHVPEFVDGALNHIIQMSKVLGEQIGSGHPVWPVAIHGHYADAGDAAALLSGALNVPMLFTGHSLGRDKLEQLLRQSRLSKDEINKTYKIMRRIEAEELSLDASEIVITSTRQEIEQQWRLYDGFDPVLERKLRARIRRNVSCYGRIMPRMVVIPPGMEFHHIVPHEGDMDGETEGNEDQPTSPDPPIWPEIMRFFTNPRKPMILALARPDPKKNLTTLVEAFGECRPLRELANLTLIMGNRDDVDEMSSTNSSVLLSILKLIDKYDLYGQVAYPKHHKQSEVPNIYRLAAKTKGVFINPAFIEPFGLTLIEAAAYGLPIVATKNGGPVDIHRALDNGLLVDPHDRQSIADALLKLVADKQLWAKCRQNGLKNIHLFSWPEHCKTYLSRIAACKLRQPWWQRSDDGNENSESDSPSDSWRDIQDISLNLKFSLDGEKNEGSGNADSSLDFEDRKSKLENAVLTWSKGVQKGTQKAGLTEKADQNSTAGKFPALRRRKNIVVIAMDFGAISDLSESIRKIFDAMAKERTEGSIGFILATSFTLSEVQSFLISGGLSPSDFDAFICNSGSDLYYSSLNSEDKPFVVDLYYHSHIEYRWGGEGLRKTLIRWAGSITDKKGENEEQIVTEDEKISTNYCYAFKVQNAGKDPPVKEIRKLMRIQALRCHVIYCQNGNKINVIPVLASRSQALRYLYLRWGVDLSKMVVFVGESGDTDYEGLLGGIHKSVILKGVCSGPTHQLHANRTYPLSDVLPIDSPNIVQAAEECSGADLRTSLGKLEFIKG

>AeSPS1

MAGNDWINSYLEAILDVGPGIDDAKSSLLLRERGRFSPTRYFVEQVIGFDETDLYRSWVKAAATRSPREAEYETRETCAGGFGIWLARKSRFGVLFEMVVTRISGGLKILDVEFCFGYVYTCGYLYEHQRITVLEGEEAQRMAKRRLERERGRREATADMSEDLSEGEKGDTVSDLSAHGESNRATRYTYRVKVSLSLHGLIRGENMELGRDSDTGGQGFGFNARSLYRVDLLTRQVSSPEVDWSYGEPTEMLPPRNSDGLMDEMGESSGAYIIRIPFGPRDKYVPKELLWPHIPEFVDGALNHIIQMSKVLGEQIGSGHPVWPVAIHGHYADAGDAAALLSGALNVPMLFTGHSLGRDKLEQLLRQSRLSKDEINKTYKIMRRIEAEELSLDASEIVITSTRQEIEQQWRLYDGFDPVLERKLRARIRRNVSCYGRFMPRMVVMPPGMEFHHIVPHEGDMDGETEGNEDQPTSPDPPIWPEIVRFFTNPRKPMILALARPDPKKNLATLVEAFGECRPLRELANLTLIMGNRGDVDEMSSTNSSVLLSILKLIDKYDLYGQVAYPKHHKQSDVPDIYRLAAKTKGVFINPAVIEPFGLTLIEAAAYGLPIVATKNGGPVDIHRALDNGLLVDPHDQKSIADALLKLVADKQLWSKCRQNGLKNIYLFSWPEHCKTYLSRIAACKLRQPWWQRSDDGDENSESDSPSDSLRDISLNLKFSLDGEKNEGSGNADSSLEFEDRKSKLENAVLTWSKGFQKGTQKVGLTEKADSNITAGKFPVLRRRKNIIVIAVDFGAISDFSESIRKIFDAVEKERTEGSIGFILATSFTLSEVHSFLISGGLSPSDFDAFICNSGSDLYYSSLNSEDNPFVVDLYYHSHIEYRWGGEGLRKTLIRWTGSINDKKGENEEQIVTEDEKISTNYCYAFKVRNAGKVPPVKEIRKLMRIQAHRCHVIYCQNGNKINVIPVLASRCQALRYLYLRWGMDLSKMVVFVGESGDTDYEGLLGGIHKSVILKGVCSGPTNQLHANRTYPLSDVLPIDSPNIVQAAEECSSADLRTSLLKLGFIKG

>AeSPS2

MAGNEWINGYLEAILDSGAAAIEDQSKPIPVNLRERGNFNPTKYFVEEVVTGVDETDLHRTWIKVVATRNTRERSSRLENMCWRIWHLTRKKKQLEWEDFQRMAHRRWEREQGRRDATEDMSEDLSEGEKGDVLGETMVNESPRKKFQRNFSNLEVWSDNNKEKKLYIVLISLHGLVRGENMELGRDSDTGGQIKYVVELARALARMPGVYRVDLFTRQISSPEVDWSYGEPTEMLTAGADDDADVGESSGAYIVRIPFGPRDKYMRKELLWPYVQEFVDGALAHILNMSKALGEQIGGGQPVWPYVIHGHYADAGDSAALLSGALNVPMVLTGHSLGRNKLEQLLKQGRQSKEDINSTYKIMRRIEAEELSLDVAELVITSTKQEIDEQWGLYDGFDVKLEKVLRARVRRGVNCHGRYMPRMAVIPPGMDFSNVVVQEDTAEADGELTALTTADGSSPKAFPPIWSEMMRFLTNPHKPMILALSRPDPKKNITTLLKAFGECRPLRELANLTLIMGNRDDIDEMSAGNASVLITVLKLVDKYDLYGQVAFPKHHKQSDVPEIYRLAGKTKGVFINPALVEPFGLTLIEAAAHGLPMVATKNGGPVDIHRALNNGLLVDPHDQEAIANALLKLVSEKNLWHDCRKNGWKNIHLFSWPEHCRTYLTRVAACRMRHPQWQTDTPADEFAAEESLNDSLKDVQDMSLRLSVDGERTSLNESLDHVTATGGGPELQDQVKQVVSRMRKPETKAQDSEGSGKLVDNIASKYPMLRRRRRLIVIALDCYDSDGAPEKKMIRIIQEIFRAVNVVSQTARFSGFALSTAMSMSELKAFLKTGNIQVNEFDALICSSGSEVYYPGTYTQEDGKLYPDPDYATHIDYRWGCDGLKKTIWKLMNSHEGGSSHSKSPIEEDVKSSNSHCVSYLIKDLSKAKKVDDMRQKLRMRGLRCHLMYCRNSTRMQVVPLLASRAQALRYLFVRWRLNVSNMYVILGETGDTDYEELISGTHKTLIMKNMVEKGSEELLRAAGSYLKDDIVPEESPLVTYTSGEAKADDIANALKQISKSSPGI

>AeSPS3

MANNEWINGYLEAILDAGSRRSGLRENGYADEGIRSNNSMNNMSIRKRFEDKLRIEKFDDDKGKEEKLFSPTKYFVEEVVNCFDESDLHRTWIKVIATRNTRERSNRLENMCWRIWHLARKKKQAAKRRIEREKGRNDAAEDLSELSEGEKEKGDANQTEPISEKISRINSDMKIWSDDDKSRRLYIVLISLHGLVRGENMELGRDSDTGGQVKYVVELARALANMKGIYRVDLLTRQITSSEIDFSYGEPNEMLSCPSDGSGSCGAYIIRIPCGPRDKYIPKESLWPHIPEFVDGALSHIVNMARAIGEQVDGGKPTWPYVIHGHYADAGEVAARLSGALNVPMVLTGHSLGRNKFEQLLKQGRLSREDINSAYKIMRRIEAEELGLDAAEMVVTSTRQEIEEQWGLYDGFDIKLERKLRVRKRRGVSCLGRYMPRMVVTPPGMDFSYVTTQDSLEGDGDLKSLIGSDRTQNKRHIPPIWSEVMRFFTNPHKPMILALSRPDPKKNVTTLLKAFGECRPLKELANLTLILGNRDDIEDMSNSSSVVLTTVLKLIDKYDLYGQVAYPKHHKQSEVPEIYRLAAKTKGVFINPALVEPFGLTLIEAAAYGLPIVATKNGGPVDILKALNNGLLIDPHDQKAIADALLKLVAEKHLWLECRKNGLKYIHRFSWPEHCRNYLSHVEHCRNRHPTTRLEIMPTPEEPMSESLRDVEDISLKFSIDADFKLNGDLDVPNRQCKLIEALTQMGSSNSPSSTSYCPGRRQALFIIATDCYNRGGMCTETFPLVIKNVMQAAVSNSGKIGFILSTGLTLLETKEMLRHCHVNLEDFDAFVCNSGSEMYYPWRDSTADMDYEAHIEYRWPGENVRSMIMRLGRVGDGDGDGDDIMESTDAFSSRCYSYSIKQGSKTRRIDELRQRLRMRGLRCNVIYTRTSSRLKVVPLFASRAQALRYLSVRWAIDLSKMVVFVGERGDTDYEDLLVGLHKTVILRNSVEYGSEMLLRSEESFKREDVVPQDSPRIAFGACYETHDISAALDALQVI
